# 1,2-Hydrogen atom transfer of aminyl radicals under photoredox catalysis for the synthesis of α-amino phosphine oxides

**DOI:** 10.1039/d5sc00268k

**Published:** 2025-11-05

**Authors:** Ailin Pan, Madeline E. Rotella, Yamiao Meng, Xun Tian, Shengzu Duan, Yonggang Jiang, Guogang Deng, Bart Limburg, Hongbin Zhang, Marisa C. Kozlowski, Patrick J. Walsh, Xiaodong Yang

**Affiliations:** a Key Laboratory of Medicinal Chemistry for Natural Resource, Ministry of Education, Yunnan Key Laboratory of Research and Development for Natural Products, School of Pharmacy, Yunnan University, Southwest United Graduate School Kunming 650500 People's Republic of China xdyang@ynu.edu.cn zhanghb@ynu.edu.cn; b Roy and Diana Vagelos Laboratories, Penn/Merck Laboratory for High-Throughput Experimentation, Department of Chemistry, University of Pennsylvania Philadelphia PA 19104 USA pwalsh@sas.upenn.edu marisa@sas.upenn.edu; c Department of Inorganic and Organic Chemistry, University of Barcelona Carrer Martí i Franquès 1 08028 Barcelona Spain

## Abstract

α-Amino phosphorus compounds are significant structural motifs in natural products, pharmaceuticals and organocatalysis. The construction of such motifs by hydrogen atom transfer (HAT), however, is rare. Herein, we describe a photocatalytic net 1,2-HAT of nitrogen-centered radicals for the formation of C(sp^3^)–P bonds. This net 1,2-HAT proceeds *via* deprotonation of N-centered radicals without the requirement of external strong bases, instead being facilitated by the benzoate anion generated during the reaction. This process enables the synthesis of α-amino phosphine oxides bearing various functional groups under mild conditions. The gram-scale synthesis demonstrates the scalability of the net 1,2-HAT rearrangement/radical–radical coupling reaction, which has been used in the preparation of an antitumor agent. Mechanistic investigations, including radical trapping experiments, Stern–Volmer fluorescence quenching experiments, cyclic voltammogram studies, cross-over experiments, KIE studies and DFT calculations support a net 1,2-HAT pathway.

## Abstract 2

Photocatalytic 1,2-HAT of N-centered radicals leads to C-centered α-amino radicals, with trapping by phosphine oxides to access α-amino phosphine oxides. Mechanistic experiments and DFT calculations support a 1,2-HAT pathway.

## Graphical abstract

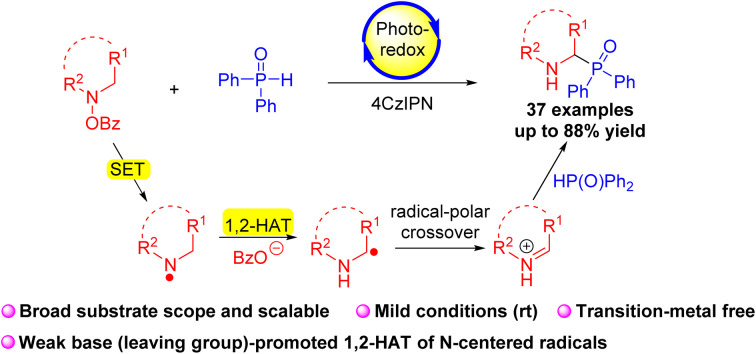

## Introduction

Organophosphorus compounds are common structural motifs in natural products and play vital roles in pharmaceuticals and organocatalysis.^1–6^ As a subclass of these, α-aminophosphonates and their derivatives are of interest in the pharmaceutical and agricultural industries, because they possess unique biological activities allowing uses as herbicides,^7^ cancer cell inhibitors,^8^ and virus inhibitors.^9–12^ Moreover, α-amino phosphorus compounds can be efficient catalysts^13^ in organic synthesis and they are also used as P,N ligands^14,15^ in organometallic chemistry and catalysis. As such, more efficient and eco-friendly methods to prepare α-amino phosphorus compounds remain in demand.

**Scheme 1:**
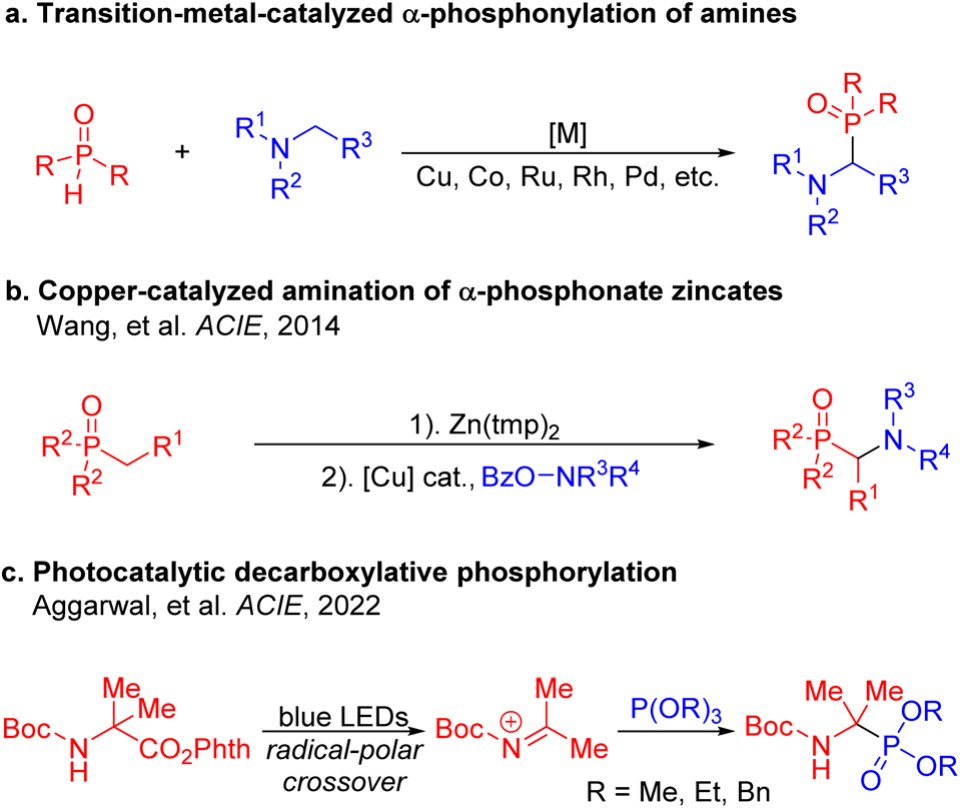
Representative approaches to the synthesis of α-amino phosphorus derivatives. (a) Transition-metal-catalyzed α-phosphonylation of amines. (b) Copper-catalyzed amination of α-phosphonate zincates. (c) Photocatalytic decarboxylative phosphorylation.

Given the utility of α-amino phosphorus compounds, various approaches have been developed for their preparation. The Kabachnik–Fields reaction^16,17^ (hydrophosphonylation of imines), which involves the reaction of an amine, an aldehyde or a ketone, and a phosphinic acid, represents one of the most common methods toward α-amino phosphorus compounds. Alternatively, the α-phosphonylation of amines to prepare α-amino phosphorus compounds has been demonstrated with various transition-metal catalysts, including Cu, Co, Rh, Ru and Pd (Scheme 1a). In some cases, these reactions are conducted with high temperatures and pressures.^18–26^ In 2014, Wang and co-workers developed an approach to α-aminophosphine oxides using copper-catalyzed electrophilic α-amination of α-phosphonate zincates to form C–N bonds (Scheme 1b).^27^ Recently, progress has been made in the formation of C–P bonds through catalytic reactions employing light and electrochemistry.^28–32^ For example, Aggarwal's team utilized photoredox catalysis^33–37^ to facilitate the decarboxylation of α-amino acid derivatives. The resulting alkyl radicals were oxidized to *N*-acyliminium ions, ultimately being captured using triphenyl phosphite (Scheme 1c).^38^

**Scheme 2:**
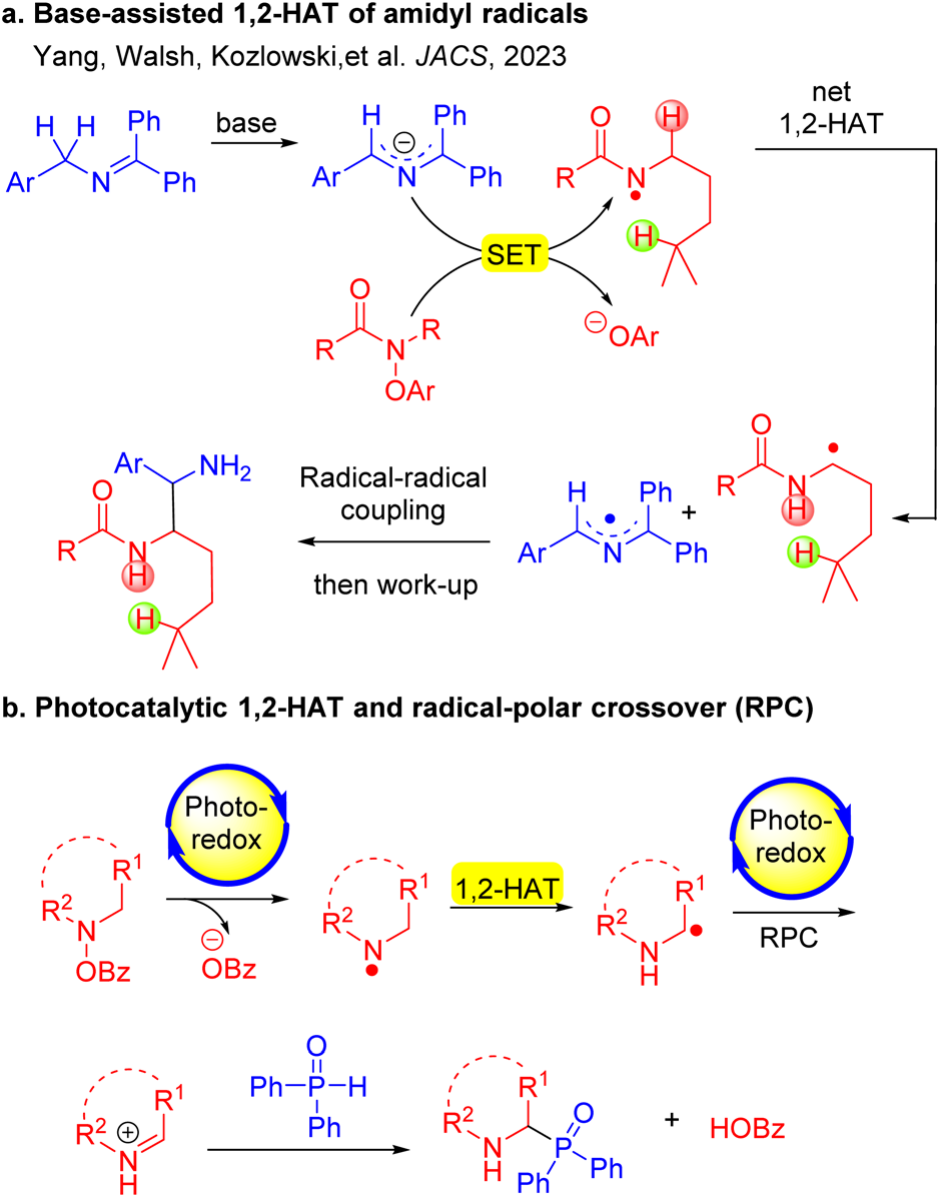
1,2-HAT of nitrogen-centered radicals. (a) Base-assisted net 1,2-HAT of amidyl radicals. (b) Photoredox initiated net 1,2-HAT of nitrogen-centered radicals followed by radical-polar crossover (this work).

Hydrogen atom transfer (HAT) processes are strategically important for achieving regioselective functionalization of C–H bonds. Heteroatom (O,N)-centered radicals, which generally undergo intramolecular 1,5-HAT reactions (Hofmann–Löffler–Freytag reaction),^39^ produce translocated C-centered radicals. 1,2-HAT is less frequently observed compared to the intramolecular 1,5-HAT process. This is because the 1,5-HAT process is favored by a chair-type six-membered ring transition state, resulting in a lower energy^40^ requirement for hydrogen atom migration. Recently, there have been a limited number of reports highlighting the net 1,2-HAT of O-centered radicals (O·) in the literature.^41–43^ Notably, 1,2-HAT reactions of nitrogen-centered radicals (N·) exhibit great potential in synthesis, but are rarely observed. Recently, our team developed a method to coax amidyl radicals to undergo net 1,2-HAT processes to deliver α-amino carbon-centered radicals (C·) (Scheme 2a). The newly formed C-centered radicals coupled with 2-azaallyl radicals^37,44–46^ to generate 1,2-diamine derivatives. Mechanistic experiments and density functional theory (DFT) calculations revealed that this 1,2-HAT is a base-assisted, stepwise process (Scheme 2a).^47^ Subsequently, the Chen group reported base-promoted 1,2-HAT processes to prepare fluorinated amines.^48,49^

Considering the significance of α-amino phosphorus compounds, we hypothesized that our newly developed 1,2-HAT of nitrogen-centered radicals (N˙) could lead to a new approach to form C–P bonds. Previous reports on the reaction between diphenylphosphine oxide and *O*-acylhydroxylamines resulted in P–N bond formation to afford phosphinic amides 
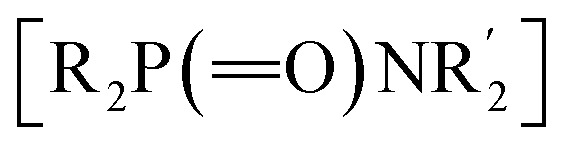
.^50,51^ However, to the best of our knowledge, there are no reports on reactions of these coupling partners to access α-amino phosphorus derivatives.

Herein, we report a photocatalytic net 1,2-HAT of nitrogen-centered radicals for the formation of C(sp^3^)–P bonds (Scheme 2b). Diverging from former net 1,2-HAT protocols, the present approach uniquely employs the benzoate anion—functioning dually as a leaving group and a base promoter—to facilitate the reaction. Specifically, hydroxyl amines were found to undergo a photocatalytic single electron transfer (SET) process to generate N-centered radicals.^52^ The N-centered radical undergoes a benzoate-promoted isomerization through a net 1,2-HAT process to generate a C-centered α-amino radical, which undergoes SET with the photoredox catalyst (4CzIPN) to form the iminium ion in a radical-polar crossover (RPC). A phosphine oxide participates in an intermolecular coupling with the resultant iminium to furnish α-amino phosphorus products in good yields. This method enables the synthesis of α-amino phosphine oxides bearing various functional groups (37 examples, up to 88% yield). Mechanistic investigations, including radical trapping experiments, Stern–Volmer fluorescence quenching experiments, cyclic voltammogram studies, cross-over experiments, KIE studies and DFT calculations, support a net 1,2-HAT pathway. Notably, this net 1,2-HAT reaction takes place employing a simple organic photoredox catalyst without the addition of base or transition metals.

## Results and discussion

We selected 4-benzoyloxy-morpholine (1a) as the initial substrate because the morpholine group is common in bioactive molecules. Reaction of 1a was performed with diphenylphosphine oxide 2a in the presence of photoredox catalysts (2 mol%) in PhCF_3_ at room temperature under irradiation with 450 nm blue LEDs for 24 h. Initially, a series of commercial photoredox catalysts including iridium, ruthenium and organic photoredox catalysts were evaluated (Table 1, entries 1–7). Among them, the organic photoredox catalyst 4CzIPN generated the α-amino phosphine oxide 3aa in 79% assay yield (AY, as determined by ^1^H NMR integration against an internal standard, entry 7), while other iridium and ruthenium photoredox catalysts resulted in product 3aa in lower AY (up to 72%) or no reaction. Using 4CzIPN as photoredox catalyst, we examined a series of solvents including THF, DCE, MeCN, DMF, DMSO and toluene (entries 8–14). However, these solvents led to lower AY (up to 64%) or no reaction. To improve the solubility of 4CzIPN, we tested mixed solvent systems (see the SI for details). Among them, the reaction performed best in 1 : 1 MeCN : PhCF_3_ (83% AY, entry 15). Reducing the photocatalyst loading from 2 to 1 mol% afforded only 51% AY (entry 16). In contrast, raising the photoredox catalyst to 3 mol% provided the desired product 3aa in 85% AY and 81% isolated yield (entry 17). Conducting the reaction using 4 mol% photoredox catalyst led to a slight decrease to 82% AY (entry 18). Finally, control experiments (entries 19 and 20), which confirmed that both photoredox catalyst 4CzIPN and blue LED irradiation are required to form product 3aa. Based on these results, the optimal conditions for the net 1,2-HAT are those in entry 17 of Table 1.

**Table 1:** Optimization of the reaction conditions^a^^,^^b^

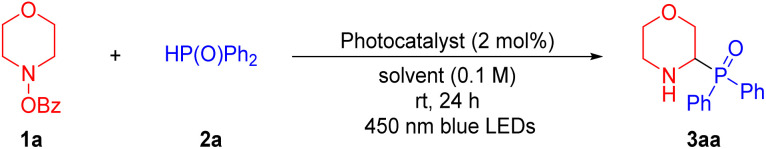			
Entry	Photocatalyst	Solvent	Yield (%)
1	Ir(ppy)_2_(bpy)PF_6_	PhCF_3_	Trace
2	Ir(ppy)_3_	PhCF_3_	0
3	Ir(*p*-tBu-ppy)_3_	PhCF_3_	72
4	Ir[dF(CF_3_)ppy]_2_(dtbpy)PF_6_	PhCF_3_	48
5	Ir(dtbbpy)(ppy)_2_PF_6_	PhCF_3_	69
6	Ru(bpy)_3_(PF_6_)_2_	PhCF_3_	47
7	4CzIPN	PhCF_3_	79
8	4CzIPN	THF	0
9	4CzIPN	DCM	35
10	4CzIPN	DCE	64
11	4CzIPN	MeCN	50
12	4CzIPN	DMF	0
13	4CzIPN	DMSO	0
14	4CzIPN	Toluene	Trace
15	4CzIPN	MeCN : PhCF_3_ = 1 : 1	83
16	4CzIPN (1 mol%)	MeCN : PhCF_3_ = 1 : 1	51
**17**	**4CzIPN (3 mol%)**	**MeCN : PhCF**_**3**_ **= 1 : 1**	**85 (81)** c
18	4CzIPN (4 mol%)	MeCN : PhCF_3_ = 1 : 1	82
19	—	MeCN : PhCF_3_ = 1 : 1	0
20^d^	4CzIPN	MeCN : PhCF_3_ = 1 : 1	0

a Reaction conditions: 1a (0.2 mmol, 2.0 equiv.), 2a (0.1 mmol, 1.0 equiv.), photoredox catalyst (2 mol%), rt, 24 h, under blue LEDs (450 nm).

b Assay yields determined by ^1^H NMR spectroscopy of the unpurified reaction mixtures using CH_2_Br_2_ as an internal standard.

c Isolated yield.

d Without blue LEDs.

With effective α-amino phosphorylation conditions identified (Table 1, entry 17), we evaluated the scope of the phosphine oxide substrates. As shown in Table 2, a wide range of diarylphosphine oxides with different aryl groups delivered the α-amino phosphine oxides in moderate to good yields. Diarylphosphine oxides with electron-donating groups at the 4-position, such as 4-Me (2b), 4-^*t*^Bu (2c), 4-Ph (2d), 4-OMe (2e) and 4-SMe (2f), reacted with 4-benzoyloxy-morpholine 1a to generate α-amino phosphorylation products 3ab, 3ac, 3ad, 3ae and 3af in 45–78% yields, respectively. Diarylphosphine oxides with electronegative and electron-withdrawing groups at the 4-position, such as 4-F (2g), 4-Cl (2h) and 4-CF_3_ (2i) furnished products 3ag, 3ah and 3ai in 88%, 76% and 56% yields, respectively. Diarylphosphine oxides with groups at the 3-position, such as 3-Me (2j), 3-F (2k), 3-Cl (2l) and 3-CF_3_ (2m) led to products 3aj, 3ak, 3al and 3am in 47–63% yields. Diarylphosphine oxides containing disubstituted aryl groups, such as 3,5-di-Me (2n), 3,5-di-F (2o) and 3-F-4-Me (2p) gave products 3an, 3ao and 3ap in 42–61% yields. Furthermore, the structure of product 3ao was confirmed by X-ray crystallography (CCDC 2293469; see the SI for details). The π-extended 2-naphthyl substrate (2q) afforded product 3aq in 60% yield. It is noteworthy that this method was also well-suited for medicinally relevant heterocyclic derivatives. Diarylphosphine oxides bearing benzofuranyl (2r), piperonyl (2s) and 2-thiophenyl (2t) substituents furnished the corresponding products 3ar, 3as and 3at in 56–70% yields. Finally, when diarylphosphine oxides containing two different aryl groups (2u and 2v) were employed, the products 3au and 3av were formed in 55% and 50% yields, respectively, with no diastereoselectivity (dr = 1 : 1).

**Table 2:** Scope of phosphine oxides 2^a^^,^^b^

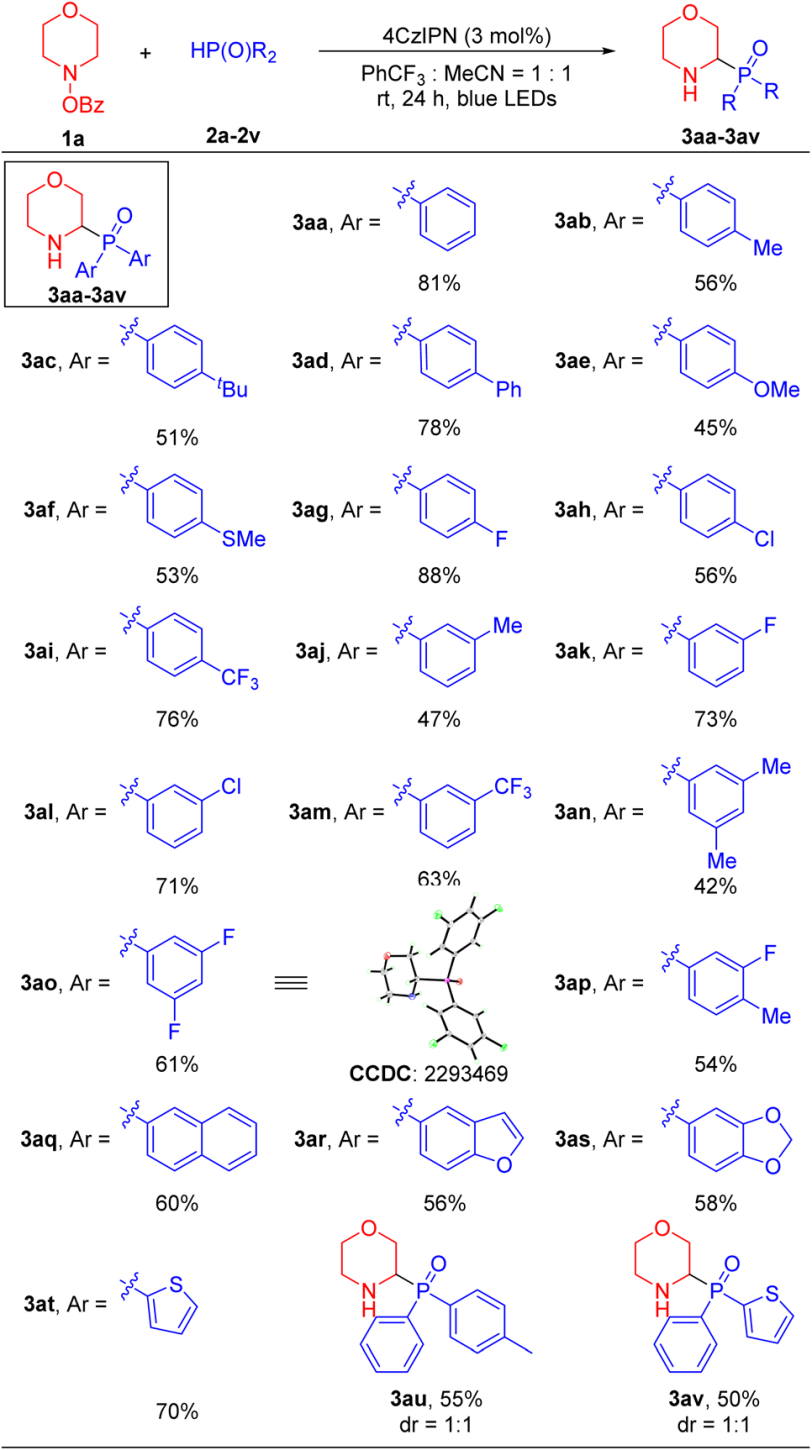

a Reactions were conducted on a 0.4 mmol scale using 2.0 equiv. 1a, 1.0 equiv. 2 and photoredox catalyst (3 mol%) at 0.1 M.

b Yields of isolated products after chromatographic purification.

Next, we evaluated the ability of the net 1,2-HAT radical reaction to accommodate various hydroxylamine derivatives, which were easily synthesized from the corresponding amines and benzoyl peroxide^53–55^ (see the SI for details). Generally, a wide range of cyclic and acyclic amines were compatible with our method, generating the α-amino phosphine oxides in moderate to good yields (Table 3). Cyclic amines including pyrrolidine (1b), piperidine (1c), thiomorpholine (1d), hexamethyleneimine (1e), 4,4-dimethylpiperidine (1f), 4-piperidone ethylene ketal (1g) and *cis*-octahydroisoindole (1h), reacted with diphenylphosphine oxide 2a to afford the desired products 3ba, 3ca, 3da, 3ea, 3fa, 3ga and 3ha in 47–81% yields. Furthermore, acyclic dialkylamino groups, such as dimethylamine (1i), diethylamine (1j), isopropylmethylamine (1k), dibutylamine (1l), *N*-methyl-cyclohexylamine (1m) and *N*-methyl-4-amino-tetrahydropyran (1n), were also suitable partners, delivering the corresponding products 3ia, 3ja, 3ka, 3la, 3ma and 3na in 43–60% yields. It is noteworthy that in the cases of 3ka and 3am the aminyl radical intermediate undergoes deprotonation preferentially at the least sterically hindered position, giving the higher energy primary radical over the more stable tertiary radical. Finally, in order to showcase the synthetic utility of our approach and further probe selectivity in the net 1,2-HAT, we carried out the phosphorylation of druglike molecules. For instance, hydroxylamine derivatives from antidepressant nortriptyline^56,57^ (1o) and fluoxetine^58,59^ (1p) underwent net 1,2-HAT reactions to afford products containing the α-phosphorylation fragment in 3oa and 3pa in 43% and 45% yields, respectively. Here again, selective deprotonation of the α-aminyl radical at the methyl group over the secondary C–H positions was observed.

**Table 3:** Scope of hydroxylamine derivatives 1^a^^,^^b^

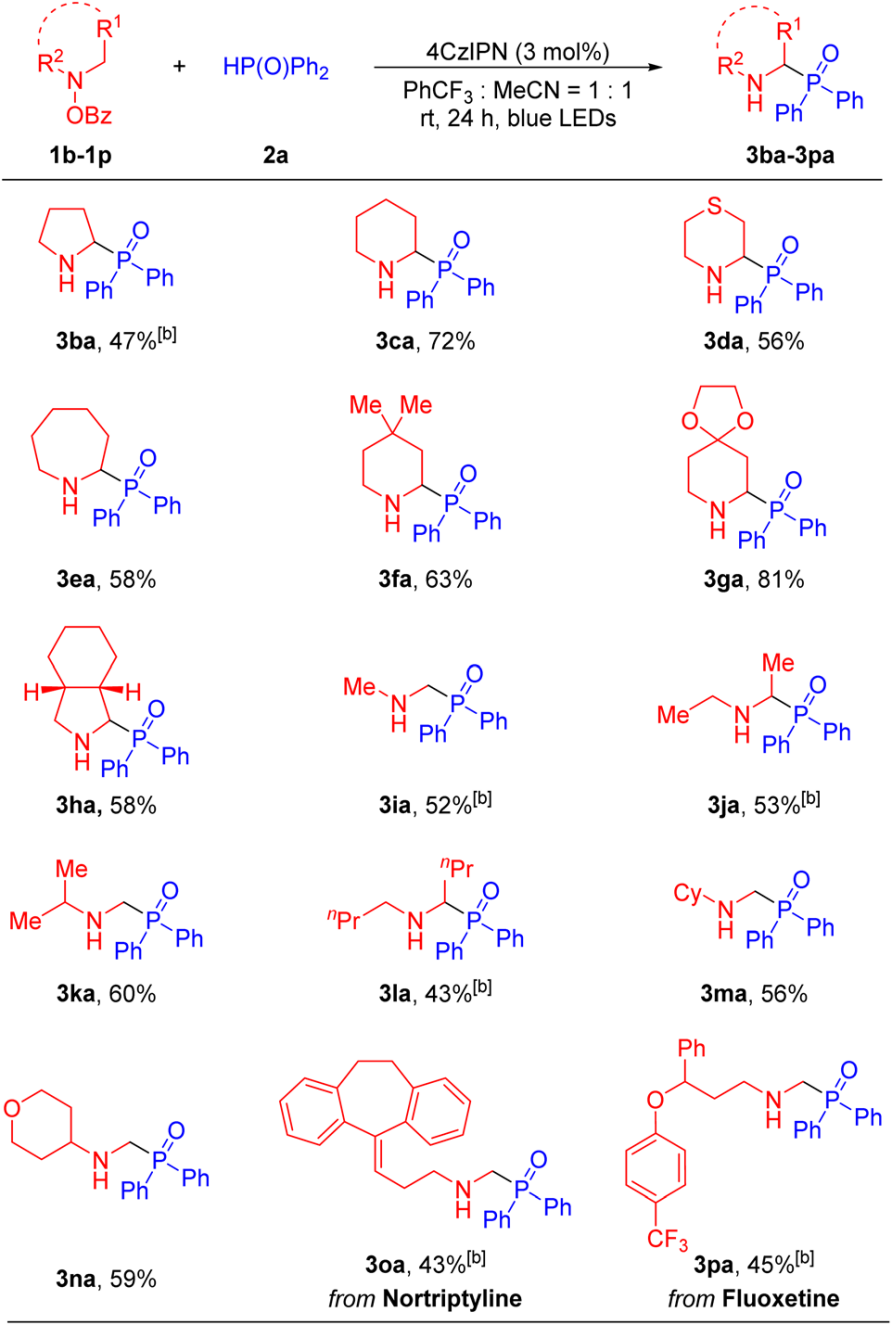

a Reactions were conducted on a 0.4 mmol scale using 2.0 equiv. 1, 1.0 equiv. 2a and photoredox catalyst (3 mol%) at 0.1 M.

b Yield of isolated product after chromatographic purification.

c 3.0 equiv. 1 was used and 3.0 equiv. NaHCO_3_ was used.

**Scheme 3:**
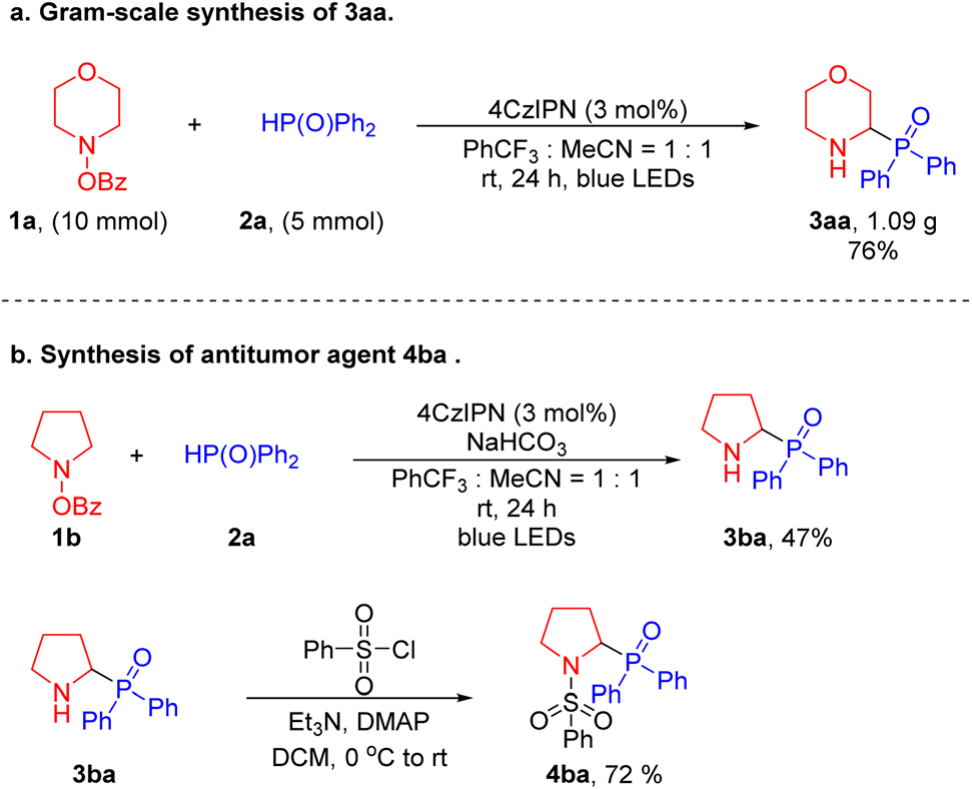
Gram-scale synthesis and product derivatization. (a) Gram-scale synthesis of 3aa. (b) Preparation of an antitumor agent (4ba).

To test the scalability of this net 1,2-HAT radical coupling process, we carried out a gram-scale synthesis (Scheme 3a). Reaction of 4-benzoyloxy-morpholine 1a (10 mmol) was performed with diphenylphosphine oxide 2a (5 mmol) under the standard conditions for 24 hours to provide 1.09 g of the target product 3aa with a yield of 76%. This result is comparable to the 81% yield achieved on a 0.1 mmol scale (Table 1). To establish the synthetic utility of our method, we employed a two-step transformation to synthesize the antitumor agent 4ba.^60^ First, α-amino phosphine oxide 3ba was afforded in 47% yield under the standard conditions. Then, 3ba was treated with benzenesulfonyl chloride under basic conditions to generate the active 4ba in 72% yield. The synthesis of this bioactive product illustrates the practical application of our method to prepare pharmaceutically relevant substances (Scheme 3b).

**Scheme 4:**
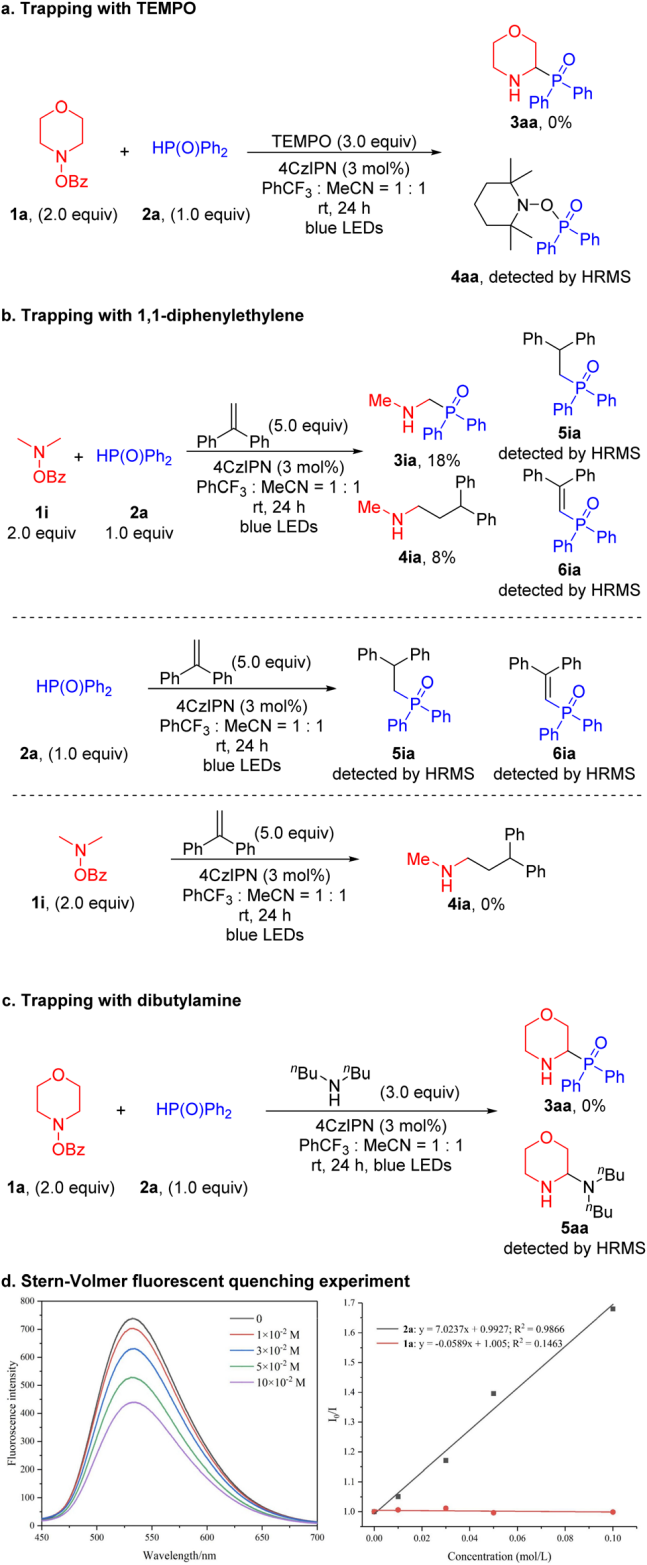
Mechanistic studies. (a) Trapping with TEMPO. (b) Trapping with 1,1-diphenylethylene. (c) Trapping with dibutylamine. (d) Stern–Volmer fluorescent quenching experiments. Quenching of the 4CzIPN emission (4.0 × 10^−4^ M in MeCN : PhCF_3_ = 1 : 1) in the presence of increasing amounts of 2a.

To elucidate the reaction mechanism, we undertook a series of investigative experiments. First, a radical trapping experiment using 2,2,6,6-tetramethyl piperidine-1-oxyl (TEMPO, 3.0 equiv.) was conducted with 1a and 2a as substrates (Scheme 4a). The reaction failed to generate the product 3aa and the oxidized phosphine oxide trapped with TEMPO (4aa) was detected by HRMS. Product 4aa is not stable to silica gel and decomposed during isolation. Additionally, when 2a was used and no 1a was added, 4aa was again detected (see the SI for details). Based on the work of Doyle, we conducted trapping experiments using 1i and 2a as substrates, along with the radical trap 1,1-diphenylethylene (5.0 equiv.) (Scheme 4b).^61^ The yield of the expected product 3ia was only 18%. Trace products formed from addition of a phosphorus-based intermediate to the 1,1-diphenlethylene (5ia and 6ia) were detected by HRMS, further supporting generation of a P-centered radical. Given that only trapping of the P-centered radical is observed and no trapped α-amino radical is detected, we hypothesize that the photoredox catalyst initiates the process through oxidation of the phosphine oxide (as discussed below). We also isolated the product formed from the α-amino radical addition to diphenylethylene (4ia, 8% yield). The reaction between 2a and 1,1-diphenylethylene under the standard conditions, but in the absence of amine, also led to coupling products 5ia and 6ia (detected by HRMS). However, the reaction between 1i and 1,1-diphenylethylene in the absence of phosphine oxide did not generate the trapping product 4ia. This result supports the photoredox catalyst first oxidizing the phosphorus coupling partner and then reducing the amine derivative. Furthermore, when dibutylamine, a nucleophile with low reactivity toward radical reactions, was added, the corresponding aminal compound 5aa was generated and detected by HRMS (Scheme 4c). This result supports the presence of an iminium cation as an intermediate.

To further explore this mechanistic pathway, a series of Stern–Volmer quenching experiments were performed (Scheme 4d). These experiments also revealed that the excited state of 4CzIPN* was quenched by the phosphorus coupling partner rather than the amine derivative (see the SI for details). Additionally, the photoredox catalyst 4CzIPN with an *E*_1/2_(*P/*P*^−^) of +1.35 V (*vs.* SCE) can oxidize 2a (*E*_ox_ = +1.0 V *vs.* SCE).^62,63^ On the other hand, the photoredox catalyst 4CzIPN with an *E*_1/2_(*P*^+^/P*) of −1.04 V (*vs.* SCE)^62^ is unlikely to reduce 1a (*E*_re_ = −2.04 V *vs.* SCE, see the SI for more details). This result is also consistent with a photocatalytic cycle initiated with the oxidation of 2a.

**Scheme 5:**
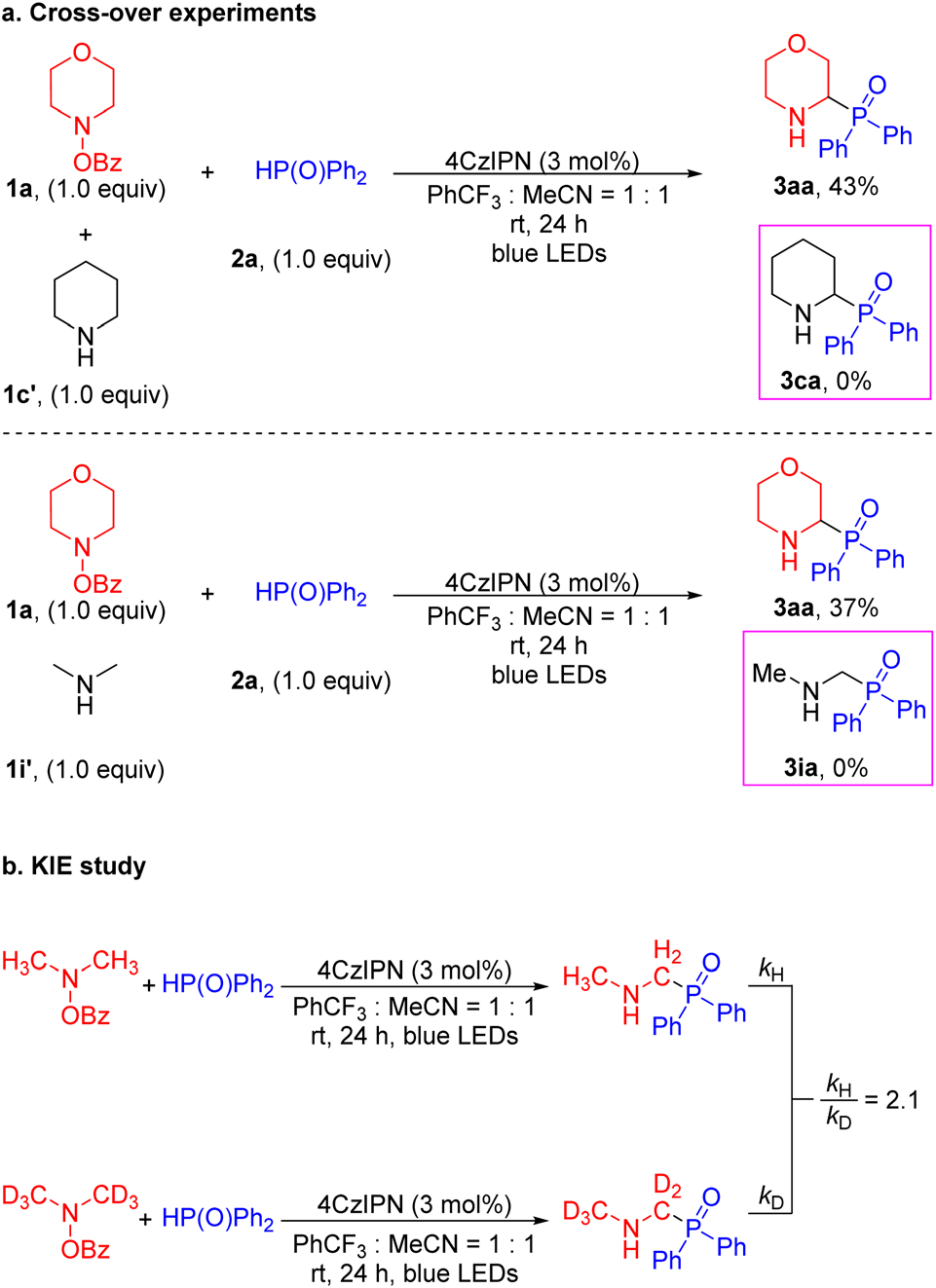
Mechanistic experiments. (a) Cross-over experiments. (b) KIE study.

It is known that the α-amino C–H bonds are generally weaker than other C–H bonds and the resulting radicals are stabilized relative to isolated C-centered radicals.^47,64–68^ With this in mind, we probed whether the HAT process was an intra- or intermolecular reaction by conducting cross-over experiments (Scheme 5a). In the event, treatment of a 1 : 1 mixture of 4-benzoyloxy-morpholine 1a and piperidine 1c′ in the presence of diphenylphosphine oxide 2a under the standard conditions led to the morpholine-derived product 3aa (43% yield) without the generation of the cross-over piperidinyl-containing product 3ca (Scheme 5a). Similarly, reaction of 4-benzoyloxy-morpholine 1a and dimethylamine 1i′ with 2a furnished the product 3aa (37% yield) without the cross-over product 3ia. The cross-over experiments verified that the generation of the α-amino radical was an intramolecular process. Moreover, additional evidence was obtained by examining the parallel kinetic isotope effect (KIE). A KIE value of 2.1 was measured, consistent with removal of the α-amino C–H/D contributing to the rate-limiting step (Scheme 5b).^69^ This observation leads us to propose a low barrier for cleavage of the N–O bond.

**Scheme 6:**
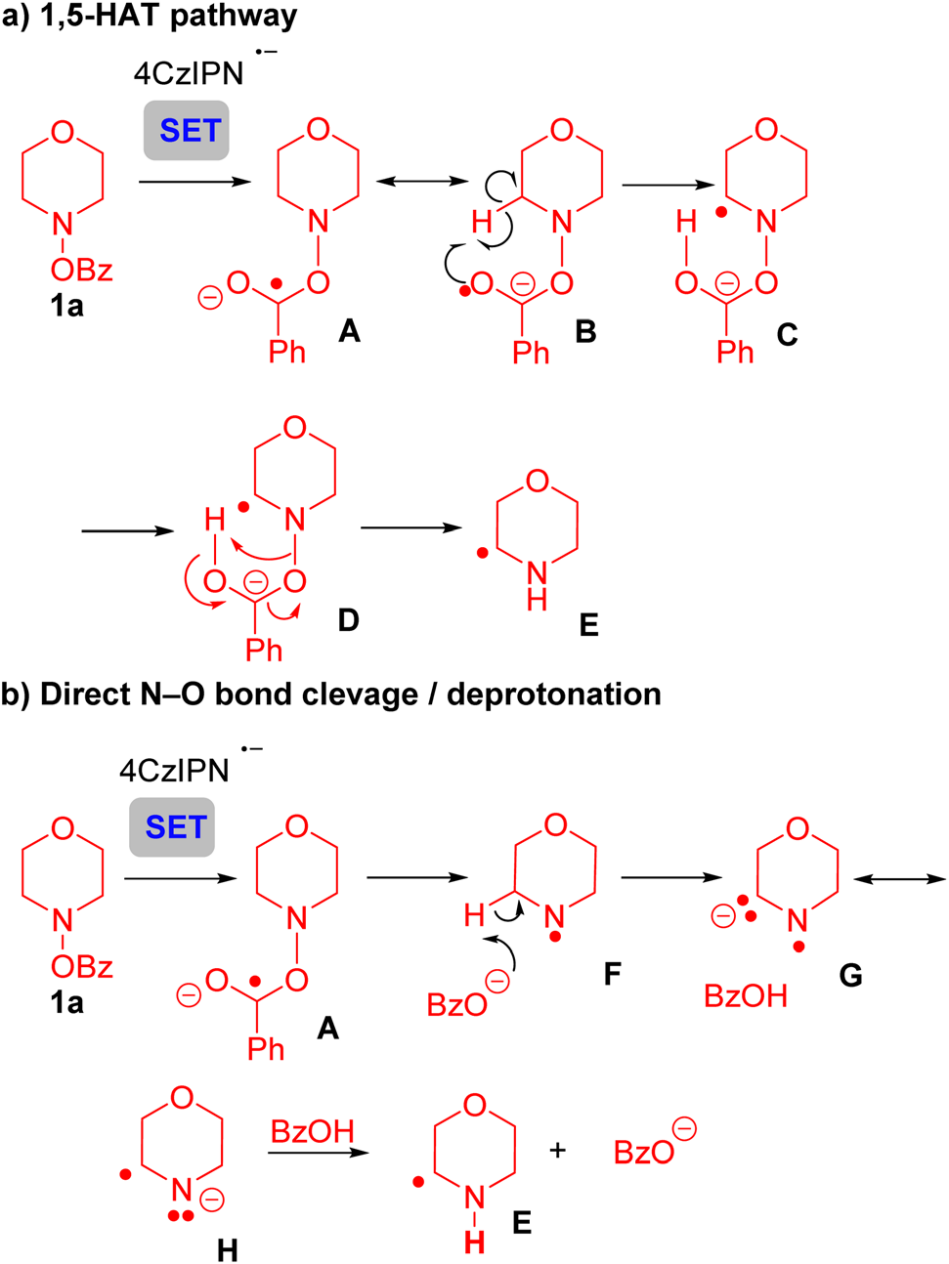
Overview of the mechanism. (a) 1,5-HAT pathway. (b) Direct N–O bond cleavage/deprotonation.

To further probe the mechanism of this transformation, we turned to density functional theory (DFT) calculations [UM06/6-311+G(d,p)-CPCM(acetonitrile)//UB3LYP/6-31G(d),^70–76^ see SI for full computational details]. Given the KIE of 2.1 (Scheme 5) we initially considered two mechanisms that could account for these results. Initially, we explored the formation of product 3aa*via* a 1,5-HAT process (Scheme 6a and Fig. S12). Here, it was envisioned that the reduced photoredox catalyst underwent SET with the *N*-benzyloxy substrate 1a to give the reduced intermediate A and its resonance form B. Intermediate B, with radical character on the oxygen could be envisioned to undergo a 1,5-HAT to give C. Intermediate C might undergo N–O bond cleavage with proton transfer to generate the α-amino radical. While the transition state for the transfer of a hydrogen atom from the α-carbon directly to the benzoate oxygen could be located with the bonds frozen, the removal of the constraints led to the N–O bond being cleaved directly after SET (Fig. S13). We attribute this observation to the lability of the N–O bond as well as the unfavorable, rigid nature of the 1,5-HAT transition state. Similarly, transfer of the hydrogen atom to the nitrogen with the N–O bond intact (C-TS-E, Fig. S14) could not be located without the bonds constrained. Thus, the 1,5-HAT pathway was discounted.

The second pathway, a net 1,2-HAT process, yields product 3aa, as outlined in Scheme 6b. Following SET from the photocatalyst to substrate 1a, radical anion A is again formed. N–O bond rupture of A gives the N-centered radical F and benzoate. Deprotonation of radical F by benzoate generates the resonance stabilized anion G/H. Reprotonation at nitrogen forms the α-amino radical E.

**Scheme 7:**
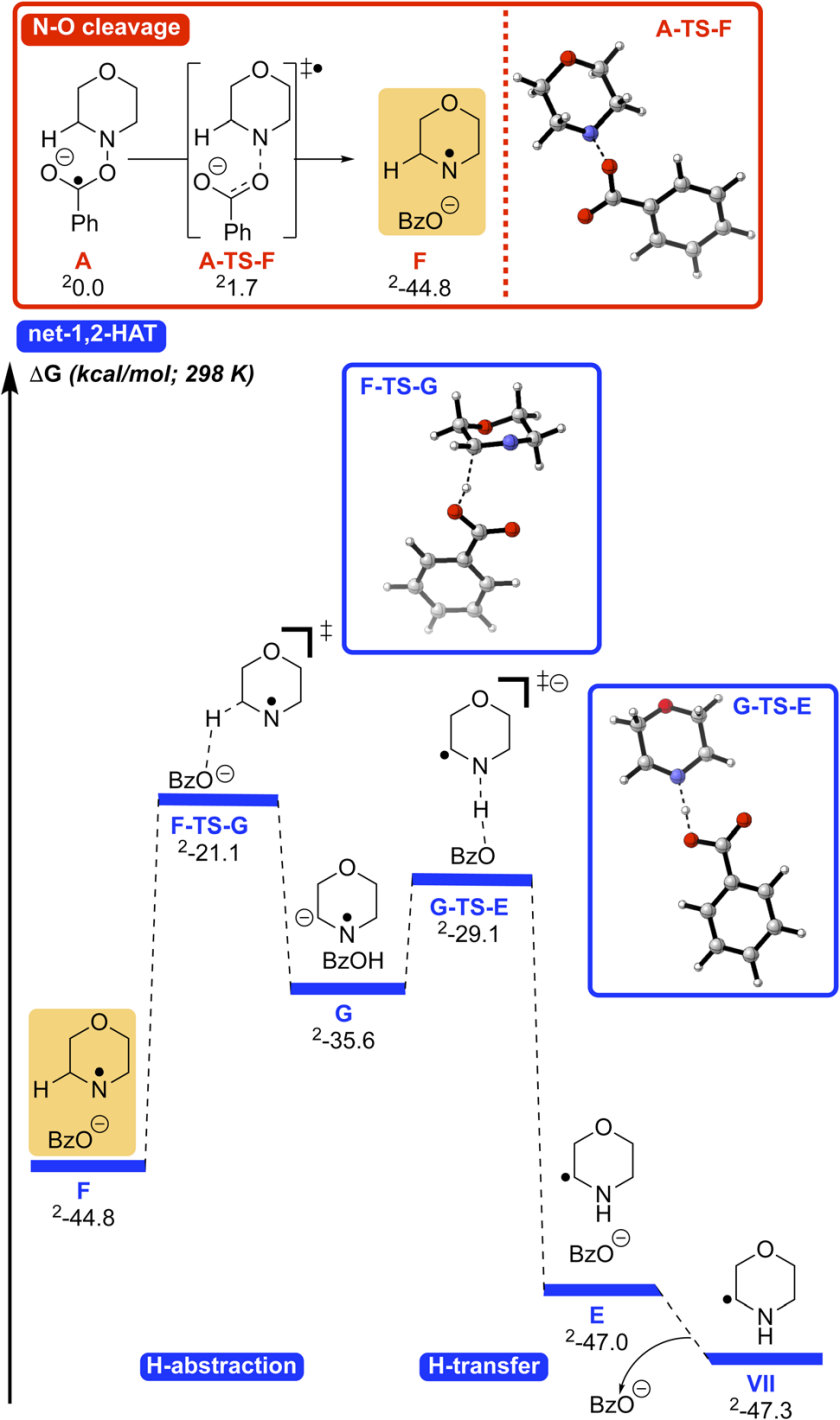
Generation of α-amino C-centered radical intermediate VII*via* N–O cleavage (red) followed by base-assisted, stepwise 1,2-HAT of F (blue). Free energies were computed using UM06-D3/6-311+G(d,p)-CPCM(acetonitrile)//UM06-D3/6-31+G(d,p)-CPCM(acetonitrile).

The energetics of a direct 1,2-HAT (Fig. S15) *vs.* base assisted net 1,2-HAT (Scheme 6a) were compared using DFT calculations. Both processes begin with species 1a′ undergoing facile N–O cleavage (Scheme 7, top, A-TS-F, 1.7 kcal mol^−1^) to generate N-centered radical F and the benzoate anion (−44.8 kcal mol^−1^). Dissociation of the benzoate anion followed by direct-1,2-HAT *via* the 3-centered transition state (F1-TS-VII, Fig. S15) has a high barrier of 42.8 kcal mol^−1^ as expected.^48^ Consequently, we explored the formation of the C-centered radical VII by an indirect, base-assisted-1,2-HAT, similar to our previous findings.^48^ In this pathway (Scheme 7, bottom), the benzoate anion deprotonates the C–H bond alpha to the N-centered radical *via*F-TS-G (barrier of 23.7 kcal mol^−1^) to form radical anion G. Subsequent transfer of the H atom to the nitrogen *via*G-TS-E is facile (barrier of 6.5 kcal mol^−1^) and exergonic to form the benzoate anion and C-centered radical E (−47.0 kcal mol^−1^). Dissociation of the benzoate anion leads to α-amino C-centered radical intermediate VII, which can subsequently undergo SET to form the iminium and couple with phosphine I (not calculated) to yield the product 3aa.

**Scheme 8:**
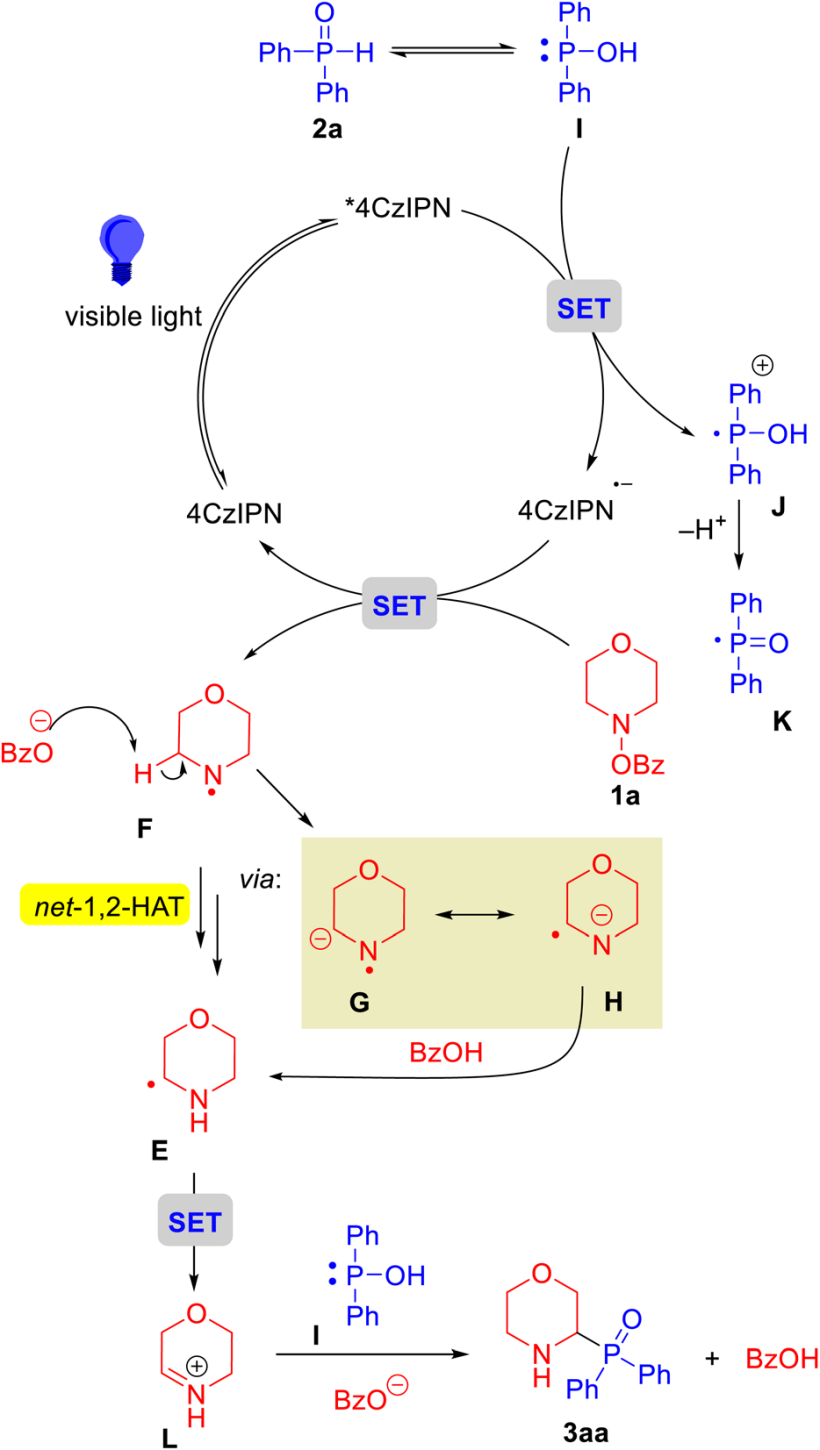
Proposed mechanism.

Based on the above experiments, DFT calculations and relevant literature,^47,68,77^ a plausible mechanism for the photocatalytic net 1,2-HAT of nitrogen radicals to access α-amino phosphine oxides is presented in Scheme 8. Initially, the photocatalyst 4CzIPN absorbs light to access the excited state 4CzIPN*. Phosphine oxide 2a is in equilibrium with Ph_2_P–OH (intermediate I), which undergoes a SET process with 4CzIPN* to generate P-centered radical cation J and 4CzIPN˙^−^. Generation of the P-centered radical is consistent with the trapping experiments in Scheme 4a and b and Stern–Volmer studies in Scheme 4c. The P-centered radical J can lose a proton to generate K. Subsequently, 4CzIPN˙^−^ transfers an electron to 1a*via* SET, causing dissociation of the N–O bond to afford N-centered radical intermediate F and benzoate anion. Next, aminyl radical F, which has acidified α-C–H bonds due to the neighboring electron deficient 7-electron nitrogen, undergoes C–H deprotonation by the benzoate anion to give the resonance stabilized radical anion G/H. Protonation at nitrogen completes the net 1,2-HAT process to form the α-amino C-centered radical intermediate E. Finally, rather than quenching an additional equivalent of excited *4CzIPN with a fresh equivalent of the phosphorus reagent, the newly formed C-centered radical E can undergo SET to generate the iminium species L and 4CzIPN˙^−^. Phosphine intermediate I can add to the α-carbon of the iminium to form a C(sp^3^)–P bond and generate α-amino phosphine oxide 3aa.

## Conclusions

The net 1,2-HAT of N-centered radicals is rare but provides a novel mechanism to functionalize carbons situated alpha to nitrogen centers. Herein, we have successfully developed a photocatalytic net 1,2-HAT of nitrogen-centered radicals (N˙) for the formation of C(sp^3^)–P bonds. Unlike past advances in the net 1,2-HAT process, this hydrogen atom transfer is unique because it is facilitated by a very weak base, specifically the benzoate counteranion released upon N–O cleavage. This observation is instructive in that it underscores the high acidity of the aminyl radical α-C–H bonds. Unsymmetrically substituted nitrogen-centered radicals show excellent selectivity in the HAT, which enables the synthesis of α-amino phosphine oxides bearing various functional groups (37 examples, up to 88% yield). A gram-scale synthesis demonstrates the scalability of the net 1,2-HAT coupling reaction. The synthesis of antitumor-active molecules highlights the promising potential applications of this reaction. Mechanistic investigations, including radical trapping experiments, Stern–Volmer fluorescence quenching experiments, cyclic voltammogram studies, cross-over experiments, KIE experiments, and DFT calculations support a net 1,2-HAT radical pathway followed by a radical polar crossover. It is also noteworthy that this net 1,2-HAT reaction involves a simple organic photoredox catalyst at room temperature, and without the addition of transition metals. The sustainability of the approach makes it an attractive choice for applications in the pharmaceutical industry.

## Author contributions

X. Y. conceived of the project. H. Z., B. L. and P. J. W. designed the experiments. A. P., Y. M., X. T., S. D. and Y. J. performed the research. M. E. R. and M. C. K. performed DFT calculations. X. Y., P. J. W. and M. C. K. wrote the manuscript.

## Conflicts of interest

There are no conflicts to declare.

## Supplementary Material

SC-OLF-D5SC00268K-s001

SC-OLF-D5SC00268K-s002

SC-OLF-D5SC00268K-s003

## Data Availability

CCDC 2293469 contains the supplementary crystallographic data for this paper.^78^ All experimental data, procedures for data analysis, and pertinent data sets are provided in the supplementary information (SI). Supplementary information is available. See DOI: https://doi.org/10.1039/d5sc00268k.
