# Supplementary material for: 1,2-Hydrogen atom transfer of aminyl radicals under photoredox catalysis for the synthesis of α-amino phosphine oxides
Source: Chem Sci. 2025 Nov 5;17(1):412–20. doi: 10.1039/d5sc00268k (PMC12617445; doi:10.1039/d5sc00268k)

Supplementary Information (SI) for Chemical Science.
This journal is © The Royal Society of Chemistry 2025

Electronic Supplementary Information (ESI)

1,2-Hydrogen Atom Transfer of Aminyl Radicals under Photoredox Catalysis for the Synthesis of α-Amino Phosphine Oxides

Ailin Pan,† Madeline E. Rotella,‡ Yamiao Meng,† Xuan Tian,† Shengzu Duan,† Yonggang Jiang,† Guogang Deng,† Bart Limburg,§ Hongbin Zhang,† Marisa C. Kozlowski,,‡ Patrick J. Walsh*,‡ & Xiaodong Yang*,†*

†Key Laboratory of Medicinal Chemistry for Natural Resource, Ministry of Education; Yunnan Key Laboratory of Research and Development for Natural Products; School of Pharmacy; Yunnan University, Southwest United Graduate School, Kunming, 650091, P. R. China. *e-mail: xdyang@ynu.edu.cn; zhanghb@ynu.edu.cn;

‡Roy and Diana Vagelos Laboratories, Penn/Merck Laboratory for High-Throughput Experimentation, Department of Chemistry, University of Pennsylvania, 231 South 34th Street, Philadelphia, Pennsylvania, 19104, USA. *e-mail: pwalsh@sas.upenn.edu; marisa@sas.upenn.edu;

§Department of Inorganic and Organic Chemistry, University of Barcelona, Carrer Martí i Franquès 1, 08028, Barcelona, Spain.

TABLE OF CONTENT

1. General Methods 2
2. Preparation of *O*-Benzoylhydroxylamines 2
3 Preparation of Phosphine Oxides 5
4. Detailed Reaction Optimizations (Table 1) 7
5. General Procedure and Characterization of the Synthesis of α-Amino Phosphine Oxides (Table 2 and 3) 10
6. Gram-scale Synthesis of **3aa** 26
7. Synthesis of an antitumor agent (**4ba**). 27
8. Mechanistic Studies 27
9. Computational Details 36
10. X-ray Crystal Structure of Compound **3ao** 49
11. Supplementary References 51
12. NMR Spectra of the Products 53

1. General Methods

All air- and moisture-sensitive solutions and chemicals were handled under a nitrogen atmosphere of a glovebox and solutions were transferred via "Titan" brand pipettor. Anhydrous solvents, including $PhCF_3$ (benzotrifluoride), PhMe (toluene), PhCl (chlorobenzene), THF (tetrahydrofuran), 1,4-dioxane, MeCN (acetonitrile), DMF (*N*,*N*-dimethylformamide)，DMSO (dimethyl sulfoxide)，DCM (dichloromethane) and DCE (1,2-dichloroethane) were purchased from Sigma-Aldrich and used without further purification. Unless otherwise stated, all reagents were commercially available and used as received without further purification. Other chemicals were obtained from Sigma-Aldrich, Acros, TCI and Alfa-Aesar. TLC was performed with Merck TLC Silica gel60 F_{254} plates with detection under UV light at 254 nm. Silica gel (200-300 mesh, Qingdao) was used for flash chromatography. Deactivated silica gel was prepared by addition of 15 mL Et_3N to 1 L of silica gel. ^{1}H and ^{13}C{^{1}H} NMR spectra were obtained using a Brüker DRX 400 spectrometer at 400 MHz and 100 MHz, respectively. Chemical shifts were reported in units of parts per million (ppm) downfield from tetramethylsilane (TMS), and all coupling constants were reported in hertz. X-Ray single crystal diffraction data were recorded on Brüker D8 VENTURE. High resolution mass spectra were taken on an AB QSTAR Pulsar mass spectrometer. Melting points were obtained on an XT-4 melting-point apparatus and were uncorrected.

2. Preparation of *O*-Benzoylhydroxylamines

General Procedure A: ***Preparation of O-benzoylhydroxylamines 1a – d-1i.***

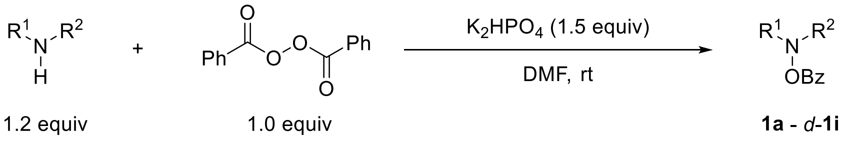

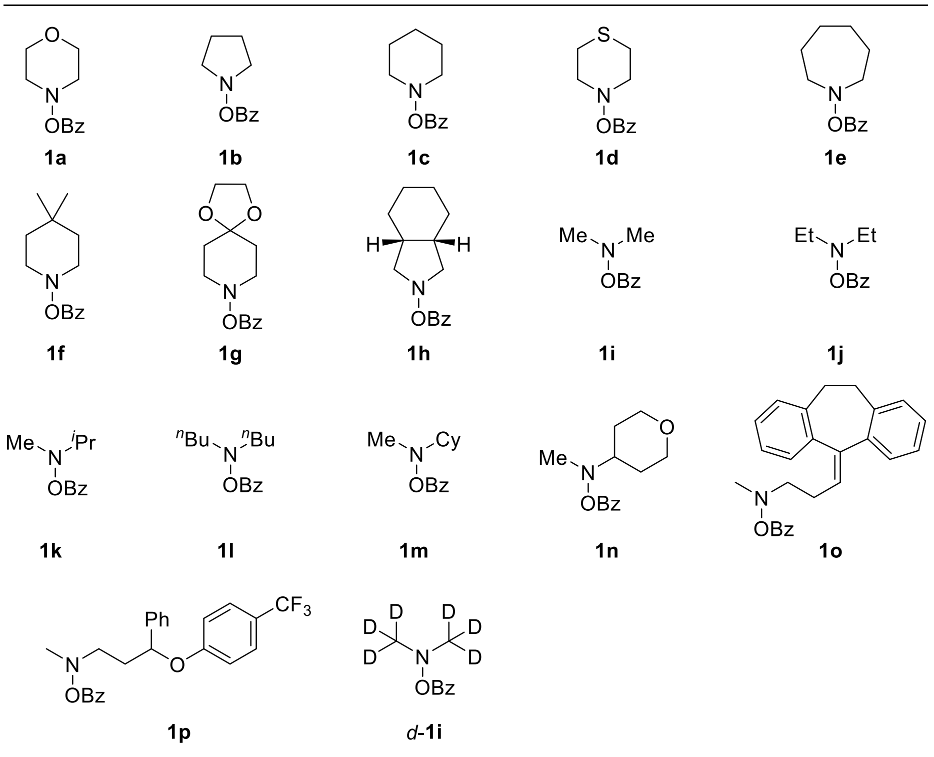

The reaction was performed following the literature procedures[1-7]

A 100 mL, one-necked, round-bottomed flask equipped with a Teflon-coated magnetic stir bar was charged with benzoyl peroxide (2.42 g, 10.0 mmol, 1.0 equiv.), dipotassium hydrogen phosphate (2.61 g, 15.0 mmol, 1.5 equiv.), and DMF (25 mL). The suspension was stirred and the amine (12.0 mmol, 1.2 equiv.) was added via syringe in one portion. The suspension was stirred at ambient temperature for the indicated reaction time. Deionized water (40 mL) was added, and the contents were stirred vigorously for several minutes until all solids dissolved. The reaction mixture was transferred to a 250 ml separatory funnel and extracted with 80 mL of ethyl acetate. The organic phase was collected and washed with two 50 mL portions of saturated $NaHCO_3$ solution. All of the aqueous fractions were combined and extracted with three 50 mL portions of ethyl acetate, dried over $MgSO_4$, and concentrated by rotary evaporation. The resulting crude product mixture was purified by flash column chromatography, eluting with the indicated solvent system (typically using PE/EtOAc = 15:1 to 1:1 as elution), to afford the desired product. The product was stored at sub-ambient temperature under anhydrous conditions. The ^{1}H and ^{13}C{^{1}H} data for these compounds match the literature data.[1-7]

(3a*R*,7a*S*)-Octahydro-2*H*-isoindol-2-yl benzoate (1h)

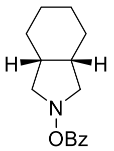

The reaction was performed following **General Procedure A**: Purification by flash chromatography over silica gel (hexanes:ethyl acetate = 10:1) led to the desired product **1h** (1.30 g, 53% yield); Yellow oil; **R*f*** = 0.41 (hexanes:ethyl acetate = 7:1); **^{1}H NMR** (400 MHz, Chloroform-*d*) δ 7.95 (d, *J* = 7.6 Hz, 2H), 7.51 (t, *J* = 7.2 Hz, 1H), 7.39 (t, *J* = 8.0 Hz, 2H), 3.48 – 3.44 (m, 2H), 3.25 – 3.20 (m, 2H), 2.30 – 2.25 (m, 2H), 1.65 – 1.33 (m, 8H) ppm; **^{13}C NMR** (100 MHz, Chloroform-*d*) δ 165.3, 132.9, 129.5, 129.3, 128.4, 62.3, 36.2, 26.1, 22.6 ppm; **HRMS** calc'd for $C_{15}H_{20}NO_2^+$ 246.1489, found 246.1493 $[M+H]^+$.

***O*-Benzoyl-*N*-methyl-*N*-(tetrahydro-2*H*-pyran-4-yl)hydroxylamine (1n)**

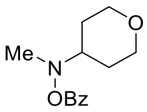

The reaction was performed following **General Procedure A**: Purification by flash chromatography over silica gel (hexanes:ethyl acetate = 2:1) led to the desired product **1n** (1.67 g, 71% yield); Colorless oil; **R*f*** = 0.43 (hexanes:ethyl acetate = 1:1); **^{1}H NMR** (400 MHz, Chloroform-*d*) δ 7.92 (d, *J* = 7.2 Hz, 2H), 7.47 (t, *J* = 7.6 Hz, 1H), 7.34 (t, *J* = 8.0 Hz, 2H), 3.96 – 3.91 (m, 2H), 3.35 – 3.28 (m, 2H), 2.97 – 2.90 (m, 1H), 2.80 (s, 3H), 1.80 – 1.75 (m, 2H), 1.70 – 1.60 (m, 2H) ppm; **^{13}C NMR** (100 MHz, Chloroform-*d*) δ 164.9, 133.0, 129.3, 128.9, 128.3, 66.4, 64.0, 42.8, 29.3 ppm; **HRMS** calc'd for $C_{13}H_{18}NO_3^+$ 236.1281, found 236.1283 $[M+H]^+$.

***O*-benzoyl-*N*,*N*-bis(methyl-*d*$_3$)hydroxylamine**

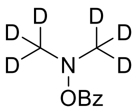

The reaction was performed following **General Procedure A**: Purification by flash chromatography over silica gel (hexanes:ethyl acetate = 4:1) led to the desired product *d*-**1i** (1.12 g, 65% yield); Colorless oil;

R*f* = 0.43 (hexanes:ethyl acetate = 3:1); **¹H NMR** (400 MHz, Chloroform-*d*) δ 8.00 (d, *J* = 7.2 Hz, 2H), 7.55 (t, *J* = 7.6 Hz, 1H), 7.42 (t, *J* = 7.6 Hz, 2H) ppm; **¹³C NMR** (100 MHz, Chloroform-*d*) δ 165.1, 133.1, 129.5, 129.4, 128.5, 48.5 – 47.2 (m) ppm; **HRMS** calc'd for $C_9H_6D_6NO_2^+$ 172.1239, found 172.1239 $[M+H]^+$.

General Procedure B: ***Preparation of O-benzoylhydroxylamines 1q and 1r.***

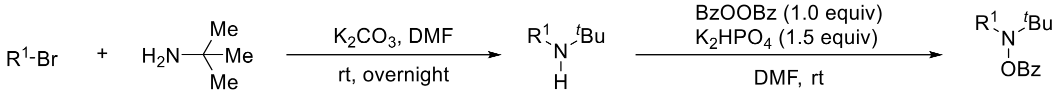

1q, 1r

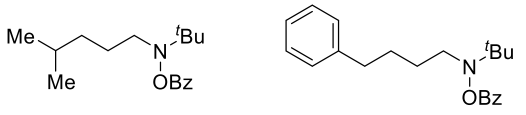

1q **1r**

Step 1: A flame-dried 50 mL round-bottom flask, equipped with a stir bar, was charged with *tert*-butylamine (2.5 mL, 24.0 mmol, 1.0 equiv.) and K_2CO_3 (4.97 g, 36.0 mmol, 1.5 equiv.) in DMF (20 mL). At room temperature was added alkyl bromides (28.8 mmol, 1.2 equiv.). The mixture was allowed to stir at room temperature for overnight. Filter the resulting reaction solution and add three 5 ml batches of DMF wash to the filter funnel, mixing all solutions together.

Step 2: A 100 mL, one-necked, round-bottomed flask equipped with a Teflon-coated magnetic stirbar was charged with all solutions (Step 1), dipotassium hydrogen phosphate (5.22 g, 30 mmol, 1.5 equiv.). The suspension was stirred and benzoyl peroxide (4.84 g, 20.0 mmol, 1.0 equiv.) was added via syringe in one portion. The suspension was stirred at ambient temperature for the indicated reaction time. Deionized water (40 mL) was added and the contents were stirred vigorously for several minutes until all solids dissolved. The reaction mixture was transferred to a 250 ml separatory funnel and extracted with 80 mL of ethyl acetate. The organic phase was collected and washed with two 50 mL portions of saturated $NaHCO_3$ solution. All of the aqueous fractions were combined and extracted with three 50 mL portions of ethyl acetate, dried over $MgSO_4$, and concentrated by rotary evaporation. The resulting crude product mixture was purified by flash column chromatography, eluting with the indicated solvent system (typically using PE/EtOAc = 15:1 to 10:1 as elution), to afford desired product. The product was stored at sub-ambient temperature under anhydrous conditions.

***O*-Benzoyl-*N*-(*tert*-butyl)-*N*-(4-methylpentyl)hydroxylamine (1q)**

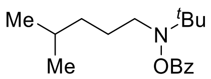

The reaction was performed following **General Procedure B**: Purification by flash chromatography over silica gel (hexanes:ethyl acetate = 20:1); led to the desired product **1q** (2.83 g, 51% yield); Colorless oil; **R*f*** = 0.25 (hexanes:ethyl acetate = 20:1); **¹H NMR** (400 MHz, Chloroform-*d*) δ 8.04 (d, *J* = 8.4 Hz, 2H), 7.56 (t, *J* = 6.8 Hz, 1H), 7.44 (t, *J* = 8.0 Hz, 2H), 2.89 (t, *J* = 7.6 Hz, 2H), 1.54 – 1.45 (m, 3H), 1.20 (s, 9H), 1.23 – 1.17 (m, 2H), 0.82 (d, *J* = 6.4 Hz, 6H) ppm; **¹³C NMR** (100 MHz, Chloroform-*d*) δ 166.2, 133.0, 129.7, 129.6, 128.5, 60.4, 52.2, 36.7, 27.9, 25.9, 25.7, 22.7 ppm; **HRMS** calc'd for $C_{17}H_{28}NO_2^+$

278.2115, found 278.2111 [M+H]$^+$.

***O*-Benzoyl-*N*-(*tert*-butyl)-*N*-(4-phenylbutyl)hydroxylamine (1r)**

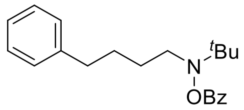

The reaction was performed following **General Procedure B**: Purification by flash chromatography over silica gel (hexanes:ethyl acetate = 20:1); led to the desired product **1r** (2.41 g, 37% yield); Colorless oil; **R*f*** = 0.23 (hexanes:ethyl acetate = 20:1); **^{1}H NMR** (400 MHz, Chloroform-*d*) δ 8.15 (d, *J* = 7.6 Hz, 2H), 7.69 (t, *J* = 6.8 Hz, 1H), 7.57 (t, *J* = 7.6 Hz, 2H), 7.35 – 7.24 (m, 5H), 3.06 (t, *J* = 7.2 Hz, 2H), 2.70 (t, *J* = 7.6 Hz, 2H), 1.85 – 1.68 (m, 4H), 1.33 (s, 9H) ppm. **^{13}C NMR** (100 MHz, Chloroform-*d*) δ 166.2, 142.6, 133.0, 129.7, 129.5, 128.6, 128.5, 128.3, 125.7, 60.4, 51.7, 35.9, 29.3, 27.7, 25.7 ppm; **HRMS** calc'd for $C_{21}H_{28}NO_2^+$ 326.2115, found 326.2110 [M+H]$^+$.

3 Preparation of Phosphine Oxides

(**2a**) was purchased from Sigma-Aldrich and directly used.

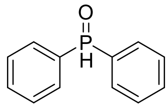

2a

General Procedure C: ***Preparation of phosphine oxides 2b, 2d, 2e, 2g, 2h, 2j, 2p.***

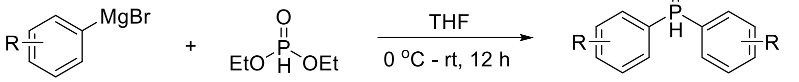

2b, 2d, 2e, 2g, 2h, 2j, 2p

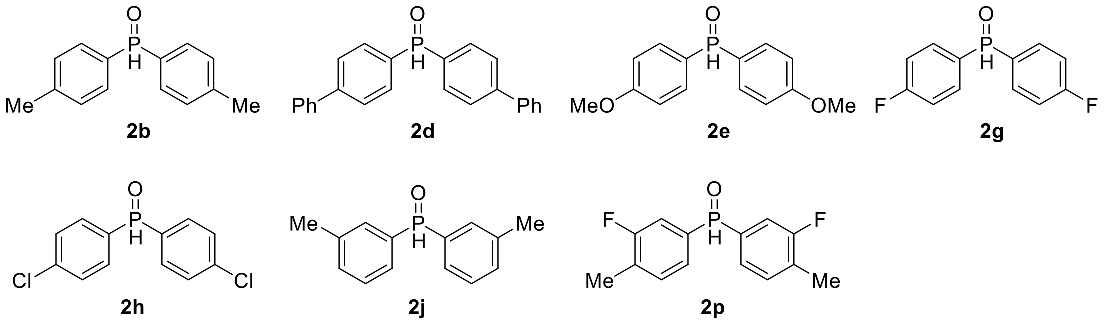

The reaction was performed following the literature procedures[8-11]

A flame-dried 250 mL flask which equipped with a stir bar was purged with N_2 (3 times), charged with aryl magnesium bromide/THF (30.0 mmol, 3.0 equiv.) by syringe, and the solution cooled to 0 °C in an ice bath. A solution of diethylphosphite (1.3 mL, 10 mmol, 1.0 equiv.) in anhydrous THF (5 mL) was then added dropwise over 10 min. The reaction mixture was allowed to stir at room temperature for 12 h. After completion of the reaction, the reaction vial was opened and the mixture was cooled to 0 °C in an ice bath, then quenched by slowly adding 100 ml 0.1 N HCl. The reaction mixture was washed by CH_2Cl_2 (100 mL X 5). The organic phases were separated, collected, dried over anhydrous Na_2SO_4 and

filtered. Silica gel was added, and the mixture was concentrated under reduced pressure. Purification of the residue by flash chromatography on silica gel using ethyl acetate as eluant afforded the desired phosphine oxides.

General Procedure D: ***Preparation of phosphine oxides 2c, 2f, 2i, 2k-2o, 2q-2t.***

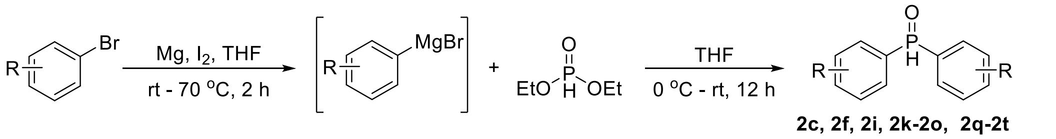

2c, 2f, 2i, 2k-2o, 2q-2t

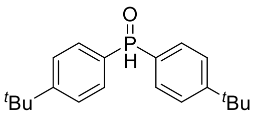

2c

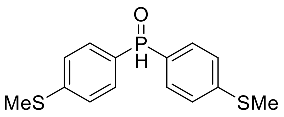

2f

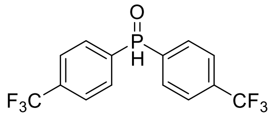

2i

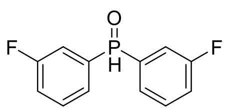

2k

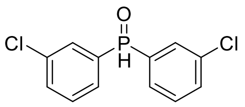

2l

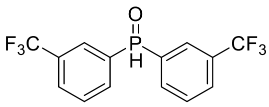

2m

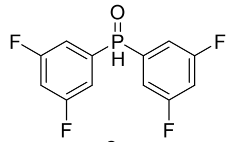

2n

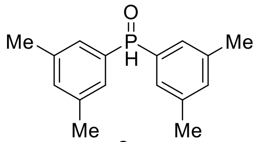

2o

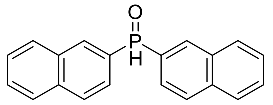

2q

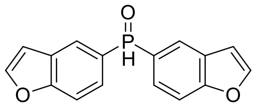

2r

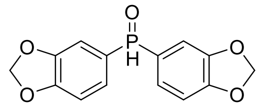

2s

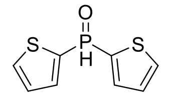

2t

The reaction was performed following the literature procedures[8-11]

Aryl bromide (30.0 mol, 3.0 equiv.) in THF (30 mL) was added slowly to a stirred THF (50 mL) solution of I_2 (25.4 mg, 0.1 mmol, 1 mol%) containing magnesium turnings (729.2 mg, 30.0 mol, 3.0 equiv.), and heated to reflux under nitrogen for 2 h. Next, the solution was cooled to 0 °C in an ice bath and a solution of diethylphosphite (1.3 mL, 10 mmol, 1.0 equiv.) in anhydrous THF (5 mL) was then added dropwise over 10 min. The reaction mixture was allowed to stir at room temperature for 12 h. After completion of the reaction, the reaction vial was opened and the mixture was cooled to 0 °C in an ice bath, then quenched by slowly adding 100 ml 0.1 N HCl. The reaction mixture was washed with CH_2Cl_2 (100 mL X 5). The organic phases were collected, dried over anhydrous Na_2SO_4 and filtered. Silica gel was added, and the organic mixture was concentrated under reduced pressure. Purification of the residue by flash chromatography on silica gel using ethyl acetate as eluant afforded the desired esters.

General Procedure E: ***Preparation of phosphine oxide 2u.***

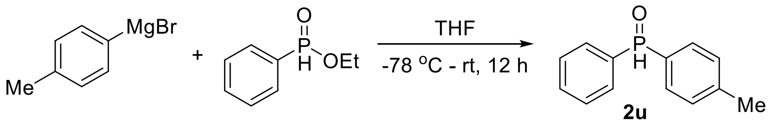

2u

The reaction was performed following the literature procedures[8-11]

A flame-dried 250 mL flask which equipped with a stir bar was purged with N_2 (3 times), charged with *p*-tolylmagnesium bromide/THF (22.0 mmol, 2.2 equiv.) by syringe, and the solution cooled to -78 °C. A solution of ethyl phenylphosphinate (1.5 mL, 10 mmol, 1.0 equiv.) in anhydrous THF (5 mL) was then

added dropwise over 10 min. The reaction mixture was allowed to stir at room temperature for 12 h. After completion of the reaction, the reaction vial was opened and the mixture was cooled to 0 °C in an ice bath, then quenched by slowly adding 100 ml 0.1 N HCl. The reaction mixture was washed with CH_2Cl_2 (100 mL X 5). The organic phases were collected, dried over anhydrous Na_2SO_4 and filtered. To the filtrate, silica gel was added, and the mixture was concentrated under reduced pressure. Purification of the residue by flash chromatography on silica gel using ethyl acetate as eluant afforded the desired esters.

General Procedure F: ***Preparation of phosphine oxide 2v.***

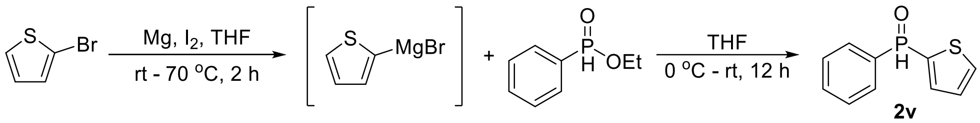

The reaction was performed following the literature procedures[8-11]

2-Bromothiophene (22.0 mol, 2.2 equiv.) in THF (30 mL) was added slowly to a stirred THF (50 mL) solution of I_2 (25.4 mg, 0.1 mmol, 1 mol%) containing magnesium turnings (534.8 mg, 22.0 mol, 2.2 equiv.), and heated to reflux under nitrogen for 2 hours. Next, the solution was cooled to 0 °C in an ice bath and a solution of ethyl phenylphosphinate (1.3 mL, 10 mmol, 1.0 equiv.) in anhydrous THF (5 mL) was then added dropwise over 10 min. The reaction mixture was allowed to stir at room temperature for 12 h. After completion of the reaction, the reaction vial was opened and the mixture was cooled to 0 °C in an ice bath, then quenched by slowly adding 100 ml 0.1 N HCl. The reaction mixture was washed with CH_2Cl_2 (100 mL X 5). The organic phases were combined, dried over anhydrous Na_2SO_4 and filtered. Silica gel was added, and the organic mixture was concentrated under reduced pressure. Purification of the residue by flash chromatography on silica gel using ethyl acetate as eluant afforded the desired esters.

4. Detailed Reaction Optimizations (Table 1)

Table S1. Screening of photocatalyst[a]

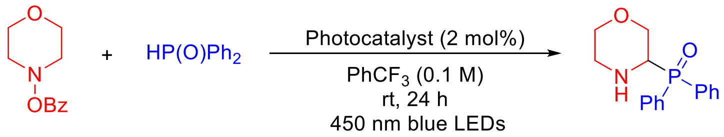

1a **2a** **3aa**

entry	photocatalyst	CAS number	yield of **3aa** (%)[b]
1	Ir(ppy)$_3$	94928-86-6	0
2	Ir(*p*-^{t}Bu-ppy)$_3$	359014-76-9	72
3	[Ir(ppy)$_2$(bpy)]PF$_6$	106294-60-4	trace
4	Ir[dF(CF$_3$)ppy]$_2$(dtbpy)[PF$_6$]	870987-63-6	48
5	[Ir(dtbbpy)(ppy)$_2$]PF$_6$	676525-77-2	69
6	Ru(bpy)$_3$[PF$_6$]$_2$	60804-74-2	47
7	Perylene	198-55-0	30
8	4CzIPN	1416881-52-1	79

[a]Reactions conducted on a 0.1 mmol scale using 2.0 equiv. of **1a**, and 1.0 equiv. of **2a**, photocatalyst (2 mol%) in 1 mL of $PhCF_3$ at room temperature under blue LEDs (450 nm) for 24 h. [b]Assay yields (AY)

were determined by ^{1}H NMR spectroscopy with CH_2Br_2 as the internal standard.

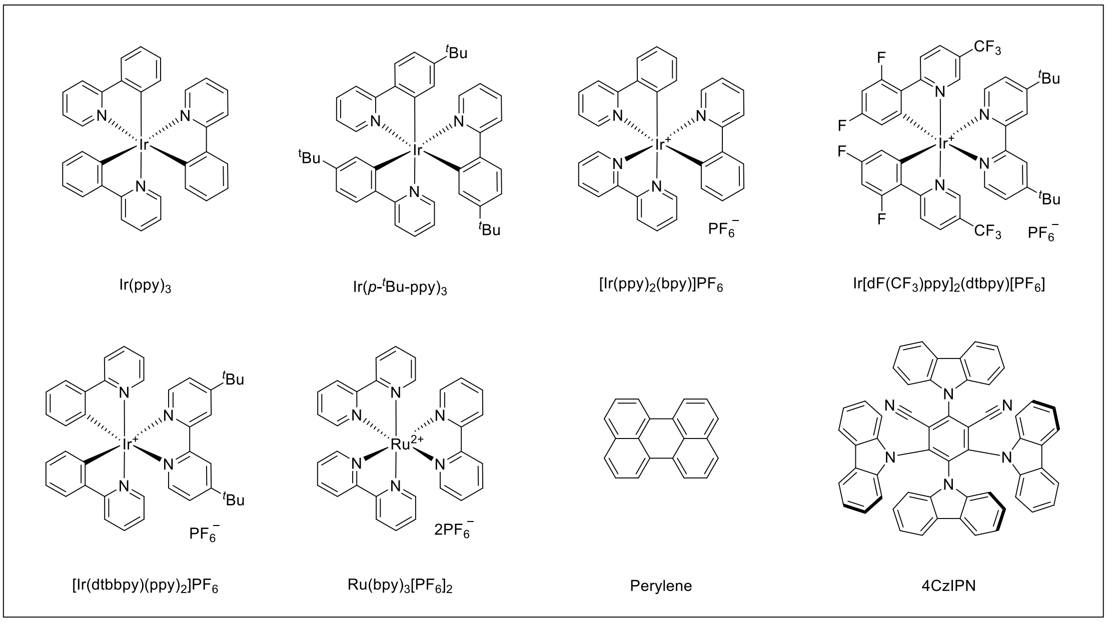

Table S2. Screening of solvent[a]

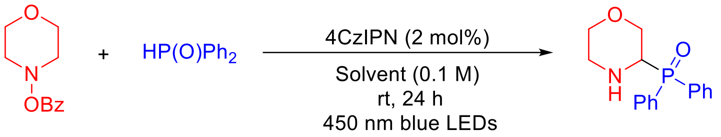

1a **2a** **3aa**

entry	Solvent	yield of **3aa** (%)[b]
1	$PhCF_3$	79
2	PhCl	8
3	Toluene	trace
4	THF	0
5	1,4-dioxane	0
6	DCM	35
7	DCE	64
8	MeCN	50
9	DMF	0
10	DMSO	0
11	MeCN : $PhCF_3$ = 1 : 1	83
12	MeCN : $PhCF_3$ = 2 : 1	82
13	MeCN : $PhCF_3$ = 1 : 2	83

[a]Reactions conducted on a 0.1 mmol scale using 2.0 equiv. of **1a**, and 1.0 equiv. of **2a**, 4CzIPN (2 mol%) in 1 mL of solvent at room temperature under blue LEDs (450 nm) for 24 h. [b]Assay yields (AY) were determined by ^{1}H NMR spectroscopy with CH_2Br_2 as the internal standard.

Table S3. Screening of photocatalyst equivalents[a]

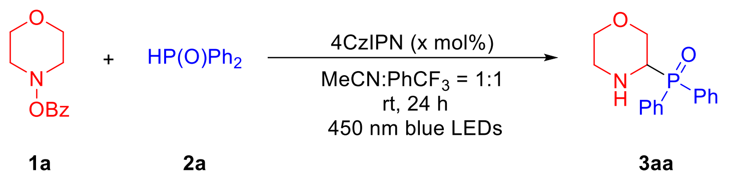

entry	x (mol%)	yield of **3aa** (%)[b]
1	1	51
2	2	83
3	3	85
4	4	82
5	0	0

[a]Reactions conducted on a 0.1 mmol scale using 2.0 equiv. of **1a**, and 1.0 equiv. of **2a**, 4CzIPN (x mol%) in 1 mL of MeCN : $PhCF_3$ = 1 : 1 at room temperature under blue LEDs (450 nm) for 24 h. [b]Assay yields (AY) were determined by ^1H NMR spectroscopy with CH_2Br_2 as the internal standard.

Table S4. Screening of ratio of 1a/2a[a]

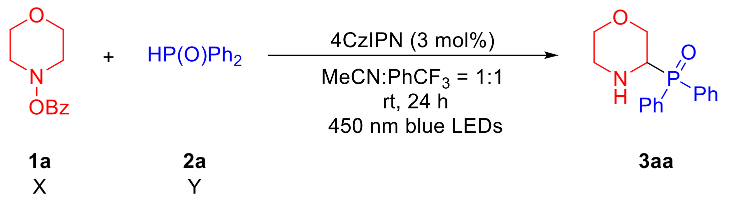

entry	**1a**/**2a** (mol%)	yield of **3aa** (%)[b]
1	3.0/1	72
2	2.5/1	70
3	2.0/1	85
4	1.5/1	82
5	1.2/1	56

[a]Reactions conducted on a 0.1 mmol scale using X equiv. of **1a**, and Y equiv. of **2a**, 4CzIPN (3 mol%) in 1 mL of MeCN : $PhCF_3$ = 1 : 1 at room temperature under blue LEDs (450 nm) for 24 h. [b]Assay yields (AY) were determined by ^1H NMR spectroscopy with CH_2Br_2 as the internal standard.

Table S5. Screening of visible light wavelength[a]

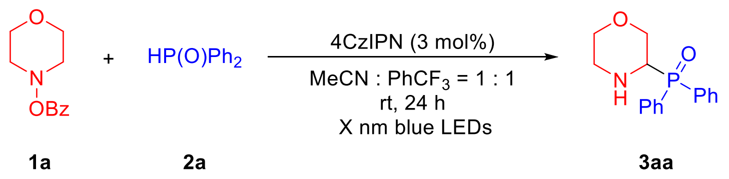

entry	visible light wavelength (nm)	yield of **3aa** (%)[b]
1	450	85
2	430	75
3	none	0

[a]Reactions conducted on a 0.1 mmol scale using 2.0 equiv. of **1a**, and 1.0 equiv. of **2a**, 4CzIPN (3 mol%) in 1 mL of MeCN : $PhCF_3$ = 1 : 1 at room temperature under blue LEDs (X nm) for 24 h. [b]Assay yields

(AY) were determined by ^{1}H NMR spectroscopy with CH_2Br_2 as the internal standard.

5. General Procedure and Characterization of the Synthesis of α-Amino Phosphine Oxides (Table 2 and 3)

General Procedure G:

An oven-dried 10 mL reaction vial equipped with a stir bar in a glove box under a nitrogen atmosphere at room temperature was charged with 4CzIPN (9.5 mg, 0.012 mmol, 3 mol%), *O*-benzoyl hydroxylamines (**1**, 0.8 mmol, 2.0 equiv.) and phosphine oxides (**2**, 0.4 mmol, 1.0 equiv.). Next, MeCN : $PhCF_3$ = 1 : 1 (4.0 mL) was sequentially added via syringe and the mixture was stirred and until all the solids dissolved. The vial was capped, removed from the glove box, and stirred for 24 h with blue LEDs irradiation (see photo below of apparatus, Figure S1). The temperature was maintained at room temperature via cooling with a fan (Figure S1). After the reaction period, the lights were turned off, the cap was removed, reaction mixture was transferred to a round bottom flask and the solution was concentrated in vacuo. The resulting mixed product was dissolved by adding 15 mL of ethyl acetate and washed with $NaHCO_3$ (10 mL X 2). The aqueous phase was collected and then extracted with ethyl acetate (10 mL X 2), all organic phases were collected and dried with Na_2SO_4, and the combined organic solutions were concentrated in *vacuo*. The crude material was loaded onto a silica gel column and purified by flash chromatography.

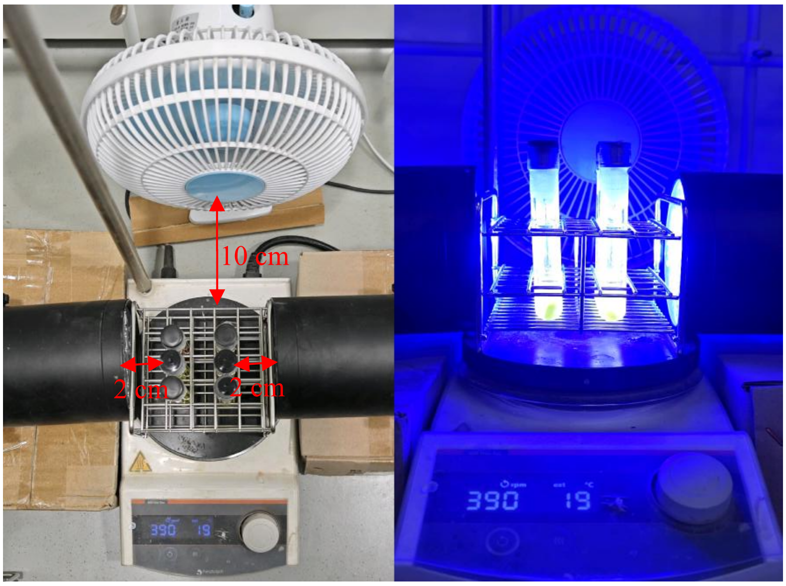

Figure S1. Experimental setup

Note: The reactions were performed on a wire frame (12.5 X 12.5 cm) with a fan (5 W, 24 X 24 cm. The fan was at a distance of about 10.0 cm from the frame) to offset the heat generated from two 18 W 450nm light emitting diode (LED) lamps (the distance was about 2.0 cm from vials to LEDs.). Irradiation was performed at room temperature.

Characterization of Products:

Morpholin-3-yldiphenylphosphine oxide (3aa):

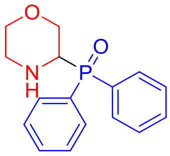

The reaction was performed following the **General Procedure G** with morpholino benzoate **1a** (165.7 mg, 0.8 mmol) and diphenylphosphine oxide **2a** (80.8 mg, 0.4 mmol). The crude product was separated by flash chromatography on deactivated silica gel (hexane:ethyl acetate = 1:1 to ethyl acetate:methanol = 10:1) to give the product **3aa** (93.0 mg, 81% yield) as a light yellow oil. **R*f*** = 0.34 (ethyl acetate:methanol = 10:1). **^{1}H NMR** (400 MHz, Chloroform-*d*) δ 7.92 – 7.86 (m, 2H), 7.79 – 7.73 (m, 2H), 7.55 – 7.42 (m, 6H), 3.90 – 3.85 (m, 1H), 3.80 – 3.72 (m, 2H), 3.60 (td, *J* = 10.8, 3.2 Hz, 1H), 3.45 (td, *J* = 11.2, 3.6 Hz, 1H), 2.97 – 2.85 (m, 2H), 2.00 (s, 1H) ppm; **^{13}C{^{1}H} NMR** (100 MHz, Chloroform-*d*) δ 132.4, 132.3, 131.7 (d, *J* = 8.9 Hz), 131.3 (d, *J* = 9.0 Hz), 130.6 (d, *J* = 43.4 Hz), 129.6 (d, *J* = 44.2 Hz), 128.9 (d, *J* = 8.6 Hz), 128.7 (d, *J* = 8.5 Hz), 67.6, 66.8 (d, *J* = 4.8 Hz), 55.2 (d, *J* = 79.5 Hz), 46.3 (d, *J* = 10.7 Hz) ppm; **^{31}P NMR** (162 MHz, Chloroform-*d*) δ 27.85 ppm; **HRMS** calc'd for $C_{16}H_{19}NO_2P^+$ 288.1148, found 288.1144 $[M+H]^+$.

Morpholin-3-yldi-*p*-tolylphosphine oxide (3ab):

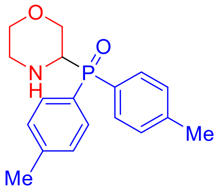

The reaction was performed following the **General Procedure G** with morpholino benzoate **1a** (165.7 mg, 0.8 mmol) and di-*p*-tolylphosphine oxide **2b** (92.0 mg, 0.4 mmol). The crude product was separated by flash chromatography on deactivated silica gel (hexane:ethyl acetate = 1:1 to ethyl acetate:methanol = 10:1) to give the product **3ab** (70.6 mg, 56% yield) as a colorless oil. **R*f*** = 0.36 (ethyl acetate:methanol = 10:1). **^{1}H NMR** (400 MHz, Chloroform-*d*) δ 7.79 – 7.74 (m, 2H), 7.66 – 7.62 (m, 2H), 7.30 – 7.25 (m, 4H), 3.90 – 3.86 (m, 1H), 3.77 – 3.70 (m, 2H), 3.60 (td, *J* = 10.8, 2.8 Hz, 1H), 3.47 (td, *J* = 10.4, 3.6 Hz, 1H), 2.97 – 2.86 (m, 2H), 2.38 (s, 3H), 2.37 (s, 3H), 2.16 (s, 1H) ppm; **^{13}C{^{1}H} NMR** (100 MHz, Chloroform-*d*) δ 142.89 (d, *J* = 1.4 Hz), 142.86 (d, *J* = 1.8 Hz), 131.7 (d, *J* = 9.3 Hz), 131.3 (d, *J* = 9.5 Hz), 129.6 (d, *J* = 6.8 Hz), 129.5 (d, *J* = 6.7 Hz), 127.42 (d, *J* = 41.4 Hz), 126.41 (d, *J* = 40.4 Hz), 67.6, 67.0 (d, *J* = 4.7 Hz), 55.3 (d, *J* = 79.7 Hz), 46.4 (d, *J* = 10.8 Hz), 21.68, 21.66 ppm; **^{31}P NMR** (162 MHz, Chloroform-*d*) δ 28.38 ppm; **HRMS** calc'd for $C_{18}H_{23}NO_2P^+$ 316.1461, found 316.1467 $[M+H]^+$.

Bis(4-(*tert*-butyl)phenyl)(morpholin-3-yl)phosphine oxide (3ac):

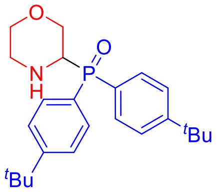

The reaction was performed following the **General Procedure G** with morpholino benzoate **1a** (165.7 mg, 0.8 mmol) and bis(4-(*tert*-butyl)phenyl)phosphine oxide **2c** (125.7 mg, 0.4 mmol). The crude product was separated by flash chromatography on deactivated silica gel (hexane:ethyl acetate = 2:1 to ethyl acetate:methanol = 20:1) to give the product **3ac** (81.4 mg, 51% yield) as a colorless oil. **R*f*** = 0.42 (ethyl acetate:methanol = 20:1). **^{1}H NMR** (400 MHz, Chloroform-*d*) δ 7.84 – 7.79 (m, 2H), 7.72 – 7.67 (m, 2H), 7.51 – 7.46 (m, 4H), 3.90 – 3.86 (m, 1H), 3.78 – 3.72 (m, 2H), 3.63 (td, *J* = 10.8, 2.8 Hz, 1H), 3.49 (td, *J* = 10.8, 4.0 Hz, 1H), 2.99 – 2.88 (m, 2H), 2.13 (s, 1H), 1.31 (s, 9H), 1.30 (s, 9H) ppm; **^{13}C{^{1}H} NMR** (100 MHz, Chloroform-*d*) δ 155.8 (d, *J* = 2.7 Hz), 155.7 (d, *J* = 2.7 Hz), 131.6 (d, *J* = 9.3 Hz),

131.3 (d, J = 9.5 Hz), 127.5 (d, J = 36.6 Hz), 126.5 (d, J = 37.7 Hz), 125.9 (d, J = 4.4 Hz), 125.8 (d, J = 4.5 Hz), 67.7, 67.1 (d, J = 4.8 Hz), 55.4 (d, J = 79.5 Hz), 46.5 (d, J = 10.9 Hz), 35.1, 31.19, 31.17 ppm, one resonance was not observed due to overlapping peaks; **^{31}P NMR** (162 MHz, Chloroform-d) δ 27.78 ppm; **HRMS** calc'd for $C_{24}H_{35}NO_2P^+$ 400.2400, found 400.2398 $[M+H]^+$.

Di([1,1'-biphenyl]-4-yl)(morpholin-3-yl)phosphine oxide (3ad):

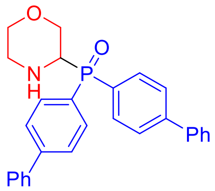

The reaction was performed following the **General Procedure G** with morpholino benzoate **1a** (165.7 mg, 0.8 mmol) and di([1,1'-biphenyl]-4-yl)phosphine oxide **2d** (141.6 mg, 0.4 mmol). The crude product was separated by flash chromatography on deactivated silica gel (hexane:ethyl acetate = 2:1 to ethyl acetate:methanol = 20:1) to give the product **3ad** (137.0 mg, 78% yield) as a light yellow oil. **R$_f$** = 0.40 (ethyl acetate:methanol = 20:1). **^{1}H NMR** (400 MHz, Chloroform-d) δ 8.05 – 8.00 (m, 2H), 7.92 – 7.87 (m, 2H), 7.76 – 7.71 (m, 4H), 7.63 – 7.58 (m, 4H), 7.49 – 7.45 (m, 4H), 7.42 – 7.37 (m, 2H), 4.01 – 3.97 (m, 1H), 3.90 – 3.79 (m, 2H), 3.71 (td, J = 11.2, 3.2 Hz, 1H), 3.54 (td, J = 11.2, 3.6 Hz, 1H), 3.05 – 2.94 (m, 2H), 2.06 (s, 1H) ppm; **^{13}C{^{1}H} NMR** (100 MHz, Chloroform-d) δ 145.3 (d, J = 2.4 Hz), 145.2 (d, J = 2.5 Hz), 139.9, 139.8, 132.3 (d, J = 9.3 Hz), 131.9 (d, J = 9.5 Hz), 129.3 (d, J = 37.1 Hz), 129.1, 128.4, 128.3, 128.3 (d, J = 38.2 Hz), 127.7 (d, J = 7.3 Hz), 127.5 (d, J = 7.3 Hz), 127.4, 67.7, 67.0 (d, J = 4.8 Hz), 55.5 (d, J = 79.7 Hz), 46.5 (d, J = 10.8 Hz) ppm, two resonance was not observed due to overlapping peaks; **^{31}P NMR** (162 MHz, Chloroform-d) δ 27.93 ppm; **HRMS** calc'd for $C_{28}H_{27}NO_2P^+$ 440.1774, found 440.1774 $[M+H]^+$.

Bis(4-methoxyphenyl)(morpholin-3-yl)phosphine oxide (3ae):

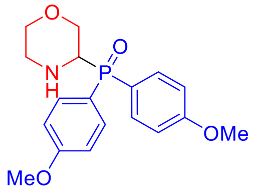

The reaction was performed following the **General Procedure G** with morpholino benzoate **1a** (165.7 mg, 0.8 mmol) and bis(4-methoxyphenyl)phosphine oxide **2e** (104.8 mg, 0.4 mmol). The crude product was separated by flash chromatography on deactivated silica gel (hexane:ethyl acetate = 1:1 to ethyl acetate:methanol = 5:1) to give the product **3ae** (62.5 mg, 45% yield) as a colorless oil. **R$_f$** = 0.32 (ethyl acetate:methanol = 5:1). **^{1}H NMR** (400 MHz, Chloroform-d) δ 7.84 – 7.79 (m, 2H), 7.70 – 7.66 (m, 2H), 7.00 – 6.95 (m, 4H), 3.91 – 3.86 (m, 1H), 3.84 (s, 3H), 3.83 (s, 3H), 3.76 (d, J = 10.4 Hz, 1H), 3.71 – 3.68 (m, 1H), 3.58 (td, J = 10.8, 2.8 Hz, 1H), 3.48 (td, J = 10.8, 3.6 Hz, 1H), 2.98 – 2.87 (m, 2H), 2.08 (s, 1H) ppm; **^{13}C{^{1}H} NMR** (100 MHz, Chloroform-d) δ 162.80 (d, J = 2.4 Hz), 162.77 (d, J = 2.4 Hz), 133.7 (d, J = 10.3 Hz), 133.3 (d, J = 10.4 Hz), 122.0 (d, J = 46.5 Hz), 120.9 (d, J = 47.7 Hz), 114.5 (d, J = 8.8 Hz), 114.4 (d, J = 8.8 Hz), 67.6, 67.1 (d, J = 4.8 Hz), 55.6 (d, J = 80.4 Hz), 55.36, 55.35, 46.5 (d, J = 10.8 Hz) ppm; **^{31}P NMR** (162 MHz, Chloroform-d) δ 28.05 ppm; **HRMS** calc'd for $C_{18}H_{23}NO_4P^+$ 348.1359, found 348.1361 $[M+H]^+$.

Bis(4-(methylthio)phenyl)(morpholin-3-yl)phosphine oxide (3af):

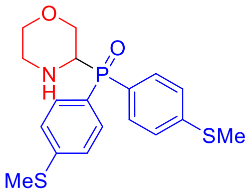

The reaction was performed following the **General Procedure G** with morpholino benzoate **1a** (165.7 mg, 0.8 mmol) and bis(4-(methylthio)phenyl)phosphine oxide **2f** (117.6 mg, 0.4 mmol). The crude product was separated by flash chromatography on deactivated silica gel (hexane:ethyl acetate = 1:1 to ethyl acetate:methanol = 10:1) to give the product **3af** (80.4 mg, 53% yield) as a light yellow oil. $\mathbf{R}_f$ = 0.37 (ethyl acetate:methanol = 10:1). **^{1}H NMR** (400 MHz, Chloroform-*d*) δ 7.80 – 7.74 (m, 2H), 7.66 – 7.61 (m, 2H), 7.32 – 7.27 (m, 4H), 3.92 – 3.88 (m, 1H), 3.79 – 3.69 (m, 2H), 3.58 (td, *J* = 11.2, 3.2 Hz, 1H), 3.47 (td, *J* = 10.4, 3.6 Hz, 1H), 2.97 – 2.87 (m, 2H), 2.49 (s, 3H), 2.48 (s, 3H), 2.00 (s, 1H) ppm; **^{13}C{^{1}H} NMR** (100 MHz, Chloroform-*d*) δ 145.1 (d, *J* = 2.9 Hz), 145.0 (d, *J* = 3.0 Hz), 132.0 (d, *J* = 9.6 Hz), 131.6 (d, *J* = 9.8 Hz), 126.0 (d, *J* = 46.4 Hz), 125.6 (d, *J* = 8.6 Hz), 125.4 (d, *J* = 8.6 Hz), 125.0 (d, *J* = 47.6 Hz), 67.7, 67.0 (d, *J* = 4.7 Hz), 55.4 (d, *J* = 80.4 Hz), 46.4 (d, *J* = 10.7 Hz), 14.8 ppm, one resonance was not observed due to overlapping peaks; **^{31}P NMR** (162 MHz, Chloroform-*d*) δ 27.90 ppm; **HRMS** calc'd for $C_{18}H_{23}NO_2PS_2^+$ 380.0902, found 380.0900 $[M+H]^+$.

Bis(4-fluorophenyl)(morpholin-3-yl)phosphine oxide (3ag):

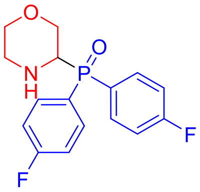

The reaction was performed following the **General Procedure G** with morpholino benzoate **1a** (165.7 mg, 0.8 mmol) and bis(4-fluorophenyl)phosphine oxide **2g** (95.2 mg, 0.4 mmol). The crude product was separated by flash chromatography on deactivated silica gel (hexane:ethyl acetate = 1:1 to ethyl acetate:methanol = 20:1) to give the product **3ag** (113.7 mg, 88% yield) as a light yellow oil. $\mathbf{R}_f$ = 0.30 (ethyl acetate:methanol = 20:1). **^{1}H NMR** (400 MHz, Chloroform-*d*) δ 7.93 – 7.85 (m, 2H), 7.78 – 7.70 (m, 2H), 7.19 – 7.13 (m, 4H), 3.88 – 3.84 (m, 1H), 3.77 – 3.69 (m, 2H), 3.53 (td, *J* = 11.2, 3.6 Hz, 1H), 3.43 (td, *J* = 10.8, 3.6 Hz, 1H), 2.95 – 2.85 (m, 2H), 1.96 (s, 1H) ppm; **^{13}C{^{1}H} NMR** (100 MHz, Chloroform-*d*) δ 166.6 (dd, *J* = 4.5, 3.4 Hz), 164.1 (dd, *J* = 4.6, 3.2 Hz), 134.3 (dd, *J* = 10.3, 8.6 Hz), 133.8 (dd, *J* = 10.7, 8.8 Hz), 126.4 (dd, *J* = 52.3, 3.6 Hz), 125.4 (dd, *J* = 53.3, 3.4 Hz), 116.5 (dd, *J* = 14.3, 7.1 Hz), 116.2 (dd, *J* = 14.3, 7.1 Hz), 67.6, 66.7 (d, *J* = 5.0 Hz), 55.4 (d, *J* = 81.0 Hz), 46.3 (d, *J* = 10.9 Hz) ppm; **^{31}P NMR** (162 MHz, Chloroform-*d*) δ 26.86 ppm; **^{19}F NMR** (376 MHz, Chloroform-*d*) δ -105.52, -105.62 ppm; **HRMS** calc'd for $C_{16}H_{17}F_2NO_2P^+$ 324.0959, found 324.0954 $[M+H]^+$.

Bis(4-chlorophenyl)(morpholin-3-yl)phosphine oxide (3ah):

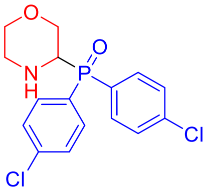

The reaction was performed following the **General Procedure G** with morpholino benzoate **1a** (165.7 mg, 0.8 mmol) and bis(4-chlorophenyl)phosphine oxide **2h** (108.0 mg, 0.4 mmol). The crude product was separated by flash chromatography on deactivated silica gel (hexane:ethyl acetate = 2:1 to ethyl acetate:methanol = 20:1) to give the product **3ah** (79.5 mg, 56% yield) as a light yellow oil. **R*f*** = 0.43 (ethyl acetate:methanol = 20:1). **^{1}H NMR** (400 MHz, Chloroform-*d*) δ 7.84 – 7.80 (m, 2H), 7.70 – 7.65 (m, 2H), 7.48 – 7.43 (m, 4H), 3.88 – 3.84 (m 1H), 3.78 – 3.70 (m, 2H), 3.55 (td, *J* = 10.8, 3.3 Hz, 1H), 3.44 (td, *J* = 10.8, 4.0 Hz, 1H), 2.96 – 2.86 (m, 2H), 2.02 (s, 1H) ppm; **^{13}C{^{1}H} NMR** (100 MHz, Chloroform-*d*) δ 139.31, 139.28, 133.1 (d, *J* = 9.6 Hz), 132.7 (d, *J* = 10.1 Hz), 129.4 (d, *J* = 12.5 Hz), 129.3 (d, *J* = 12.5 Hz), 128.8 (d, *J* = 48.5 Hz), 127.8 (d, *J* = 49.6 Hz), 67.6, 66.6 (d, *J* = 5.0 Hz), 55.2 (d, *J* = 80.6 Hz), 46.2 (d, *J* = 10.8 Hz) ppm; **^{31}P NMR** (162 MHz, Chloroform-*d*) δ 27.00 ppm; **HRMS** calc'd for $C_{16}H_{17}Cl_2NO_2P^+$ 356.0368, found 356.0365 $[M+H]^+$.

Morpholin-3-ylbis(4-(trifluoromethyl)phenyl)phosphine oxide (3ai):

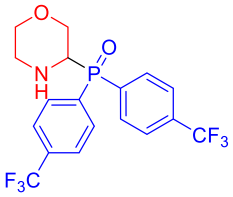

The reaction was performed following the **General Procedure G** with morpholino benzoate **1a** (165.7 mg, 0.8 mmol) and bis(4-(trifluoromethyl)phenyl)phosphine oxide **2i** (135.2 mg, 0.4 mmol). The crude product was separated by flash chromatography on deactivated silica gel (hexane:ethyl acetate = 3:1 to hexane:ethyl acetate = 1:1) to give the product **3ai** (128.6 mg, 76% yield) as a white solid. **Mp**: 159 – 160 °C. **R*f*** = 0.23 (hexane:ethyl acetate = 1:1). **^{1}H NMR** (400 MHz, Chloroform-*d*) δ 8.09 – 8.05 (m, 2H), 7.96 – 7.91 (m, 2H), 7.79 – 7.75 (m, 4H), 3.92 – 3.77 (m, 3H), 3.62 (td, *J* = 10.8, 4.0 Hz, 1H), 3.49 (td, *J* = 10.4, 4.0 Hz, 1H), 3.01 – 2.91 (m, 2H), 1.97 (s, 1H) ppm; **^{13}C{^{1}H} NMR** (100 MHz, Chloroform-*d*) δ 134.59 (q, *J* = 38.1 Hz), 134.58 (d, *J* = 43.2 Hz), 134.56 (q, *J* = 37.9 Hz), 133.6 (d, *J* = 42.7 Hz), 133.4 (d, *J* = 9.3 Hz), 131.9 (d, *J* = 9.5 Hz), 125.8 (dq, *J* = 11.6, 3.6 Hz), 125.7 (dq, *J* = 11.3, 3.4 Hz), 123.4 (q, *J* = 271.1 Hz), 123.3 (q, *J* = 271.3 Hz), 67.8, 66.6 (d, *J* = 5.1 Hz), 55.2 (d, *J* = 80.2 Hz), 46.2 (d, *J* = 10.7 Hz) ppm; **^{31}P NMR** (162 MHz, Chloroform-*d*) δ 25.95 ppm; **^{19}F NMR** (376 MHz, Chloroform-*d*) δ -63.35 ppm; **HRMS** calc'd for $C_{18}H_{17}F_6NO_2P^+$ 424.0896, found 424.0893 $[M+H]^+$.

Morpholin-3-yldi-*m*-tolylphosphine oxide (3aj):

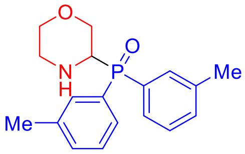

The reaction was performed following the **General Procedure G** with morpholino benzoate **1a** (165.7 mg, 0.8 mmol) and di-*m*-tolylphosphine oxide **2j** (92.0 mg, 0.4 mmol). The crude product was separated by flash chromatography on deactivated silica gel (hexane:ethyl acetate = 2:1 to ethyl acetate:methanol = 20:1) to give the product **3aj** (59.2 mg, 47% yield) as a light yellow oil. **R*f*** = 0.28 (ethyl acetate:methanol = 20:1). **^{1}H NMR** (400 MHz, Chloroform-*d*) δ 7.72 – 7.59 (m, 3H), 7.54 – 7.49 (m, 1H), 7.38 – 7.30 (m, 4H), 3.89 – 3.85 (m, 1H), 3.77 – 3.73 (m, 2H), 3.63 (td, *J* = 10.8, 3.2 Hz, 1H), 3.47 (td, *J* = 10.8, 3.6 Hz, 1H), 2.97 – 2.86 (m, 2H), 2.36 (s, 3H), 2.35 (s, 3H), 2.07 (s, 1H) ppm; **^{13}C{^{1}H} NMR** (100 MHz, Chloroform-*d*) δ 138.8 (d, *J* = 6.1 Hz), 138.7 (d, *J* = 6.2 Hz), 133.12, 133.09, 132.2 (d,

J = 8.7 Hz), 131.7 (d, J = 8.8 Hz), 130.5 (d, J = 37.7 Hz), 129.6 (d, J = 38.8 Hz), 128.7 (d, J = 7.6 Hz), 128.6 (d, J = 7.6 Hz), 128.4 (d, J = 9.3 Hz), 128.2 (d, J = 9.5 Hz), 67.6, 66.9 (d, J = 4.8 Hz), 55.1 (d, J = 79.2 Hz), 46.4 (d, J = 10.9 Hz), 21.51, 21.48 ppm; **^{31}P NMR** (162 MHz, Chloroform-*d*) δ 27.88 ppm; **HRMS** calc'd for $C_{18}H_{23}NO_2P^+$ 316.1461, found 316.1460 $[M+H]^+$.

Bis(3-fluorophenyl)(morpholin-3-yl)phosphine oxide (3ak):

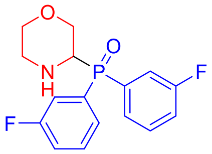

The reaction was performed following the **General Procedure G** with morpholino benzoate **1a** (165.7 mg, 0.8 mmol) and bis(3-fluorophenyl)phosphine oxide **2k** (95.2 mg, 0.4 mmol). The crude product was separated by flash chromatography on deactivated silica gel (hexane:ethyl acetate = 2:1 to ethyl acetate:methanol = 20:1) to give the product **3ak** (94.3 mg, 73% yield) as a light yellow oil. **R_f** = 0.41 (ethyl acetate:methanol = 20:1). **^{1}H NMR** (400 MHz, Chloroform-*d*) δ 7.69 – 7.61 (m, 2H), 7.55 – 7.43 (m, 4H), 7.27 – 7.20 (m, 2H), 3.89 – 3.85 (m, 1H), 3.78 – 3.73 (m, 2H), 3.59 (td, J = 10.8, 3.2 Hz, 1H), 3.46 (td, J = 10.8, 4.0 Hz 1H), 2.97 – 2.87 (m, 2H), 1.93 (s, 1H) ppm; **^{13}C{^{1}H} NMR** (100 MHz, Chloroform-*d*) δ 163.9 (dd, J = 16.2, 8.7 Hz), 161.4 (dd, J = 16.2, 8.2 Hz), 132.9 (dd, J = 51.9, 5.6 Hz), 131.9 (dd, J = 53.3, 5.5 Hz), 131.1 (dd, J = 13.5, 7.4 Hz), 130.9 (dd, J = 13.5, 7.4 Hz), 127.4 (dd, J = 8.5, 3.3 Hz), 126.9 (dd, J = 8.7, 3.3 Hz), 120.0 (d, J = 2.7 Hz), 119.7 (d, J = 2.6 Hz), 118.7 (dd, J = 22.5, 9.8 Hz), 118.3 (dd, J = 22.4, 10.0 Hz), 67.6, 66.6 (d, J = 5.0 Hz), 55.2 (d, J = 80.6 Hz), 46.2 (d, J = 10.9 Hz) ppm; **^{31}P NMR** (162 MHz, Chloroform-*d*) δ 25.91 ppm; **^{19}F NMR** (376 MHz, Chloroform-*d*) δ -110.00, -110.22 ppm; **HRMS** calc'd for $C_{16}H_{17}F_2NO_2P^+$ 324.0959, found 324.0954 $[M+H]^+$.

Bis(3-chlorophenyl)(morpholin-3-yl)phosphine oxide (3al):

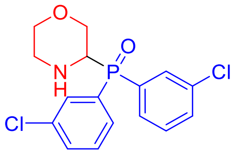

The reaction was performed following the **General Procedure G** with morpholino benzoate **1a** (165.7 mg, 0.8 mmol) and bis(3-chlorophenyl)phosphine oxide **2l** (108.0 mg, 0.4 mmol). The crude product was separated by flash chromatography on deactivated silica gel (hexane:ethyl acetate = 2:1 to ethyl acetate:methanol = 20:1) to give the product **3al** (100.8 mg, 71% yield) as a light yellow oil. **R_f** = 0.42 (ethyl acetate:methanol = 20:1). **^{1}H NMR** (400 MHz, Chloroform-*d*) δ 7.93 – 7.89 (m, 1H), 7.80 – 7.75 (m, 2H), 7.66 – 7.61 (m, 1H), 7.56 – 7.52 (m, 2H), 7.48 – 7.42 (m, 2H), 3.89 (m, 1H), 3.81 – 3.75 (m, 2H), 3.61 (td, J = 10.8, 3.6 Hz, 1H), 3.48 (td, J = 10.8, 4.4 Hz, 1H), 3.00 – 2.91 (m, 2H), 1.86 (s, 1H) ppm; **^{13}C{^{1}H} NMR** (100 MHz, Chloroform-*d*) δ 135.6 (d, J = 14.9 Hz), 135.5 (d, J = 15.0 Hz), 132.91, 132.89, 132.6 (d, J = 45.4 Hz), 131.7 (d, J = 47.0 Hz), 131.6 (d, J = 9.7 Hz), 131.2 (d, J = 9.9 Hz), 130.5 (d, J = 12.4 Hz), 130.4 (d, J = 12.5 Hz), 129.7 (d, J = 8.7 Hz), 129.3 (d, J = 8.9 Hz), 67.7, 66.7 (d, J = 5.1 Hz), 55.2 (d, J = 80.4 Hz), 46.3 (d, J = 10.9 Hz) ppm; **^{31}P NMR** (162 MHz, Chloroform-*d*) δ 25.89 ppm; **HRMS** calc'd for $C_{16}H_{17}Cl_2NO_2P^+$ 356.0368, found 356.0364 $[M+H]^+$.

Morpholin-3-ylbis(3-(trifluoromethyl)phenyl)phosphine oxide (3am):

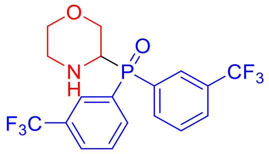

The reaction was performed following the **General Procedure G** with morpholino benzoate **1a** (165.7 mg, 0.8 mmol) and bis(3-(trifluoromethyl)phenyl)phosphine oxide **2m** (135.2 mg, 0.4 mmol). The crude product was separated by flash chromatography on deactivated silica gel (hexane:ethyl acetate = 2:1 to ethyl acetate:methanol = 20:1) to give the product **3am** (106.6 mg, 63% yield) as a light yellow oil. $\mathbf{R}_f$ = 0.36 (ethyl acetate:methanol = 20:1). **^{1}H NMR** (400 MHz, Chloroform-*d*) δ 8.25 (d, *J* = 15.6 Hz, 1H), 8.12 – 8.07 (m, 2H), 7.94 (m, 1H), 7.85 – 7.82 (m, 2H), 7.69 – 7.63 (m, 2H), 3.90 – 3.76 (m, 3H), 3.60 (td, *J* = 10.4, 4.4 Hz, 1H), 3.47 (td, *J* = 11.2, 4.0 Hz, 1H), 3.00 – 2.91 (m, 2H), 1.90 (s, 1H) ppm; **^{13}C{^{1}H} NMR** (100 MHz, Chloroform-*d*) δ 134.9 (d, *J* = 9.2 Hz), 134.4 (d, *J* = 9.2 Hz), 131.8 (dq, *J* = 50.9, 6.1 Hz), 131.7 (d, *J* = 47.9 Hz), 131.6 (dq, *J* = 50.7, 6.0 Hz), 130.8 (d, *J* = 49.7 Hz), 129.8 (d, *J* = 11.5 Hz), 129.6 (d, *J* = 9.6 Hz), 129.58 – 129.53 (m), 129.52 – 129.4 (m), 128.8 (dq, *J* = 10.7, 3.7 Hz), 128.2 (dq, *J* = 10.5, 3.8 Hz), 123.6 (d, *J* = 271.2 Hz), 123.5 (d, *J* = 271.6 Hz), 67.7, 66.5 (d, *J* = 5.2 Hz), 55.2 (d, *J* = 80.7 Hz), 46.2 (d, *J* = 10.7 Hz) ppm; **^{31}P NMR** (162 MHz, Chloroform-*d*) δ 25.86 ppm; **^{19}F NMR** (376 MHz, Chloroform-*d*) δ -62.86, -62.87 ppm; **HRMS** calc'd for $C_{18}H_{17}F_6NO_2P^+$ 424.0896, found 424.0894 $[M+H]^+$.

Bis(3,5-dimethylphenyl)(morpholin-3-yl)phosphine oxide (3an):

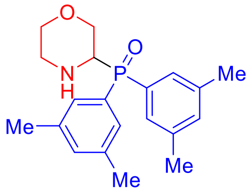

The reaction was performed following the **General Procedure G** with morpholino benzoate **1a** (165.7 mg, 0.8 mmol) and bis(3,5-dimethylphenyl)phosphine oxide **2n** (103.2 mg, 0.4 mmol). The crude product was separated by flash chromatography on deactivated silica gel (hexane:ethyl acetate = 2:1 to ethyl acetate:methanol = 20:1) to give the product **3an** (57.7 mg, 42% yield) as a colorless oil. $\mathbf{R}_f$ = 0.30 (ethyl acetate:methanol = 20:1). **^{1}H NMR** (400 MHz, Chloroform-*d*) δ 7.49 – 7.46 (m, 2H), 7.39 – 7.35 (m, 2H), 7.15 – 7.13 (m, 2H), 3.88 – 3.84 (m, 1H), 3.78 – 3.72 (m, 2H), 3.65 (td, *J* = 11.2, 3.2 Hz, 1H), 3.49 (td, *J* = 10.8, 3.6 Hz, 1H), 2.99 – 2.88 (m, 2H), 2.33 (s, 6H), 2.32 (s, 6H), 1.98 (s, 1H) ppm; **^{13}C{^{1}H} NMR** (100 MHz, Chloroform-*d*) δ 138.6 (d, *J* = 6.0 Hz), 138.5 (d, *J* = 6.2 Hz), 134.1 (d, *J* = 3.3 Hz), 134.0 (d, *J* = 3.3 Hz), 130.6 (d, *J* = 28.5 Hz), 129.6 (d, *J* = 29.6 Hz), 129.0 (d, *J* = 9.0 Hz), 128.7 (d, *J* = 9.0 Hz), 67.7, 67.0 (d, *J* = 4.8 Hz), 54.9 (d, *J* = 78.9 Hz), 46.4 (d, *J* = 10.7 Hz), 21.5, 21.4 ppm; **^{31}P NMR** (162 MHz, Chloroform-*d*) δ 27.90 ppm; **HRMS** calc'd for $C_{20}H_{27}NO_2P^+$ 344.1774, found 344.1773 $[M+H]^+$.

Bis(3,5-difluorophenyl)(morpholin-3-yl)phosphine oxide (3ao):

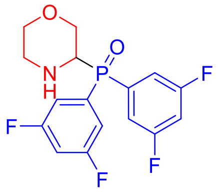

The reaction was performed following the **General Procedure G** with morpholino benzoate **1a** (165.7 mg, 0.8 mmol) and bis(3,5-difluorophenyl)phosphine oxide **2o** (109.6 mg, 0.4 mmol). The crude product was separated by flash chromatography on deactivated silica gel (hexane:ethyl acetate = 3:1 to hexane:ethyl acetate = 1:1) to give the product **3ao** (87.6 mg, 61% yield) as a white solid. **Mp**: 169 – 171 °C. **R*f*** = 0.28 (hexane:ethyl acetate = 1:1). **^{1}H NMR** (400 MHz, Chloroform-*d*) δ 7.51 – 7.46 (m, 2H), 7.35 – 7.30 (m, 2H), 7.07 – 7.00 (m, 2H), 3.91 (m, 1H), 3.83 – 3.74 (m, 2H), 3.61 (td, *J* = 10.8, 2.8 Hz, 1H), 3.49 (td, *J* = 11.2, 4.4 Hz, 1H), 3.01 – 2.93 (m, 2H), 1.89 (s, 1H) ppm; **^{13}C{^{1}H} NMR** (100 MHz, Chloroform-*d*) δ 164.4 (dt, *J* = 19.1, 11.4 Hz), 161.9 (dt, *J* = 19.2, 11.0 Hz), 134.1 (dt, *J* = 56.4, 7.1 Hz), 133.1 (dt, *J* = 58.3, 6.9 Hz), 114.9 – 114.6 (m), 114.4 – 114.0 (m), 108.52 (t, *J* = 24.9 Hz), 108.50 (t, *J* = 24.9 Hz), 67.7, 66.3 (d, *J* = 5.4 Hz), 55.1 (d, *J* = 81.7 Hz), 46.1 (d, *J* = 11.0 Hz) ppm; **^{31}P NMR** (162 MHz, Chloroform-*d*) δ 24.96 ppm; **^{19}F NMR** (376 MHz, Chloroform-*d*) δ -105.63, -105.65, -105.96, -105.98 ppm; **HRMS** calc'd for $C_{16}H_{15}F_4NO_2P^+$ 360.0771, found 360.0776 [M+H]$^+$.

Bis(3-fluoro-4-methylphenyl)(morpholin-3-yl)phosphine oxide (3ap):

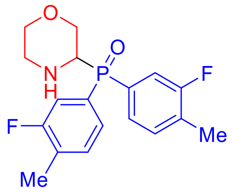

The reaction was performed following the **General Procedure G** with morpholino benzoate **1a** (165.7 mg, 0.8 mmol) and bis(3-fluoro-4-methylphenyl)phosphine oxide **2p** (106.4 mg, 0.4 mmol). The crude product was separated by flash chromatography on deactivated silica gel (hexane:ethyl acetate = 3:1 to ethyl acetate:methanol = 20:1) to give the product **3ap** (75.8 mg, 54% yield) as a light yellow oil. **R*f*** = 0.39 (ethyl acetate:methanol = 20:1). **^{1}H NMR** (400 MHz, Chloroform-*d*) δ 7.77 – 7.60 (m, 3H), 7.56 – 7.49 (m, 1H), 7.14 – 7.07 (m, 2H), 3.89 – 3.85 (m, 1H), 3.79 – 3.69 (m, 2H), 3.58 (td, *J* = 10.8, 3.2 Hz, 1H), 3.46 (td, *J* = 10.8, 4.0 Hz, 1H), 2.98 – 2.88 (m, 2H), 2.30 (s, 3H), 2.29 (s, 3H), 1.94 (s, 1H) ppm; **^{13}C{^{1}H} NMR** (100 MHz, Chloroform-*d*) δ 165.3 (t, *J* = 3.7 Hz), 162.7 (t, *J* = 3.8 Hz), 135.5 (dd, *J* = 9.8, 6.1 Hz), 135.0 (dd, *J* = 10.0, 6.1 Hz), 131.2 (dd, *J* = 10.5, 8.8 Hz), 130.9 (dd, *J* = 10.3, 8.8 Hz), 126.5 (dd, *J* = 11.9, 5.9 Hz), 126.2 (dd, *J* = 11.6, 5.8 Hz), 126.1 (dd, *J* = 45.4, 4.0 Hz), 125.1 (dd, *J* = 46.5, 3.9 Hz), 116.0 (dd, *J* = 13.1, 11.1 Hz), 115.7 (dd, *J* = 11.5, 9.2 Hz), 67.7, 66.9 (d, *J* = 5.0 Hz), 55.3 (d, *J* = 80.3 Hz), 46.4 (d, *J* = 10.7 Hz), 14.69 (d, *J* = 1.7 Hz), 14.66 (d, *J* = 1.6 Hz) ppm; **^{31}P NMR** (162 MHz, Chloroform-*d*) δ 26.92 ppm; **^{19}F NMR** (376 MHz, Chloroform-*d*) δ -110.07, -110.18 ppm; **HRMS** calc'd for $C_{18}H_{21}F_2NO_2P^+$ 352.1272, found 352.1269 [M+H]$^+$.

Morpholin-3-yldi(naphthalen-2-yl)phosphine oxide (3aq):

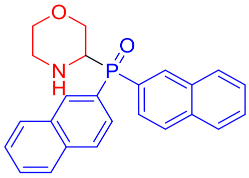

The reaction was performed following the **General Procedure G** with morpholino benzoate **1a** (165.7 mg, 0.8 mmol) and di(naphthalen-2-yl)phosphine oxide **2q** (120.8 mg, 0.4 mmol). The crude product was separated by flash chromatography on deactivated silica gel (hexane:ethyl acetate = 2:1 to ethyl acetate:methanol = 20:1) to give the product **3aq** (92.9 mg, 60% yield) as a light yellow oil. **R*f*** = 0.41

(ethyl acetate:methanol = 20:1). **^{1}H NMR** (400 MHz, Chloroform-*d*) δ 8.59 (d, *J* = 3.3 Hz, 1H), 8.46 (d, *J* = 3.4, 1H), 7.97 – 7.86 (m, 7H), 7.81 – 7.76 (m, 1H), 7.62 – 7.53 (m, 4H), 4.04 – 3.98 (m, 2H), 3.82 – 3.78 (m, 1H), 3.74 (td, *J* = 11.6, 3.2 Hz, 1H), 3.52 (td, *J* = 11.2, 2.8 Hz, 1H), 3.04 – 2.91 (m, 2H), 2.10 (s, 1H) ppm; **^{13}C{^{1}H} NMR** (100 MHz, Chloroform-*d*) δ 135.0 (d, *J* = 2.5 Hz), 134.9 (d, *J* = 2.5 Hz), 134.1 (d, *J* = 8.1 Hz), 133.7 (d, *J* = 8.3 Hz), 132.7 (d, *J* = 3.0 Hz), 132.6 (d, *J* = 3.0 Hz), 129.1, 129.0, 128.8 (d, *J* = 11.6 Hz), 128.7 (d, *J* = 11.4 Hz), 128.54, 128.50, 128.0, 127.94, 127.86 (d, *J* = 34.0 Hz), 127.2, 127.1, 126.9 (d, *J* = 35.0 Hz), 126.2 (d, *J* = 10.0 Hz), 125.8 (d, *J* = 10.3 Hz), 67.7, 67.1 (d, *J* = 4.7 Hz), 55.3 (d, *J* = 79.6 Hz), 46.5 (d, *J* = 10.8 Hz) ppm; **^{31}P NMR** (162 MHz, Chloroform-*d*) δ 28.17 ppm; **HRMS** calc'd for $C_{24}H_{23}NO_2P^+$ 388.1461, found 388.1457 $[M+H]^+$.

Di(benzofuran-5-yl)(morpholin-3-yl)phosphine oxide (3ar):

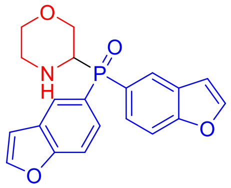

The reaction was performed following the **General Procedure G** with morpholino benzoate **1a** (165.7 mg, 0.8 mmol) and di(benzofuran-5-yl)phosphine oxide **2r** (112.8 mg, 0.4 mmol). The crude product was separated by flash chromatography on deactivated silica gel (hexane:ethyl acetate = 2:1 to ethyl acetate:methanol = 20:1) to give the product **3ar** (82.2 mg, 56% yield) as a light yellow oil. **R_f** = 0.35 (ethyl acetate:methanol = 20:1). **^{1}H NMR** (400 MHz, Chloroform-*d*) δ 8.25 (d, *J* = 10.0 Hz, 1H), 8.11 (d, *J* = 10.4 Hz, 1H), 7.88 – 7.83 (m, 1H), 7.73 – 7.68 (m, 3H), 7.63 – 7.58 (m, 2H), 6.83 – 6.80 (m, 2H), 3.96 – 3.92 (m, 1H), 3.90 – 3.84 (m, 1H), 3.80 – 3.75 (m, 1H), 3.65 (td, *J* = 10.8, 3.2 Hz, 1H), 3.49 (td, *J* = 11.2, 3.2 Hz, 1H), 3.01 – 2.89 (m, 2H), 2.13 (s, 1H) ppm; **^{13}C{^{1}H} NMR** (100 MHz, Chloroform-*d*) δ 157.02 (d, *J* = 2.8 Hz), 156.96 (d, *J* = 2.9 Hz), 146.5, 146.4, 128.1 (d, *J* = 6.1 Hz), 128.0 (d, *J* = 6.2 Hz), 127.5 (d, *J* = 11.2 Hz), 127.0 (d, *J* = 11.5 Hz), 126.2 (d, *J* = 10.3 Hz), 125.7 (d, *J* = 10.7 Hz), 125.1 (d, *J* = 49.7 Hz), 124.1 (d, *J* = 50.6 Hz), 112.3 (d, *J* = 11.2 Hz), 112.1 (d, *J* = 11.2 Hz), 107.0 106.9, 67.7, 67.1 (d, *J* = 4.8 Hz), 55.7 (d, *J* = 79.8 Hz), 46.5 (d, *J* = 10.8 Hz) ppm; **^{31}P NMR** (162 MHz, Chloroform-*d*) δ 29.59 ppm; **HRMS** calc'd for $C_{20}H_{19}NO_4P^+$ 368.1046, found 368.1050 $[M+H]^+$.

Bis(benzo[*d*][1,3]dioxol-5-yl)(morpholin-3-yl)phosphine oxide (3as):

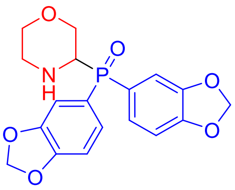

The reaction was performed following the **General Procedure G** with morpholino benzoate **1a** (165.7 mg, 0.8 mmol) and bis(benzo[*d*][1,3]dioxol-5-yl)phosphine oxide **2s** (116.0 mg, 0.4 mmol). The crude product was separated by flash chromatography on deactivated silica gel (hexane:ethyl acetate = 1:1 to ethyl acetate:methanol = 20:1) to give the product **3as** (87.0 mg, 58% yield) as a light yellow oil. **R_f** = 0.25 (ethyl acetate:methanol = 20:1). **^{1}H NMR** (400 MHz, Chloroform-*d*) δ 7.43 (m, 1H), 7.34 (m, 1H), 7.31 – 7.27 (m, 1H), 7.18 – 7.15 (m, 1H), 6.93 – 6.88 (m, 2H), 6.02 (s, 2H), 6.01 (s, 2H), 3.92 – 3.88 (m, 1H), 3.79 – 3.74 (m, 1H), 3.70 – 3.64 (m, 1H), 3.58 (td, *J* = 10.8, 2.8 Hz, 1H), 3.47 (td, *J* = 11.8, 4.0 Hz, 1H), 2.98 – 2.88 (m, 2H), 1.98 (s, 1H) ppm; **^{13}C{^{1}H} NMR** (100 MHz, Chloroform-*d*) δ 151.25 (d, *J* =

2.6 Hz), 151.22 (d, J = 2.7 Hz), 148.3 (d, J = 14.9 Hz), 148.2 (d, J = 15.1 Hz), 127.3 (d, J = 9.9 Hz), 126.8 (d, J = 10.2 Hz), 123.7 (d, J = 60.4 Hz), 122.7 (d, J = 61.7 Hz), 111.2 (d, J = 11.8 Hz), 110.8 (d, J = 12.1 Hz), 109.1 (d, J = 5.1 Hz), 109.0 (d, J = 5.1 Hz), 101.83, 101.79, 67.7, 67.0 (d, J = 4.9 Hz), 55.6 (d, J = 80.9 Hz), 46.5 (d, J = 10.8 Hz) ppm; **^{31}P NMR** (162 MHz, Chloroform-*d*) δ 28.28 ppm; **HRMS** calc'd for $C_{13}H_{19}NO_6P^+$ 376.0945, found 376.0947 $[M+H]^+$.

Morpholin-3-yldi(thiophen-2-yl)phosphine oxide (3at):

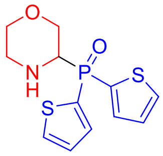

The reaction was performed following the **General Procedure G** with morpholino benzoate **1a** (165.7 mg, 0.8 mmol) and di(thiophen-2-yl)phosphine oxide **2t** (85.6 mg, 0.4 mmol). The crude product was separated by flash chromatography on deactivated silica gel (hexane:ethyl acetate = 2:1 to ethyl acetate:methanol = 20:1) to give the product **3at** (83.7 mg, 70% yield) as a light yellow oil. **R*f*** = 0.38 (ethyl acetate:methanol = 20:1). **^{1}H NMR** (400 MHz, Chloroform-*d*) δ 7.81 – 7.75 (m, 3H), 7.65 (m, 1H), 7.25 – 7.20 (m, 2H), 4.06 – 4.02 (m, 1H), 3.80 – 3.76 (m, 1H), 3.70 – 3.57 (m, 2H), 3.53 – 3.47 (m, 1H), 3.00 – 2.92 (m, 2H), 2.01 (s, 1H) ppm; **^{13}C{^{1}H} NMR** (100 MHz, Chloroform-*d*) δ 137.5 (d, J = 9.4 Hz), 136.8 (d, J = 9.7 Hz), 134.6 (d, J = 4.4 Hz), 134.4 (d, J = 4.7 Hz), 131.0 (d, J = 110.6 Hz), 129.7 (d, J = 111.3 Hz), 128.6 (d, J = 13.9 Hz), 128.4 (d, J = 14.1 Hz), 67.4, 66.7 (d, J = 4.4 Hz), 57.3 (d, J = 90.4 Hz), 46.2 (d, J = 12.2 Hz) ppm; **^{31}P NMR** (162 MHz, Chloroform-*d*) δ 19.57 ppm; **HRMS** calc'd for $C_{12}H_{15}NO_2PS_2^+$ 300.0276, found 300.0276 $[M+H]^+$.

Morpholin-3-yl(phenyl)(*p*-tolyl)phosphine oxide (3au):

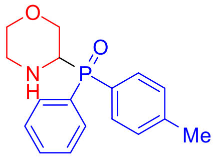

The reaction was performed following the **General Procedure G** with morpholino benzoate **1a** (165.7 mg, 0.8 mmol) and phenyl(*p*-tolyl)phosphine oxide **2u** (86.4 mg, 0.4 mmol). The crude product was separated by flash chromatography on deactivated silica gel (hexane:ethyl acetate = 3:1 to ethyl acetate:methanol = 20:1) to give the product **3au** (66.2 mg, 55% yield, dr = 1:1) as a light yellow oil. **R*f*** = 0.34 (ethyl acetate:methanol = 20:1). **^{1}H NMR** (400 MHz, Chloroform-*d*) δ 7.90 – 7.85 (m, 1H), 7.79 – 7.72 (m, 2H), 7.66 – 7.61 (m, 1H), 7.54 – 7.41 (m, 3H), 7.29 – 7.24 (m, 2H), 3.89 – 3.85 (m, 1H), 3.76 – 3.71 (m, 2H), 3.59 (td, J = 10.8, 3.2 Hz, 1H), 3.45 (td, J = 12.0, 3.2 Hz, 1H), 2.95 – 2.84 (m, 2H), 2.36 (s, 1.5H), 2.35 (s, 1.5H), 1.99 (s, 1H) ppm; **^{13}C{^{1}H} NMR** (100 MHz, Chloroform-*d*) δ 142.92, 142.89, 132.23, 132.20, 131.7 (d, J = 9.3 Hz), 131.6 (d, J = 9.0 Hz), 131.30 (d, J = 9.5 Hz), 131.27 (d, J = 9.1 Hz), 130.9 (d, J = 41.4 Hz), 130.0 (d, J = 42.6 Hz), 129.8 (d, J = 9.0 Hz), 129.7 (d, J = 9.1 Hz), 129.6 (d, J = 12.0 Hz), 129.5 (d, J = 12.0 Hz), 127.2 (d, J = 45.1 Hz), 126.2 (d, J = 46.2 Hz), 67.6, 66.9 (d, J = 4.9 Hz), 55.3 (d, J = 79.5 Hz), 46.4 (d, J = 1.4 Hz), 46.3 (d, J = 1.3 Hz), 21.65 (d, J = 1.5 Hz), 21.62 (d, J = 1.4 Hz) ppm; **^{31}P NMR** (162 MHz, Chloroform-*d*) δ 27.94 ppm; **HRMS** calc'd for $C_{17}H_{21}NO_2P^+$ 302.1304, found 302.1301 $[M+H]^+$.

Morpholin-3-yl(phenyl)(thiophen-2-yl)phosphine oxide (3av):

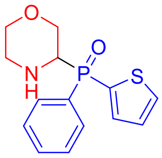

The reaction was performed following the **General Procedure G** with morpholino benzoate **1a** (165.7 mg, 0.8 mmol) and phenyl(thiophen-2-yl)phosphine oxide **2v** (83.2 mg, 0.4 mmol). The crude product was separated by flash chromatography on deactivated silica gel (hexane:ethyl acetate = 1:1 to ethyl acetate:methanol = 20:1) to give the product **3av** (58.6 mg, 50% yield, dr = 1:1) as a light yellow oil. $\mathbf{R}_f$ = 0.32 (ethyl acetate:methanol = 20:1). ^{1}**H NMR** (400 MHz, Chloroform-*d*) δ 7.95 – 7.89 (m, 1H), 7.82 – 7.70 (m, 2.5H), 7.62 – 7.45 (m, 3.5H), 7.22 – 7.17 (m, 1H), 4.01 – 3.89 (m, 1H), 3.78 – 3.73 (m, 1H), 3.71 – 3.54 (m, 2H), 3.51 – 3.44 (m, 1H), 2.98 – 2.86 (m, 2H), 1.86 (s, 1H) ppm; ^{13}**C{^{1}H} NMR** (100 MHz, Chloroform-*d*) δ 137.2 (d, *J* = 8.9 Hz), 136.6 (d, *J* = 9.1 Hz), 134.2 (d, *J* = 4.1 Hz), 134.1 (d, *J* = 4.2 Hz), 132.68 (d, *J* = 2.0 Hz), 132.65 (d, *J* = 2.6 Hz), 131.6 (d, *J* = 9.3 Hz), 131.3 (d, *J* = 9.6 Hz), 131.0 (d, *J* = 40.1 Hz), 130.3 (d, *J* = 6.4 Hz), 130.0 (d, *J* = 38.8 Hz), 129.3 (d, *J* = 7.6 Hz), 128.9 (d, *J* = 7.7 Hz), 128.8 (d, *J* = 7.7 Hz), 128.5 (d, *J* = 13.4 Hz), 128.3 (d, *J* = 13.6 Hz), 67.53, 67.47, 66.8 (d, *J* = 4.2 Hz), 66.7 (d, *J* = 5.0 Hz), 57.0 (d, *J* = 50.8 Hz), 56.1 (d, *J* = 50.7 Hz), 46.4 (d, *J* = 11.5 Hz), 46.2 (d, *J* = 11.4 Hz) ppm; ^{31}**P NMR** (162 MHz, Chloroform-*d*) δ 24.55, 23.32 ppm; **HRMS** calc'd for $C_{14}H_{17}NO_2PS^+$ 294.0712, found 294.0717 $[M+H]^+$.

Diphenyl(pyrrolidin-2-yl)phosphine oxide (3ba):

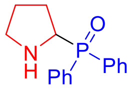

The reaction was performed following the **General Procedure G** with pyrrolidin-1-yl benzoate **1b** (229.3 mg, 1.2 mmol), $NaHCO_3$ (100.8 mg, 1.2 mmol) and diphenylphosphine oxide **2a** (80.8 mg, 0.4 mmol). The crude product was separated by flash chromatography on deactivated silica gel (hexane:ethyl acetate = 1:1 to ethyl acetate:methanol = 5:1) to give the product **3ba** (51.1 mg, 47% yield) as a colorless oil. $\mathbf{R}_f$ = 0.39 (ethyl acetate:methanol = 5:1). ^{1}**H NMR** (400 MHz, Chloroform-*d*) δ 7.95 – 7.91 (m, 2H), 7.82 – 7.77 (m, 2H), 7.53 – 7.42 (m, 6H), 3.88 – 3.83 (m, 1H), 3.02 – 2.86 (m, 2H), 2.02 – 1.85 (m, 3H), 1.76 – 1.68 (m, 2H) ppm; ^{13}**C{^{1}H} NMR** (100 MHz, Chloroform-*d*) δ 132.6 (d, *J* = 95.0 Hz), 131.944 (d, *J* = 3.1 Hz), 131.941 (d, *J* = 8.7 Hz), 131.8 (d, *J* = 2.8 Hz), 131.5 (d, *J* = 95.4 Hz), 131.2 (d, *J* = 8.7 Hz), 128.7 (d, *J* = 4.5 Hz), 128.6 (d, *J* = 4.7 Hz), 57.0 (d, *J* = 84.5 Hz), 48.5 (d, *J* = 8.7 Hz), 26.7, 26.5 (d, *J* = 6.5 Hz) ppm; ^{31}**P NMR** (162 MHz, Chloroform-*d*) δ 31.49 ppm; **HRMS** calc'd for $C_{16}H_{19}NOP^+$ 272.1199, found 272.1198 $[M+H]^+$.

Diphenyl(piperidin-2-yl)phosphine oxide (3ca):

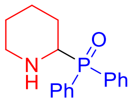

The reaction was performed following the **General Procedure G** with piperidin-1-yl benzoate **1c** (164.1 mg, 0.8 mmol) and diphenylphosphine oxide **2a** (80.8 mg, 0.4 mmol). The crude product was separated by flash chromatography on deactivated silica gel (hexane:ethyl acetate = 2:1 to ethyl acetate:methanol = 10:1) to give the product **3ca** (82.1 mg, 72% yield) as a white solid. **Mp**: 134 – 135 ºC. $\mathbf{R}_f$ = 0.41 (ethyl acetate:methanol = 10:1). ^{1}**H NMR** (400 MHz, Chloroform-*d*) δ 7.93 – 7.88 (m, 2H), 7.80 – 7.75 (m,

2H), 7.53 – 7.40 (m, 6H), 3.46 – 7.41 (m, 1H), 3.11 – 3.07 (m, 1H), 2.58 (td, J = 12.0, 2.8 Hz, 1H), 1.84 – 1.72 (m, 3H), 1.59 – 1.46 (m, 2H), 1.42 – 1.31 (m, 2H) ppm; ^{13}**C{^{1}H} NMR** (100 MHz, Chloroform-*d*) δ 131.93, 131.91 (d, J = 8.6 Hz), 131.88, 131.5 (d, J = 8.8 Hz), 131.4 (d, J = 73.1 Hz), 130.5 (d, J = 73.9 Hz), 128.6 (d, J = 7.3 Hz), 128.5 (d, J = 7.2 Hz), 56.6 (d, J = 83.8 Hz), 47.7 (d, J = 13.4 Hz), 25.9 (d, J = 78.4 Hz), 24.9, 24.8 ppm; ^{31}**P NMR** (162 MHz, Chloroform-*d*) δ 30.40 ppm; **HRMS** calc'd for $C_{17}H_{21}NOP^{+}$ 286.1355, found 286.1351 $[M+H]^{+}$.

Diphenyl(thiomorpholin-3-yl)phosphine oxide (3da):

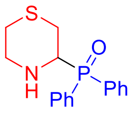

The reaction was performed following the **General Procedure G** with thiomorpholino benzoate **1d** (178.5 mg, 0.8 mmol) and diphenylphosphine oxide **2a** (80.8 mg, 0.4 mmol). The crude product was separated by flash chromatography on deactivated silica gel (hexane:ethyl acetate = 2:1 to ethyl acetate:methanol = 20:1) to give the product **3da** (67.9 mg, 56% yield) as a light yellow oil. $\mathbf{R}_f$ = 0.32 (ethyl acetate:methanol = 20:1). ^{1}**H NMR** (400 MHz, Chloroform-*d*) δ 7.89 – 7.76 (m, 4H), 7.56 – 7.43 (m, 6H), 3.83 – 3.77 (m, 1H), 3.37 – 3.32 (m, 1H), 3.02 – 2.95 (m, 1H), 2.86 – 2.78 (m, J = 13.6, 11.2, 4.0 Hz, 1H), 2.75 – 2.68 (m, 1H), 2.47 – 2.42 (m, 1H), 2.34 – 2.29 (m, 1H), 1.81 (s, 1H) ppm; ^{13}**C{^{1}H} NMR** (100 MHz, Chloroform-*d*) δ 132.30, 132.28, 131.8 (d, J = 8.8 Hz), 131.5 (d, J = 8.9 Hz), 130.8 (d, J = 34.6 Hz), 129.8 (d, J = 35.2 Hz), 128.9 (d, ,J = 10.0 Hz), 128.7 (d, ,J = 10.0 Hz), 56.9 (d, J = 77.5 Hz), 48.0 (d, J = 11.5 Hz), 27.6, 26.5 (d, J = 2.2 Hz) ppm; ^{31}**P NMR** (162 MHz, Chloroform-*d*) δ 30.14 ppm; **HRMS** calc'd for $C_{16}H_{19}NOPS^{+}$ 304.0919, found 304.0918 $[M+H]^{+}$.

Azepan-2-yldiphenylphosphine oxide (3ea):

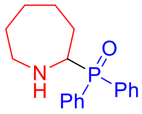

The reaction was performed following the **General Procedure G** with azepan-1-yl benzoate **1e** (175.3 mg, 0.8 mmol) and diphenylphosphine oxide **2a** (80.8 mg, 0.4 mmol). The crude product was separated by flash chromatography on deactivated silica gel (hexane:ethyl acetate = 2:1 to ethyl acetate:methanol = 30:1) to give the product **3ea** (69.4 mg, 58% yield) as a light yellow oil. $\mathbf{R}_f$ = 0.45 (ethyl acetate:methanol = 20:1). ^{1}**H NMR** (400 MHz, Chloroform-*d*) δ 7.91 – 7.85 (m, 4H), 7.52 – 7.41 (m, 6H), 3.57 – 3.51 (m, 1H), 2.97 – 2.90 (m, 1H), 2.79 – 2.73 (m, 1H), 2.21 – 2.12 (m, 1H), 1.87 – 1.39 (m, 8H) ppm; ^{13}**C{^{1}H} NMR** (100 MHz, Chloroform-*d*) δ 132.7 (d, J = 92.8 Hz), 132.2 (d, J = 8.1 Hz), 131.7 (d, J = 2.8 Hz), 131.6 (d, J = 2.6 Hz), 131.5 (d, J = 8.3 Hz), 131.3 (d, J = 94.0 Hz), 128.6 (d, J = 11.0 Hz), 128.4 (d, J = 11.0 Hz), 57.1 (d, J = 85.7 Hz), 48.2 (d, J = 13.5 Hz), 31.9, 29.6 (d, J = 3.2 Hz), 28.0 (d, J = 14.5 Hz), 27.0 ppm; ^{31}**P NMR** (162 MHz, Chloroform-*d*) δ 31.89 ppm; **HRMS** calc'd for $C_{18}H_{23}NOP^{+}$ 300.1512, found 300.1507 $[M+H]^{+}$.

(4,4-Dimethylpiperidin-2-yl)diphenylphosphine oxide (3fa):

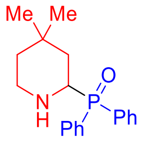

The reaction was performed following the **General Procedure G** with 4,4-dimethylpiperidin-1-yl benzoate **1f** (186.5 mg, 0.8 mmol) and diphenylphosphine oxide **2a** (80.8 mg, 0.4 mmol). The crude product was separated by flash chromatography on deactivated silica gel (hexane:ethyl acetate = 2:1 to ethyl acetate:methanol = 20:1) to give the product **3fa** (78.9 mg, 63% yield) as a white solid. **Mp**: 138 – 140 °C. **R*f*** = 0.42 (ethyl acetate:methanol = 20:1). **^{1}H NMR** (400 MHz, Chloroform-*d*) δ 7.92 – 7.87 (m, 2H), 7.82 – 7.77 (m, 2H), 7.54 – 7.43 (m, 6H), 3.68 – 3.59 (m, 1H), 2.97 – 2.92 (m, 1H), 2.84 – 2.73 (m, 1H), 1.85 (s, 1H), 1.43 – 1.39 (m, 2H), 1.29 – 1.26 (m, 2H), 0.95 (s, 3H), 0.88 (s, 3H) ppm; **^{13}C{^{1}H} NMR** (100 MHz, Chloroform-*d*) δ 132.00 (d, *J* = 3.0 Hz), 131.97 (d, *J* = 8.6 Hz), 131.96 (d, *J* = 3.2 Hz), 131.6 (d, *J* = 61.7 Hz), 131.5 (d, *J* = 8.7 Hz), 130.6 (d, *J* = 62.3 Hz), 128.7 (d, *J* = 10.8 Hz), 128.6 (d, *J* = 11.1 Hz), 52.4 (d, *J* = 84.1 Hz), 43.3 (d, *J* = 14.1 Hz), 39.1, 37.8 (d, *J* = 1.6 Hz), 33.3, 29.5 (d, *J* = 11.3 Hz), 23.7 ppm; **^{31}P NMR** (162 MHz, Chloroform-*d*) δ 31.69 ppm; **HRMS** calc'd for $C_{19}H_{25}NOP^+$ 314.1668, found 314.1671 $[M+H]^+$.

Diphenyl(1,4-dioxa-8-azaspiro[4.5]decan-7-yl)phosphine oxide (3ga):

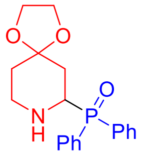

The reaction was performed following the **General Procedure G** with 1,4-dioxa-8-azaspiro[4.5]decan-8-yl benzoate **1g** (210.5 mg, 0.8 mmol) and diphenylphosphine oxide **2a** (80.8 mg, 0.4 mmol). The crude product was separated by flash chromatography on deactivated silica gel (hexane:ethyl acetate = 2:1 to ethyl acetate:methanol = 20:1) to give the product **3ga** (111.2 mg, 81% yield) as a light yellow oil. **R*f*** = 0.39 (ethyl acetate:methanol = 20:1). **^{1}H NMR** (400 MHz, Chloroform-*d*) δ 7.87 – 7.72 (m, 4H), 7.50 – 7.39 (m, 6H), 3.88 – 3.80 (m, 4H), 3.75 – 3.70 (m, 1H), 3.11 – 3.06 (m, 1H), 2.86 – 2.78 (m, 1H), 2.24 (s, 1H), 1.76 – 1.50 (m, 4H) ppm; **^{13}C{^{1}H} NMR** (100 MHz, Chloroform-*d*) δ 132.11 (d, *J* = 3.0 Hz), 132.08 (d, *J* = 2.8 Hz), 131.8 (d, *J* = 8.9 Hz), 131.4 (d, *J* = 8.8 Hz), 130.9 (d. *J* = 57.9 Hz), 130.0 (d. *J* = 58.7 Hz), 128.7 (d, *J* = 9.4 Hz), 128.6 (d, *J* = 9.6 Hz), 107.2 (d, *J* = 14.0 Hz), 64.3 (d, *J* = 4.3 Hz), 64.1, 54.0 (d, *J* = 84.7 Hz), 45.0 (d, *J* = 15.4 Hz), 36.0, 34.7 ppm; **^{31}P NMR** (162 MHz, Chloroform-*d*) δ 31.36 ppm; **HRMS** calc'd for $C_{19}H_{23}NO_3P^+$ 344.1410, found 344.1406 $[M+H]^+$.

((3a*R*,7a*S*)-Octahydro-1*H*-isoindol-1-yl)diphenylphosphine oxide (3ha):

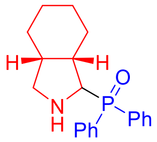

The reaction was performed following the **General Procedure G** with (3a*R*,7a*S*)-octahydro-2*H*-isoindol-2-yl benzoate **1h** (196.1 mg, 0.8 mmol) and diphenylphosphine oxide **2a** (80.8 mg, 0.4 mmol). The crude product was separated by flash chromatography on deactivated silica gel (hexane:ethyl acetate = 2:1 to ethyl acetate:methanol = 20:1) to give the product **3ha** (75.4 mg, 58% yield) as a colorless oil. **R*f*** = 0.38 (ethyl acetate:methanol = 20:1). **^{1}H NMR** (400 MHz, Chloroform-*d*) δ 8.01 – 7.96 (m, 2H), 7.87 – 7.82 (m, 2H), 7.51 – 7.40 (m, 6H), 3.79 (dd, *J* = 7.2, 4.4 Hz, 1H), 2.81 – 2.72 (m, 2H), 2.47 – 2. 34 (m, 2H), 2.04 – 2.96 (m, 1H), 1.56 – 1.43 (m, 4H), 1.39 – 1.23 (m, 4H) ppm; **^{13}C{^{1}H} NMR** (100 MHz, Chloroform-*d*) δ 133.3 (d, *J* = 92.9 Hz), 132.2 (d, *J* = 8.0 Hz), 131.7 (d, *J* = 2.6 Hz), 131.6 (d, *J* = 2.7 Hz), 131.5 (d, *J* = 94.3 Hz), 131.2 (d, *J* = 8.5 Hz), 128.5 (d, *J* = 9.1 Hz), 128.4 (d, *J* = 8.8 Hz), 59.6

(d, *J* = 86.8 Hz), 51.6 (d, *J* = 4.7 Hz), 39.2 (d, *J* = 2.6 Hz), 39.1 (d, *J* = 2.2 Hz), 27.4 (d, *J* = 5.3 Hz), 26.2, 23.5, 22.7 ppm; **^{31}P NMR** (162 MHz, Chloroform-*d*) δ 30.66 ppm; **HRMS** calc'd for $C_{20}H_{25}NOP^+$ 326.1668, found 326.1672 $[M+H]^+$.

((Methylamino)methyl)diphenylphosphine oxide (3ia):

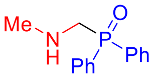

The reaction was performed following the **General Procedure G** with *O*-benzoyl-*N*,*N*-dimethylhydroxylamine **1i** (198.1 mg, 1.2 mmol), $NaHCO_3$ (100.8 mg, 1.2 mmol) and diphenylphosphine oxide **2a** (80.8 mg, 0.4 mmol). The crude product was separated by flash chromatography on deactivated silica gel (hexane:ethyl acetate = 1:1 to ethyl acetate:methanol = 5:1) to give the product **3ia** (51.2 mg, 52% yield) as a colorless oil. **R_f** = 0.37 (ethyl acetate:methanol = 5:1). **^{1}H NMR** (400 MHz, Chloroform-*d*) δ 7.81 – 7.76 (m, 4H), 7.55 – 7.44 (m, 6H), 3.45 (d, *J* = 7.6 Hz, 2H), 2.50 (s, 3H), 1.81 (s, 1H) ppm; **^{13}C{^{1}H} NMR** (100 MHz, Chloroform-*d*) δ 132.1 (d, *J* = 2.7 Hz), 132.0 (d, *J* = 97.2 Hz), 131.2 (d, *J* = 9.1 Hz), 128.8 (d, *J* = 11.5 Hz), 51.6 (d, *J* = 79.7 Hz), 38.8 (d, *J* = 14.7 Hz) ppm; **^{31}P NMR** (162 MHz, Chloroform-*d*) δ 29.05 ppm; **HRMS** calc'd for $C_{14}H_{17}NOP^+$ 246.1042, found 246.1047 $[M+H]^+$.

(((methyl-*d*$_3$)amino)methyl-*d*$_2$)diphenylphosphine oxide (*d*-3ia):

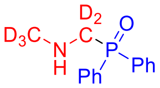

The reaction was performed following the **General Procedure G** with ***O*-benzoyl-*N*,*N*-bis(methyl-*d*$_3$)hydroxylamine** *d*-**1i** (205.3 mg, 1.2 mmol), $NaHCO_3$ (100.8 mg, 1.2 mmol) and diphenylphosphine oxide **2a** (80.8 mg, 0.4 mmol). The crude product was separated by flash chromatography on deactivated silica gel (hexane:ethyl acetate = 1:1 to ethyl acetate:methanol = 5:1) to give the product **3ia** (50.2 mg, 50% yield) as a colorless oil. **R_f** = 0.37 (ethyl acetate:methanol = 5:1). **^{1}H NMR** (400 MHz, Chloroform-*d*) δ 7.80 – 7.75 (m, 4H), 7.55 – 7.43 (m, 6H), 1.55 (s, 1H) ppm; **^{13}C{^{1}H} NMR** (100 MHz, Chloroform-*d*) δ 132.1 (d, *J* = 2.9 Hz), 132.0 (d, *J* = 96.9 Hz), 131.2 (d, *J* = 9.3 Hz), 128.8 (d, *J* = 11.5 Hz) ppm, two resonances were not observed due to overlapping peaks; **^{31}P NMR** (162 MHz, Chloroform-*d*) δ 29.01 ppm; **HRMS** calc'd for $C_{14}H_{12}D_5NOP^+$ 251.1356, found 251.1354 $[M+H]^+$.

(1-(Ethylamino)ethyl)diphenylphosphine oxide (3ja):

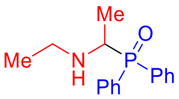

The reaction was performed following the **General Procedure G** with *O*-benzoyl-*N*,*N*-diethylhydroxylamine **1j** (231.7 mg, 1.2 mmol), $NaHCO_3$ (100.8 mg, 1.2 mmol) and diphenylphosphine oxide **2a** (80.8 mg, 0.4 mmol). The crude product was separated by flash chromatography on deactivated silica gel (hexane:ethyl acetate = 2:1 to ethyl acetate:methanol = 20:1) to give the product **3ja** (58.1 mg, 53% yield) as a light yellow oil. **R_f** = 0.40 (ethyl acetate:methanol = 20:1). **^{1}H NMR** (400 MHz, Chloroform-*d*) δ 7.91 – 7.82 (m, 4H), 7.51 – 7.40 (m, 6H), 3.55 – 3.47 (m, 1H), 2.81 – 2.73 (m, 1H), 2.51 – 2.42 (m, 1H), 1.79 (s, 1H), 1.29 (dd, *J* = 16.0, 7.2 Hz, 3H), 0.98 (t, *J* = 7.2 Hz, 3H) ppm; **^{13}C{^{1}H} NMR** (100 MHz, Chloroform-*d*) δ 132.1 (d, *J* = 90.6 Hz), 131.9 (d, *J* = 8.4 Hz), 131.78 (d, *J* = 2.3 Hz),

131.75 (d, *J* = 2.6 Hz), 131.5 (d, *J* = 8.6 Hz), 131.2 (d, *J* = 92.0 Hz), 128.5 (d, *J* = 11.1 Hz), 128.4 (d, *J* = 11.1 Hz), 52.6 (d, *J* = 83.7 Hz), 42.4 (d, *J* = 11.8 Hz), 15.4, 14.2 (d, *J* = 1.9 Hz) ppm; **^{31}P NMR** (162 MHz, Chloroform-*d*) δ 32.19 ppm; **HRMS** calc'd for $C_{16}H_{21}NOP^+$ 274.1355, found 274.1353 $[M+H]^+$.

((Isopropylamino)methyl)diphenylphosphine oxide (3ka):

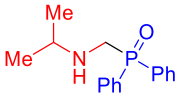

The reaction was performed following the **General Procedure G** with *O*-benzoyl-*N*-isopropyl-*N*-methylhydroxylamine **1k** (154.5 mg, 0.8 mmol) and diphenylphosphine oxide **2a** (80.8 mg, 0.4 mmol). The crude product was separated by flash chromatography on deactivated silica gel (hexane:ethyl acetate = 2:1 to ethyl acetate:methanol = 20:1) to give the product **3ka** (65.6 mg, 60% yield) as a white solid. **Mp**: 91 – 93 ºC. **R*f*** = 0.39 (ethyl acetate:methanol = 20:1). **^{1}H NMR** (400 MHz, Chloroform-*d*) δ 7.80 – 7.75 (m, 4H), 7.52 – 7.41 (m, 6H), 3.43 (d, *J* = 8.8 Hz, 2H), 2.83 – 2.77 (m, 1H), 1.74 (s, 1H), 1.02 (d, *J* = 7.6 Hz, 6H) ppm; **^{13}C{^{1}H} NMR** (100 MHz, Chloroform-*d*) δ 132.1 (d, *J* = 97.5 Hz), 132.0 (d, *J* = 2.7 Hz), 131.3 (d, *J* = 9.1 Hz), 128.6 (d, *J* = 11.5 Hz), 50.6 (d, *J* = 13.6 Hz), 47.0 (d, *J* = 81.1 Hz), 22.5 ppm; **^{31}P NMR** (162 MHz, Chloroform-*d*) δ 29.72 ppm; **HRMS** calc'd for $C_{16}H_{21}NOP^+$ 274.1355, found 274.1358 $[M+H]^+$.

(1-(Butylamino)butyl)diphenylphosphine oxide (3la):

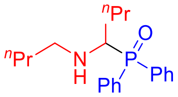

The reaction was performed following the **General Procedure G** with *O*-benzoyl-*N*,*N*-dibutylhydroxylamine **1l** (299.0 mg, 1.2 mmol), $NaHCO_3$ (100.8 mg, 1.2 mmol) and diphenylphosphine oxide **2a** (80.8 mg, 0.4 mmol). The crude product was separated by flash chromatography on deactivated silica gel (hexane:ethyl acetate = 3:1 to hexane:ethyl acetate = 1:1) to give the product **3la** (56.6 mg, 43% yield) as a colorless oil. **R*f*** = 0.41 (hexane:ethyl acetate = 1:1). **^{1}H NMR** (400 MHz, Chloroform-*d*) δ 7.98 – 7.93 (m, 2H), 7.90 – 7.85 (m, 2H), 7.52 – 7.42 (m, 6H), 3.33 – 3.29 (m, 1H), 2.63 – 2.57 (m, 1H), 2.31 – 2.25 (m, 1H), 1.88 – 1.75 (m, 1H), 1.61 – 1.30 (m, 4H), 1.29 – 1.22 (m, 2H), 1.21 – 1.11 (m, 2H), 0.86 (t, *J* = 7.2 Hz, 3H), 0.78 (t, *J* = 7.6 Hz, 3H) ppm; **^{13}C{^{1}H} NMR** (100 MHz, Chloroform-*d*) δ 133.0 (d, *J* = 90.6 Hz), 131.99 (d, *J* = 8.2 Hz), 131.95 (d, *J* = 92.0 Hz), 131.7 (d, *J* = 2.4 Hz), 131.6 (d, *J* = 2.5 Hz), 131.5 (d, *J* = 8.5 Hz), 128.5 (d, *J* = 11.1 Hz), 128.4 (d, *J* = 10.9 Hz), 58.2 (d, *J* = 82.6 Hz) ,49.3 (d, *J* = 7.3 Hz), 32.7, 32.1 (d, *J* = 3.5 Hz), 20.3, 20.1 (d, *J* = 10.5 Hz), 14.1, 14.0 ppm; **^{31}P NMR** (162 MHz, Chloroform-*d*) δ 30.66 ppm; **HRMS** calc'd for $C_{20}H_{29}NOP^+$ 330.1981, found 330.1983 $[M+H]^+$.

((Cyclohexylamino)methyl)diphenylphosphine oxide (3ma):

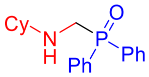

The reaction was performed following the **General Procedure G** with *O*-benzoyl-*N*-cyclohexyl-*N*-methylhydroxylamine **1m** (186.5 mg, 0.8 mmol) and diphenylphosphine oxide **2a** (80.8 mg, 0.4 mmol). The crude product was separated by flash chromatography on deactivated silica gel (hexane:ethyl acetate = 3:1 to ethyl acetate:methanol = 40:1) to give the product **3ma** (70.1 mg, 56% yield) as a white solid. **Mp**: 94 – 95 ºC. **R*f*** = 0.42 (ethyl acetate:methanol = 40:1). **^{1}H NMR** (400 MHz, Chloroform-*d*) δ 7.81

– 7.76 (m, 4H), 7.53 – 7.42 (m, 6H), 3.48 (d, *J* = 8.4 Hz, 2H), 2.47 – 2.40 (m, 1H), 1.84 – 1.80 (m, 3H), 1.70 – 1.64 (m, 2H), 1.56 – 1.52 (m, 1H), 1.24 – 1.03 (m, 5H) ppm; **^{13}C{^{1}H} NMR** (100 MHz, Chloroform-*d*) δ 132.2 (d, *J* = 97.2 Hz), 132.0 (d, *J* = 2.8 Hz), 131.3 (d, *J* = 9.2 Hz), 128.7 (d, *J* = 11.5 Hz), 58.3 (d, *J* = 13.2 Hz), 46.6 (d, *J* = 80.8 Hz), 33.0, 26.1, 24.8 ppm; **^{31}P NMR** (162 MHz, Chloroform-*d*) δ 29.84 ppm; **HRMS** calc'd for $C_{19}H_{25}NOP^+$ 314.1668, found 314.1665 [M+H]$^+$.

Diphenyl(((tetrahydro-2*H*-pyran-4-yl)amino)methyl)phosphine oxide (3na):

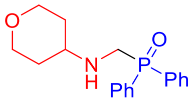

The reaction was performed following the **General Procedure G** with *O*-benzoyl-*N*-methyl-*N*-(tetrahydro-2*H*-pyran-4-yl)hydroxylamine **1n** (188.1 mg, 0.8 mmol) and diphenylphosphine oxide **2a** (80.8 mg, 0.4 mmol). The crude product was separated by flash chromatography on deactivated silica gel (hexane:ethyl acetate = 2:1 to ethyl acetate:methanol = 20:1) to give the product **3na** (74.4 mg, 59% yield) as a light yellow oil. **R*f*** = 0.28 (ethyl acetate:methanol = 20:1). **^{1}H NMR** (400 MHz, Chloroform-*d*) δ 7.79 – 7.74 (m, 4H), 7.53 – 7.42 (m, 6H), 3.90 – 3.86 (m, 2H), 3.46 (d, *J* = 8.4 Hz, 2H), 3.31 (td, *J* = 11.6, 2.4 Hz, 2H), 2.73 – 2.66 (m, 1H), 1.89 (s, 1H), 1.79 – 1.73 (m, 2H), 1.40 – 1.32 (m, 2H) ppm; **^{13}C{^{1}H} NMR** (100 MHz, Chloroform-*d*) δ 132.1 (d, *J* = 2.7 Hz), 131.9 (d, *J* = 97.8 Hz), 131.2 (d, *J* = 9.3 Hz), 128.7 (d, *J* = 11.7 Hz), 66.4, 55.2 (d, *J* = 13.3 Hz), 46.1 (d, *J* = 80.7 Hz), 33.1 ppm; **^{31}P NMR** (162 MHz, Chloroform-*d*) δ 29.74 ppm; **HRMS** calc'd for $C_{18}H_{23}NO_2P^+$ 316.1461, found 316.1466 [M+H]$^+$.

(((3-(10,11-Dihydro-5*H*-dibenzo[*a*,*d*][7]annulen-5-ylidene)propyl)amino)methyl)diphenyl-phosphine oxide (3oa):

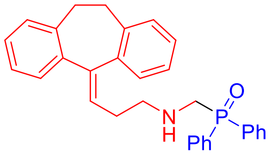

The reaction was performed following the **General Procedure G** with *O*-benzoyl-*N*-(3-(10,11-dihydro-5*H*-dibenzo[*a*,*d*][7]annulen-5-ylidene)-propyl)-N-methylhydroxylamine **1o** (459.8 mg, 1.2 mmol), $NaHCO_3$ (100.8 mg, 1.2 mmol) and diphenylphosphine oxide **2a** (80.8 mg, 0.4 mmol). The crude product was separated by flash chromatography on deactivated silica gel (hexane:ethyl acetate = 2:1 to hexane:ethyl acetate = 1:3) to give the product **3oa** (79.8 mg, 43% yield) as a colorless oil. **R*f*** = 0.26 (hexane:ethyl acetate = 1:3). **^{1}H NMR** (400 MHz, Chloroform-*d*) δ 7.80 – 7.45 (m, 4H), 7.53 – 7.41 (m, 6H), 7.23 – 7.01 (m, 8H), 5.78 (t, *J* = 7.2 Hz, 1H), 3.42 (d, *J* = 8.4 Hz, 2H), 3.33 – 3.23 (m, 2H), 2.93 – 2.70(m, 4H), 2.30 – 2.25 (m, 2H), 1.76 (s, 1H) ppm; **^{13}C{^{1}H} NMR** (100 MHz, Chloroform-*d*) δ 144.3, 141.2, 140.0, 139.4, 137.1, 132.1 (d, *J* = 2.7 Hz), 132.0 (d, *J* = 97.0 Hz), 131.3 (d, *J* = 9.1 Hz), 130.1, 129.0, 128.7 (d, *J* = 10.6 Hz), 128.6, 128.4, 128.1, 127.5, 127.2, 126.1, 125.8, 51.4 (d, *J* = 13.4 Hz), 49.3 (d, *J* = 80.3 Hz), 33.8, 32.1, 29.8 ppm; **^{31}P NMR** (162 MHz, Chloroform-*d*) δ 29.14 ppm; **HRMS** calc'd for $C_{31}H_{31}NOP^+$ 464.2138, found 464.2141 [M+H]$^+$.

Diphenyl(((3-phenyl-3-(4-(trifluoromethyl)phenoxy)propyl)amino)methyl)phosphine oxide (3pa):

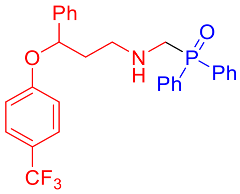

The reaction was performed following the **General Procedure G** with *O*-benzoyl-*N*-methyl-*N*-(3-phenyl-3-(4-(trifluoromethyl)phenoxy)propyl)-hydroxylamine **1p** (515.0 mg, 1.2 mmol), $NaHCO_3$ (100.8 mg, 1.2 mmol) and diphenylphosphine oxide **2a** (80.8 mg, 0.4 mmol). The crude product was separated by flash chromatography on deactivated silica gel (hexane:ethyl acetate = 2:1 to hexane:ethyl acetate = 1:3) to give the product **3pa** (91.8 mg, 45% yield) as a white solid. **Mp**: 129 – 130 ºC. **R*f*** = 0.21 (hexane:ethyl acetate = 1:2). **^{1}H NMR** (400 MHz, Chloroform-*d*) δ 7.78 – 7.73 (m, 4H), 7.55 – 7.37 (m, 8H), 7.31 – 7.27 (m, 2H), 7.25 – 7.22 (m, 3H), 6.80 (d, *J* = 8.8 Hz, 2H), 5.27 – 5.24 (m, 1H), 3.47 (d, *J* = 8.0 Hz, 2H), 2.93 – 2.79 (m, 2H), 2.20 – 2.11 (m, 1H), 2.04 – 1.93 (m, 2H) ppm; **^{13}C{^{1}H} NMR** (100 MHz, Chloroform-*d*) δ 160.6, 141.0, 132.4 (d, *J* = 13.9 Hz), 132.2 (d, *J* = 2.7 Hz), 132.1 (d, *J* = 2.8 Hz), 131.4 (d, *J* = 15.3 Hz), 131.3 (d, *J* = 4.9 Hz), 131.2 (d, *J* = 4.8 Hz), 128.9, 128.8 (d, *J* = 3.5 Hz), 128.7 (d, *J* = 3.6 Hz), 127.9, 127.2 (q, *J* = 267.2 Hz), 126.9 (q, *J* = 3.7 Hz), 125.9, 123.1 (q, *J* = 32.4 Hz), 115.8, 78.1, 49.4 (d, *J* = 80.1 Hz), 47.8 (d, *J* = 14.3 Hz), 38.6 ppm; **^{31}P NMR** (162 MHz, Chloroform-*d*) δ 29.37 ppm; **^{19}F NMR** (376 MHz, Chloroform-*d*) δ -61.50 ppm; **HRMS** calc'd for $C_{29}H_{28}F_3NO_2P^+$ 510.1804, found 510.1802 $[M+H]^+$.

6. Gram-scale Synthesis of 3aa

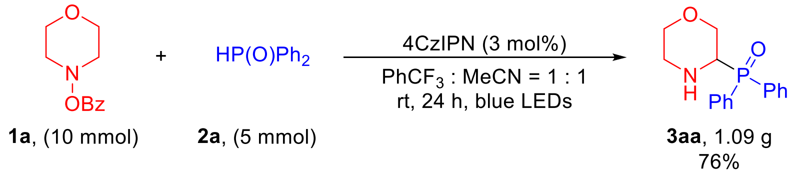

An oven-dried 100 mL reaction vial equipped with a stir bar in a glove box under a nitrogen atmosphere at room temperature was charged with 4CzIPN (118.2 mg, 0.15 mmol, 3 mol%), morpholino benzoate (**1a**, 2.07g, 10.0 mmol, 2.0 equiv.) and diphenylphosphine oxide (**2a**, 1.01g, 5.0 mmol, 1.0 equiv.). Next, MeCN : $PhCF_3$ = 1 : 1 (50 mL) was sequentially added via syringe and the mixture was stirred and until all the solids dissolved. The vial was capped, removed from the glove box, and stirred for 24 h with blue LEDs irradiation. The temperature was maintained at room temperature via cooling with a fan. After the reaction period, the lights were turned off, the reaction mixture was transferred to a round bottom flask and were concentrated in vacuo. The resulting mixed product was dissolved by adding 40 ml of ethyl acetate and washed with $NaHCO_3$ (30 mL X 2). the aqueous phase was collected and then extracted with ethyl acetate (50 mL X 2), all organic phases were collected and dried with Na_2SO_4. and the combined organic solutions were concentrated in *vacuo*. The crude material was loaded onto a silica gel column and purified by flash chromatography to give the product **3aa** (1.09 g, 76%) as a light yellow oil.

7. Synthesis of an antitumor agent (4ba).

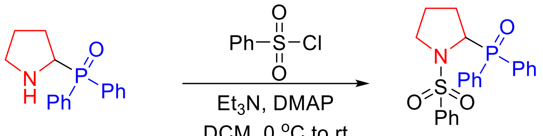

3ba **4ba**, 72 %

The reaction was performed following the literature procedures[12]

3ba (0.2 mmol, 1.0 equiv.) was dissolved in 2 mL of anhydrous CH_2Cl_2, and the solution was taken into a 10 mL round bottom flask in the ice-bath. Then, *p*-toluenesulfonyl chloride (0.3 mmol, 1.5 equiv.), triethylamine (0.4 mmol, 2 equiv.), and 4-dimethylaminopyridine (DMAP, 0.04 mmol, 20 mol %) were added, and the icebath was removed. The reaction mixture was allowed to stir at room temperature for 16 hours followed by aqueous workup (saturated NH_4Cl solution). The organic layer was separated, and the aqueous layer was extracted by CH_2Cl_2 (5 mL) two times. The combined organic layer was concentrated in vacuo, and the crude material was purified by chromatography (silica gel) to give the product **4ba** (59.2 mg, 72%)

Diphenyl(1-(phenylsulfonyl)pyrrolidin-2-yl)phosphine oxide (4ba)

^{1}H NMR (400 MHz, Chloroform-*d*) δ 7.98 – 7.89 (m, 4H), 7.59 – 7.46 (m, 9H), 7.42 – 7.37 (m, 2H), 4.99 – 4.96 (m, 1H), 3.53 – 3.46 (m, 1H), 3.32 – 3.25 (m, 1H), 2.28 – 2.17 (m, 1H), 2.00 – 1.88 (m, 1H), 1.86 – 1.76 (m, 1H), 1.59 – 1.49 (m, 1H) ppm; **^{13}C{^{1}H} NMR** (100 MHz, Chloroform-*d*) δ 138.5, 133.0, 132.3 (d, J = 8.7 Hz), 132.21, 132.19, 131.49 (d, J = 93.6 Hz), 131.43 (d, J = 8.5 Hz), 130.4 (d, J = 99.0 Hz), 129.2, 128.9 (d, J = 11.3 Hz), 128.5 (d, J = 11.7 Hz), 127.6, 58.9 (d, J = 85.3 Hz), 50.0, 26.2, 25.0 ppm; **^{31}P NMR** (162 MHz, Chloroform-*d*) δ 31.98 ppm; The other datas for these compounds match the literature data.[13]

8. Mechanistic Studies

a) Radical trapping with TEMPO experiment

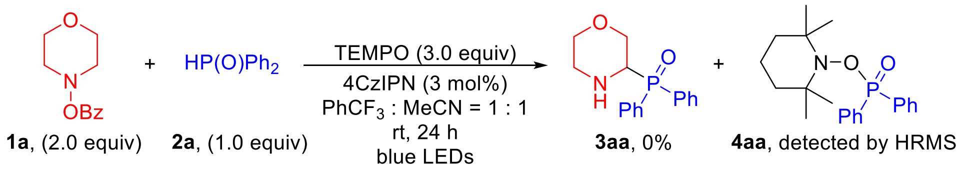

1a, (2.0 equiv) **2a**, (1.0 equiv) **3aa**, 0% **4aa**, detected by HRMS

The reaction was performed following **General Procedure G** with 4CzIPN (9.5 mg, 0.012 mmol, 3 mol%), morpholino benzoate (**1a**, 165.7 mg, 0.8 mmol, 2.0 equiv.), diphenylphosphine oxide (**2a**, 80.8 mg, 0.4 mmol, 1.0 equiv.), 2,2,6,6-tetramethylpiperidine-1-oxyl (187.5 mg, 1.2 mmol) in 4 mL MeCN : $PhCF_3$ = 1 : 1 and irradiation under blue LEDs with argon protection at rt for 24 h. The corresponding HRMS spectrums were given as below.

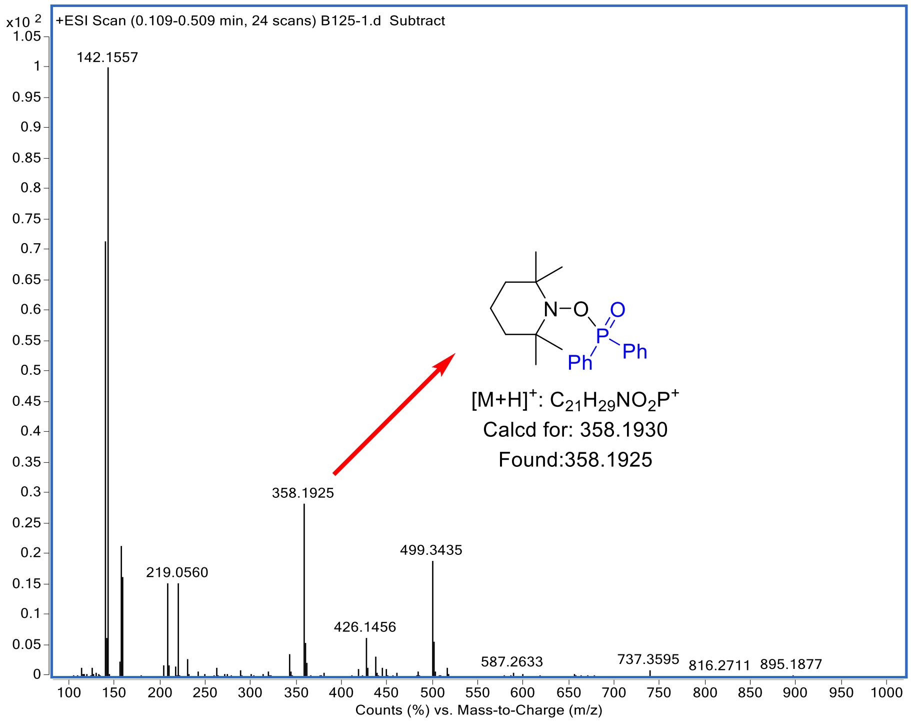

Figure S2. HRMS spectrum of standard conditions adduct with TEMPO

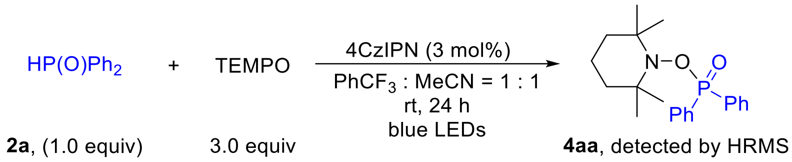

The reaction was performed following **General Procedure G** with 4CzIPN (9.5 mg, 0.012 mmol, 3 mol%), diphenylphosphine oxide (**2a**, 80.8 mg, 0.4 mmol, 1.0 equiv.), 2,2,6,6-tetramethylpiperidine-1-oxyl (187.5 mg, 1.2 mmol) in 4 mL MeCN : $PhCF_3$ = 1 : 1 and irradiation under blue LEDs with argon protection at rt for 24 h. The corresponding HRMS spectrums were given as below.

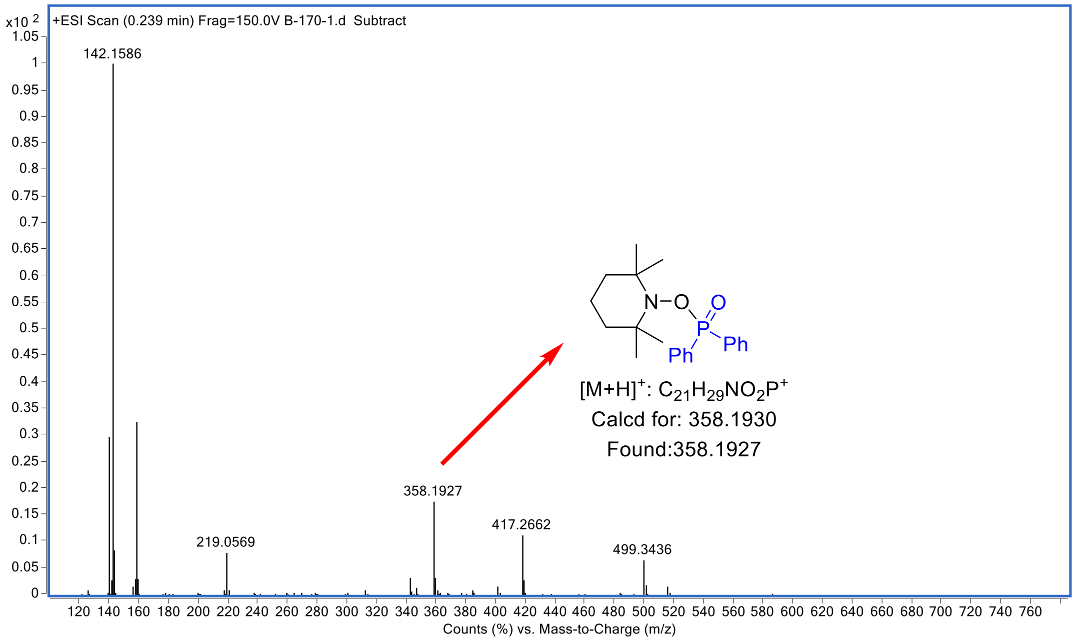

Figure S3. HRMS spectrum of **2a** adduct TEMPO

b) Radical trapping with 1,1-diphenylethylene experiment

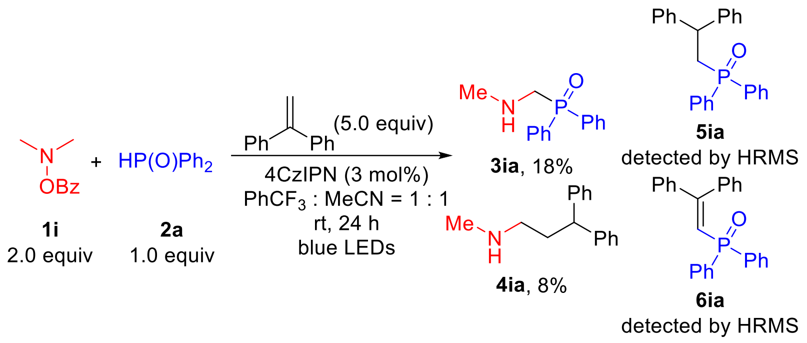

The reaction was performed following **General Procedure G** with 4CzIPN (23.8 mg, 0.03 mmol, 3 mol%), *O*-benzoyl-*N*,*N*-dimethyl-hydroxylamine (**1i**, 330.3 mg, 2.0 mmol, 2.0 equiv.), diphenylphosphine oxide (**2a**, 202.0 mg, 1.0 mmol, 1.0 equiv.), 1,1-diphenylethylene (900.5 mg, 5.0 mmol, 5.0 equiv.) in 10 mL MeCN : $PhCF_3$ = 1 : 1 and irradiation under blue LEDs with argon protection at rt for 24 h. The corresponding HRMS spectrums were given as below.

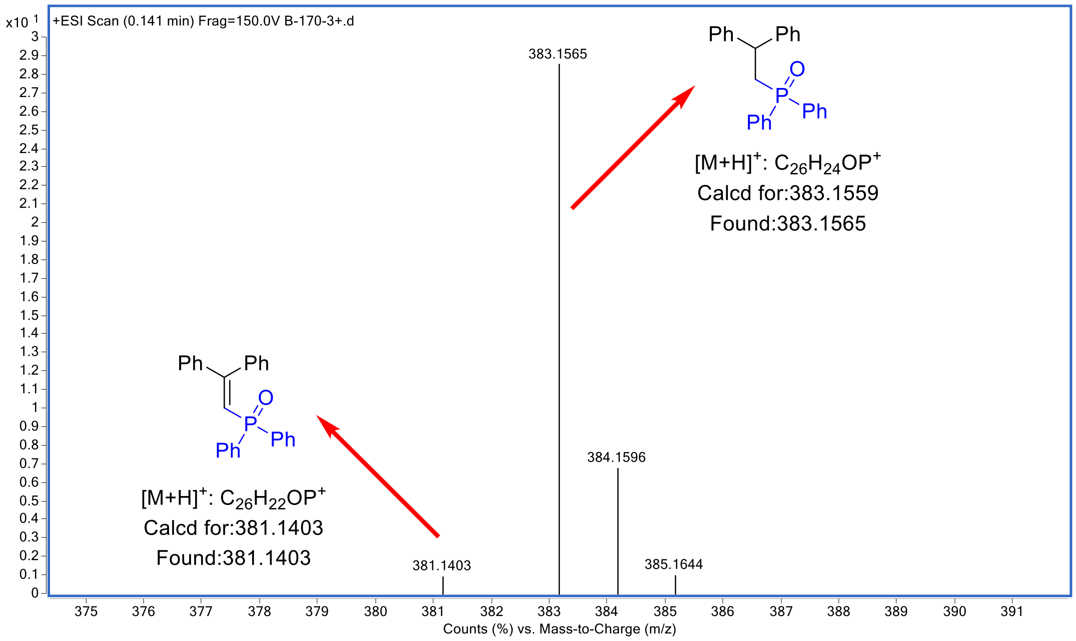

Figure S4. HRMS spectrum of standard conditions adduct with 1,1-diphenylethylene

N-Methyl-3,3-diphenylpropan-1-amine (4ia):

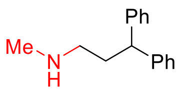

The crude product was separated by flash chromatography on deactivated silica gel (hexane:ethyl acetate = 1:2 to ethyl acetate: methanol = 2:1) to give the product **4ia** (18.0 mg, 8% yield) as yellow oil. $\mathbf{R}_f$ = 0.35 (ethyl acetate: methanol = 2:1). **^{1}H NMR** (400 MHz, Chloroform-*d*) δ 7.31 – 7.25 (m, 8H), 7.21 – 7.16 (m, 2H), 4.03 (t, *J* = 8.0 Hz, 1H), 2.56 (t, *J* = 7.2 Hz, 2H), 2.40 (s, 3H), 2.27 (q, *J* = 15.2, 7.6 Hz, 2H), 2.10 (s, 1H). ppm; **^{13}C{^{1}H} NMR** (100 MHz, Chloroform-*d*) δ 144.8, 128.6, 127.9, 126.3, 50.5,

49.1, 36.3, 35.5 ppm; **HRMS** calc'd for $C_{16}H_{20}N^{+}$ 226.1590, found 226.1587 $[M+H]^{+}$.

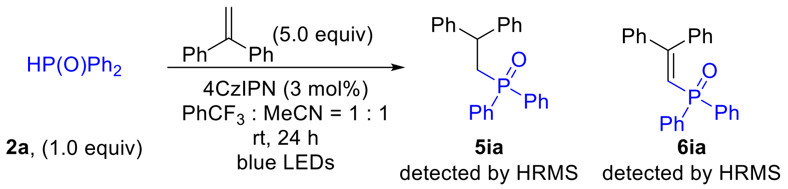

The reaction was performed following **General Procedure G** with 4CzIPN (9.5 mg, 0.012 mmol, 3 mol%), diphenylphosphine oxide (**2a**, 80.8 mg, 0.4 mmol, 1.0 equiv.), 1,1-diphenylethylene (360.2 mg, 2.0 mmol, 2.0 equiv.) in 4 mL MeCN : $PhCF_3$ = 1 : 1 and irradiation under blue LEDs with argon protection at rt for 24 h. The corresponding HRMS spectrums were given as below.

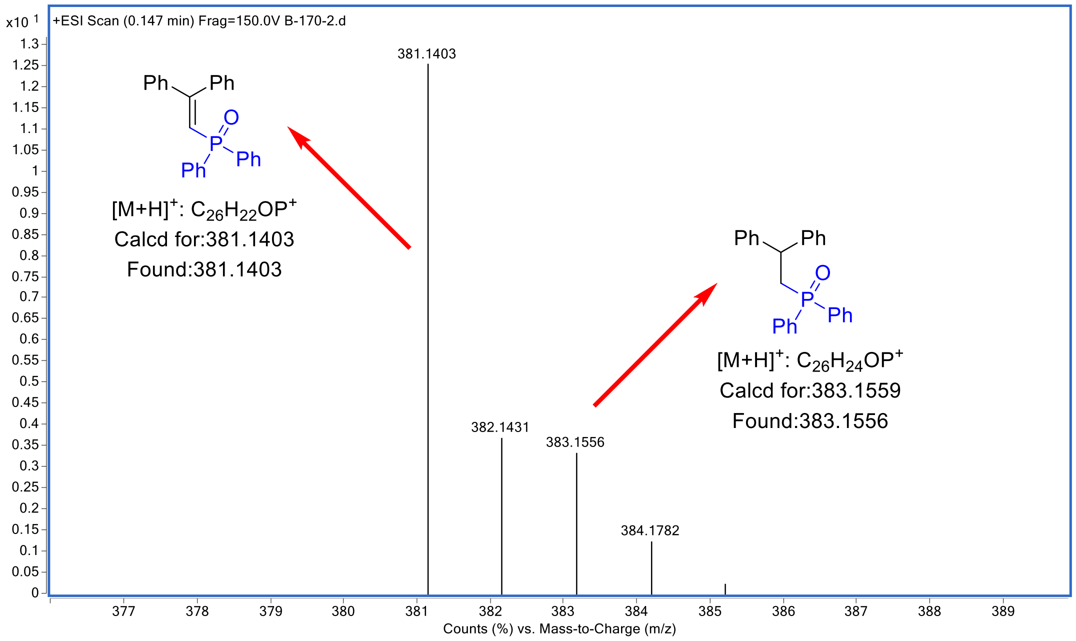

Figure S5. HRMS spectrum of **2a** adduct with 1,1-diphenylethylene

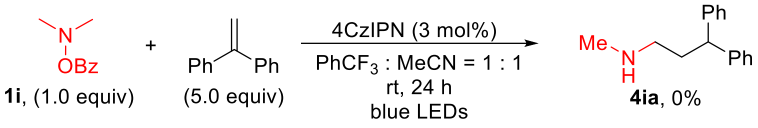

The reaction was performed following **General Procedure G** with 4CzIPN (9.5 mg, 0.012 mmol, 3 mol%), *O*-benzoyl-*N*,*N*-dimethyl-hydroxylamine (**1i**, 66.0 mg, 0.4 mmol, 1.0 equiv.), 1,1-diphenylethylene (360.2 mg, 2.0 mmol, 5.0 equiv.) in 4 mL MeCN : $PhCF_3$ = 1 : 1 and irradiation under blue LEDs with argon protection at rt for 24 h. We did not generate the trapping product from α-amino radical **4ai**.

c) Radical trapping with benzyl acrylate experiment

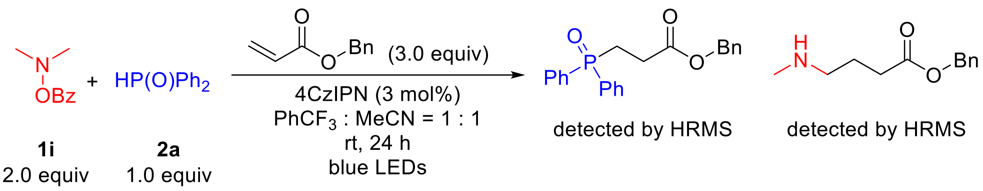

The reaction was performed following **General Procedure G** with 4CzIPN (9.5 mg, 0.012 mmol, 3 mol%), *O*-benzoyl-*N*,*N*-dimethyl-hydroxylamine (**1i**, 132.0 mg, 0.8 mmol, 2.0 equiv.), diphenylphosphine oxide (**2a**, 80.4 mg, 0.4 mmol, 1.0 equiv.), benzyl acrylate (194.6 mg, 1.2 mmol, 3.0 equiv.) in 4 mL MeCN : $PhCF_3$ = 1 : 1 and irradiation under blue LEDs with argon protection at rt for 24 h. The corresponding HRMS spectrums were given as below.

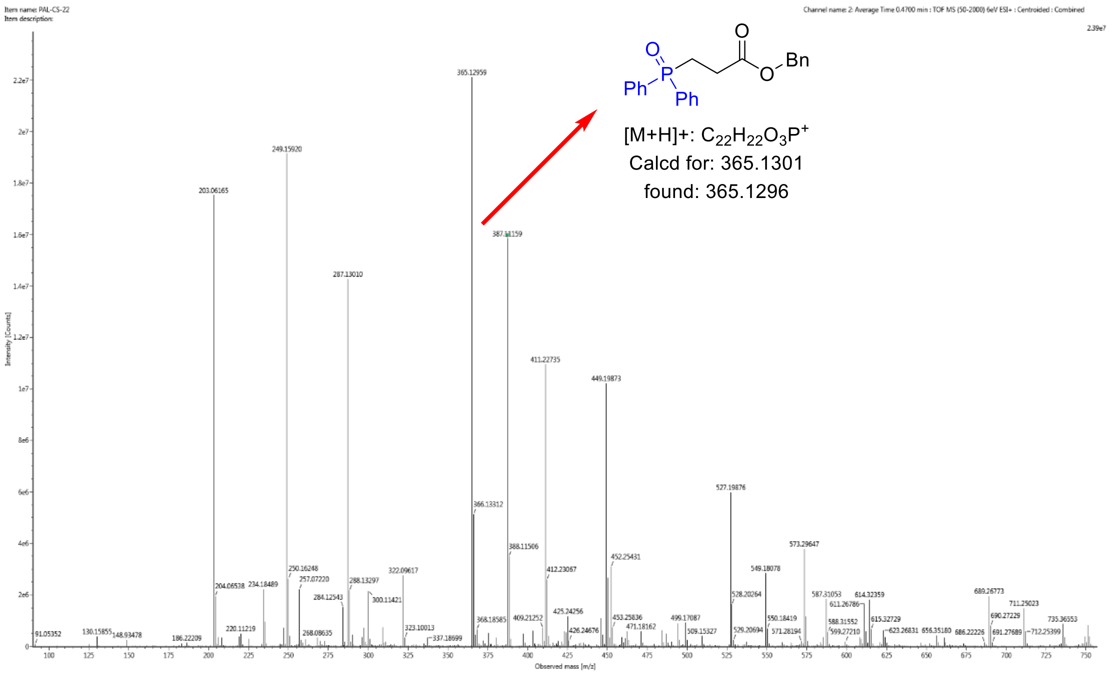

Figure S6a. HRMS spectrum of standard conditions adduct with benzyl acrylate

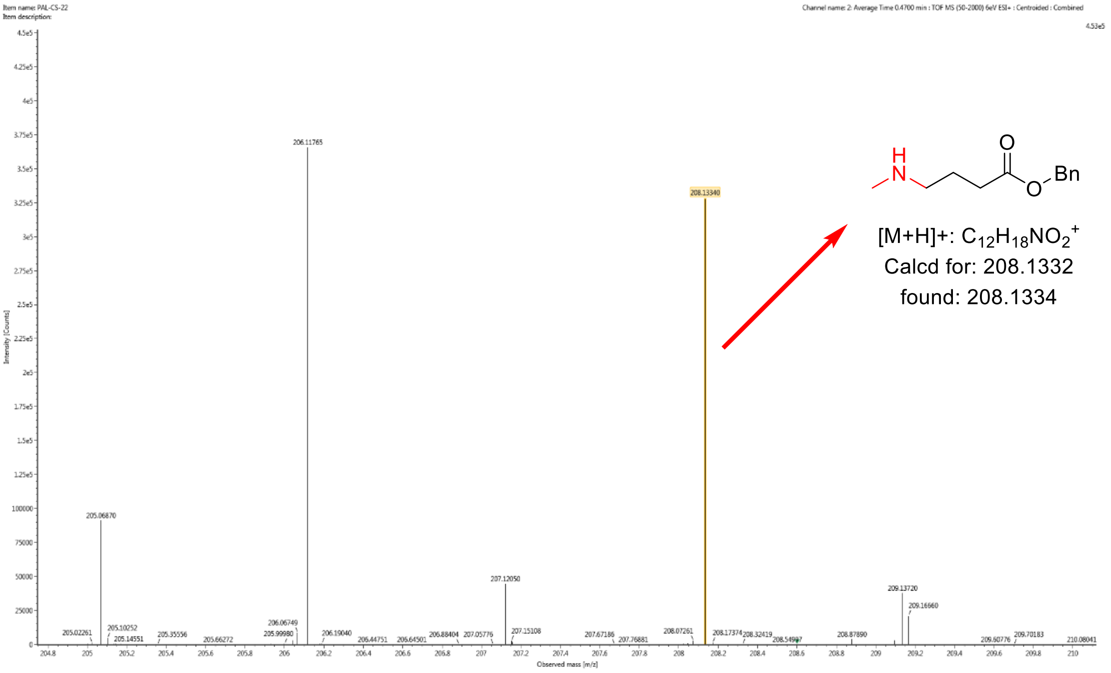

Figure S6b. HRMS spectrum of standard conditions adduct with benzyl acrylate

d) Capturing the iminium ion with dibutylamine

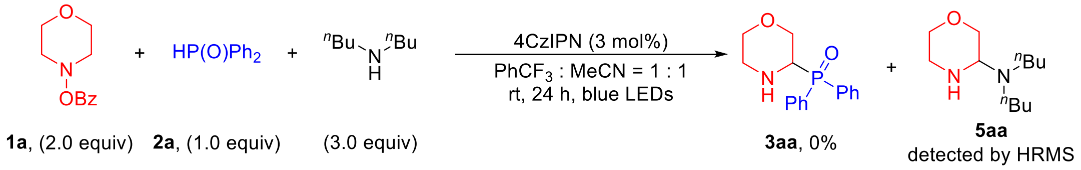

The reaction was performed following **General Procedure G** with 4CzIPN (9.5 mg, 0.012 mmol, 3 mol%), morpholino benzoate (**1a**, 165.7 mg, 0.8 mmol, 2.0 equiv.), diphenylphosphine oxide (**2a**, 80.8 mg, 0.4 mmol, 1.0 equiv.), dibutylamine (155.1 mg, 1.2 mmol) in 4 mL MeCN : $PhCF_3$ = 1 : 1 and irradiation under blue LEDs with argon protection at rt for 24 h. The corresponding HRMS spectrums were given as below.

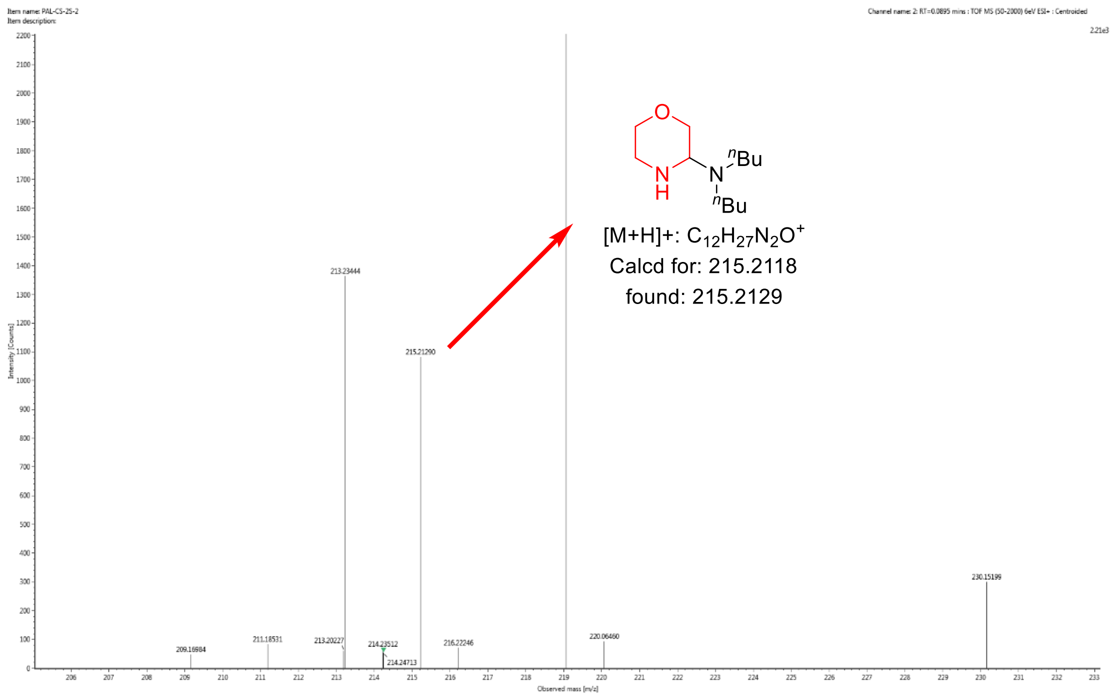

Figure S6c. HRMS spectrum of standard conditions adduct with dibutylamine

e) Stern-Volmer fluorescence quenching experiments

The stock solutions of 4CzIPN (3.2 mg in 10.0 mL MeCN : $PhCF_3$ = 1 : 1, 4.0×10^{-4} M), morpholino benzoate **1a** (207.1 mg in 10.0 mL MeCN : $PhCF_3$ = 1 : 1, 10^{-1} M), diphenylphosphine oxide **2a** (202.1 mg in 10.0 mL MeCN : $PhCF_3$ = 1 : 1, 10^{-1} M) were prepared in a glovebox. Then the sample solutions with different concentration gradients were prepared by mixing corresponding stock solutions in 5 mL brown vials and diluted to 1.0 mL with MeCN : $PhCF_3$ = 1 : 1. Subsequently the sample solution was placed in a cuvette with screw cap for further measurements on a fluorescence spectrometer. Emission intensities were recorded using Hitchi F-4700 fluorescence spectrometer for all experiments. All sample solutions were excited at 400 nm (slit 10.0 nm) and the emission intensity was collected at 420 nm-720 nm.

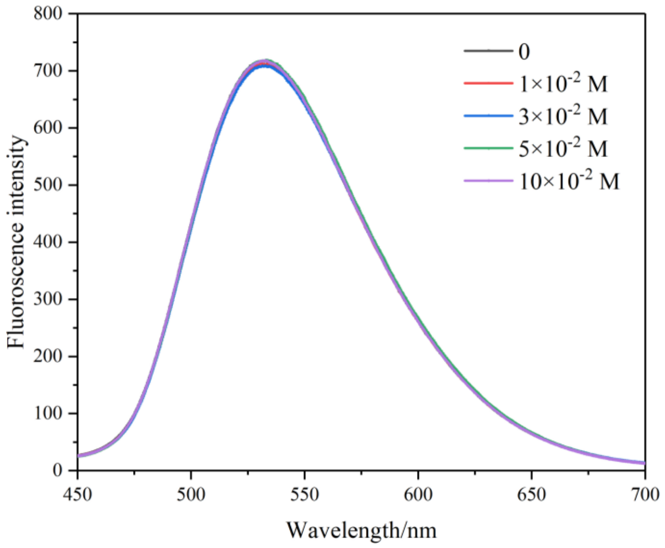

Figure S7. Emission spectra at different ratios of 4CzIPN to **1a**. c[4CzIPN]= 5 X 10^{-5} M.

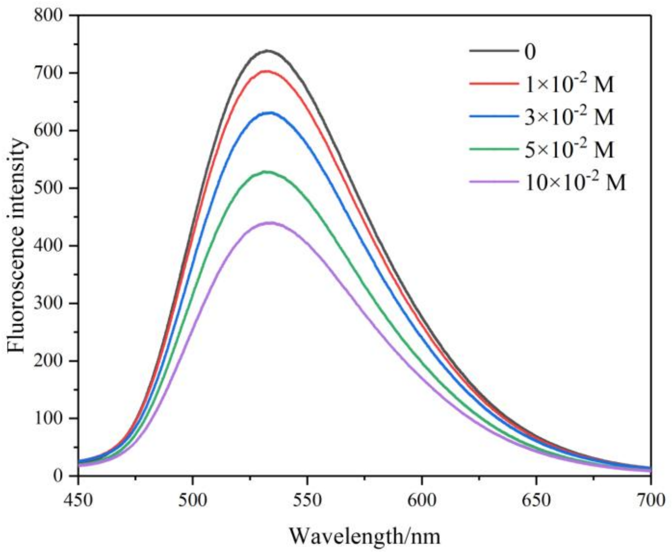

Figure S8. Emission spectra at different ratios of 4CzIPN to **2a**. c[4CzIPN]= 5 X 10^{-5} M.

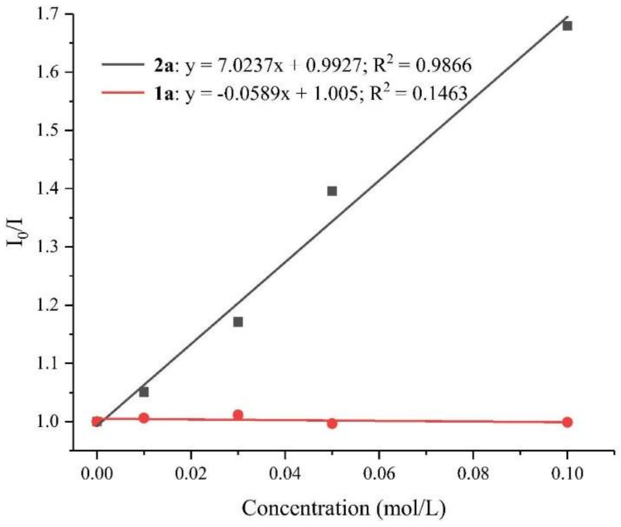

Figure S9. Stern-Volmer quenching plot in relative to **1a** or **2a**

As shown in Figure S7, the addition of morpholino benzoate **1a** to the solution of 4CzIPN didn't affect the emission spectra of the photocatalyst under the same excitation conditions. On the contrary, emission intensity of 4CzIPN is obviously diminished in the presence of diphenylphosphine oxide **2a** (Figure S8). The Stern-Volmer quenching plot is linear for the diphenylphosphine oxide **2a**.

f) Cross-Over experiments

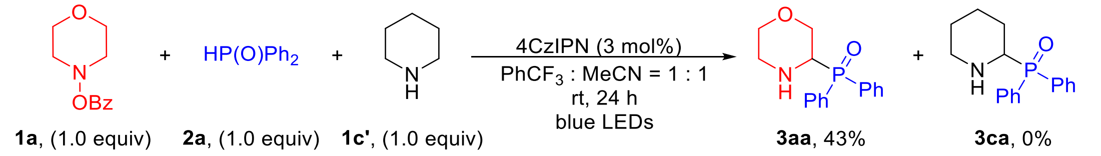

The reaction was performed following **General Procedure G** with 4CzIPN (9.5 mg, 0.012 mmol, 3 mol%), morpholino benzoate (**1a**, 82.8 mg, 0.4 mmol, 1.0 equiv.), diphenylphosphine oxide (**2a**, 80.8 mg, 0.4 mmol, 1.0 equiv.), piperidine (**1c'**, 34.0 mg, 0.4 mmol, 1.0 equiv.) in 4 mL MeCN : $PhCF_3$ = 1 : 1 and irradiation under blue LEDs with argon protection at rt for 24 h and afforded **3aa** in 43% yield and no occurrence of **3ca**.

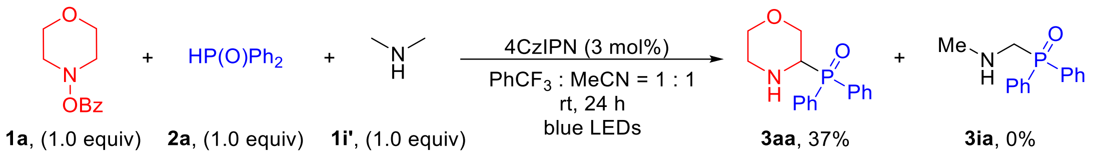

The reaction was performed following **General Procedure G** with 4CzIPN (9.5 mg, 0.012 mmol, 3 mol%), morpholino benzoate (**1a**, 82.8 mg, 0.4 mmol, 1.0 equiv.), diphenylphosphine oxide (**2a**, 80.8 mg, 0.4 mmol, 1.0 equiv.), dimethylamine (**1i'**, 18.0 mg, 0.4 mmol, 1.0 equiv.) in 4 mL MeCN : $PhCF_3$ = 1 : 1 and irradiation under blue LEDs with argon protection at rt for 24 h and afforded **3aa** in 37% yield and no occurrence of **3ia**.

g) Parallel KIE experiments

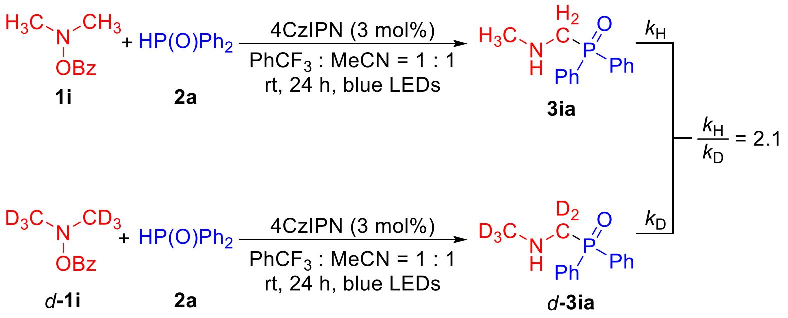

The reaction was performed following **General Procedure G** with 4CzIPN (2.4 mg, 0.003 mmol, 3 mol%), morpholino benzoate (**1i**, 33.0 mg, 0.2 mmol, 2.0 equiv.) or *d*-morpholino benzoate (*d*-**1i**, 34.2 mg, 0.2 mmol, 2.0 equiv.), diphenylphosphine oxide (**2a**, 20.2 mg, 0.1 mmol, 1.0 equiv.) in 1 mL MeCN : $PhCF_3$ = 1 : 1 and irradiation under blue LEDs with argon protection at rt. After the reaction was completed, solvent was removed under vacuum, the crude mixture was analyzed by ^{31}P-NMR with PPh_3 as an internal standard.

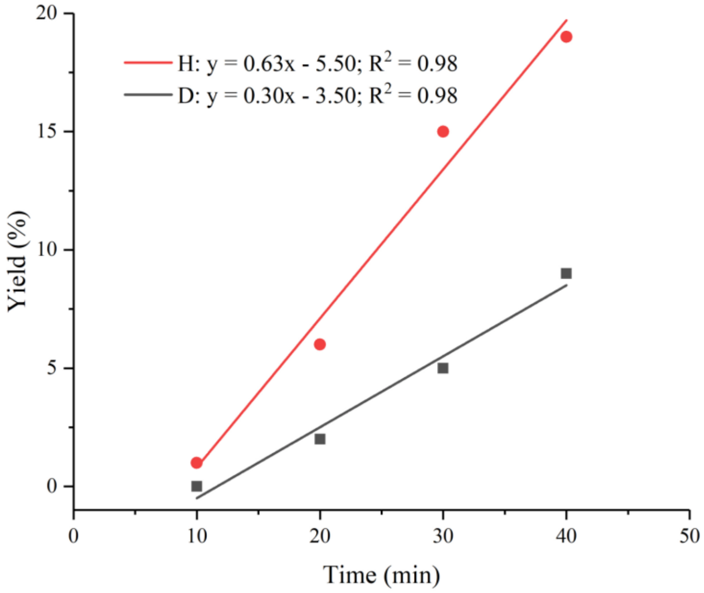

Figure S10. Reaction time-course data for substrates **1i** and *d*-**1i**

h) Cyclic Voltammetry Experiments

Tetrabutylammonium hexafluorophosphate (1.0 mmol, 387.4 mg) and morpholino benzoate **1a** (0.10 mmol, 20.7 mg) were dissolved in MeCN : $PhCF_3$ = 1 : 1 (10 mL) and the solution was vigorously bubbled with N_2 for 5 minutes prior to the measurement. The oxidation potential was measured using a glassy carbon working electrode, a platinum wire counter electrode, and a saturated calomel electrode (SCE) at 0.1 V/s scan rate.

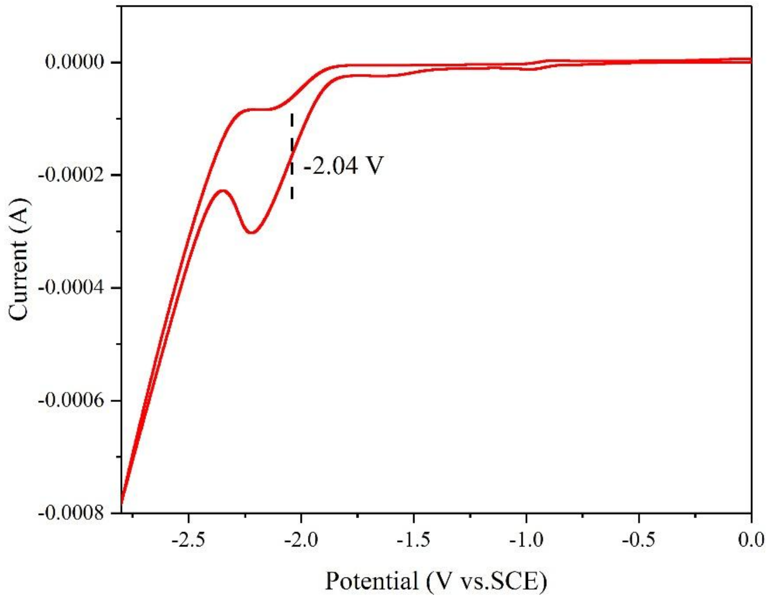

Figure S11. Cyclic voltammogram of morpholino benzoate **1a** in MeCN : $PhCF_3$ = 1 : 1

i) Effects of different leaving groups on the reaction

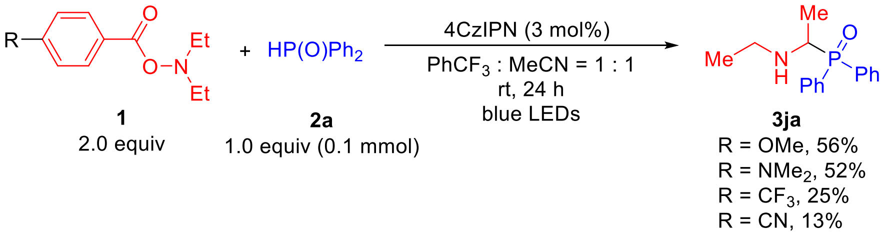

The reaction was performed following **General Procedure G**, after the reaction was completed, solvent was removed under vacuum, the crude mixture was analyzed by ^{31}P-NMR with PPh_3 as an internal standard.

These suggest that the electron-donating aryoxyl groups enhance the basicity of the benzoate anion and facilitate the 1,2-HAT process.

9. Computational Details

General Remarks

All optimizations of intermediates and transition states were calculated using unrestricted M06[14]-D3[15] /6-31G+(d,p)[16] in implicit solvent (acetonitrile) using CPCM[17] using an ultrafine (99,590) grid with the "opt=noeigen" keyword as implemented in Gaussian16. Frequency calculations, using the same method,

were used to obtain thermal corrections (at 298.15K; enthalpy and free energy) and to characterize the obtained stationary points as transition states (only one single imaginary frequency) or intermediate (zero imaginary frequencies). Conformational searches were performed manually for all intermediates and transition states, and only the lowest energy species were shown and discussed. Intrinsic reaction coordinate (IRC) calculations were undertaken to ensure transition states connected to the corresponding intermediates. Single point energy calculations using UM06-D3/6-311+G(d,p)[18] with solvent corrections calculated in implicit solvent (acetonitrile) using CPCM were also performed on all structures. The barriers to single electron transfer (SET) processes were calculated using Marcus Theory[19] with UB3LYP[20]/6-31G(d). The 3-D structures in Figures S13 and S14 were generated using GaussView 6. All other 3-D structures were generated using CYLview.[21]

Full Reference of Gaussian 16 Software

Gaussian 16, Revision B.01, M. J. Frisch, G. W. Trucks, H. B. Schlegel, G. E. Scuseria, M. A. Robb, J. R. Cheeseman, G. Scalmani, V. Barone, G. A. Petersson, H. Nakatsuji, X. Li, M. Caricato, A. V. Marenich, J. Bloino, B. G. Janesko, R. Gomperts, B. Mennucci, H. P. Hratchian, J. V. Ortiz, A. F. Izmaylov, J. L. Sonnenberg, D. Williams-Young, F. Ding, F. Lipparini, F. Egidi, J. Goings, B. Peng, A. Petrone, T. Henderson, D. Ranasinghe, V. G. Zakrzewski, J. Gao, N. Rega, G. Zheng, W. Liang, M. Hada, M. Ehara, K. Toyota, R. Fukuda, J. Hasegawa, M. Ishida, T. Nakajima, Y. Honda, O. Kitao, H. Nakai, T. Vreven, K. Throssell, J. A. Montgomery, Jr., J. E. Peralta, F. Ogliaro, M. J. Bearpark, J. J. Heyd, E. N. Brothers, K. N. Kudin, V. N. Staroverov, T. A. Keith, R. Kobayashi, J. Normand, K. Raghavachari, A. P. Rendell, J. C. Burant, S. S. Iyengar, J. Tomasi, M. Cossi, J. M. Millam, M. Klene, C. Adamo, R. Cammi, J. W. Ochterski, R. L. Martin, K. Morokuma, O. Farkas, J. B. Foresman, and D. J. Fox, Gaussian, Inc., Wallingford CT, 2016.

Supporting Figures

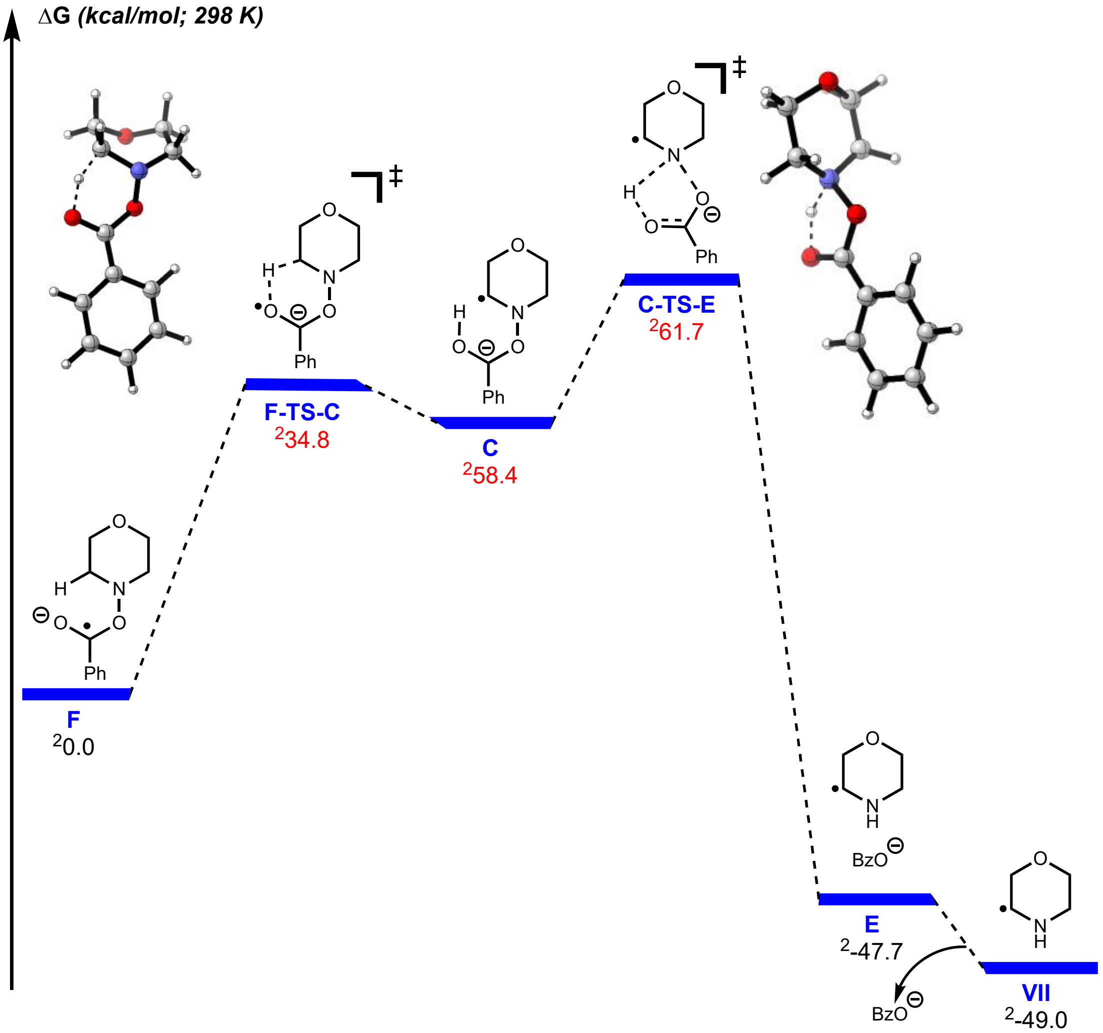

Figure S12. Generation of a-amino C-centered radical intermediate **VII** via a 1,5-HAT process. Free energies reported in red were obtained from the transition states with bonds that are forming/breaking frozen. Free energies were computed using UM06/6-311+G(d,p)-CPCM(acetonitrile)//UB3LYP/6-31G(d).

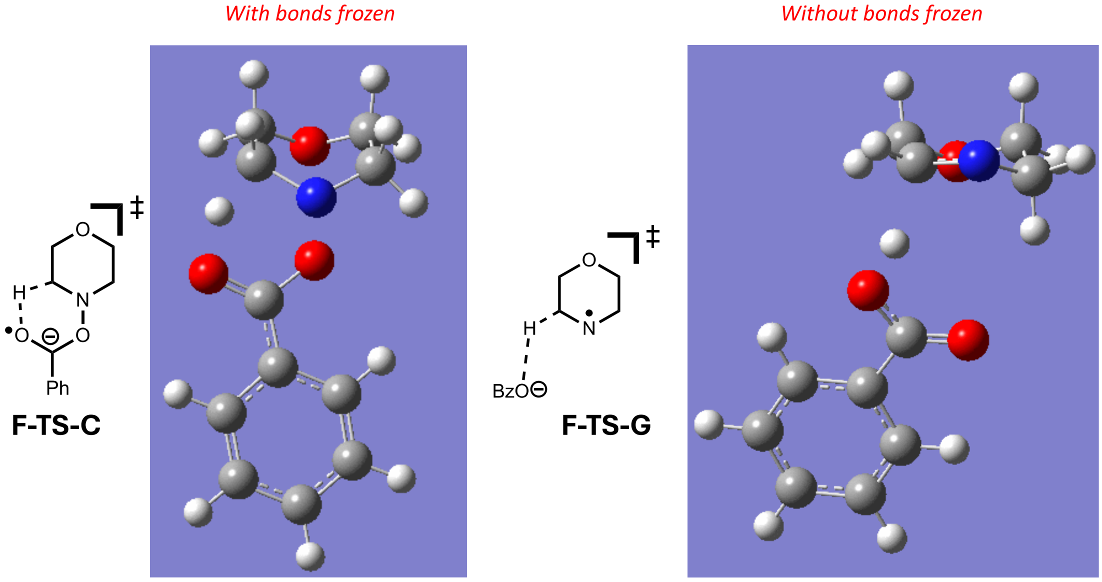

Figure S13. Removal of the bond constraints in **F-TS-C** leads to **F-TS-G.**

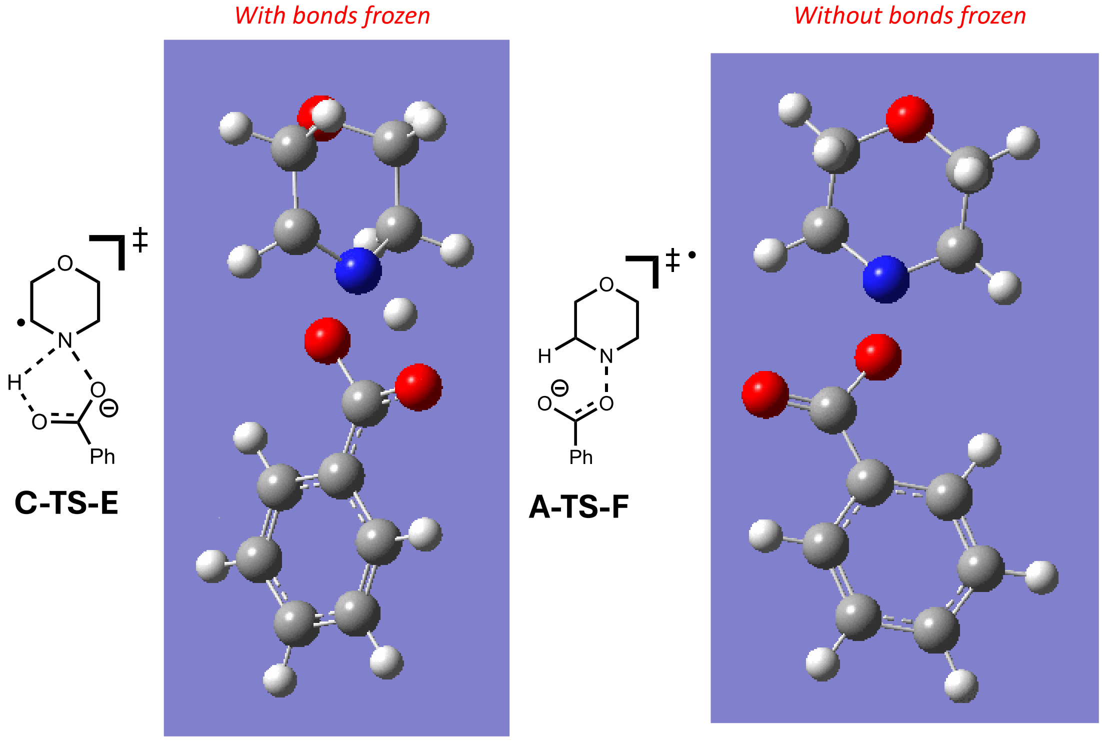

Figure S14. Removal of the bond constraints in **C-TS-E** leads to **A-TS-F.**

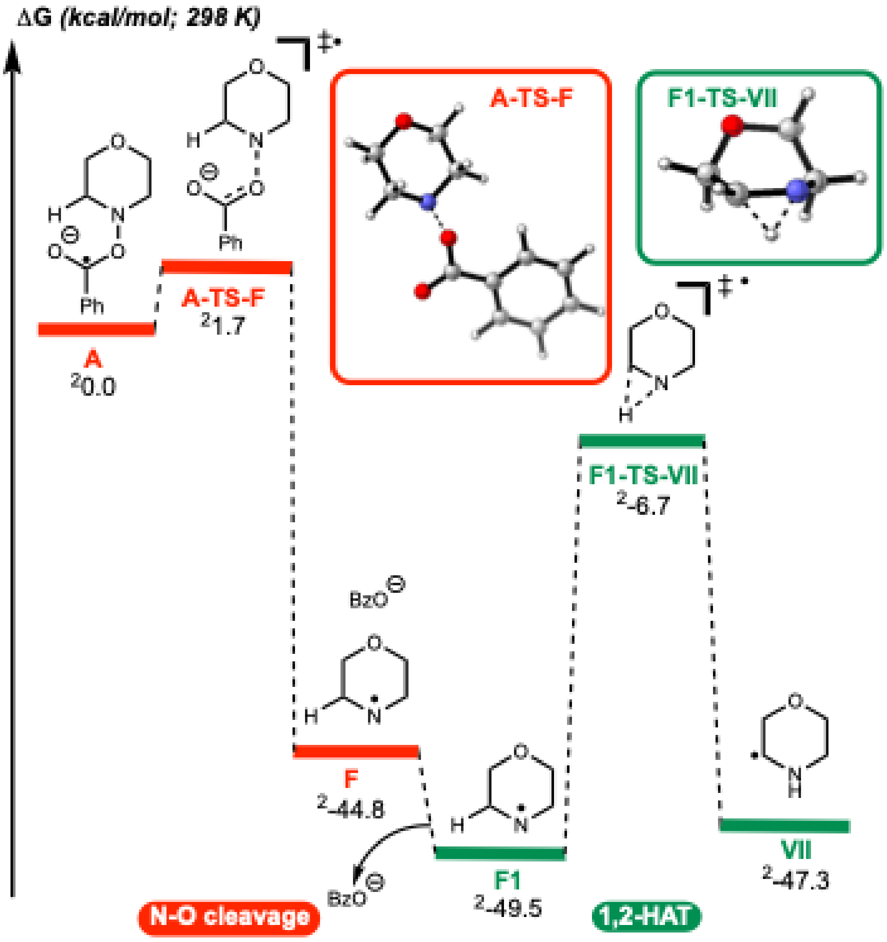

Figure S15. Generation of a-amino C-centered radical intermediate **VII** via N-O cleavage followed by direct 1,2-HAT of **F1**. Free energies were computed using UM06-D3/6-311+G(d,p)-CPCM(acetonitrile)//UM06-D3/6-31+G(d,p)-CPCM(acetonitrile).

Calculated Structures and Energies

1a

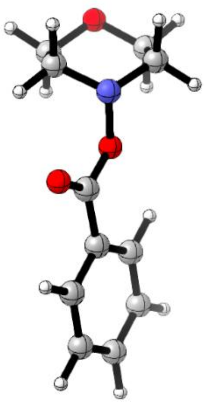

UM06-D3/6-31+G(d,p)-CPCM(acetonitrile)

Zero-point correction= 0.228673 (Hartree/Particle)

Thermal correction to Energy= 0.241269

Thermal correction to Enthalpy= 0.242214
Thermal correction to Gibbs Free Energy= 0.187987
Sum of electronic and zero-point Energies= -706.735099
Sum of electronic and thermal Energies= -706.722503
Sum of electronic and thermal Enthalpies= -706.721559
Sum of electronic and thermal Free Energies= -706.775785

UM06-D3/6-311+G(d,p)-CPCM(acetonitrile)//UM06-D3/6-31+G(d,p)-CPCM(acetonitrile)
HF = -707.1003871

A

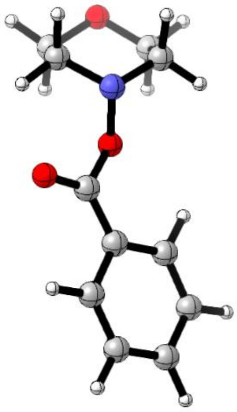

UM06-D3/6-31+G(d,p)-CPCM(acetonitrile)
Zero-point correction= 0.224793 (Hartree/Particle)
Thermal correction to Energy= 0.237670
Thermal correction to Enthalpy= 0.238614
Thermal correction to Gibbs Free Energy= 0.183707
Sum of electronic and zero-point Energies= -706.817758
Sum of electronic and thermal Energies= -706.804881
Sum of electronic and thermal Enthalpies= -706.803937
Sum of electronic and thermal Free Energies= -706.858844
UM06-D3/6-311+G(d,p)-CPCM(acetonitrile)//UM06-D3/6-31+G(d,p)-CPCM(acetonitrile)
HF = -707.1813617

A-TS-F

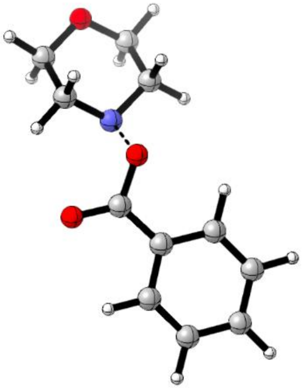

UM06-D3/6-31+G(d,p)-CPCM(acetonitrile)
Imaginary frequency = -927.23 cm^{-1}
Zero-point correction= 0.222923 (Hartree/Particle)
Thermal correction to Energy= 0.235708
Thermal correction to Enthalpy= 0.236652
Thermal correction to Gibbs Free Energy= 0.181646
Sum of electronic and zero-point Energies= -706.815041
Sum of electronic and thermal Energies= -706.802255
Sum of electronic and thermal Enthalpies= -706.801311
Sum of electronic and thermal Free Energies= -706.856318
UM06-D3/6-311+G(d,p)-CPCM(acetonitrile)//UM06-D3/6-31+G(d,p)-CPCM(acetonitrile)
HF = -707.1766561

F

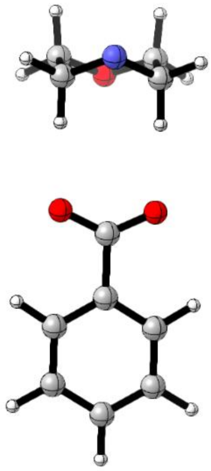

UM06-D3/6-31+G(d,p)-CPCM(acetonitrile)
Zero-point correction= 0.223471 (Hartree/Particle)
Thermal correction to Energy= 0.237741
Thermal correction to Enthalpy= 0.238685
Thermal correction to Gibbs Free Energy= 0.178857
Sum of electronic and zero-point Energies= -706.885378
Sum of electronic and thermal Energies= -706.871108
Sum of electronic and thermal Enthalpies= -706.870164
Sum of electronic and thermal Free Energies= -706.929992
UM06-D3/6-311+G(d,p)-CPCM(acetonitrile)//UM06-D3/6-31+G(d,p)-CPCM(acetonitrile)
HF = -707.247871

F-TS-G

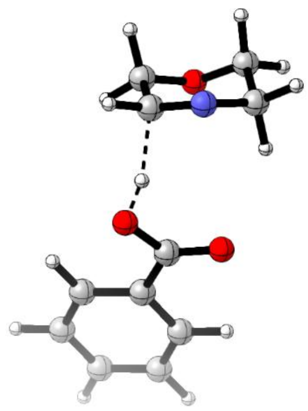

UM06-D3/6-31+G(d,p)-CPCM(acetonitrile)
Imaginary frequency = -110.05 cm^{-1}
Zero-point correction= 0.219588 (Hartree/Particle)
Thermal correction to Energy= 0.232873
Thermal correction to Enthalpy= 0.233817
Thermal correction to Gibbs Free Energy= 0.176450
Sum of electronic and zero-point Energies= -706.848637
Sum of electronic and thermal Energies= -706.835353
Sum of electronic and thermal Enthalpies= -706.834408
Sum of electronic and thermal Free Energies= -706.891775
UM06-D3/6-311+G(d,p)-CPCM(acetonitrile)//UM06-D3/6-31+G(d,p)-CPCM(acetonitrile)
HF = -707.207666

G

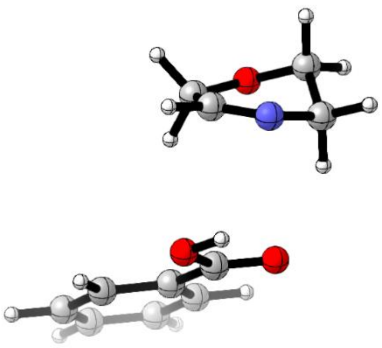

UM06-D3/6-31+G(d,p)-CPCM(acetonitrile)
Zero-point correction= 0.222385 (Hartree/Particle)
Thermal correction to Energy= 0.236862
Thermal correction to Enthalpy= 0.237806
Thermal correction to Gibbs Free Energy= 0.178887
Sum of electronic and zero-point Energies= -706.869928
Sum of electronic and thermal Energies= -706.855451
Sum of electronic and thermal Enthalpies= -706.854506
Sum of electronic and thermal Free Energies= -706.913426
UM06-D3/6-311+G(d,p)-CPCM(acetonitrile)//UM06-D3/6-31+G(d,p)-CPCM(acetonitrile)

HF = -707.2332455

G-TS-E

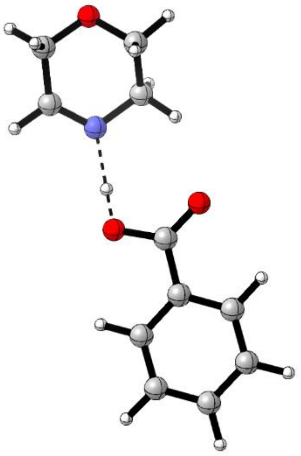

UM06-D3/6-31+G(d,p)-CPCM(acetonitrile)

Imaginary frequency = -4030.67 cm^{-1}

Zero-point correction= 0.216692 (Hartree/Particle)

Thermal correction to Energy= 0.230762

Thermal correction to Enthalpy= 0.231706

Thermal correction to Gibbs Free Energy= 0.171826

Sum of electronic and zero-point Energies= -706.859174

Sum of electronic and thermal Energies= -706.845104

Sum of electronic and thermal Enthalpies= -706.844160

Sum of electronic and thermal Free Energies= -706.904040

UM06-D3/6-311+G(d,p)-CPCM(acetonitrile)//UM06-D3/6-31+G(d,p)-CPCM(acetonitrile)

HF = -707.2158856

E

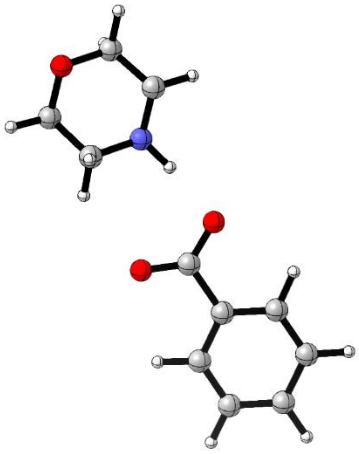

UM06-D3/6-31+G(d,p)-CPCM(acetonitrile)

Zero-point correction= 0.223902 (Hartree/Particle)

Thermal correction to Energy= 0.238247

Thermal correction to Enthalpy= 0.239191

Thermal correction to Gibbs Free Energy= 0.178519

Sum of electronic and zero-point Energies= -706.887455
Sum of electronic and thermal Energies= -706.873110
Sum of electronic and thermal Enthalpies= -706.872166
Sum of electronic and thermal Free Energies= -706.932838
UM06-D3/6-311+G(d,p)-CPCM(acetonitrile)//UM06-D3/6-31+G(d,p)-CPCM(acetonitrile)
HF = -707.251036

VII

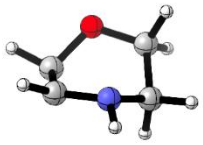

UM06-D3/6-31+G(d,p)-CPCM(acetonitrile)
Zero-point correction= 0.120111 (Hartree/Particle)
Thermal correction to Energy= 0.125978
Thermal correction to Enthalpy= 0.126922
Thermal correction to Gibbs Free Energy= 0.090271
Sum of electronic and zero-point Energies= -286.847603
Sum of electronic and thermal Energies= -286.841736
Sum of electronic and thermal Enthalpies= -286.840791
Sum of electronic and thermal Free Energies= -286.877443
UM06-D3/6-311+G(d,p)-CPCM(acetonitrile)//UM06-D3/6-31+G(d,p)-CPCM(acetonitrile)
HF = -287.0239973

OBz-

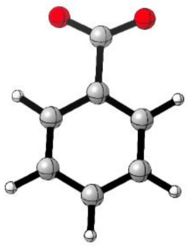

UM06-D3/6-31+G(d,p)-CPCM(acetonitrile)
Zero-point correction= 0.102068 (Hartree/Particle)
Thermal correction to Energy= 0.109041
Thermal correction to Enthalpy= 0.109985
Thermal correction to Gibbs Free Energy= 0.069809
Sum of electronic and zero-point Energies= -420.024323
Sum of electronic and thermal Energies= -420.017349
Sum of electronic and thermal Enthalpies= -420.016405
Sum of electronic and thermal Free Energies= -420.056581
UM06-D3/6-311+G(d,p)-CPCM(acetonitrile)//UM06-D3/6-31+G(d,p)-CPCM(acetonitrile)
HF = -420.2090788

F1

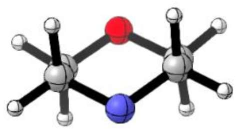

UM06-D3/6-31+G(d,p)-CPCM(acetonitrile)
Zero-point correction= 0.120370 (Hartree/Particle)
Thermal correction to Energy= 0.125701
Thermal correction to Enthalpy= 0.126645
Thermal correction to Gibbs Free Energy= 0.091271
Sum of electronic and zero-point Energies= -286.852643
Sum of electronic and thermal Energies= -286.847312
Sum of electronic and thermal Enthalpies= -286.846368
Sum of electronic and thermal Free Energies= -286.881742
UM06-D3/6-311+G(d,p)-CPCM(acetonitrile)//UM06-D3/6-31+G(d,p)-CPCM(acetonitrile)
HF = -287.0284869

F1-TS-VII

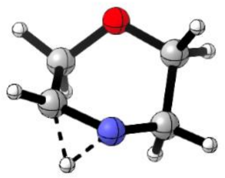

UM06-D3/6-31+G(d,p)-CPCM(acetonitrile)
Imaginary frequency = -1807.74 cm^{-1}
Zero-point correction= 0.115749 (Hartree/Particle)
Thermal correction to Energy= 0.121247
Thermal correction to Enthalpy= 0.122191
Thermal correction to Gibbs Free Energy= 0.086140
Sum of electronic and zero-point Energies= -286.784180
Sum of electronic and thermal Energies= -286.778682
Sum of electronic and thermal Enthalpies= -286.777738
Sum of electronic and thermal Free Energies= -286.813789
UM06-D3/6-311+G(d,p)-CPCM(acetonitrile)//UM06-D3/6-31+G(d,p)-CPCM(acetonitrile)
HF = -286.955211

4CzIPN

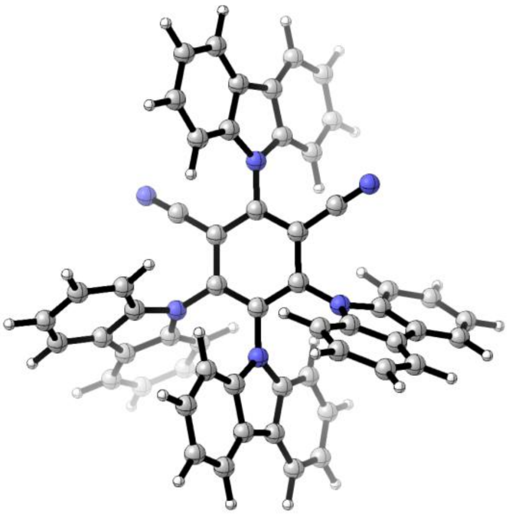

UB3LYP/6-31G(d)

Zero-point correction= 0.719079 (Hartree/Particle)

Thermal correction to Energy= 0.765254

Thermal correction to Enthalpy= 0.766198

Thermal correction to Gibbs Free Energy= 0.635740

Sum of electronic and zero-point Energies= -2480.974163

Sum of electronic and thermal Energies= -2480.927988

Sum of electronic and thermal Enthalpies= -2480.927043

Sum of electronic and thermal Free Energies= -2481.057502

***4CzIPN**

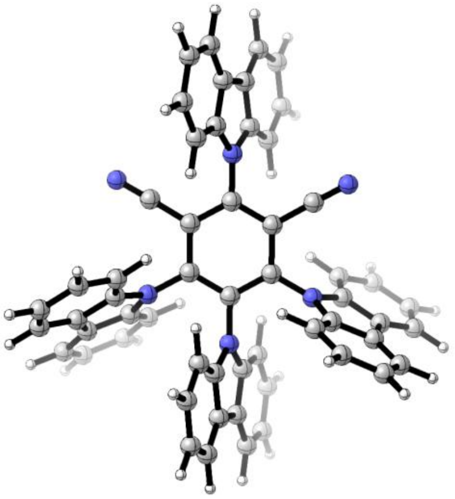

TD-B3LYP/6-31G(d)

Zero-point correction= 0.717447 (Hartree/Particle)

Thermal correction to Energy= 0.763142

Thermal correction to Enthalpy= 0.764086

Thermal correction to Gibbs Free Energy= 0.635038

Sum of electronic and zero-point Energies= -2480.966838

Sum of electronic and thermal Energies= -2480.921143

Sum of electronic and thermal Enthalpies= -2480.920198
Sum of electronic and thermal Free Energies= -2481.049246

4CzIPN-radical anion

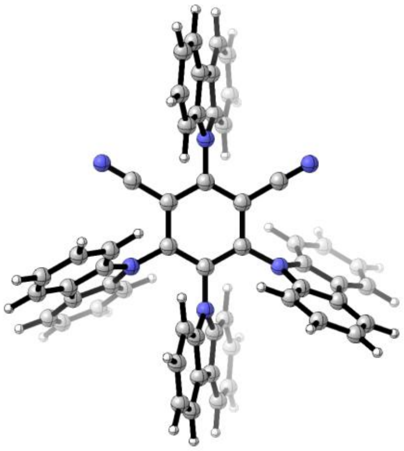

UB3LYP/6-31G(d)
Zero-point correction= 0.719477 (Hartree/Particle)
Thermal correction to Energy= 0.765589
Thermal correction to Enthalpy= 0.766533
Thermal correction to Gibbs Free Energy= 0.635790
Sum of electronic and zero-point Energies= -2481.119527
Sum of electronic and thermal Energies= -2481.073415
Sum of electronic and thermal Enthalpies= -2481.072470
Sum of electronic and thermal Free Energies= -2481.203213

J

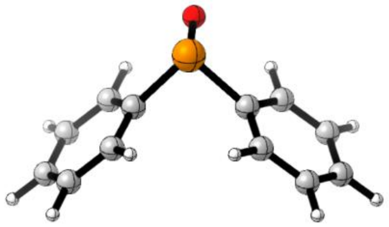

UB3LYP/6-31G(d)
Zero-point correction= 0.183944 (Hartree/Particle)
Thermal correction to Energy= 0.195857
Thermal correction to Enthalpy= 0.196801
Thermal correction to Gibbs Free Energy= 0.144244
Sum of electronic and zero-point Energies= -879.730690
Sum of electronic and thermal Energies= -879.718777
Sum of electronic and thermal Enthalpies= -879.717833
Sum of electronic and thermal Free Energies= -879.770390

K

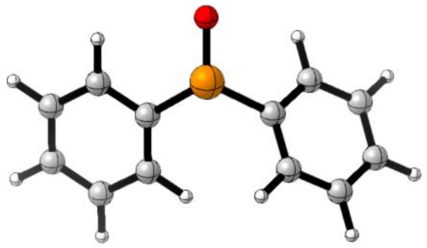

UB3LYP/6-31G(d)

Zero-point correction= 0.186457 (Hartree/Particle)
Thermal correction to Energy= 0.198272
Thermal correction to Enthalpy= 0.199216
Thermal correction to Gibbs Free Energy= 0.146060
Sum of electronic and zero-point Energies= -879.669494
Sum of electronic and thermal Energies= -879.657678
Sum of electronic and thermal Enthalpies= -879.656734
Sum of electronic and thermal Free Energies= -879.709890

10. X-ray Crystal Structure of Compound 3ao

Sample preparation: To an 8 mL vial containing **3ao** (10 mg) was added hexane and $CHCl_3$ (about 3.0 mL). The single crystal **3ao** was obtained by slowly evaporating the solvent at room temperature under air.

Crystal measurement: The integration of the data using a monoclinic unit cell yielded a total of 29036 reflections to a maximum θ angle of 28.31° (0.75 Å resolution), of which 3863 were independent (average redundancy 7.516, completeness = 99.8%, R_{int} = 4.22%, R_{sig} = 2.54%) and 3413 (88.35%) were greater than 2σ(F^2). The final cell constants of a = 17.9924(6) Å, b = 11.0902(4) Å, c = 15.8252(6) Å, β = 100.2420(10)°, volume = 3107.43(19) Å^3, are based upon the refinement of the XYZ-centroids of reflections above 20 σ(I). The calculated minimum and maximum transmission coefficients (based on crystal size) are 0.6908 and 0.7457.

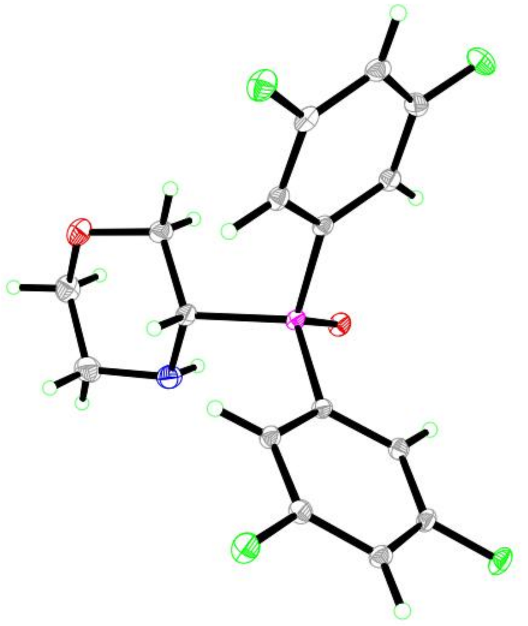

Figure S16. Crystal structure of **3ao** (**CCDC** 2293469)

Crystal data and structure refinement for 3ao.

Identification code	**3ao**	
Chemical formula	$C_{16}H_{14}F_4NO_2P$	
Formula weight	359.25 g/mol	
Wavelength	0.71073 Å	
Crystal size	0.180 x 0.200 x 0.220 mm	
Crystal system	monoclinic	
Space group	C 1 2/c 1	
Unit cell dimensions	a = 17.9924(6) Å	α = 90°
	b = 11.0902(4) Å	β = 100.2420(10)°
	c = 15.8252(6) Å	γ = 90°
Volume	3107.43(19) $Å^3$	
Z	8	
Density (calculated)	1.536 g/cm^3	
Absorption coefficient	0.230 mm^{-1}	
F(000)	1472	
Theta range for data collection	2.17 to 28.31°	
Index ranges	-23<=h<=23, -14<=k<=14, -21<=l<=21	
Reflections collected	29036	
Independent reflections	3863 [R(int) = 0.0422]	
Max. and min. transmission	0.7457 and 0.6908	
Structure solution technique	direct methods	
Structure solution program	SHELXT 2018/2 (Sheldrick, 2018)	
Refinement method	Full-matrix least-squares on F2	
Refinement program	SHELXL 2018/3 (Sheldrick, 2015)	

Function minimized	Σ w(F_o^2 - F_c^2)^2
Data / restraints / parameters	3863 / 1 / 221
Goodness-of-fit on F2	1.041
Δ/σmax	0.001
Final R indices	3413 data; I>2σ(I) R1 = 0.0404, wR2 = 0.0998
	all data R1 = 0.0461, wR2 = 0.1036
Weighting scheme	w=1/[$\sigma^2(F_o^2)+(0.0416P)^2+5.8014P$]
	where P=($F_o^2+2F_c^2$)/3
Largest diff. peak and hole	0.719 and -0.363 eÅ^{-3}
R.M.S. deviation from mean	0.058 eÅ^{-3}

11. Supplementary References

1. Berman, A. M.; Johnson, J. S., Copper-Catalyzed Electrophilic Amination of Organozinc Nucleophiles: Documentation of *O*-Benzoyl Hydroxylamines as Broadly Useful $R_2N(+)$ and RHN(+) Synthons. *J. Org. Chem.* 2**006**, *71*, 219–224.
2. Yotphan, S.; Beukeaw, D.; Reutrakul, V., Synthesis of 2-aminobenzoxazoles via copper-catalyzed electrophilic amination of benzoxazoles with *O*-benzoyl hydroxylamines. *Tetrahedron* **2013**, *69*, 6627–6633.
3. Yang, Z.; Jiang, K.; Chen, Y. C.; Wei, Y., Copper-Catalyzed Dihydroquinolinone Synthesis from Isocyanides and *O*-Benzoyl Hydroxylamines. *J. Org. Chem.* **2019**, *84*, 3725–3734.
4. Bouarfa, S.; GraßI, S.; Ivanova, M.; Langlais, T.; Bentabed-Ababsa, G.; Lassagne, F.; Erb, W.; Roisnel, T.; Dorcet, V.; Knochel, P.; Mongin, F., Copper- and Cobalt-Catalyzed Syntheses of Thiophene-Based Tertiary Amines. *Eur. J. Org. Chem.* **2019**, *2019*, 3244–3258.
5. Wu, J. Y.; Li, L. Q.; Liu, M. T; Bai, L.; Luan, X. J., Selective C(sp^3)–N Bond Cleavage of *N*,*N*-Dialkyl Tertiary Amines with the Loss of a Large Alkyl Group via an S_N1 Pathway. *Angew. Chem., Int. Ed.* **2022**, *61*, e202113820.
6. Yuan, Y.; Zhang, Y. C.; Li, W. B.; Zhao, Y. Y.; Wu, X. F., Regioselective and Enantioselective Copper-Catalyzed Hydroaminocarbonylation of Unactivated Alkenes and Alkynes. *Angew. Chem. Int. Ed.* **2023**, *62*, e202309993.
7. Chen, J. X.; Xu, Y. Z.; Shao, W.; Ji, J. H.; Wang, B. Q.; Yang, M. Y.; Mao, G. J.; Xiao, F. H.; Deng, G. J., Pd-Catalyzed C – O Bond Formation Enabling the Synthesis of Congested *N*,*N*,*O*-Trisubstituted Hydroxylamines. *Org. Lett.* **2022**, *24*, 8271–8276.
8. Xu, Q.; Zhao, C. Q.; Han, L. B., Stereospecific Nucleophilic Substitution of Optically Pure *H*-Phosphinates: A General Way for the Preparation of Chiral P-Stereogenic Phosphine Oxides. *J. Am. Chem. Soc.* **2008**, *130*, 12648–12655.
9. Peng, P.; Peng, L.; Wang, G. Y.; Wang, F. Y.; Luo, Y.; Lei, A. W., Visible light mediated aerobic radical C–H phosphorization toward arylphosphonates. *Org. Chem. Front.* **2016**, *3*, 749–752.
10. Zhou, X. C.; Xiong, T.; Jiang, J., Gold-catalyzed redox cycloisomerization/nucleophilic addition/reduction: direct access to 2-phosphoryl indolin-3-ones. *Chem. Commun.* **2022,** *58*, 8568–8571.
11. Li, C. K.; Tao, Z. K.; Shoberu, A.; Zhang, W.; Zou, J. P., Copper-Catalyzed Cross-Coupling of Alkyl and Phosphorus Radicals for C(sp^3)–P Bond Formation. *Org. Lett.* **2022,** *24*, 6083–6087.
12. Su, S. Y.; Guo, Y. H.; Parnitzke, B.; Poerio, T.; Derosa, J., A Voltage-Controlled Strategy for

Modular Shono-Type Amination. *J. Am. Chem. Soc.* **2024**, *146*, 28663–28668.

13. Smolobochkin, A. V.; Turmanov, R. A.; Gazizov, A. S.; Voloshina, A. D.; Voronina, J. K.; Sapunova, A. S.; Burilov, A. R.; Pudovik, M. A., One-pot imination / Arbuzov reaction of 4-aminobutanal derivatives: Synthesis of 2-phosphorylpyrrolidines and evaluation of anticancer activity. *Tetrahedron* **2020**, *76*, 131369.
14. Zhao, Y.; D. G. Truhlar, D. G., The M06 suite of density functionals for main group thermochemistry, thermochemical kinetics, noncovalent interactions, excited states, and transition elements: two new functionals and systematic testing of four M06-class functionals and 12 other functionals. *Theor. Chem. Acc.*, **2008**, *120*, 215-241.
15. Grimme, S.; Antony, J.; Ehrlich, S.; Krieg, H. A consistent and accurate *ab initio* parametrization of density functional dispersion correction (DFT-D) for the 94 elements H-Pu. *J. Chem. Phys*., **2010**, *132*, 154104.
16. Petersson, G. A.; Al-Laham, M. A., A complete basis set model chemistry. II. Open-shell systems and the total energies of the first-row atoms. *J. Chem. Phys.*, **1991**, *94*, 6081-6090.
17. Cossi, M.; Rega, N.; Scalmani, G.; Barone, V., Energies, structures, and electronic properties of molecules in solution with the C-PCM solvation model. *J. Comput. Chem.* **2003,** *24*, 669-681.
18. (a) McLean, A. D.; Chandler, G. S., Contracted Gaussian-basis sets for molecular calculations. 1. 2nd row atoms, Z=11-18. *J. Chem. Phys.*, **1980**, *72*, 5639-5648. (b) Raghavachari, K.; Binkley, J. S.; Seeger, R.; Pople, J. A., Self-Consistent Molecular Orbital Methods. 20. Basis set for correlated wave-functions. *J. Chem. Phys.*, **1980**, *72*, 650-654.
19. (a) Marcus, R. A. On the Theory of Oxidation-Reduction Reactions Involving Electron Transfer. III. Applications to Data on the Rates of Organic Redox Reactions *J. Chem. Phys.* **1957**, *26*, 872–877. (b) Hush, N. S. Adiabatic Rate Processes at Electrodes. I. Energy-Charge Relationships *J. Chem. Phys.* **1958**, *28*, 962–972.
20. (a) Lee, C.; Yang, W.; Parr, R. G., Development of the Colle-Salvetti correlation-energy formula into a functional of the electron density. *Phys. Rev. B* **1988,** *37*, 785–789. (b) Becke, A. D., Density-functional thermochemistry. III. The role of exact exchange. *J. Chem. Phys.* **1993,** *98*, 5648–5652.
21. CYLview20; Legault, C. Y., Université de Sherbrooke, 2020 (http://www.cylview.org)

12. NMR Spectra of the Products

Figure S17. ^{1}H NMR spectra (400 MHz, Chloroform-*d*) of (3a*R*,7a*S*)-Octahydro-2*H*-isoindol-2-yl benzoate (1h).

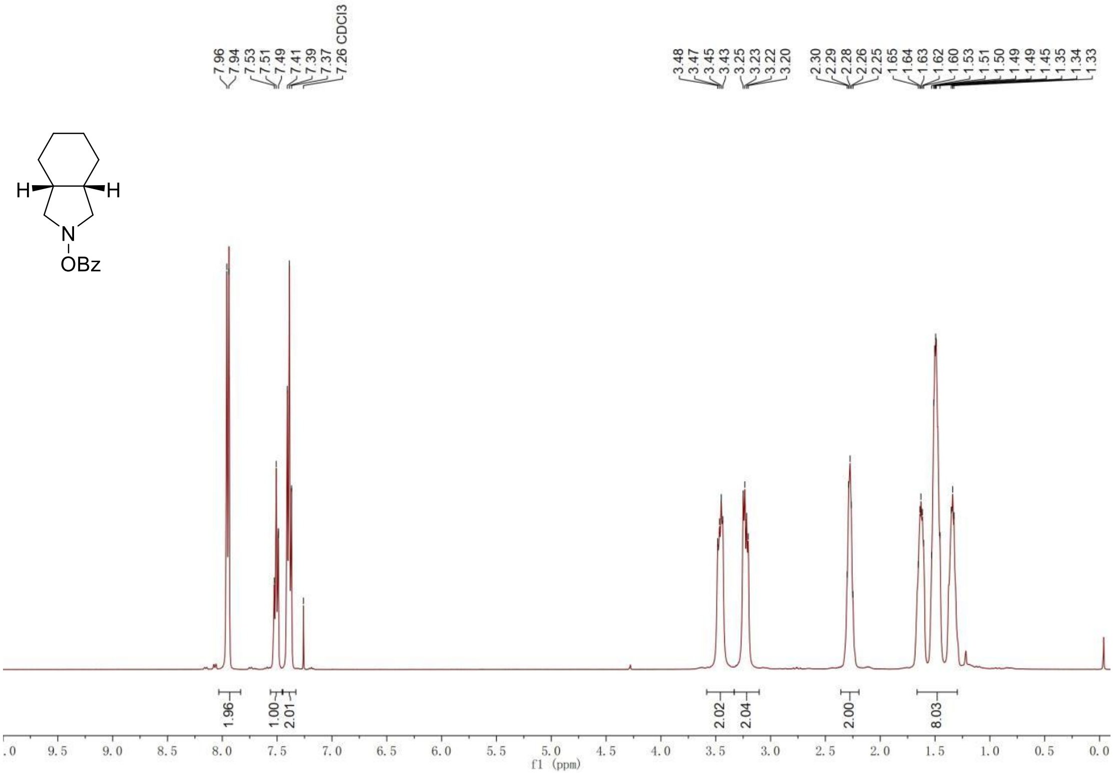

Figure S18. ^{13}C{^{1}H} NMR spectra (100 MHz, Chloroform-*d*) of (3a*R*,7a*S*)-Octahydro-2*H*-isoindol-2-yl benzoate (1h).

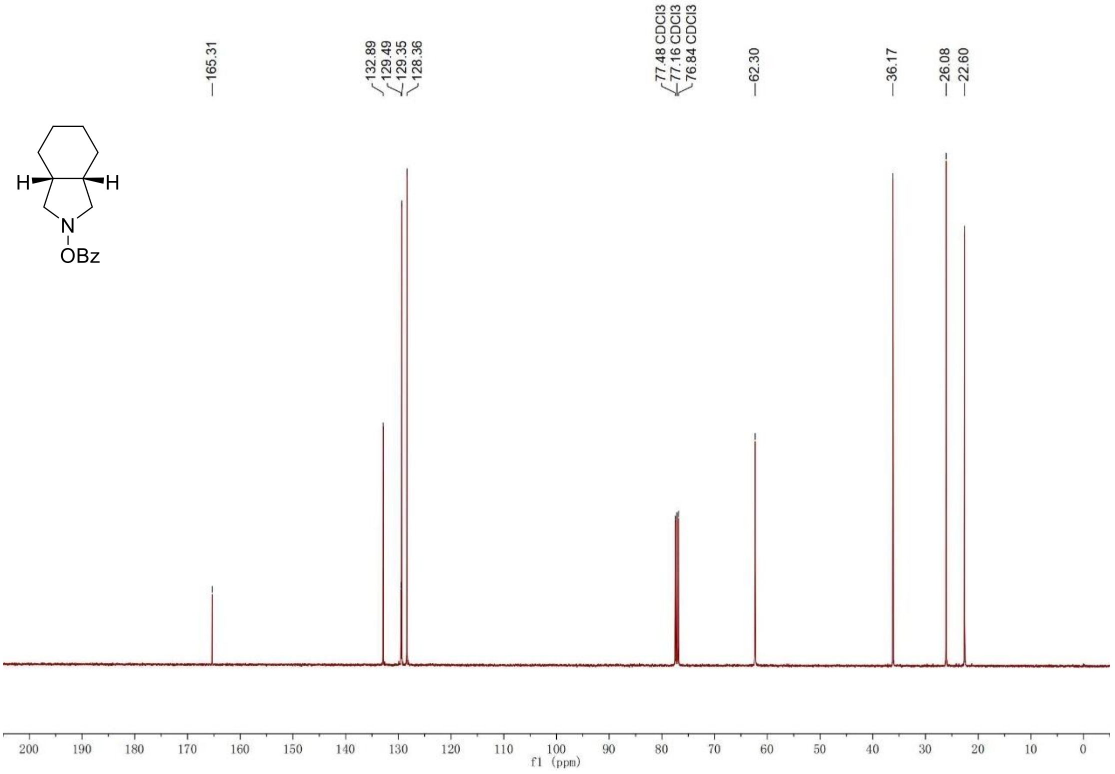

Figure S19. **^{1}H NMR spectra (400 MHz, Chloroform-*d*) of *O*-Benzoyl-*N*-methyl-*N*-(tetrahydro-2*H*-pyran-4-yl)hydroxylamine (1n).**

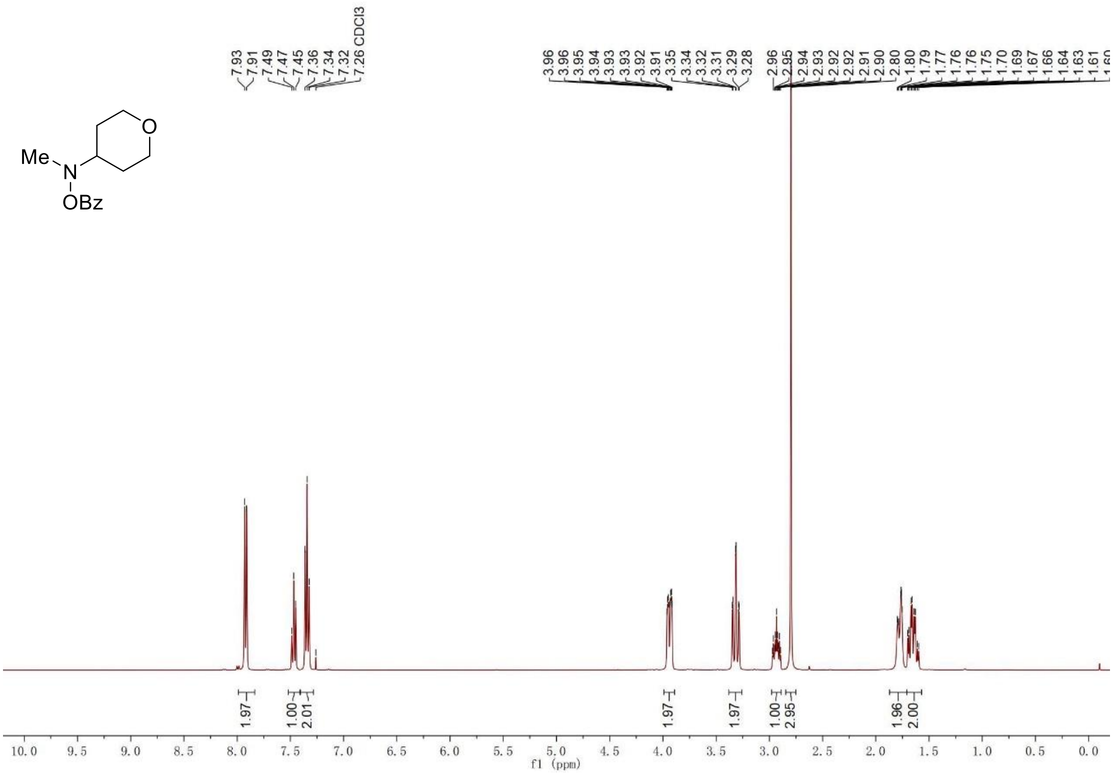

Figure S20. **^{13}C{^{1}H} NMR spectra (100 MHz, Chloroform-*d*) of *O*-Benzoyl-*N*-methyl-*N*-(tetrahydro-2*H*-pyran-4-yl)hydroxylamine (1n).**

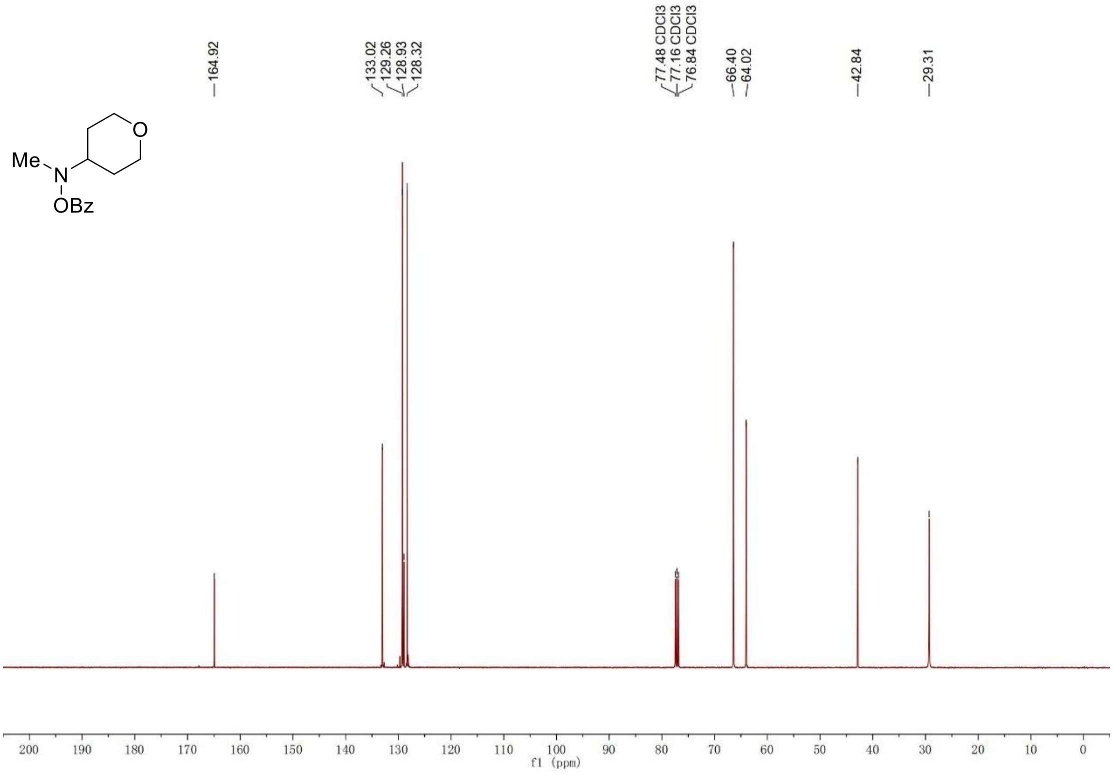

Figure S21. $^{13}C\{^{1}H\}$ **NMR spectra (100 MHz, Chloroform-*d*) of *O*-benzoyl-*N*,*N*-bis(methyl-*d*$_3$)hydroxylamine (*d*-1i).**

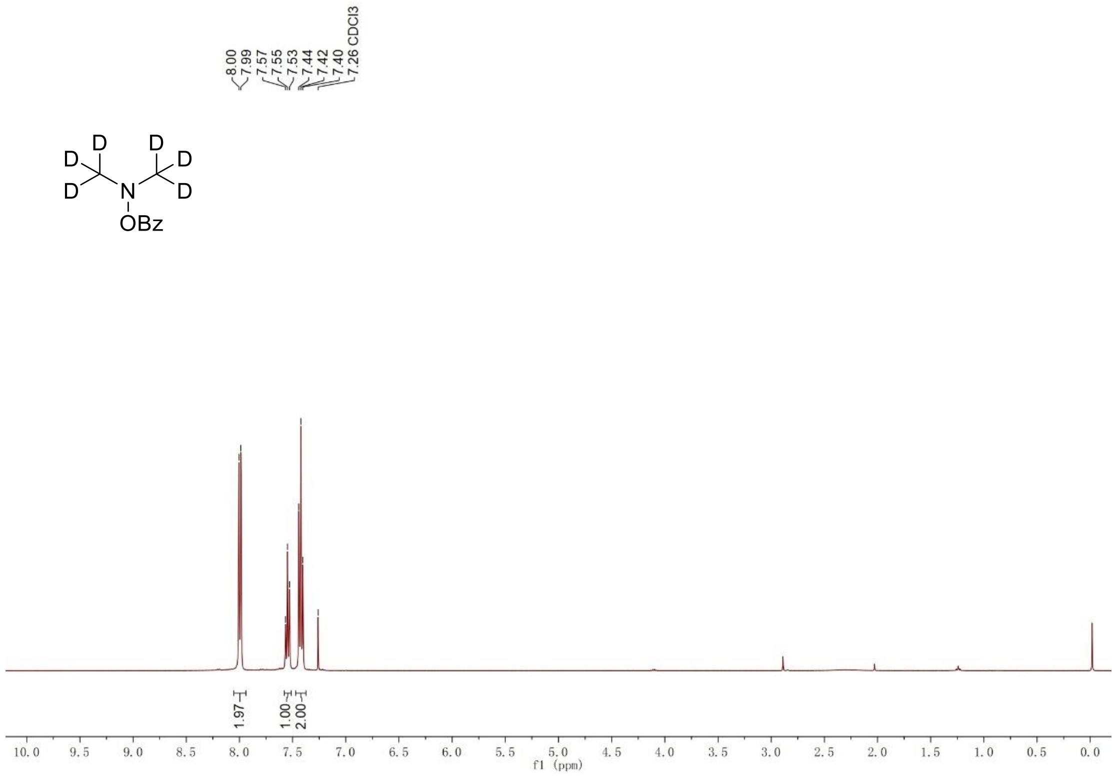

Figure S22. $^{13}C\{^{1}H\}$ **NMR spectra (100 MHz, Chloroform-*d*) of *O*-benzoyl-*N*,*N*-bis(methyl-*d*$_3$)hydroxylamine (*d*-1i).**

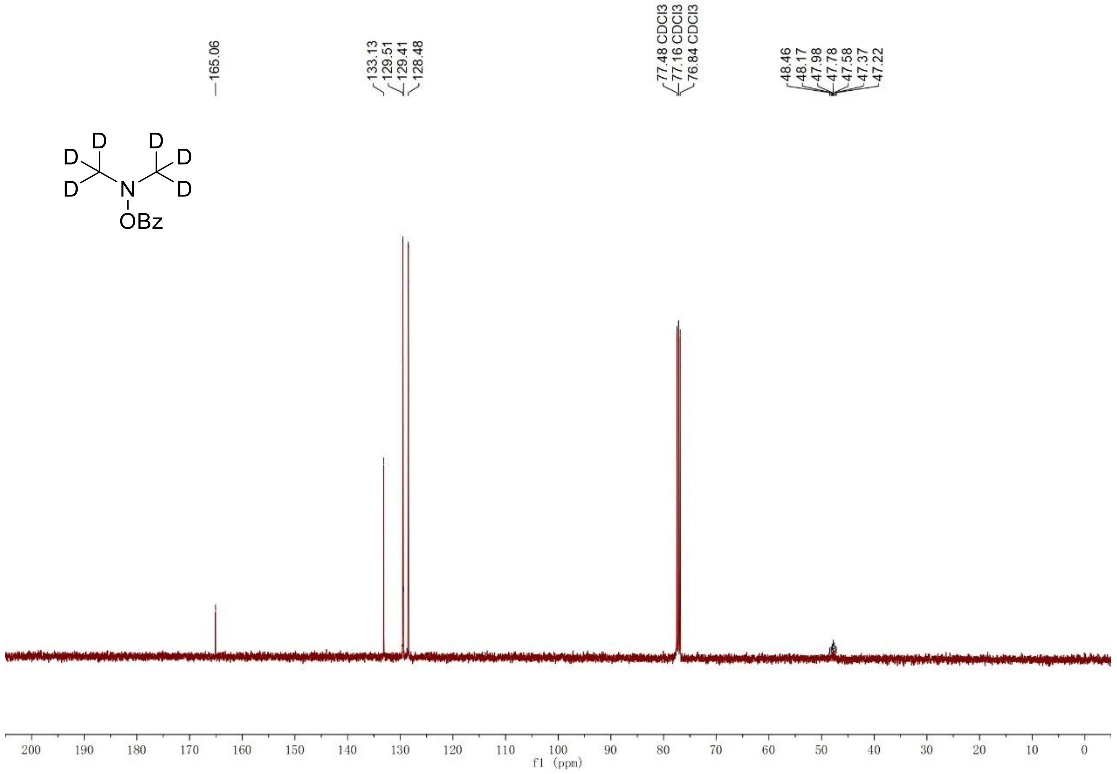

Figure S23. ^{1}**H NMR spectra (400 MHz, Chloroform-*d*) of *O*-Benzoyl-*N*-(*tert*-butyl)-*N*-(4-methylpentyl)hydroxylamine (1q).**

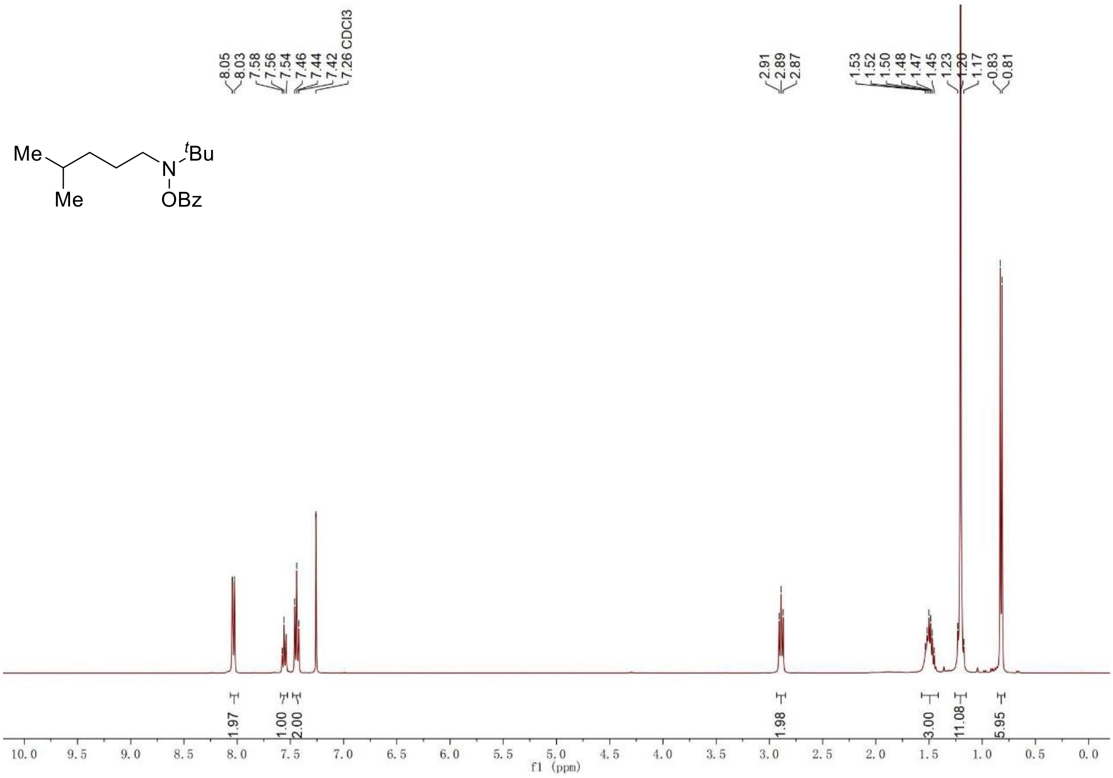

Figure S24. ^{13}**C{**^{1}**H} NMR spectra (100 MHz, Chloroform-*d*) of *O*-Benzoyl-*N*-(*tert*-butyl)-*N*-(4-methylpentyl)hydroxylamine (1q).**

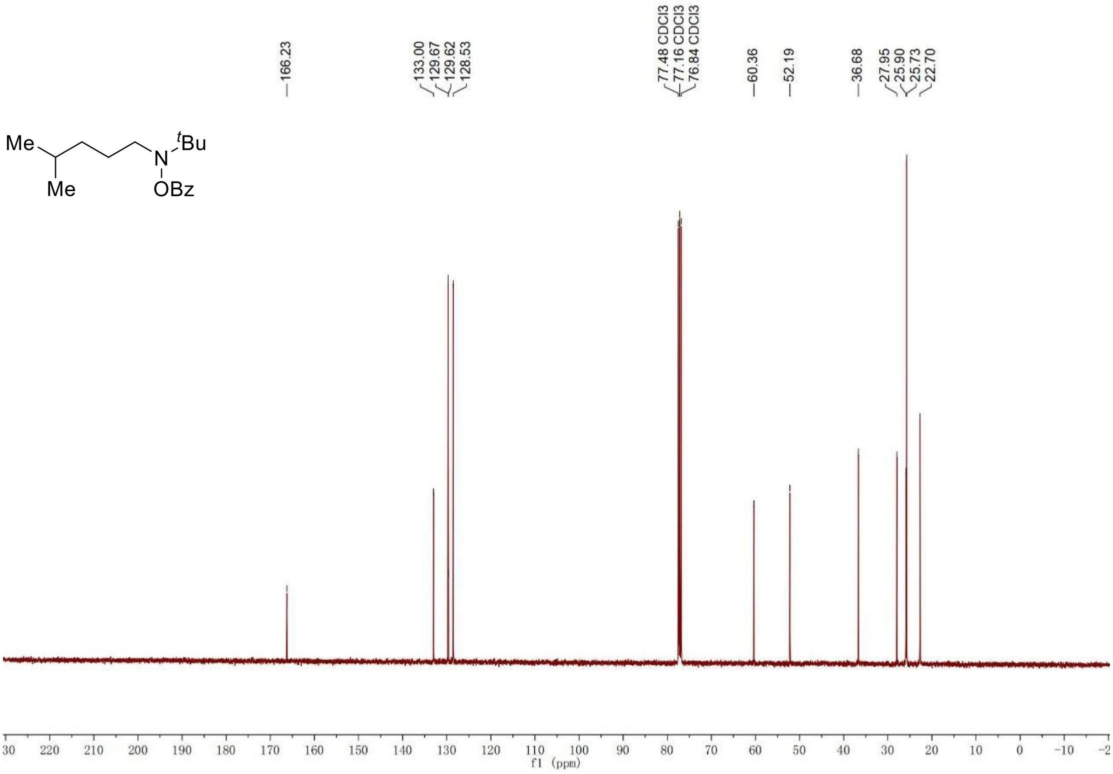

Figure S25. **^{1}H NMR spectra (400 MHz, Chloroform-*d*) of *O*-Benzoyl-*N*-(*tert*-butyl)-*N*-(4-phenylbutyl)hydroxylamine (1r).**

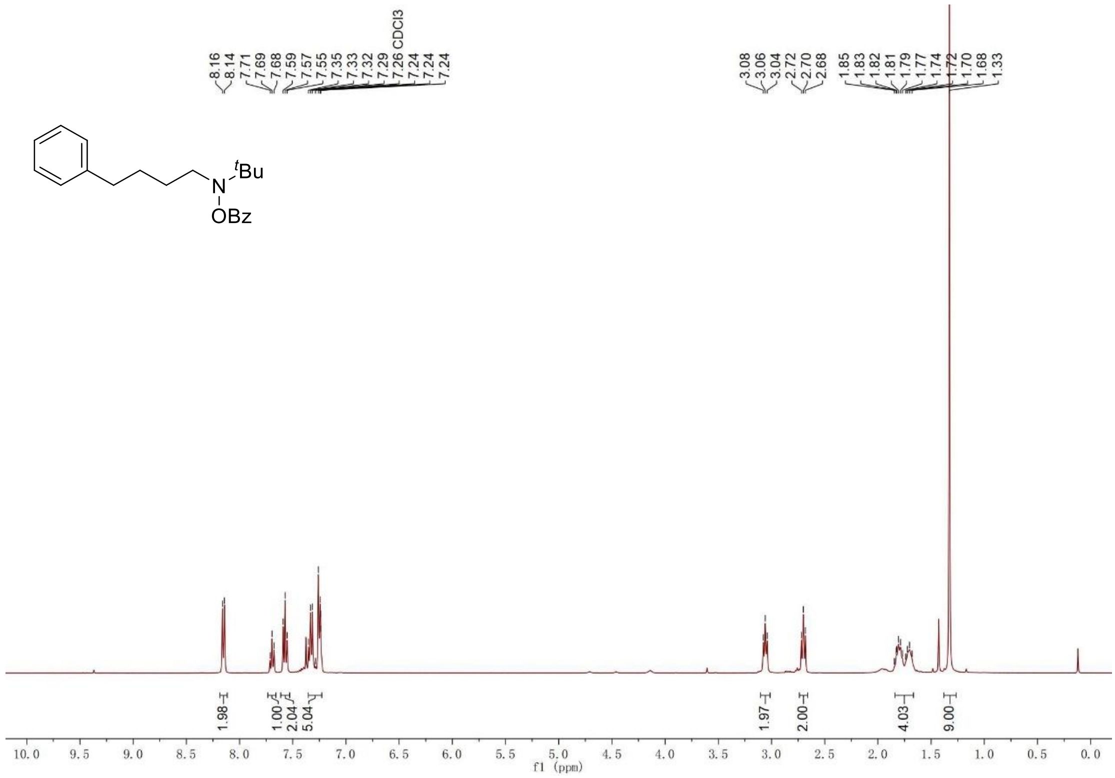

Figure S26. **^{13}C{^{1}H} NMR spectra (100 MHz, Chloroform-*d*) of *O*-Benzoyl-*N*-(*tert*-butyl)-*N*-(4-phenylbutyl)hydroxylamine (1r).**

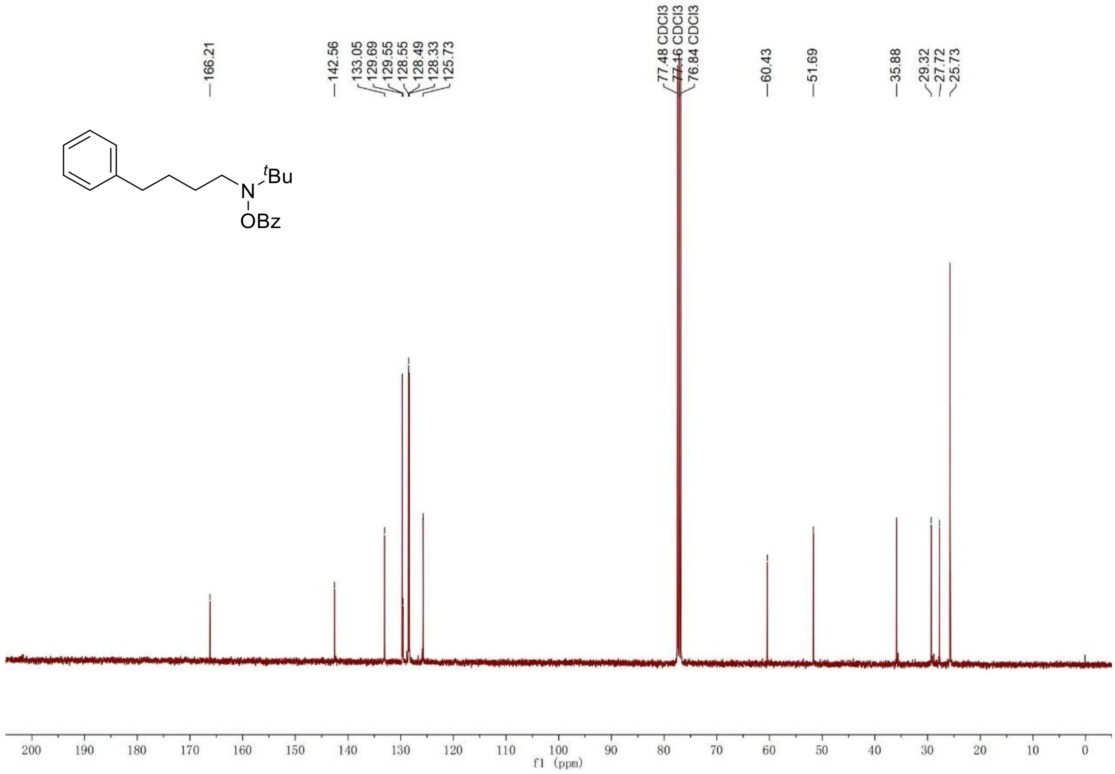

Figure S27. ^{1}H NMR spectra (400 MHz, Chloroform-*d*) of Morpholin-3-yldiphenylphosphine oxide (3aa).

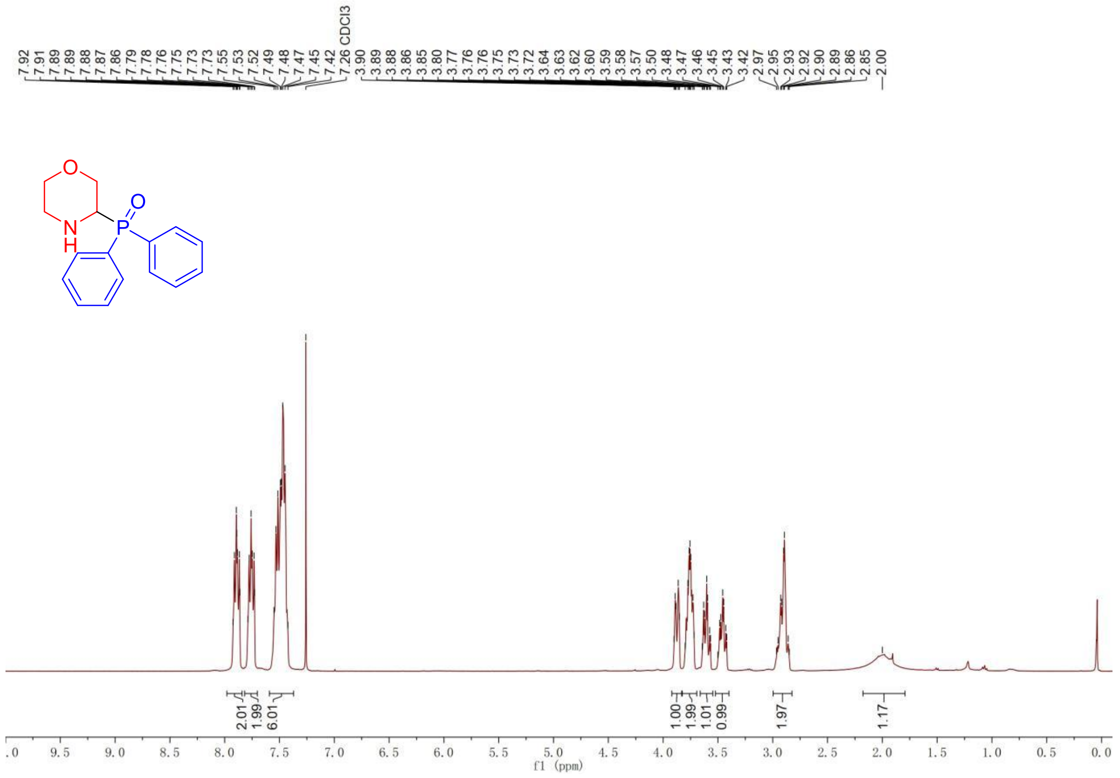

Figure S28. ^{13}C{^{1}H} NMR spectra (100 MHz, Chloroform-*d*) of Morpholin-3-yldiphenylphosphine oxide (3aa).

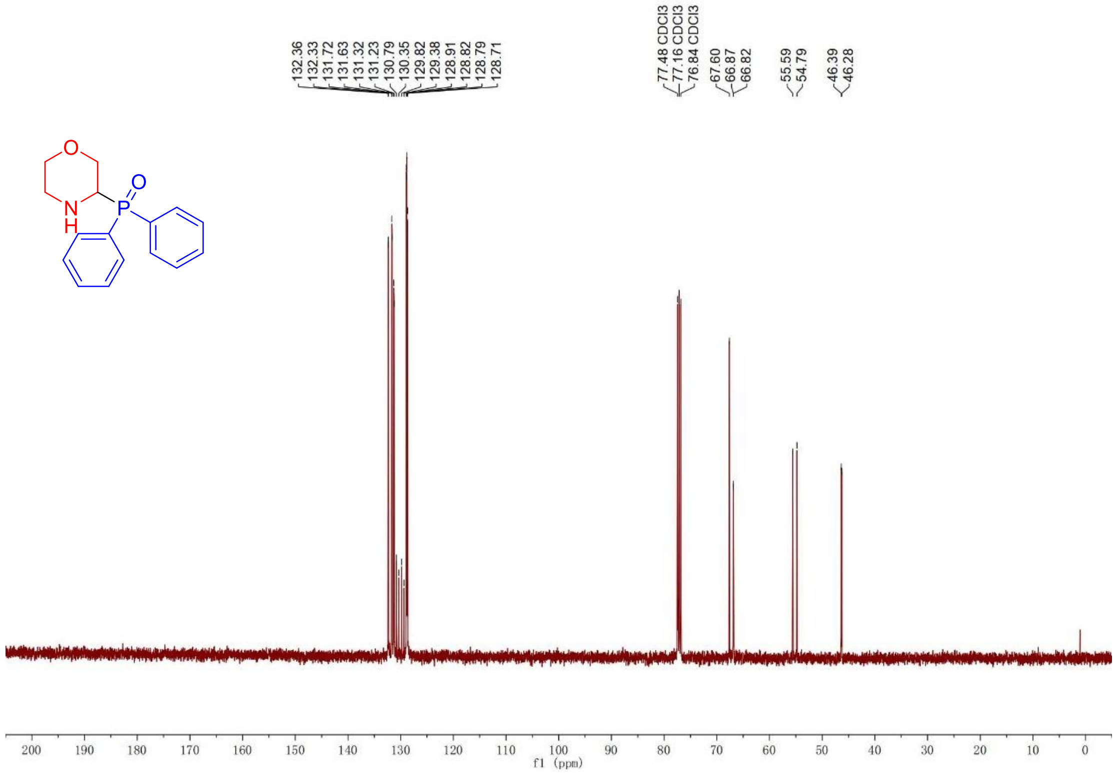

Figure S29. ^{31}P NMR spectra (162 MHz, Chloroform-*d*) of Morpholin-3-yldiphenylphosphine oxide (3aa).

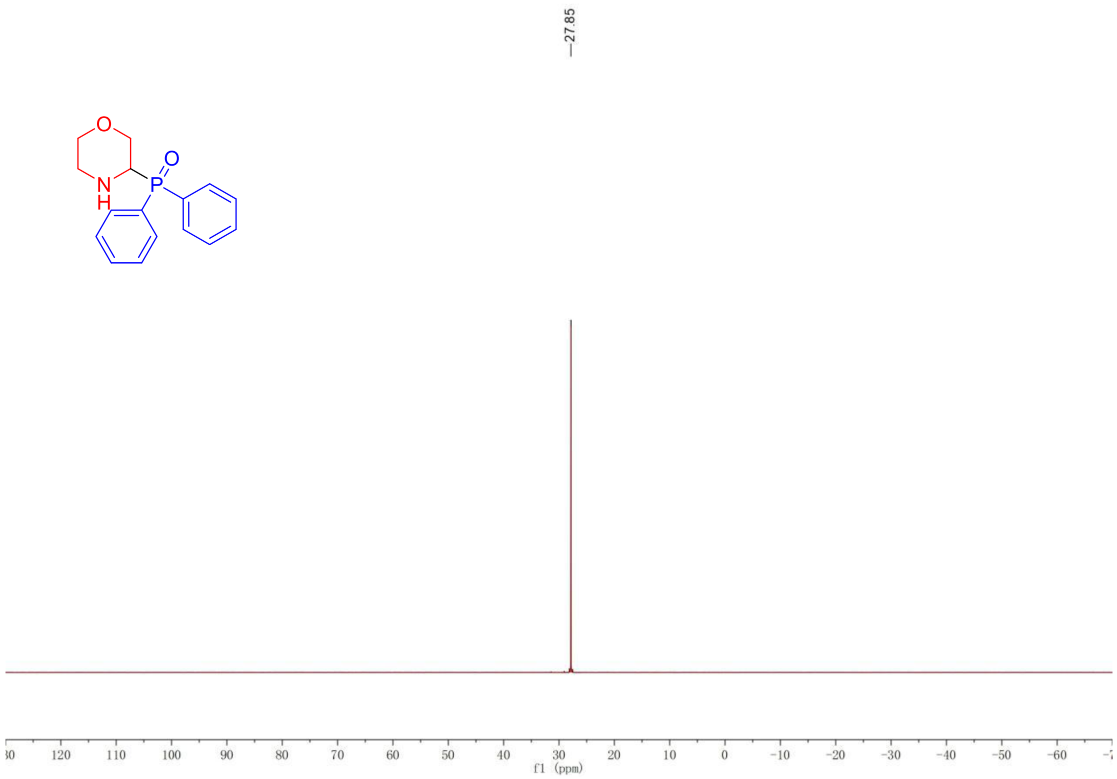

Figure S30. ^{1}H NMR spectra (400 MHz, Chloroform-*d*) of Morpholin-3-yldi-p-tolylphosphine oxide (3ab).

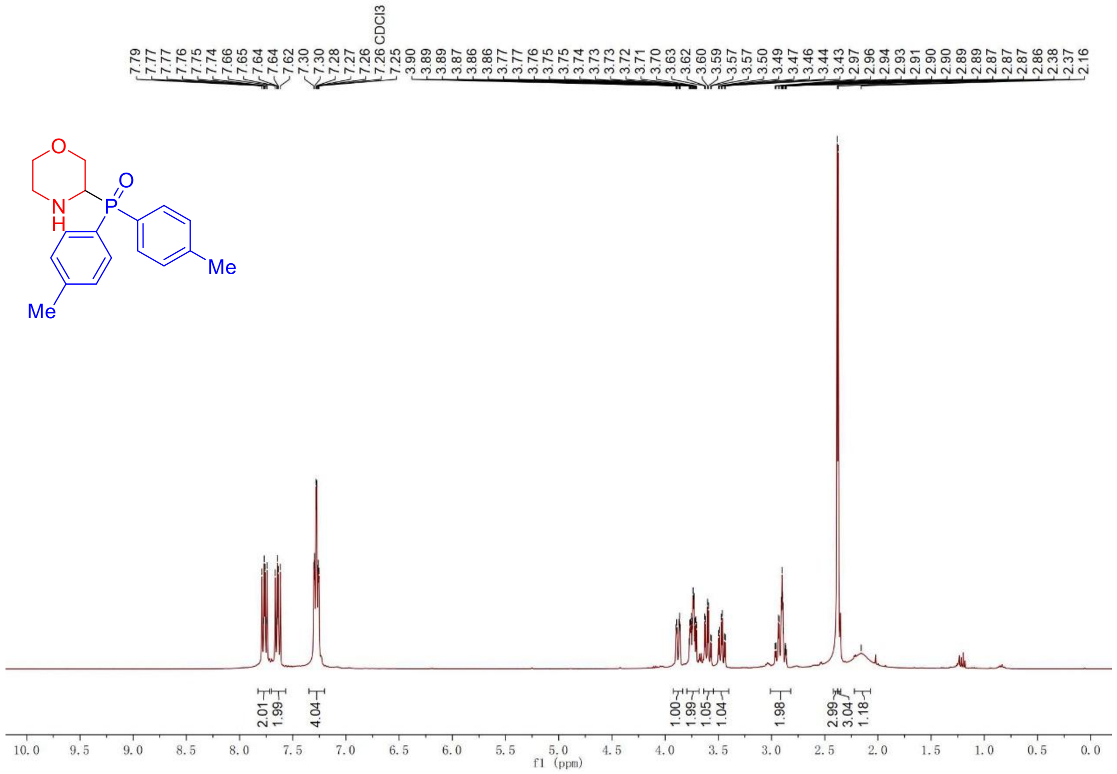

Figure S31. $^{13}C\{^{1}H\}$ **NMR spectra (100 MHz, Chloroform-*d*) of Morpholin-3-yldi-p-tolylphosphine oxide (3ab).**

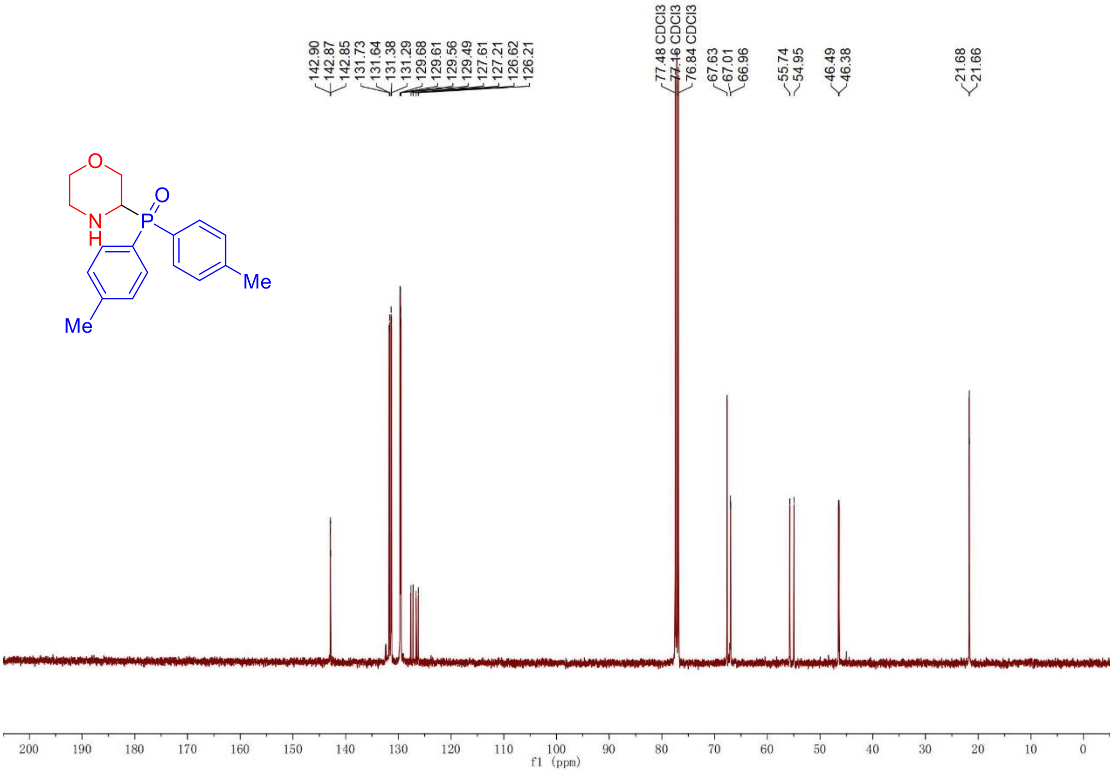

Figure S32. ^{31}P **NMR spectra (162 MHz, Chloroform-*d*) of Morpholin-3-yldi-p-tolylphosphine oxide (3ab).**

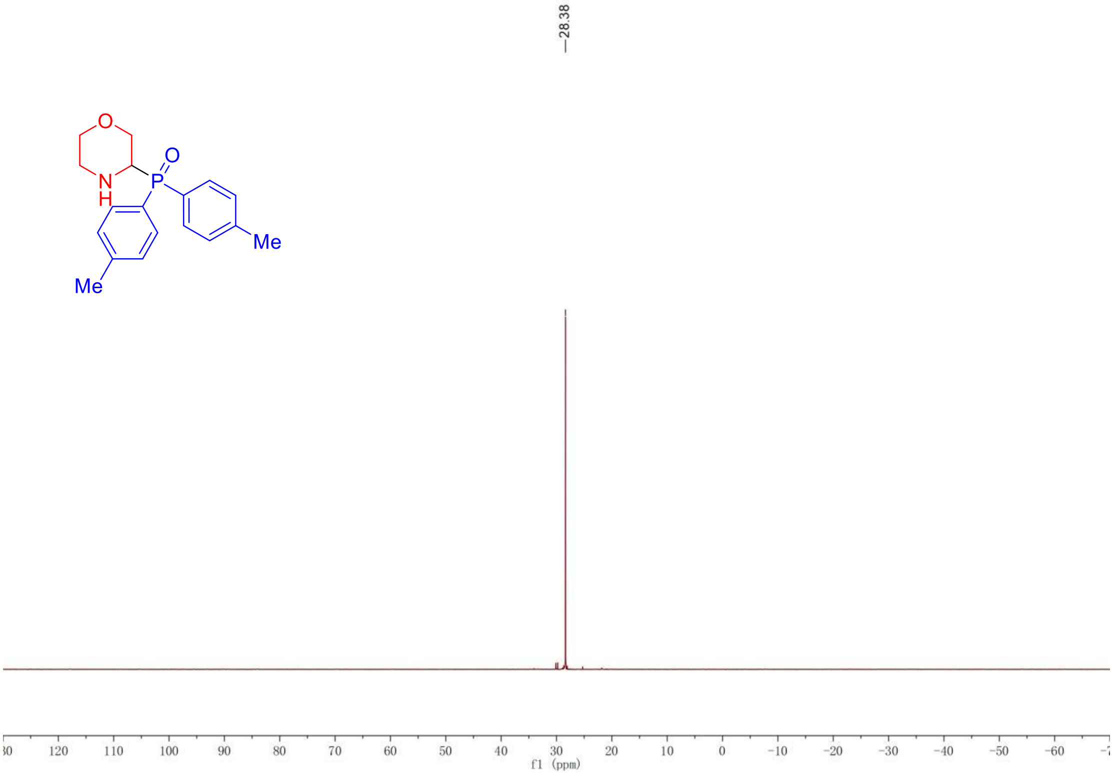

Figure S33. ^{1}**H NMR spectra (400 MHz, Chloroform-*d*) of Bis(4-(*tert*-butyl)phenyl)(morpholin-3-yl)phosphine oxide (3ac).**

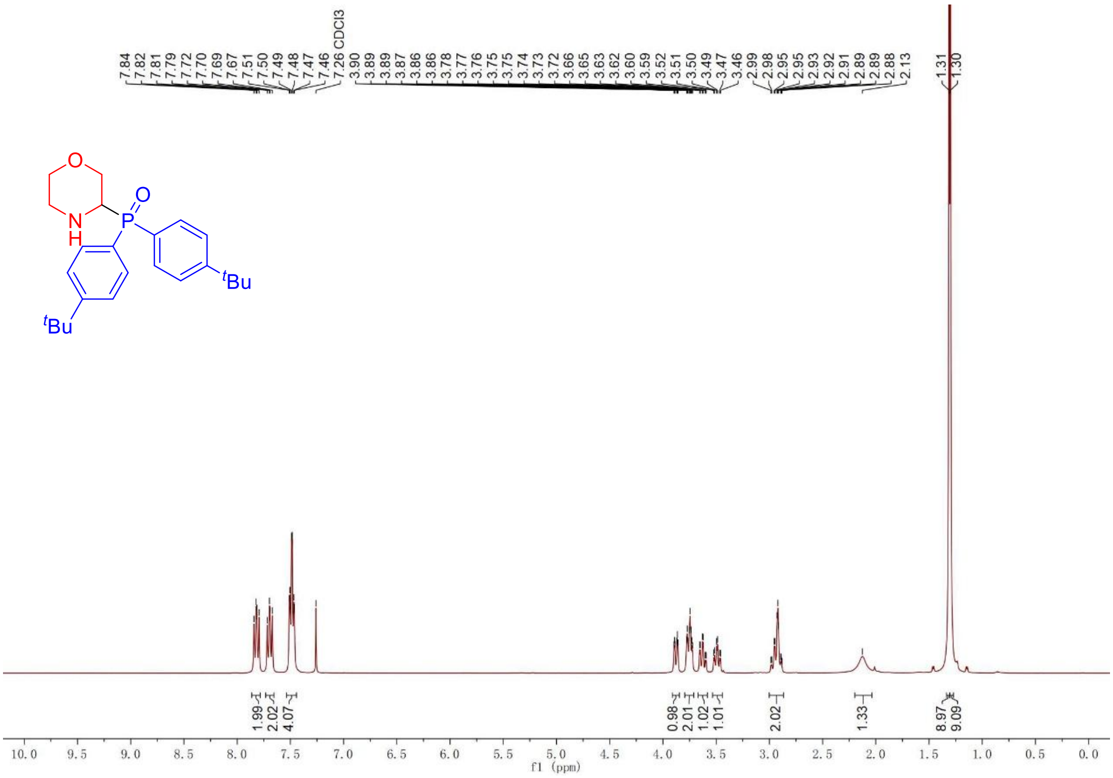

Figure S34. $^{13}C\{^{1}H\}$ **NMR spectra (100 MHz, Chloroform-*d*) of Bis(4-(*tert*-butyl)phenyl)(morpholin-3-yl)phosphine oxide (3ac).**

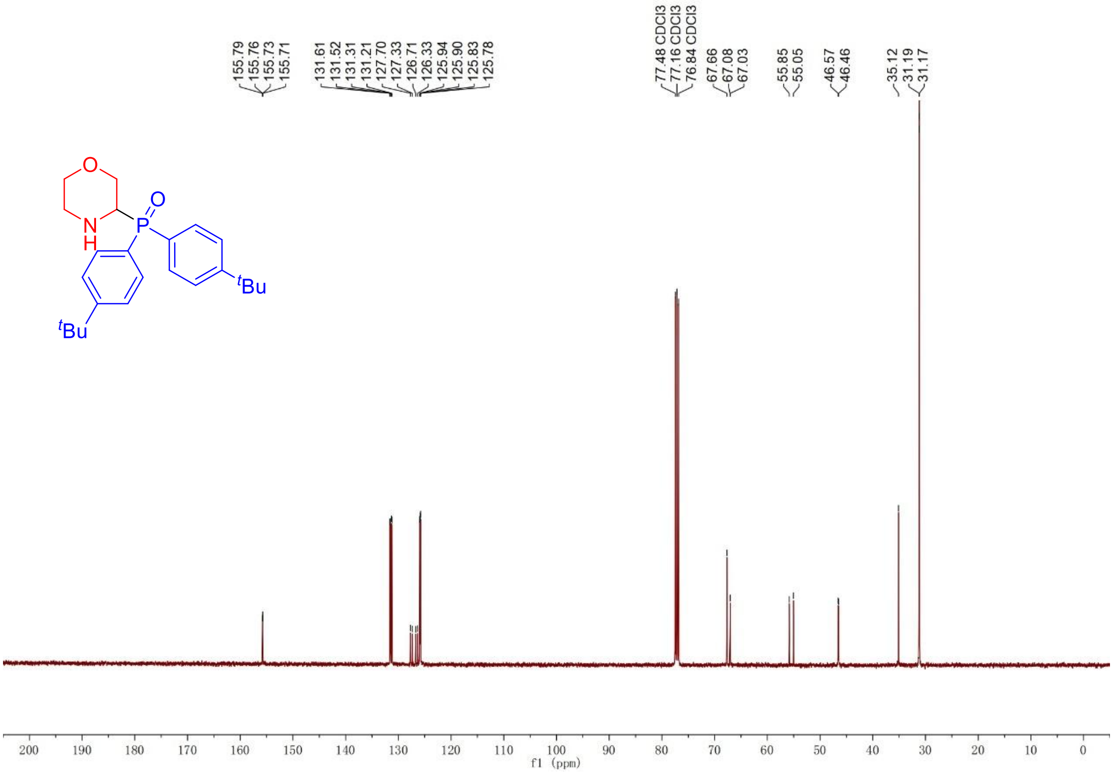

Figure S35. ^{31}**P NMR spectra (162 MHz, Chloroform-*d*) of Bis(4-(*tert*-butyl)phenyl)(morpholin-3-yl)phosphine oxide (3ac).**

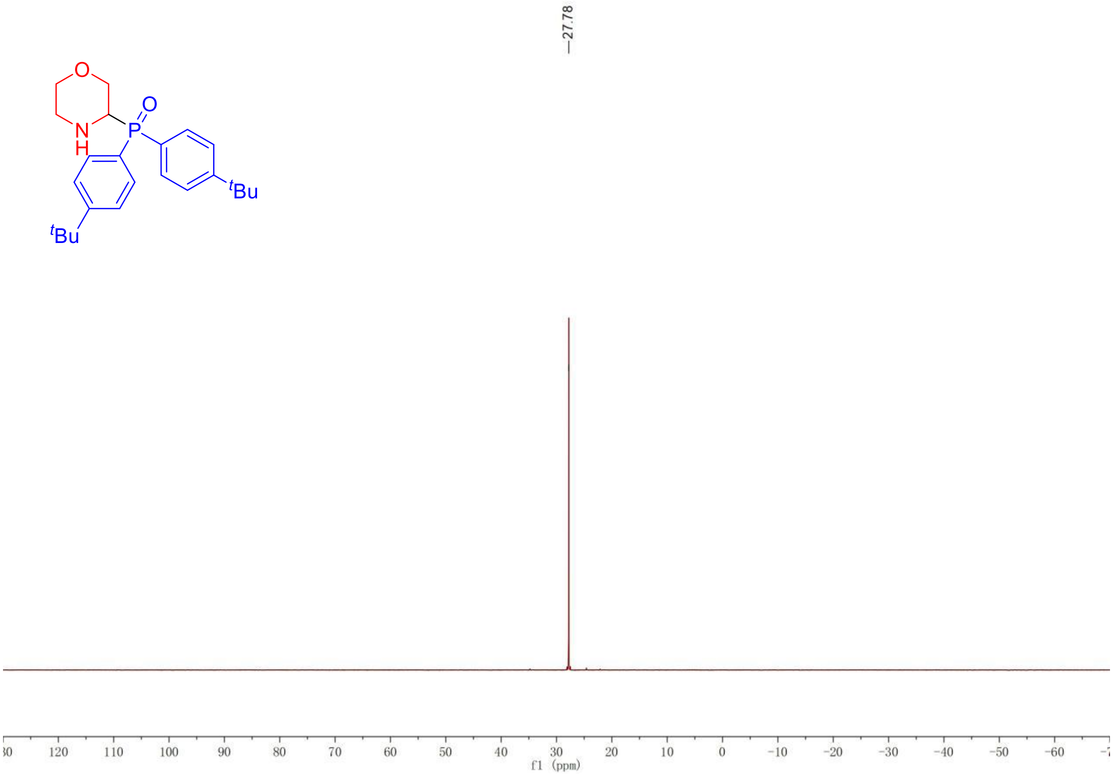

Figure S36. ^{1}**H NMR spectra (400 MHz, Chloroform-*d*) of Di([1,1'-biphenyl]-4-yl)(morpholin-3-yl)phosphine oxide (3ad).**

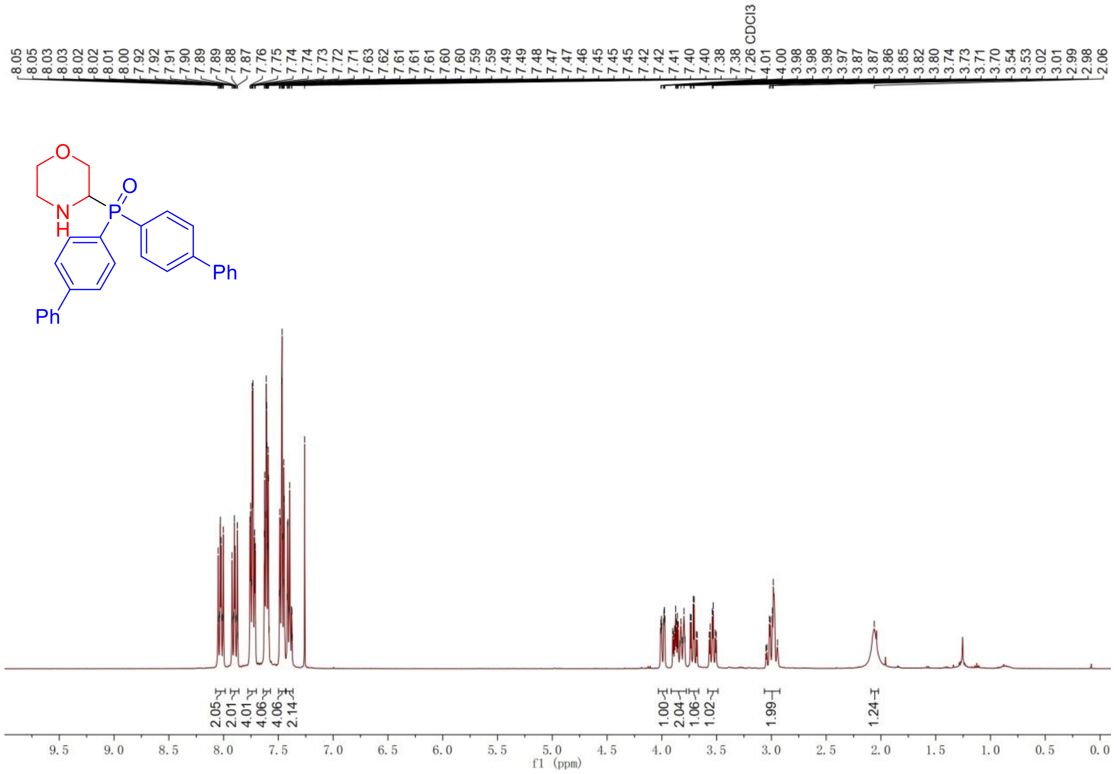

Figure S37. $^{13}C\{^{1}H\}$ **NMR spectra (100 MHz, Chloroform-*d*) of Di([1,1'-biphenyl]-4-yl)(morpholin-3-yl)phosphine oxide (3ad).**

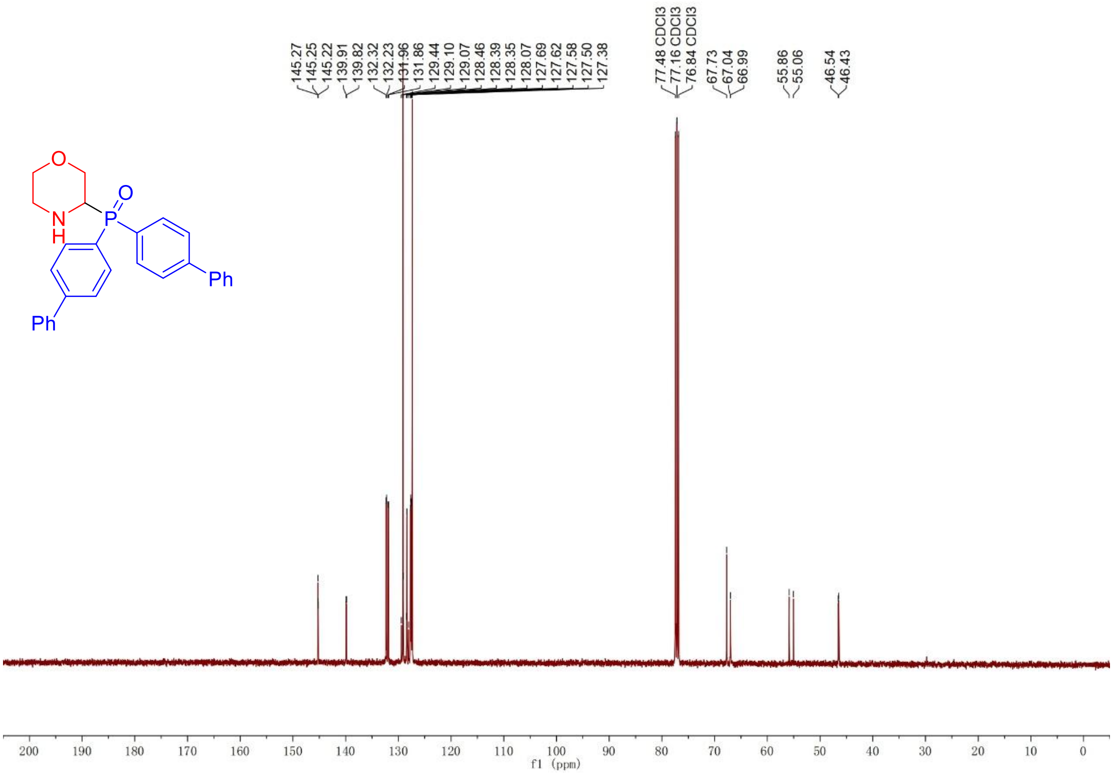

Figure S38. ^{31}P **NMR spectra (162 MHz, Chloroform-*d*) of Di([1,1'-biphenyl]-4-yl)(morpholin-3-yl)phosphine oxide (3ad).**

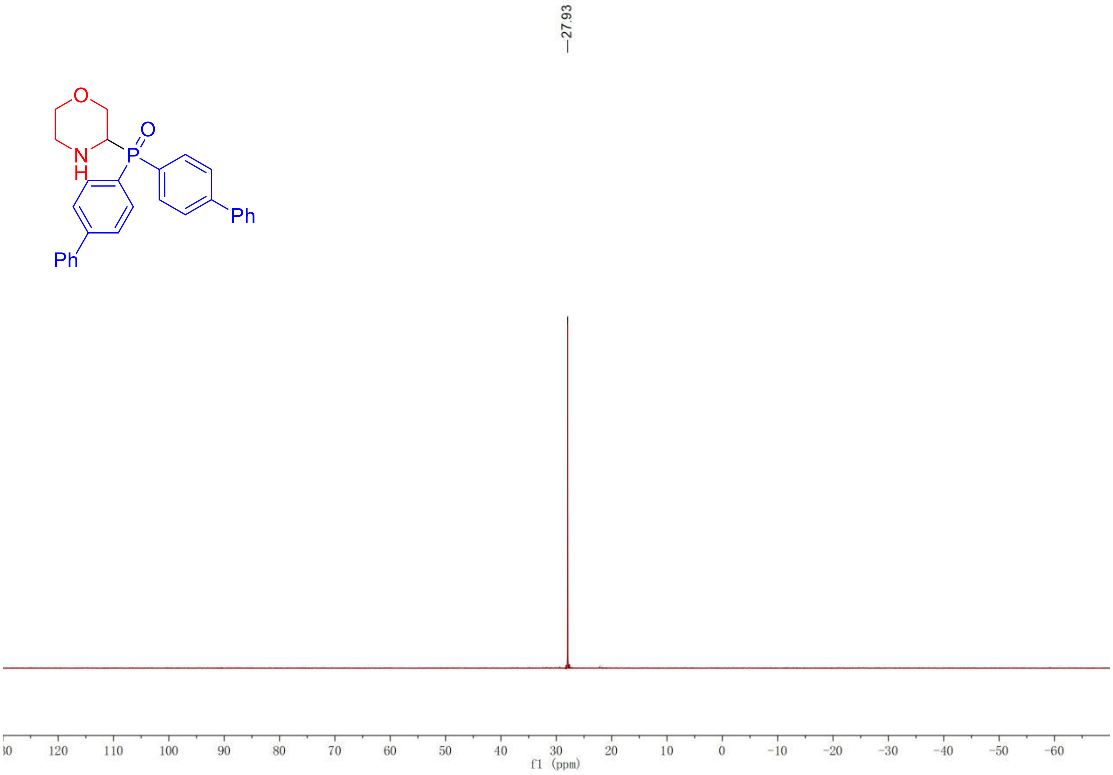

Figure S39. ^{1}H NMR spectra (400 MHz, Chloroform-*d*) of Bis(4-methoxyphenyl)(morpholin-3-yl)phosphine oxide (3ae).

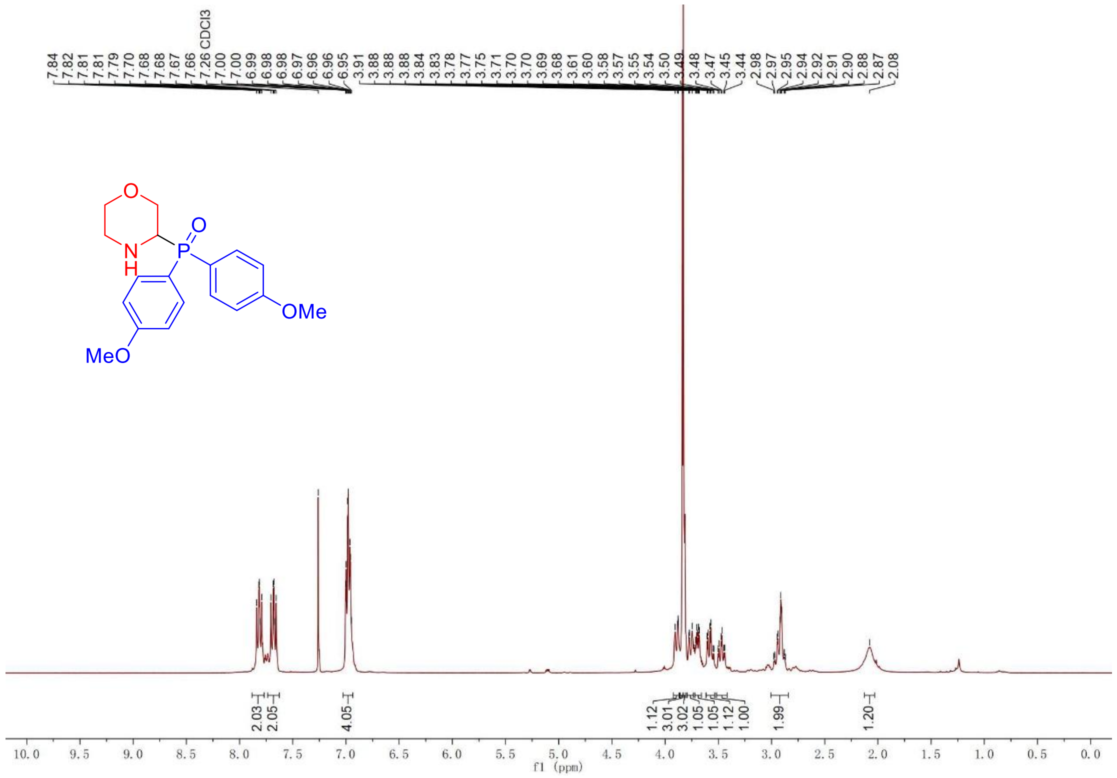

Figure S40. ^{13}C{^{1}H} NMR spectra (100 MHz, Chloroform-*d*) of Bis(4-methoxyphenyl)(morpholin-3-yl)phosphine oxide (3ae).

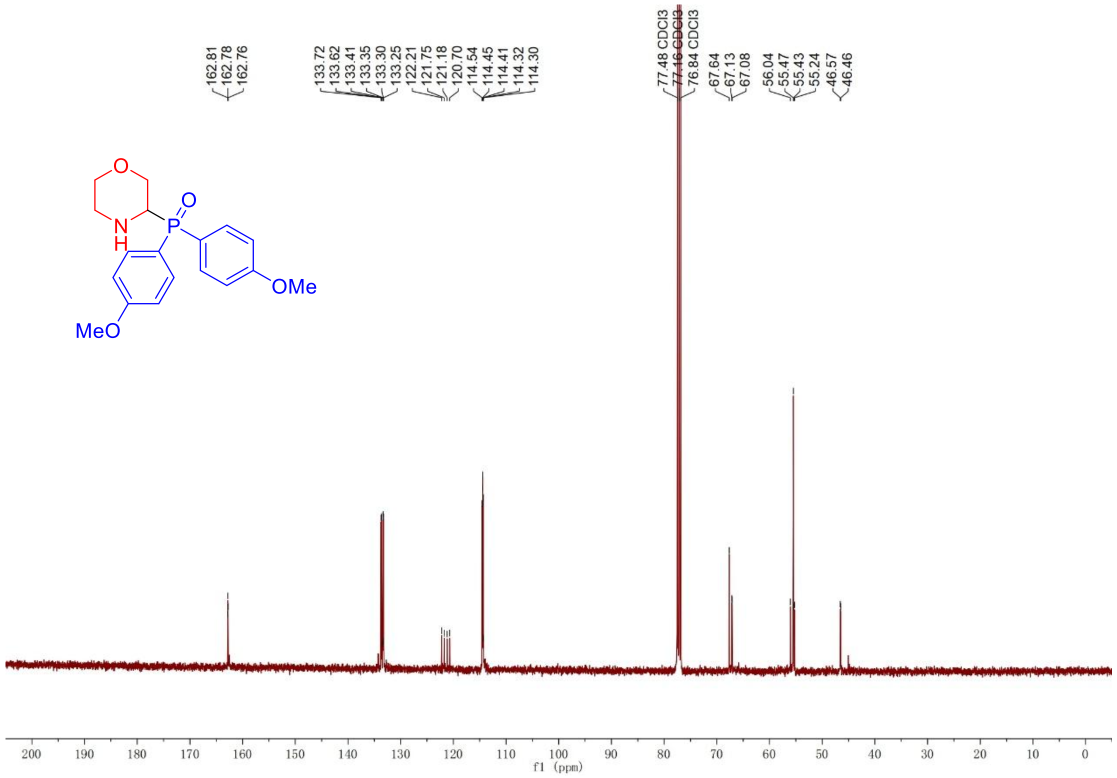

Figure S41. ^{31}**P NMR spectra (162 MHz, Chloroform-*d*) of Bis(4-methoxyphenyl)(morpholin-3-yl)phosphine oxide (3ae).**

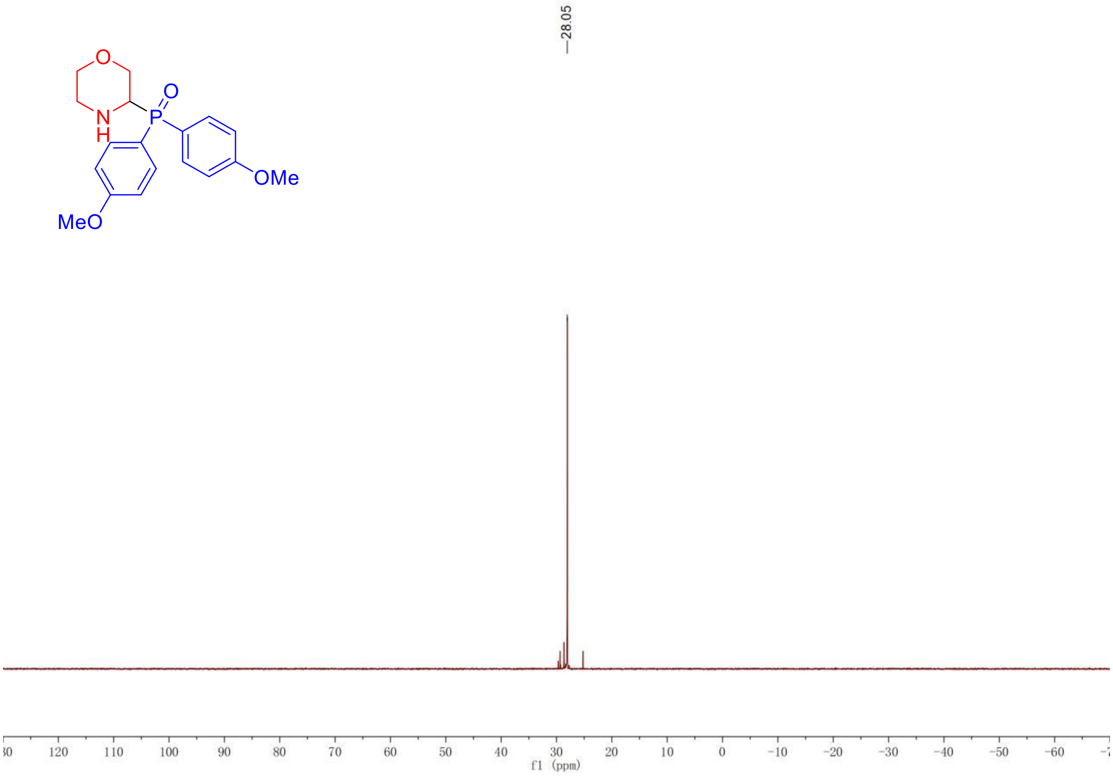

Figure S42. ^{1}**H NMR spectra (400 MHz, Chloroform-*d*) of Bis(4-(methylthio)phenyl)(morpholin-3-yl)phosphine oxide (3af).**

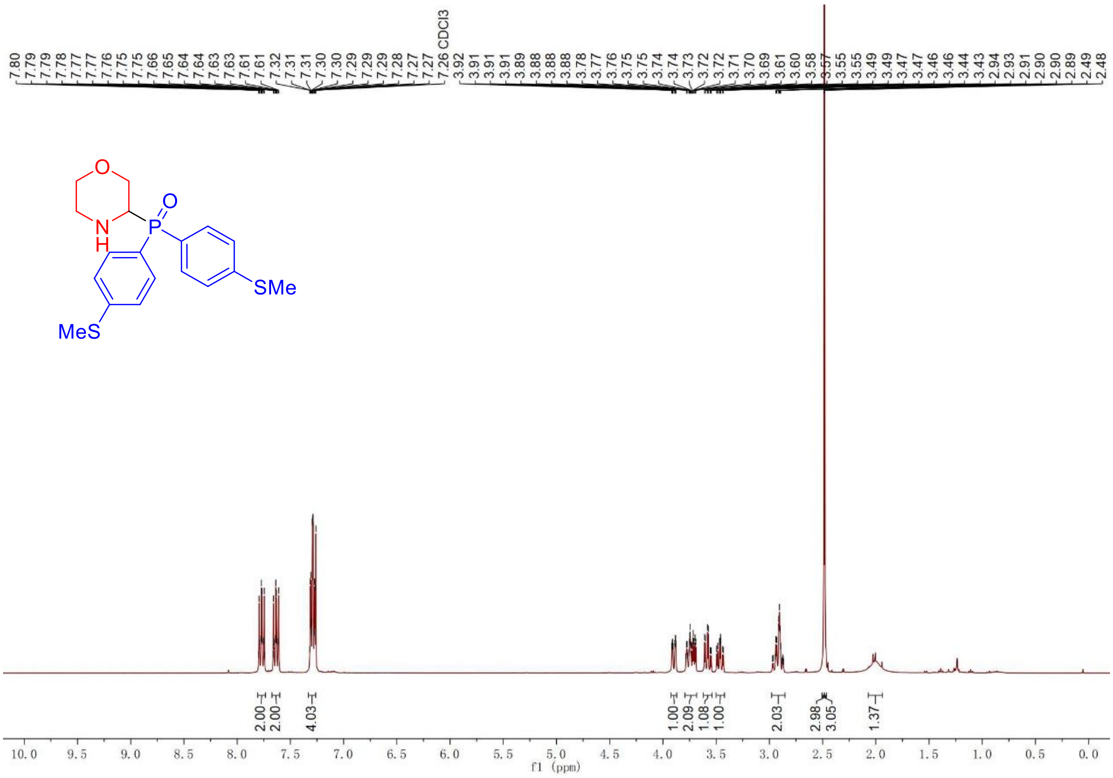

Figure S43. $^{13}C\{^{1}H\}$ **NMR spectra (100 MHz, Chloroform-*d*) of Bis(4-(methylthio)phenyl)(morpholin-3-yl)phosphine oxide (3af).**

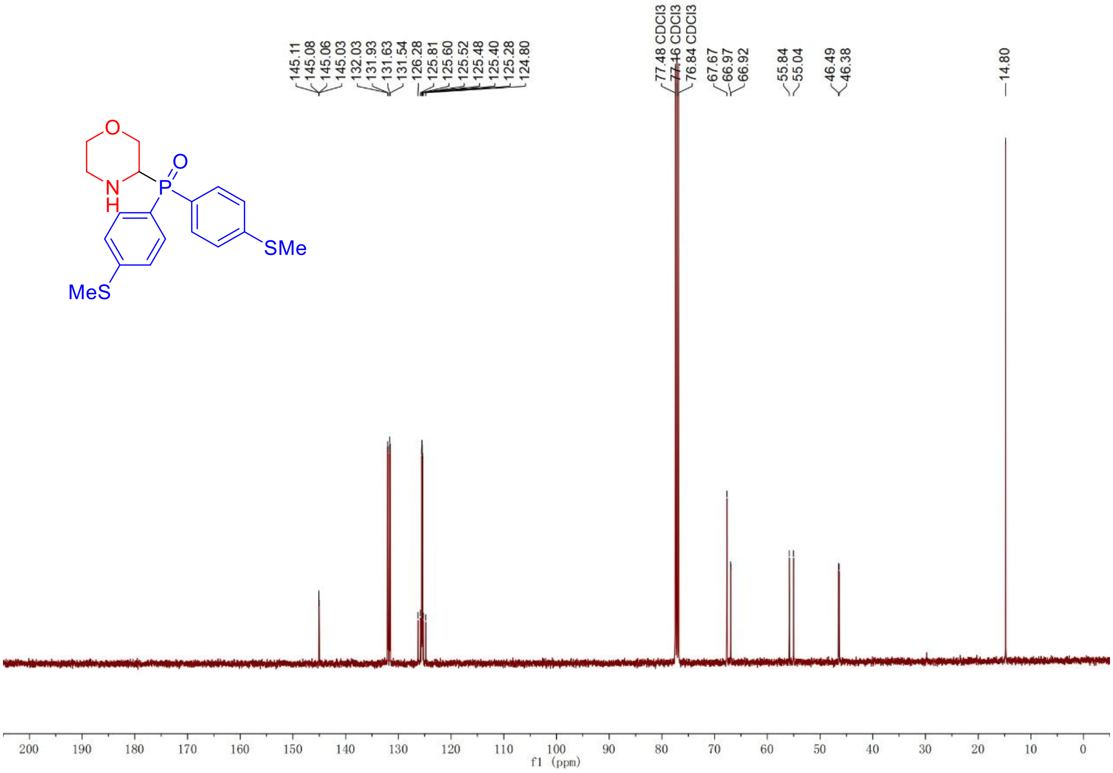

Figure S44. ^{31}P **NMR spectra (162 MHz, Chloroform-*d*) of Bis(4-(methylthio)phenyl)(morpholin-3-yl)phosphine oxide (3af).**

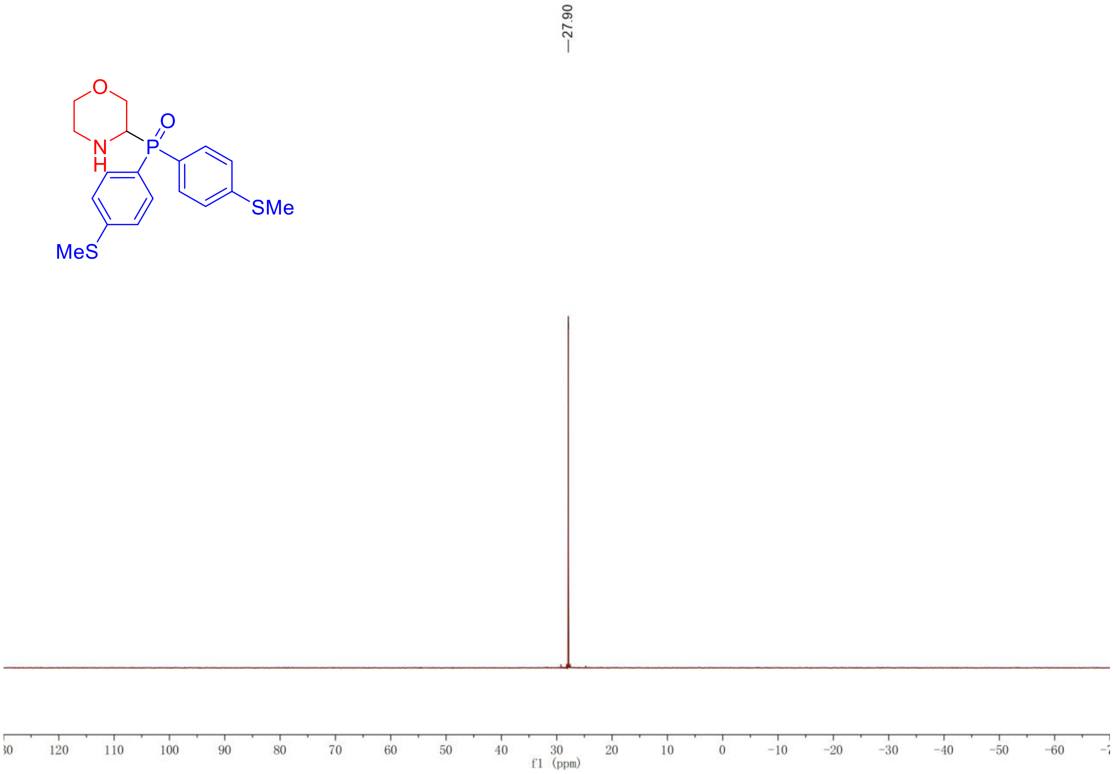

Figure S45. ^{1}H **NMR spectra (400 MHz, Chloroform-*d*) of Bis(4-fluorophenyl)(morpholin-3-yl)phosphine oxide (3ag).**

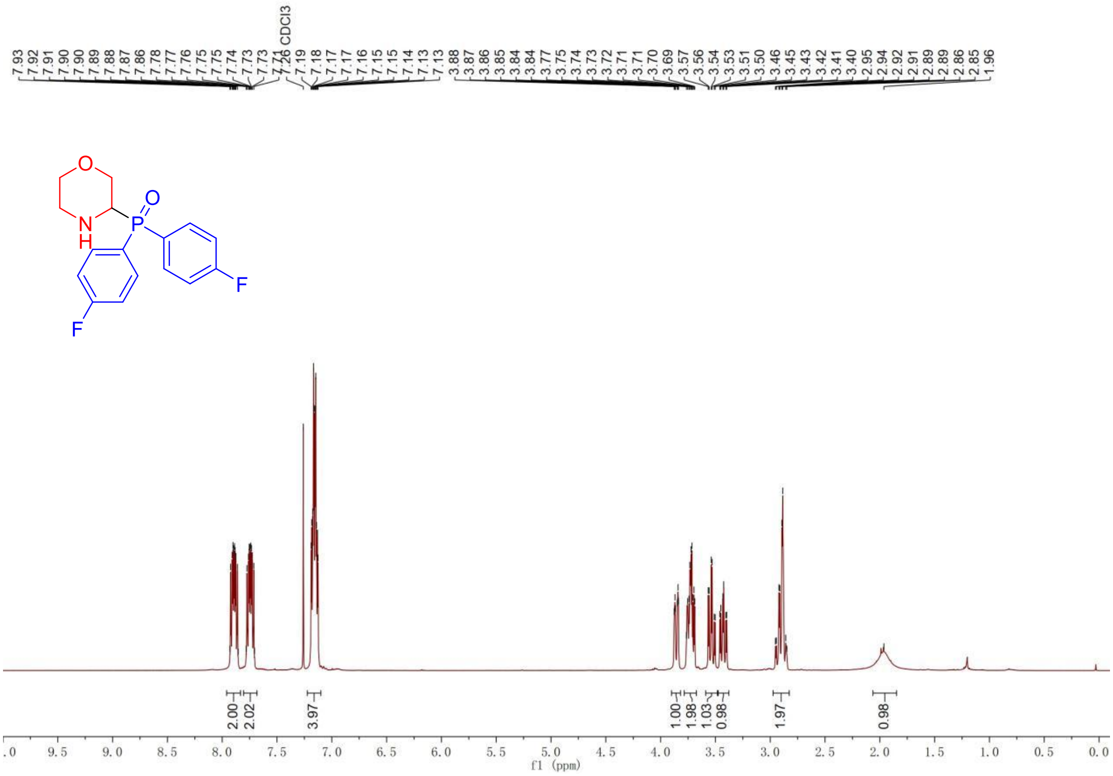

Figure S46. $^{13}C\{^{1}H\}$ **NMR spectra (100 MHz, Chloroform-*d*) of Bis(4-fluorophenyl)(morpholin-3-yl)phosphine oxide (3ag).**

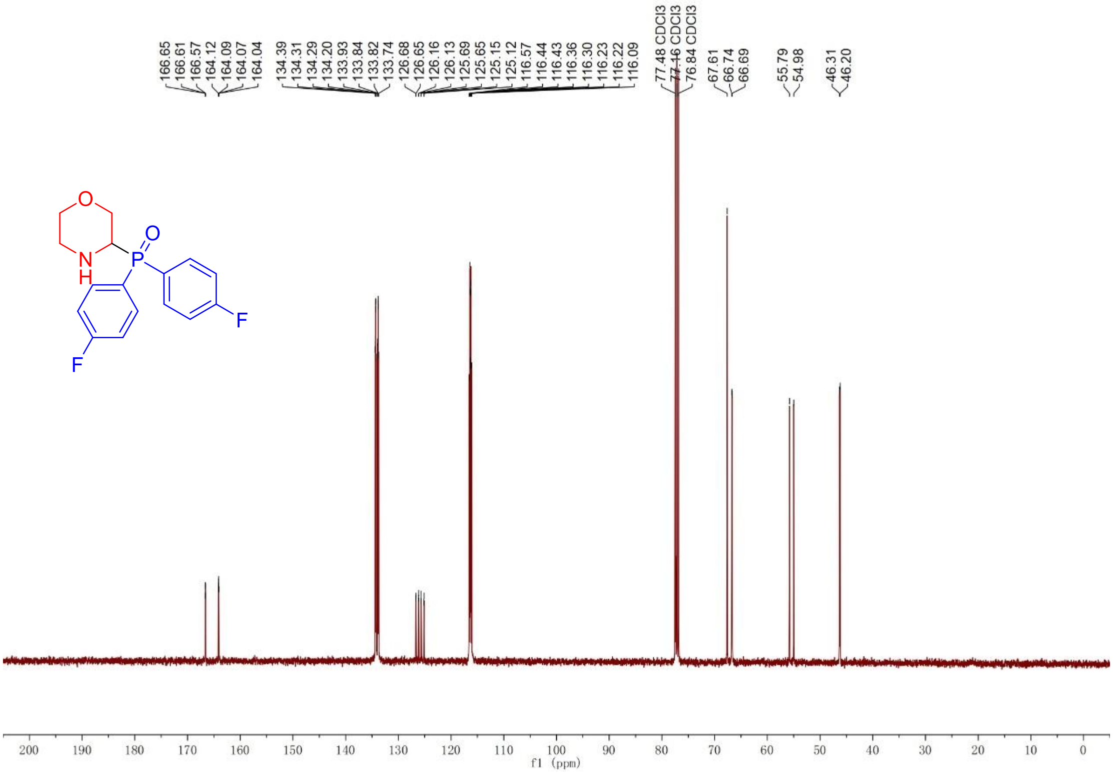

Figure S47. ^{31}P NMR spectra (162 MHz, Chloroform-*d*) of Bis(4-fluorophenyl)(morpholin-3-yl)phosphine oxide (3ag).

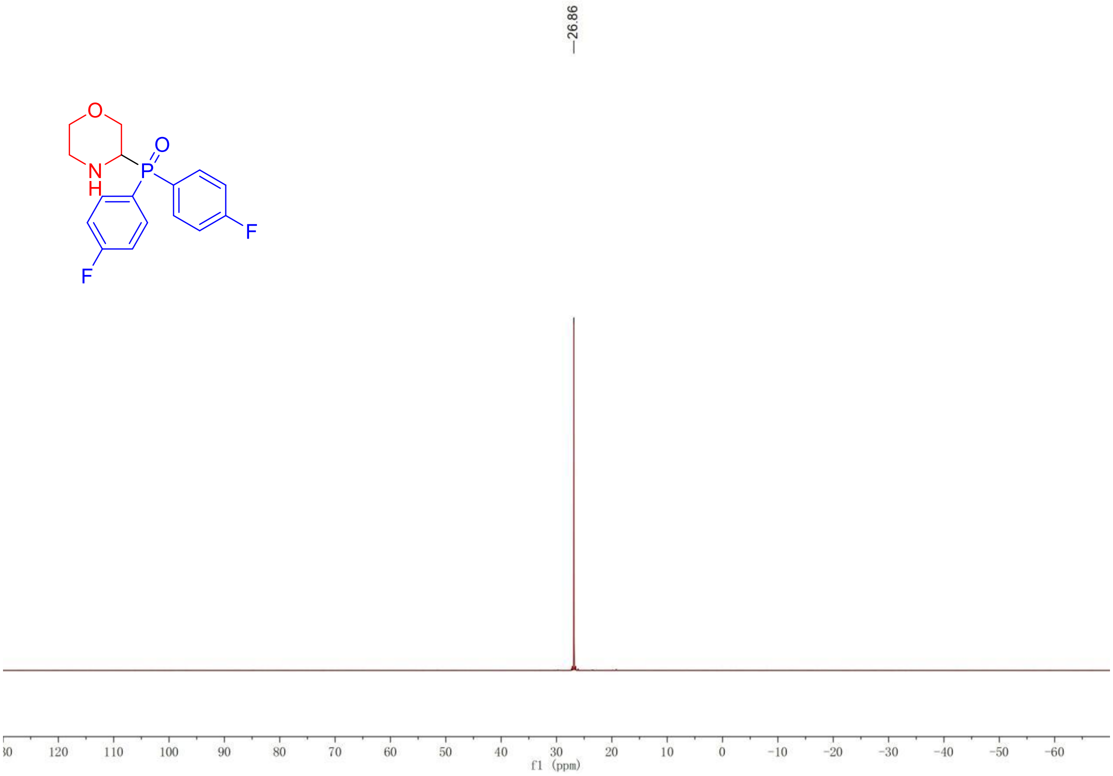

Figure S48. ^{19}F NMR spectra (376 MHz, Chloroform-*d*) of Bis(4-fluorophenyl)(morpholin-3-yl)phosphine oxide (3ag).

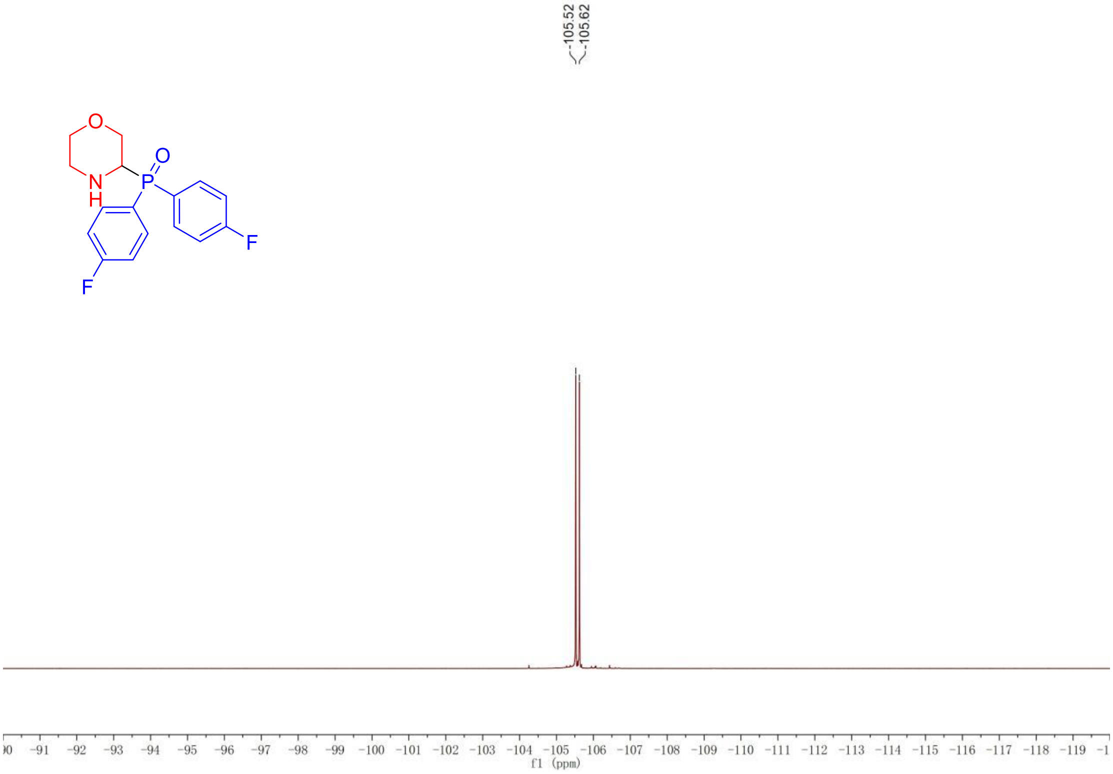

Figure S49. ^{1}H **NMR spectra (400 MHz, Chloroform-*d*) of Bis(4-chlorophenyl)(morpholin-3-yl)phosphine oxide (3ah).**

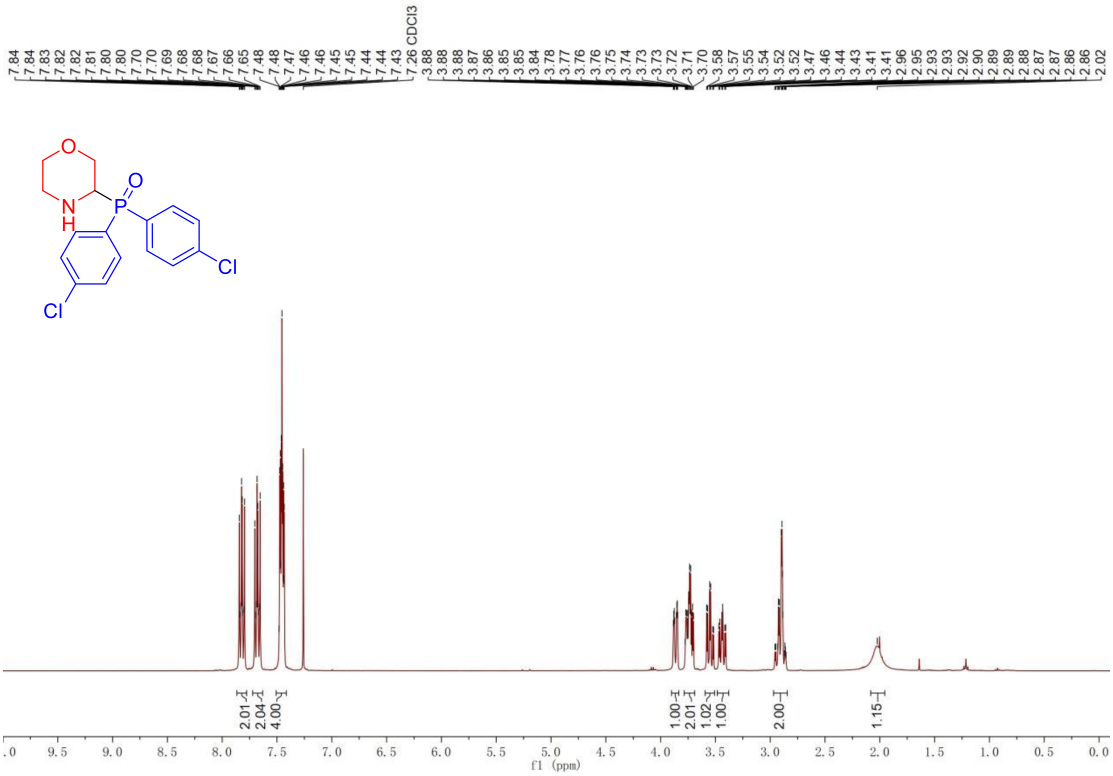

Figure S50. $^{13}C\{^{1}H\}$ **NMR spectra (100 MHz, Chloroform-*d*) of Bis(4-chlorophenyl)(morpholin-3-yl)phosphine oxide (3ah).**

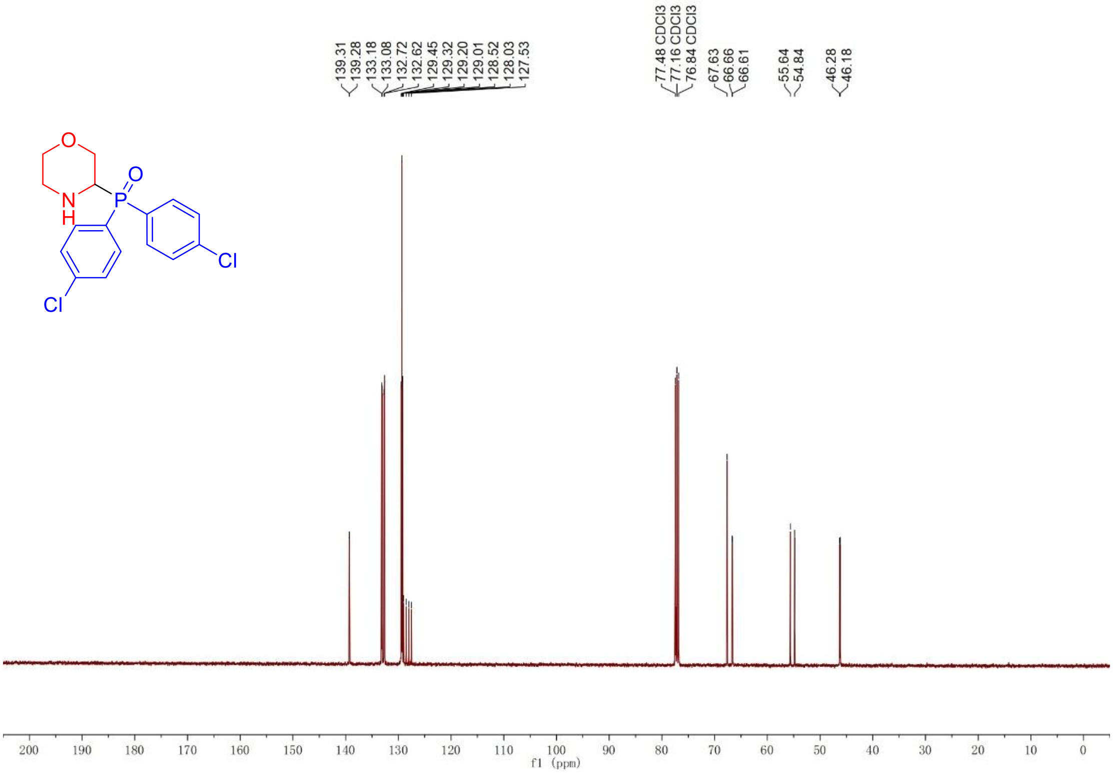

Figure S51. ^{31}P **NMR spectra (162 MHz, Chloroform-*d*) of Bis(4-chlorophenyl)(morpholin-3-yl)phosphine oxide (3ah).**

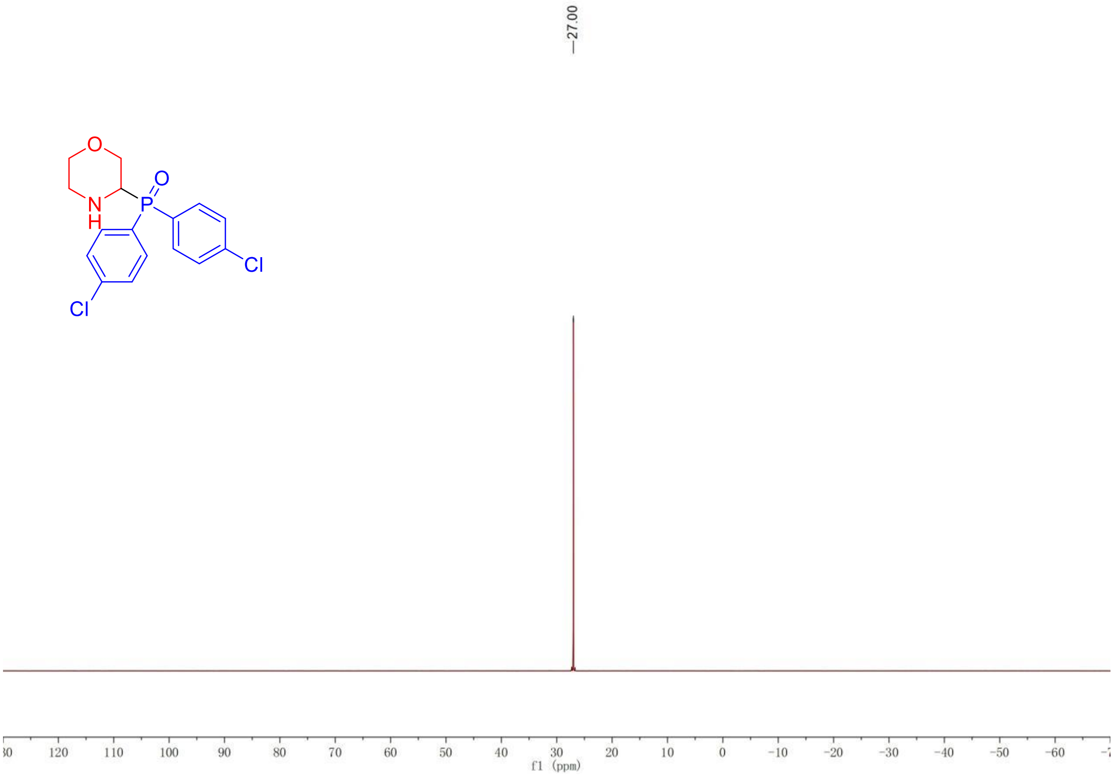

Figure S52. ^{1}H **NMR spectra (400 MHz, Chloroform-*d*) of Morpholin-3-ylbis(4-(trifluoromethyl)phenyl)phosphine oxide (3ai).**

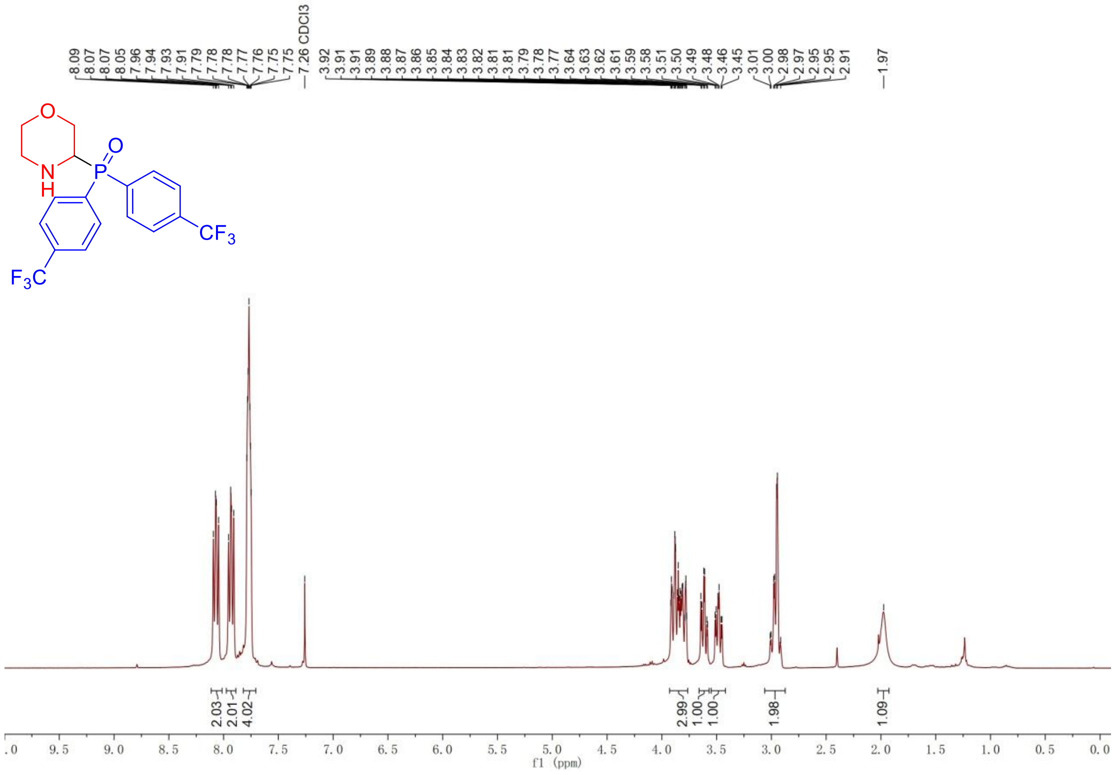

Figure S53. $^{13}C\{^1H\}$ **NMR spectra (100 MHz, Chloroform-*d*) of Morpholin-3-ylbis(4-(trifluoromethyl)phenyl)phosphine oxide (3ai).**

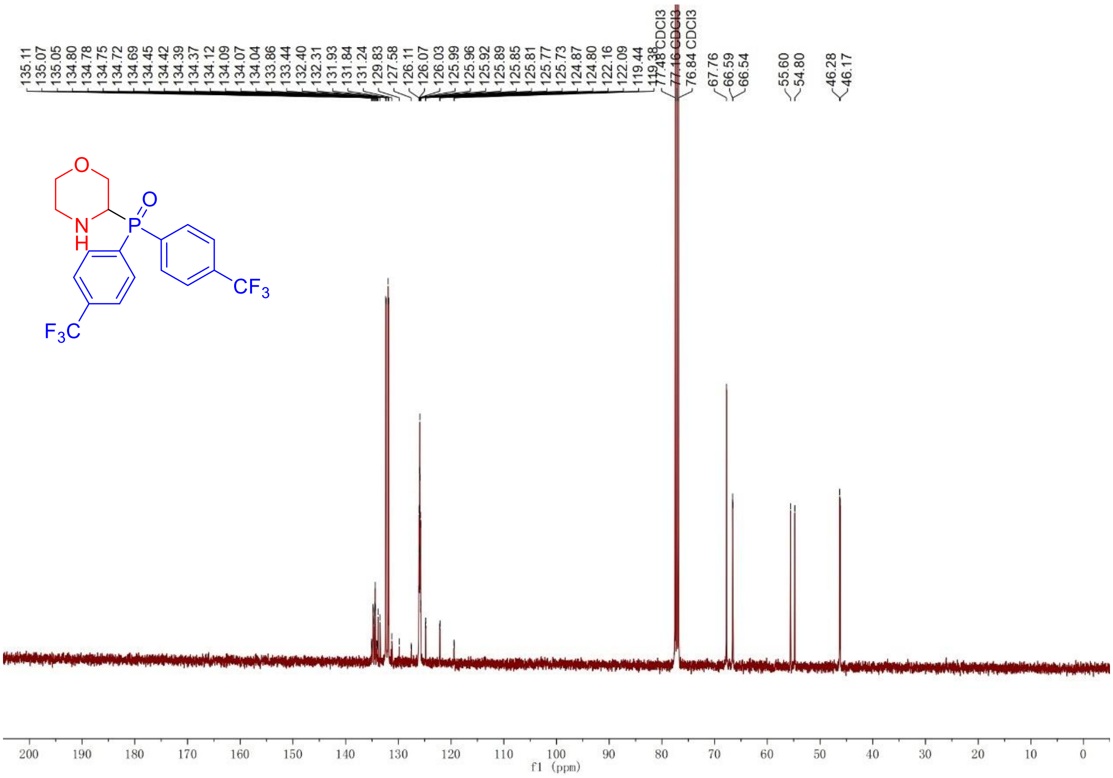

Figure S54. ^{31}P **NMR spectra (162 MHz, Chloroform-*d*) of Morpholin-3-ylbis(4-(trifluoromethyl)phenyl)phosphine oxide (3ai).**

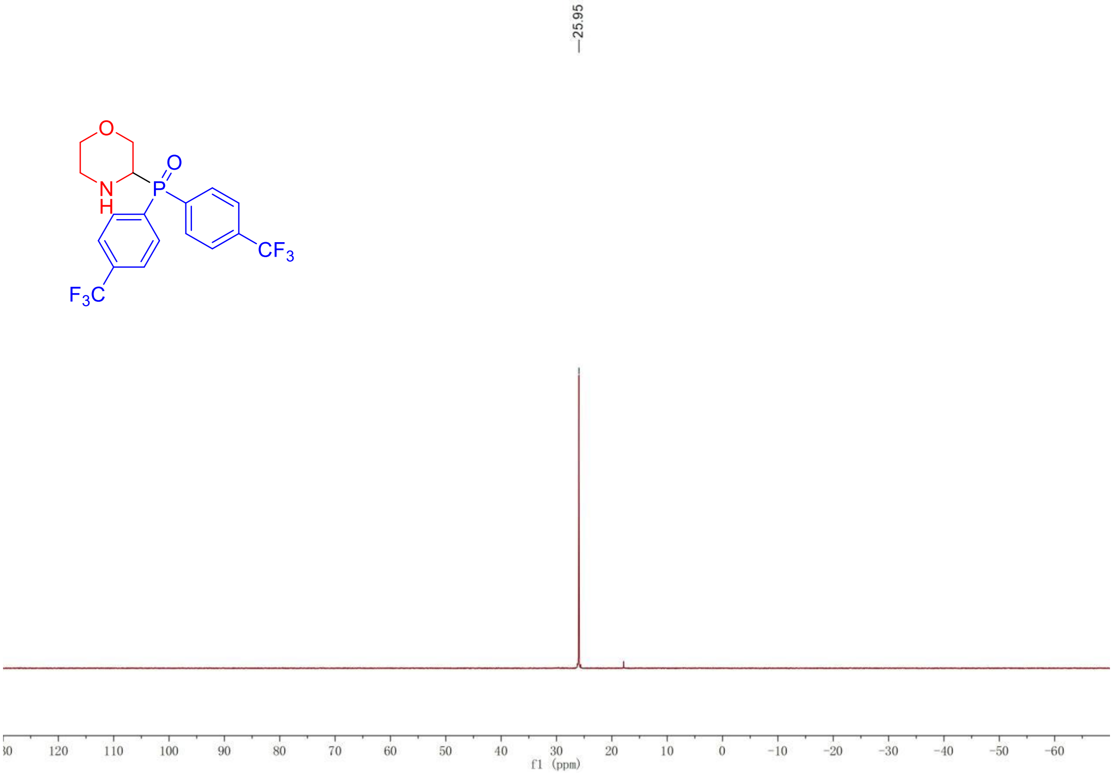

Figure S55. ^{19}**F NMR spectra (376 MHz, Chloroform-*d*) of Morpholin-3-ylbis(4-(trifluoromethyl)phenyl)phosphine oxide (3ai).**

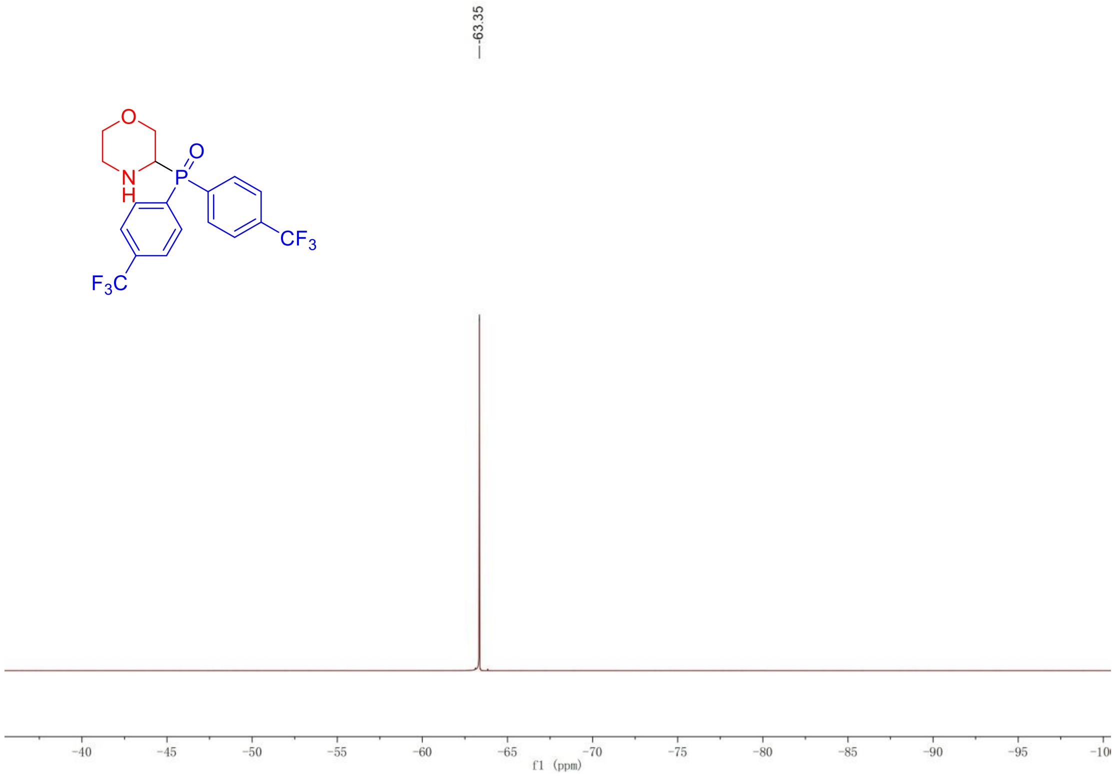

Figure S56. ^{1}**H NMR spectra (400 MHz, Chloroform-*d*) of Morpholin-3-yldi-*m*-tolylphosphine oxide (3aj).**

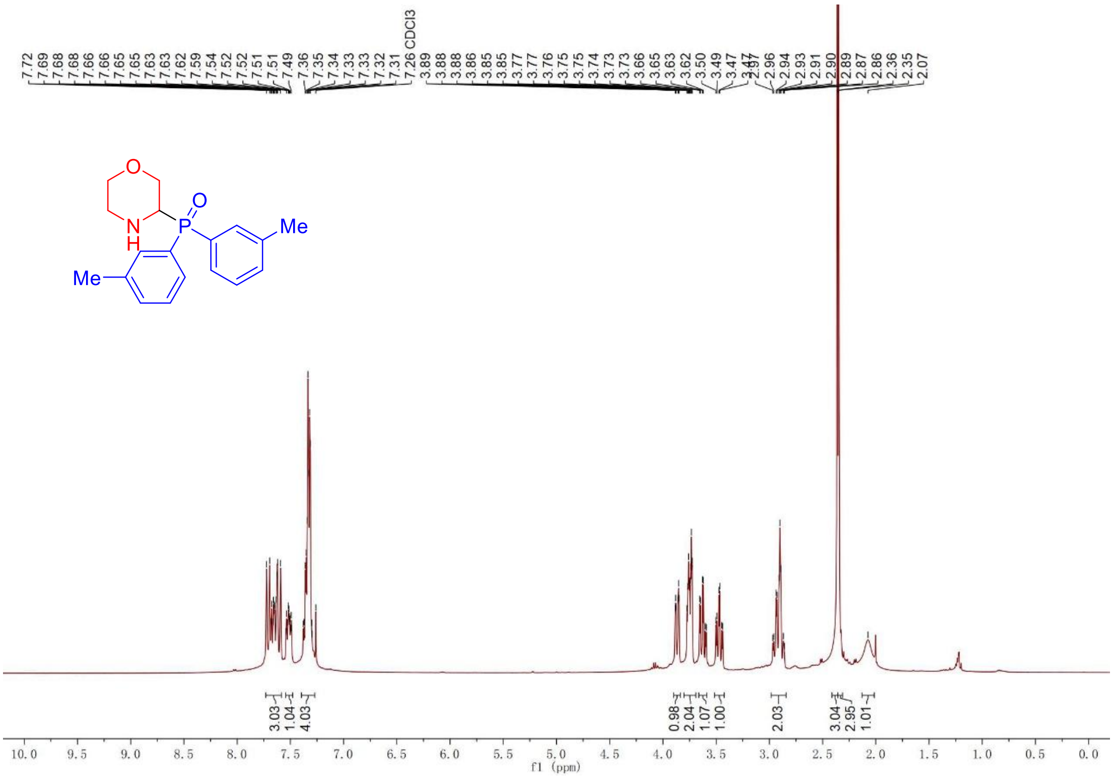

Figure S57. $^{13}C\{^{1}H\}$ **NMR spectra (100 MHz, Chloroform-*d*) of Morpholin-3-yldi-*m*-tolylphosphine oxide (3aj).**

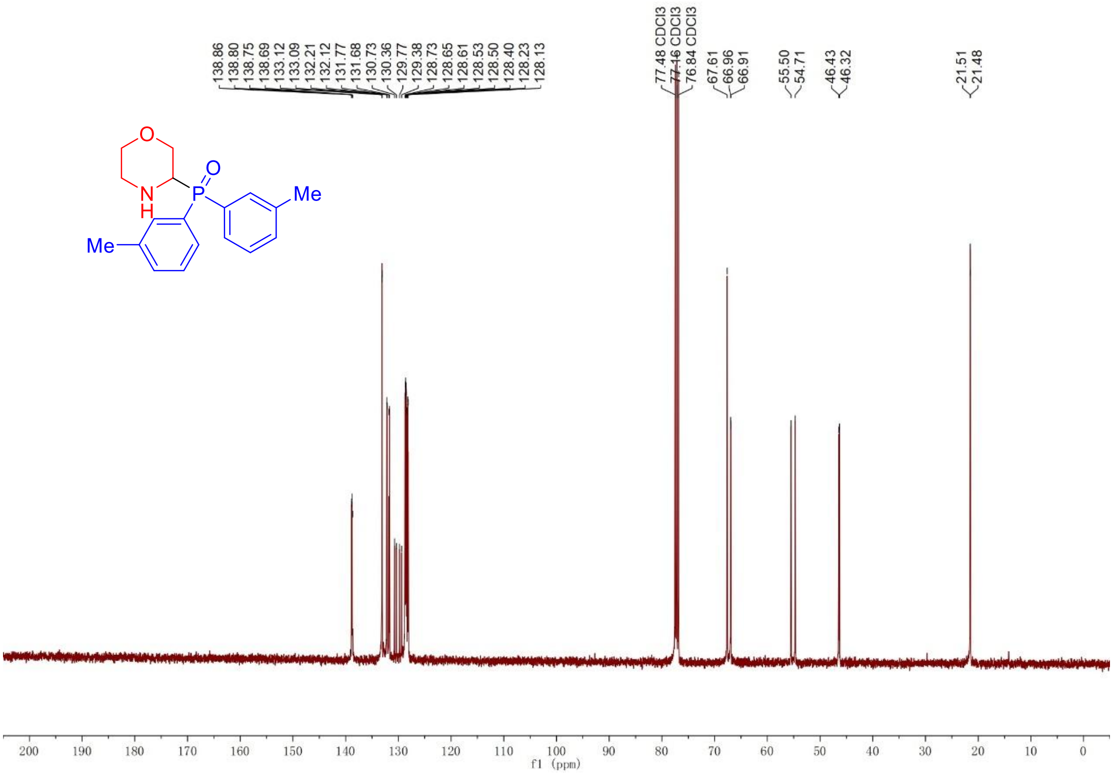

Figure S58. ^{31}P **NMR spectra (162 MHz, Chloroform-*d*) of Morpholin-3-yldi-*m*-tolylphosphine oxide (3aj).**

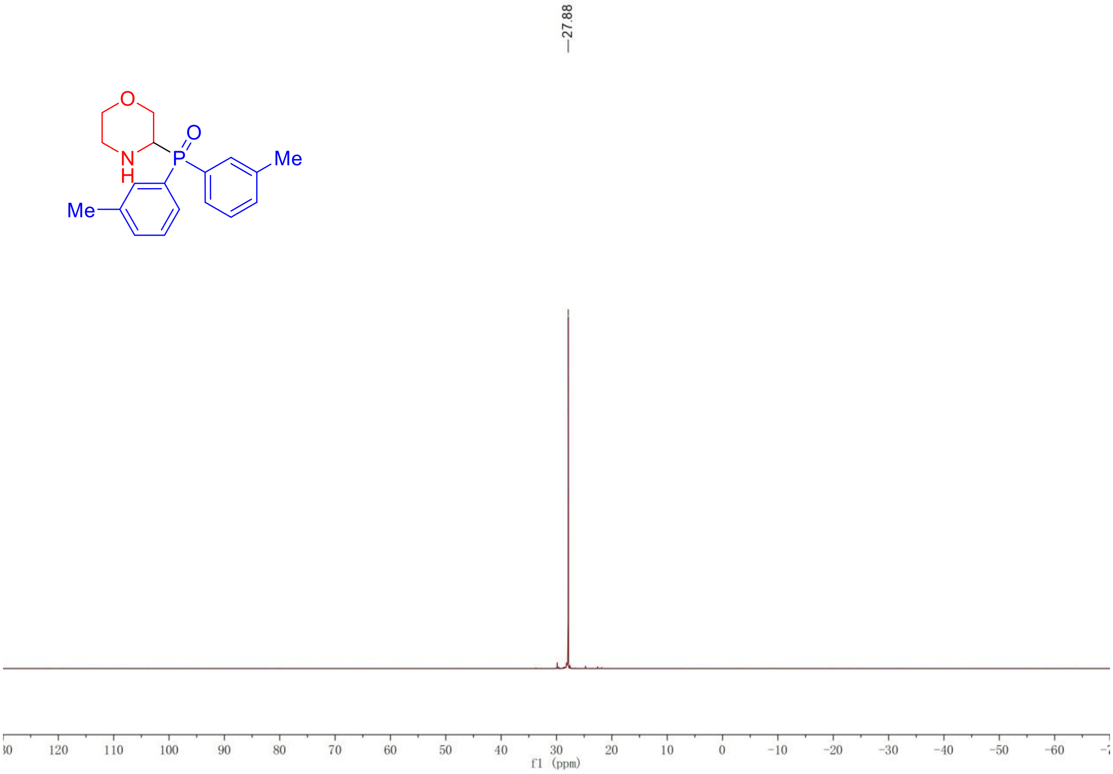

Figure S59. ^{1}H NMR spectra (400 MHz, Chloroform-*d*) of Bis(3-fluorophenyl)(morpholin-3-yl)phosphine oxide (3ak).

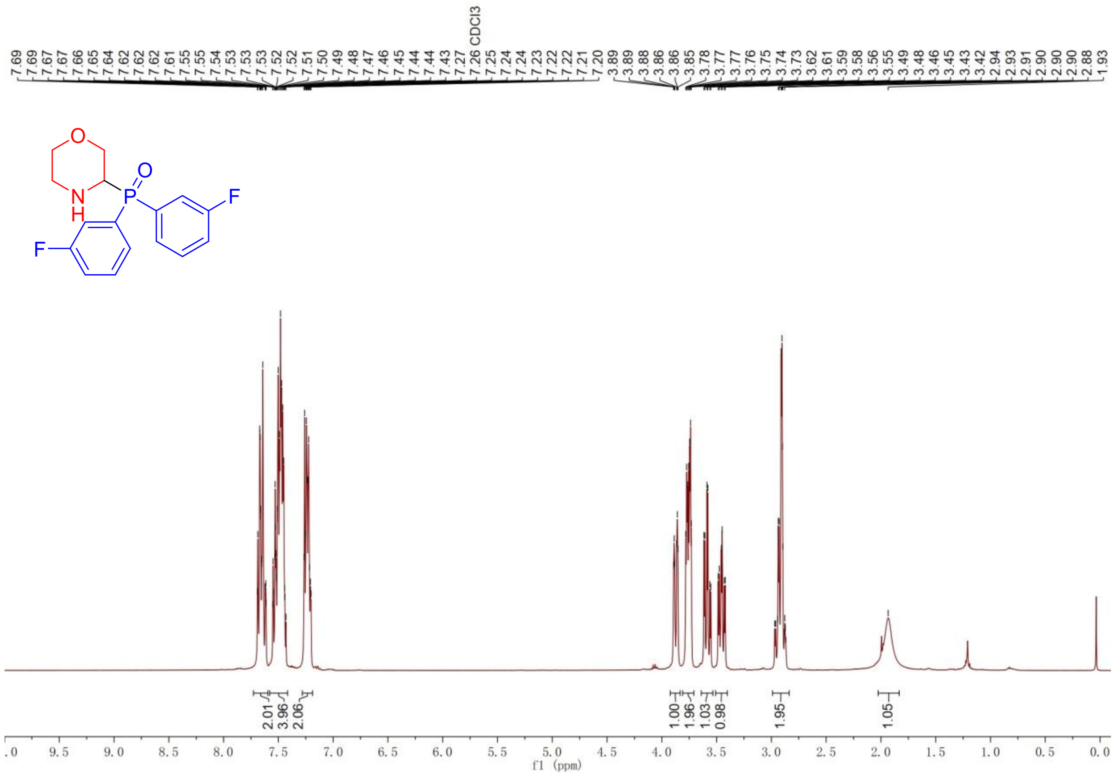

Figure S60. ^{13}C{^{1}H} NMR spectra (100 MHz, Chloroform-*d*) of Bis(3-fluorophenyl)(morpholin-3-yl)phosphine oxide (3ak).

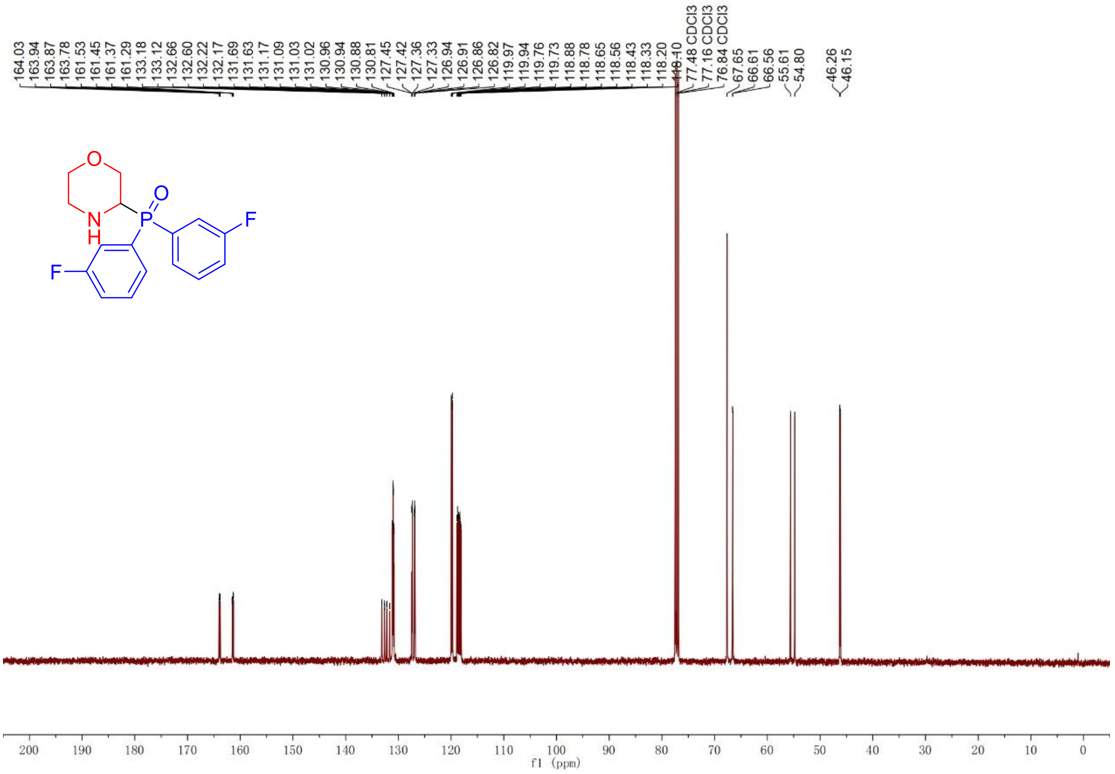

Figure S61. ^{31}P **NMR spectra (162 MHz, Chloroform-*d*) of Bis(3-fluorophenyl)(morpholin-3-yl)phosphine oxide (3ak).**

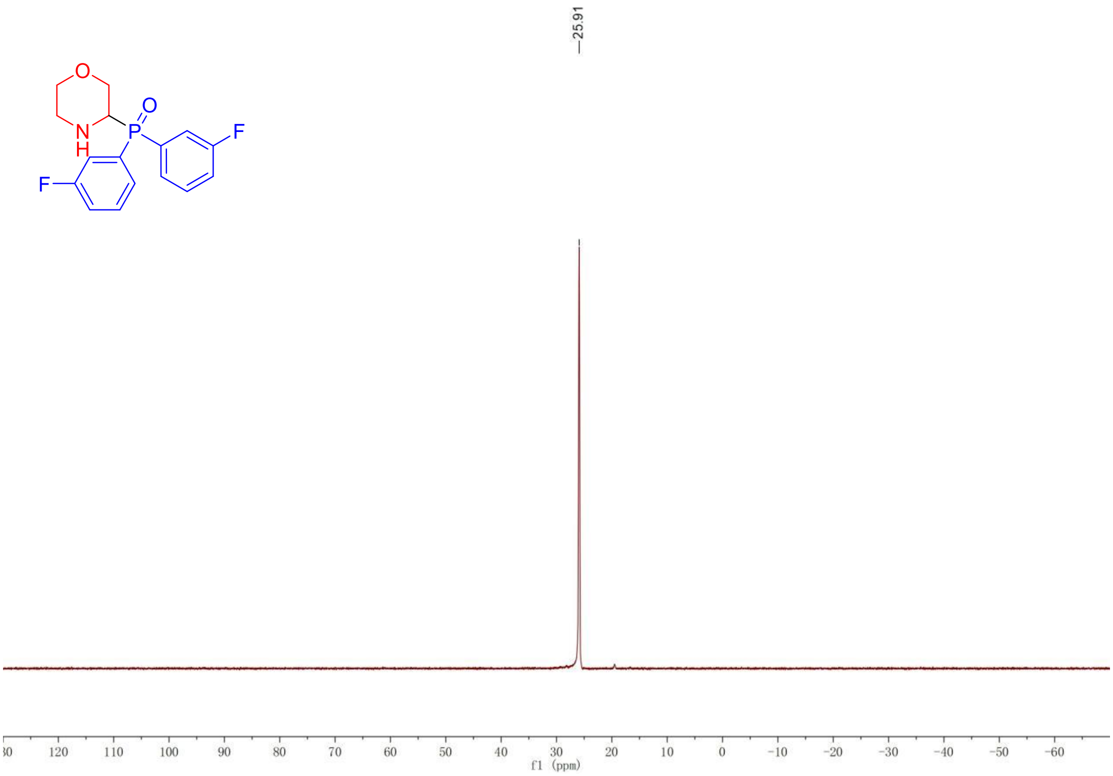

Figure S62. ^{19}F **NMR spectra (376 MHz, Chloroform-*d*) of Bis(3-fluorophenyl)(morpholin-3-yl)phosphine oxide (3ak).**

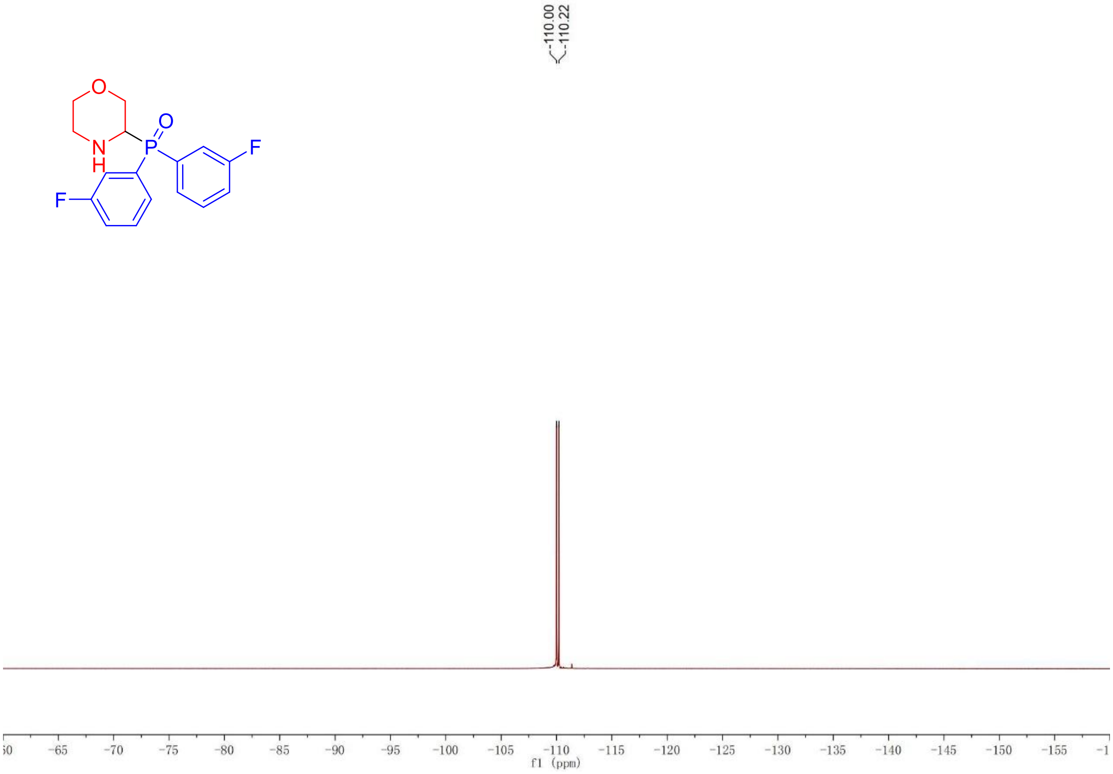

Figure S63. ^{1}H **NMR spectra (400 MHz, Chloroform-*d*) of Bis(3-chlorophenyl)(morpholin-3-yl)phosphine oxide (3al).**

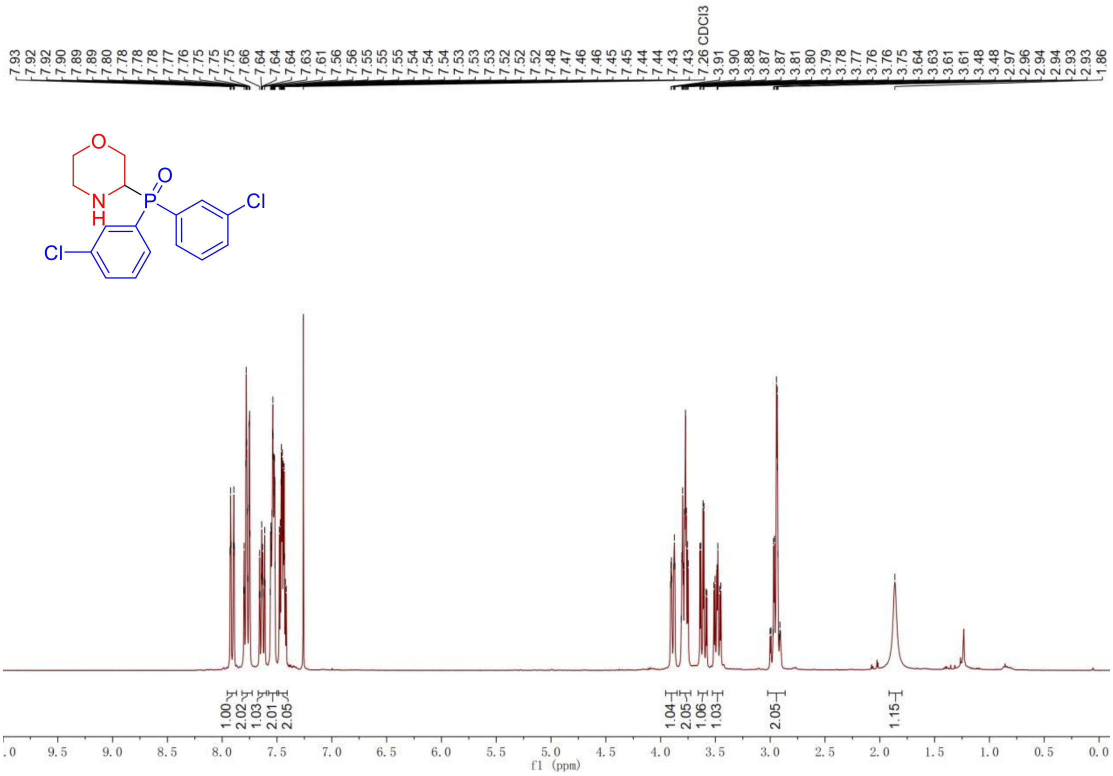

Figure S64. $^{13}C\{^{1}H\}$ **NMR spectra (100 MHz, Chloroform-*d*) of Bis(3-chlorophenyl)(morpholin-3-yl)phosphine oxide (3al).**

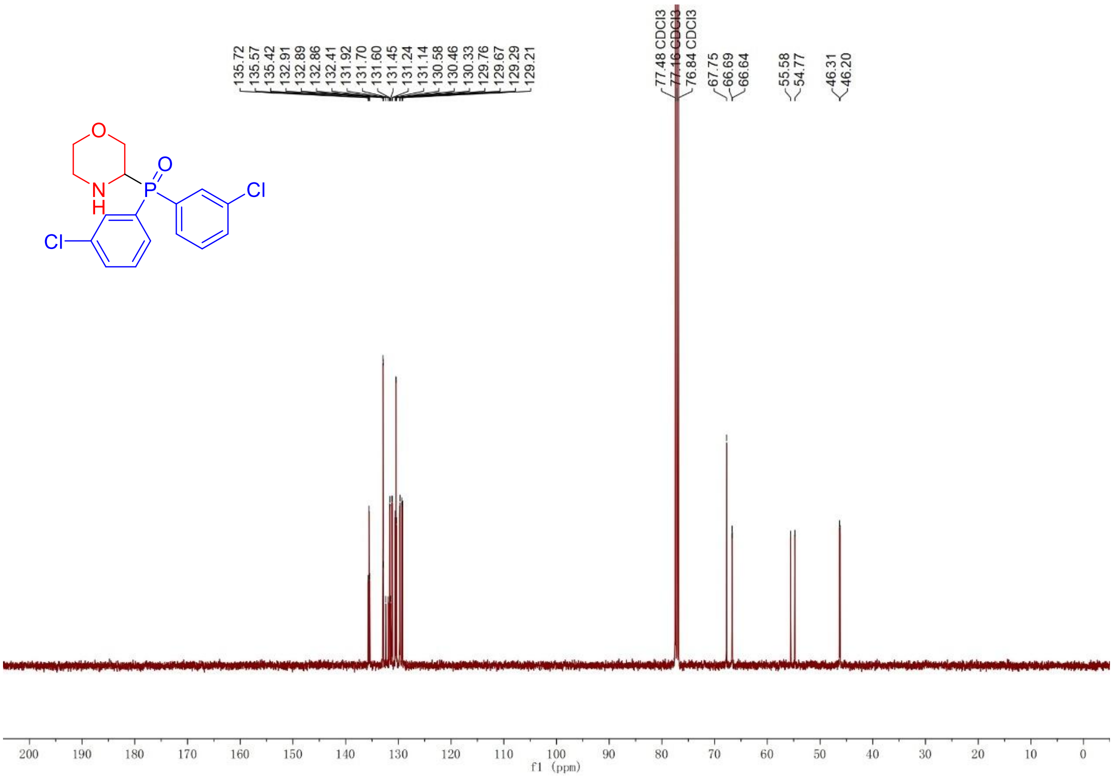

Figure S65. ^{31}P NMR spectra (162 MHz, Chloroform-*d*) of Bis(3-chlorophenyl)(morpholin-3-yl)phosphine oxide (3al).

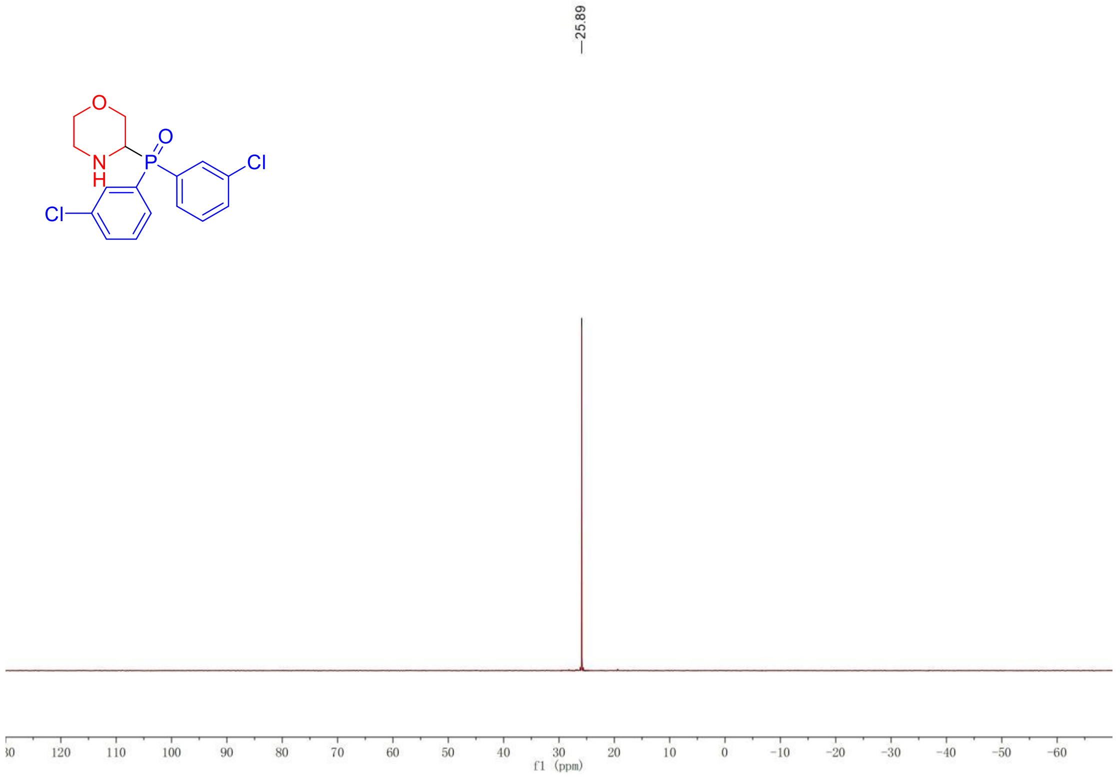

Figure S66. ^{1}H NMR spectra (400 MHz, Chloroform-*d*) of Morpholin-3-ylbis(3-(trifluoromethyl)phenyl)phosphine oxide (3am).

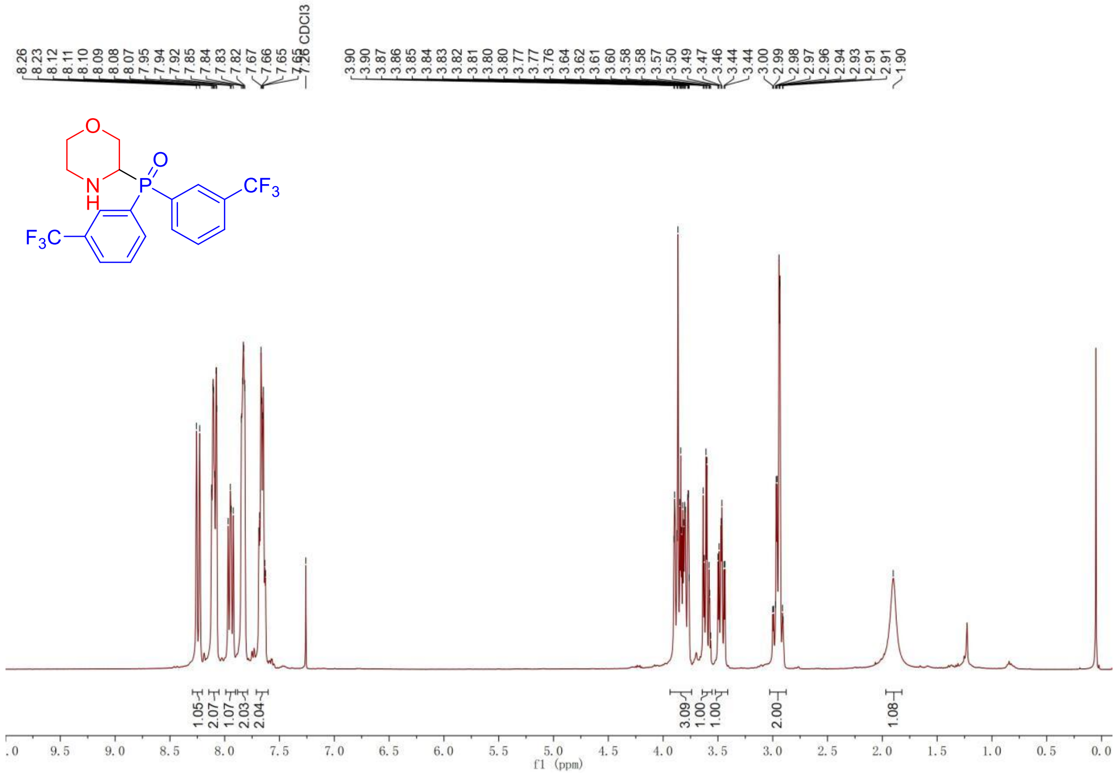

Figure S67. **$^{13}C\{^{1}H\}$ NMR spectra (100 MHz, Chloroform-*d*) of Morpholin-3-ylbis(3-(trifluoromethyl)phenyl)phosphine oxide (3am).**

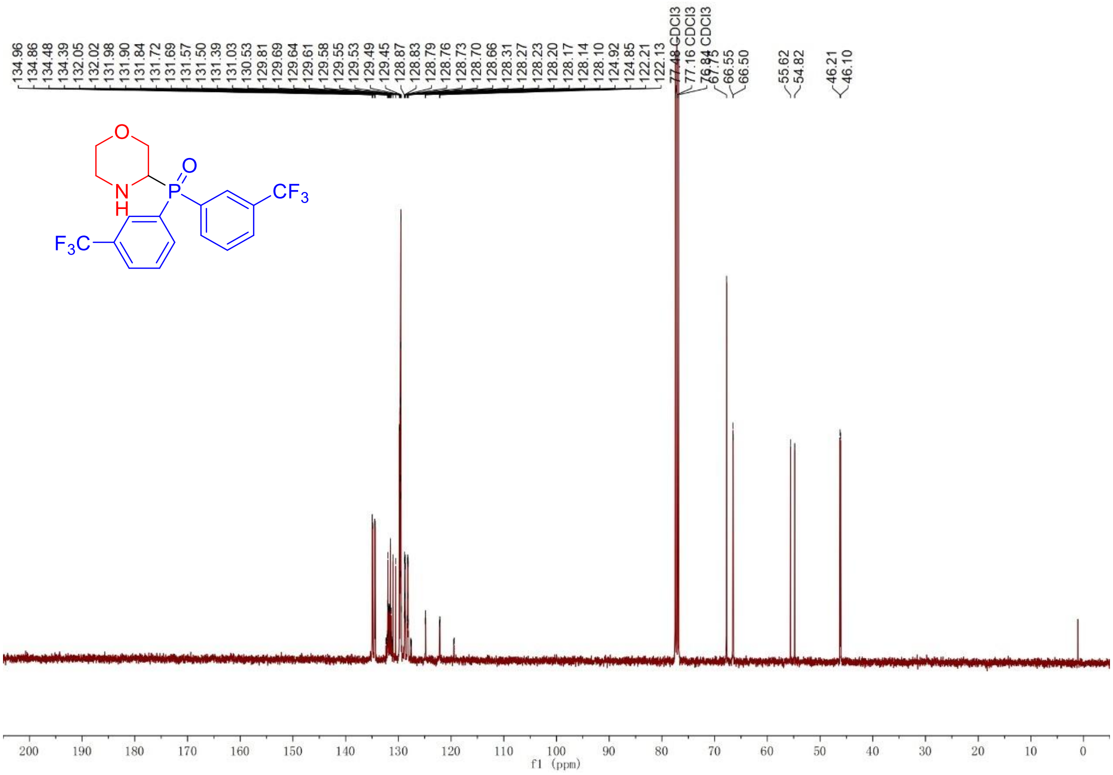

Figure S68. **^{31}P NMR spectra (162 MHz, Chloroform-*d*) of Morpholin-3-ylbis(3-(trifluoromethyl)phenyl)phosphine oxide (3am).**

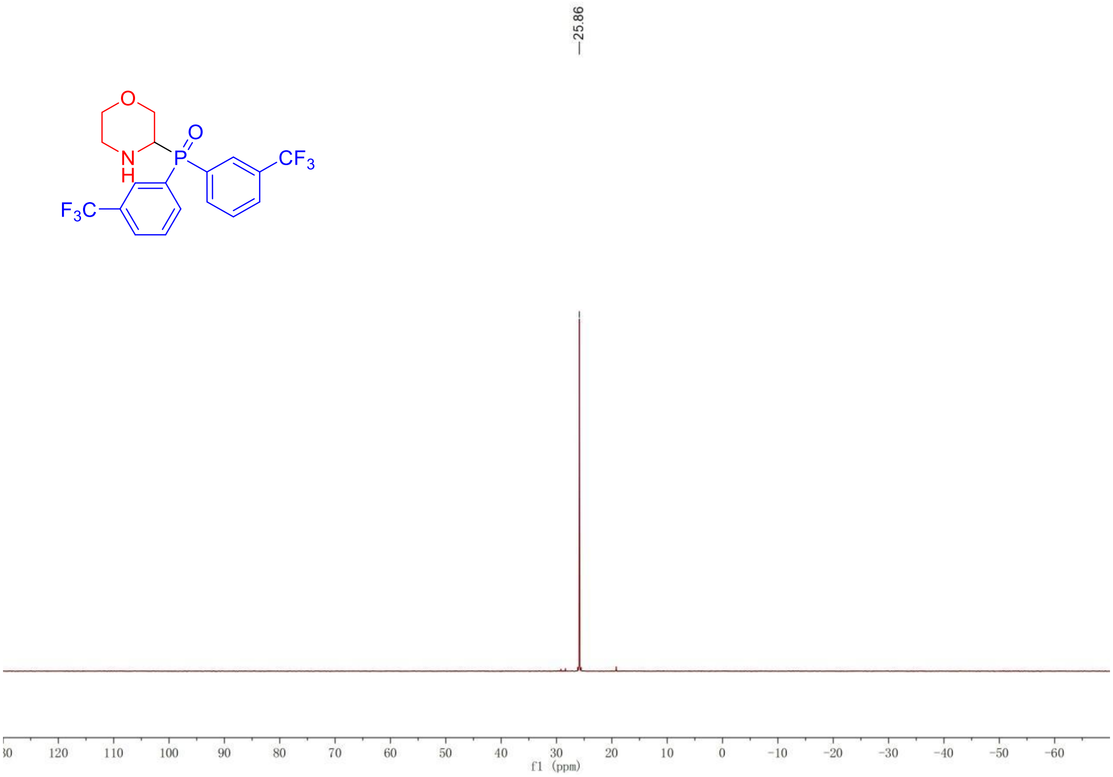

Figure S69. ^{19}F NMR spectra (376 MHz, Chloroform-*d*) of Morpholin-3-ylbis(3-(trifluoromethyl)phenyl)phosphine oxide (3am).

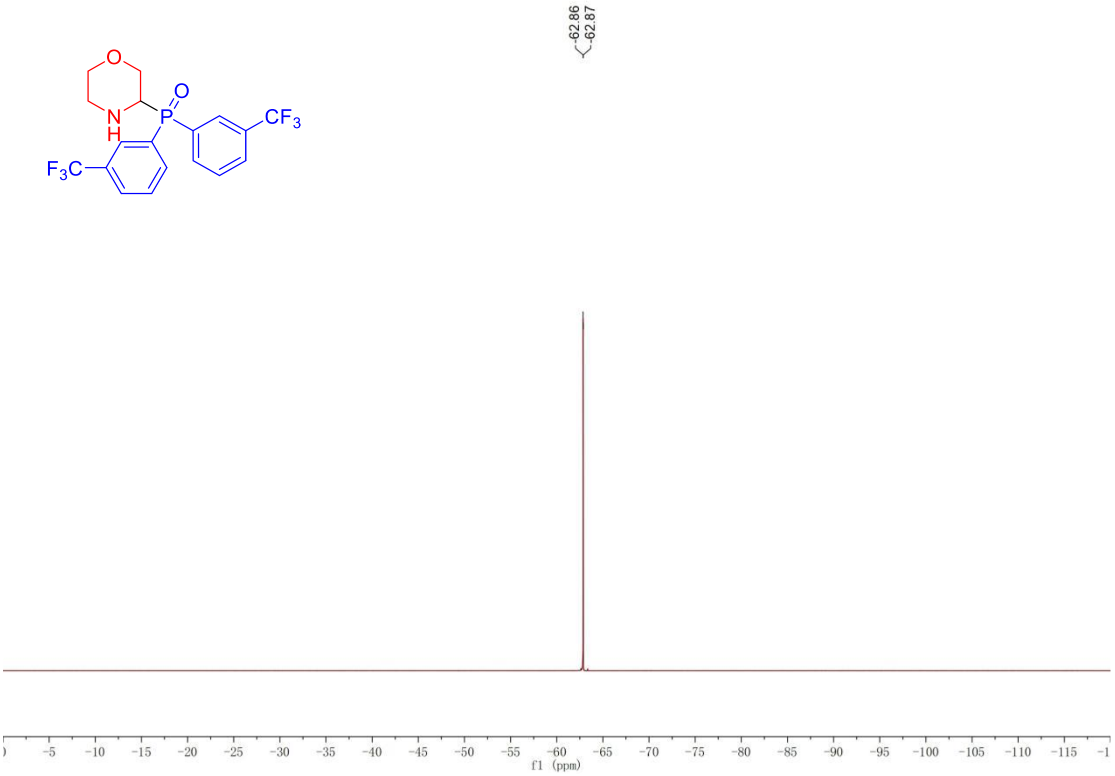

Figure S70. ^{1}H NMR spectra (400 MHz, Chloroform-*d*) of Bis(3,5-dimethylphenyl)(morpholin-3-yl)phosphine oxide (3an).

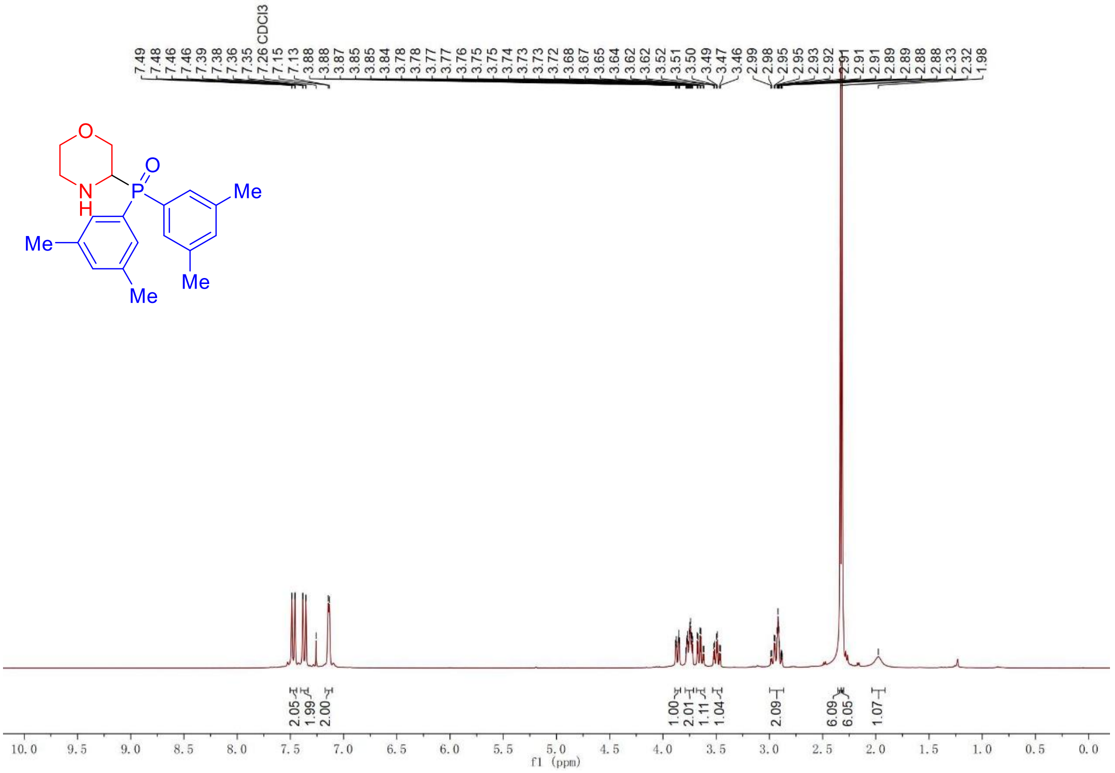

Figure S71. $^{13}C\{^{1}H\}$ **NMR spectra (100 MHz, Chloroform-*d*) of Bis(3,5-dimethylphenyl)(morpholin-3-yl)phosphine oxide (3an).**

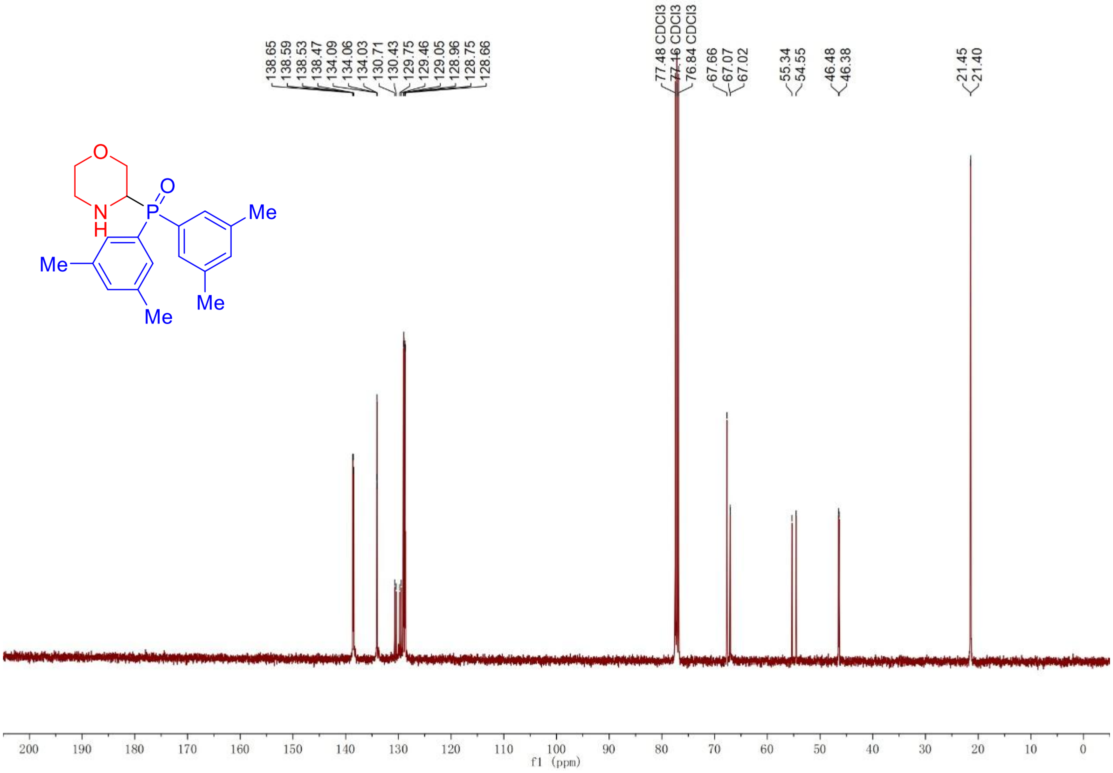

Figure S72. ^{31}P **NMR spectra (162 MHz, Chloroform-*d*) of Bis(3,5-dimethylphenyl)(morpholin-3-yl)phosphine oxide (3an).**

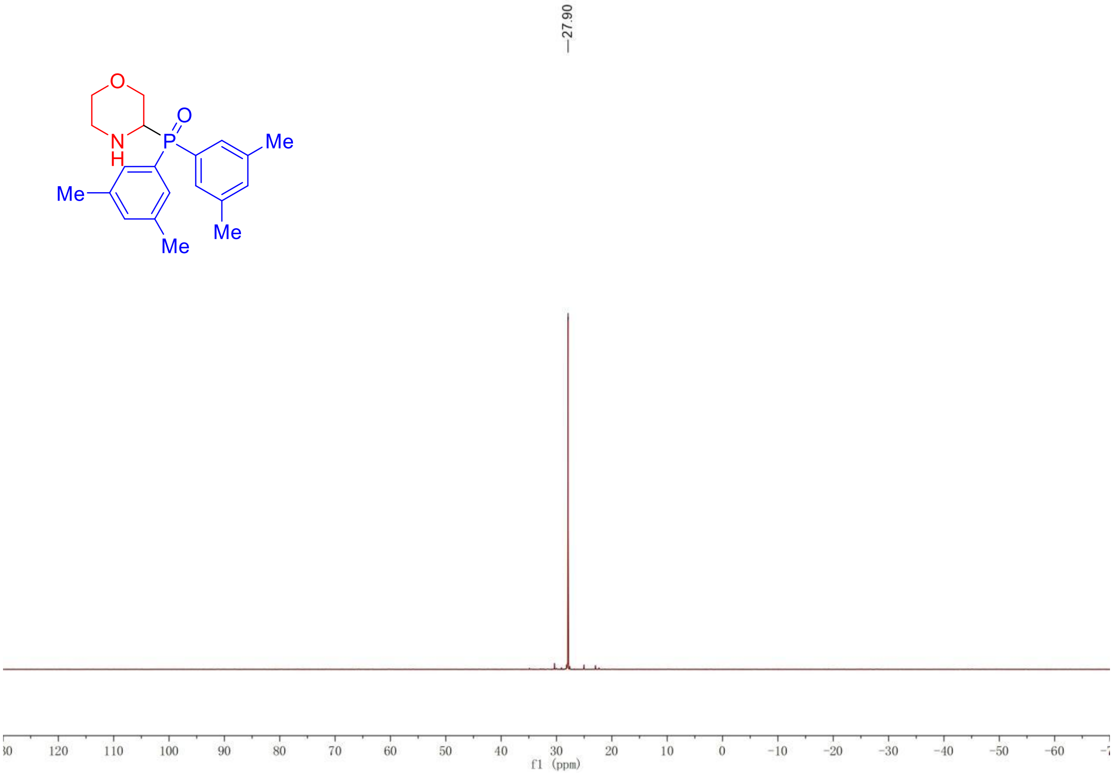

Figure S73. ^{1}H **NMR spectra (400 MHz, Chloroform-*d*) of Bis(3,5-difluorophenyl)(morpholin-3-yl)phosphine oxide (3ao).**

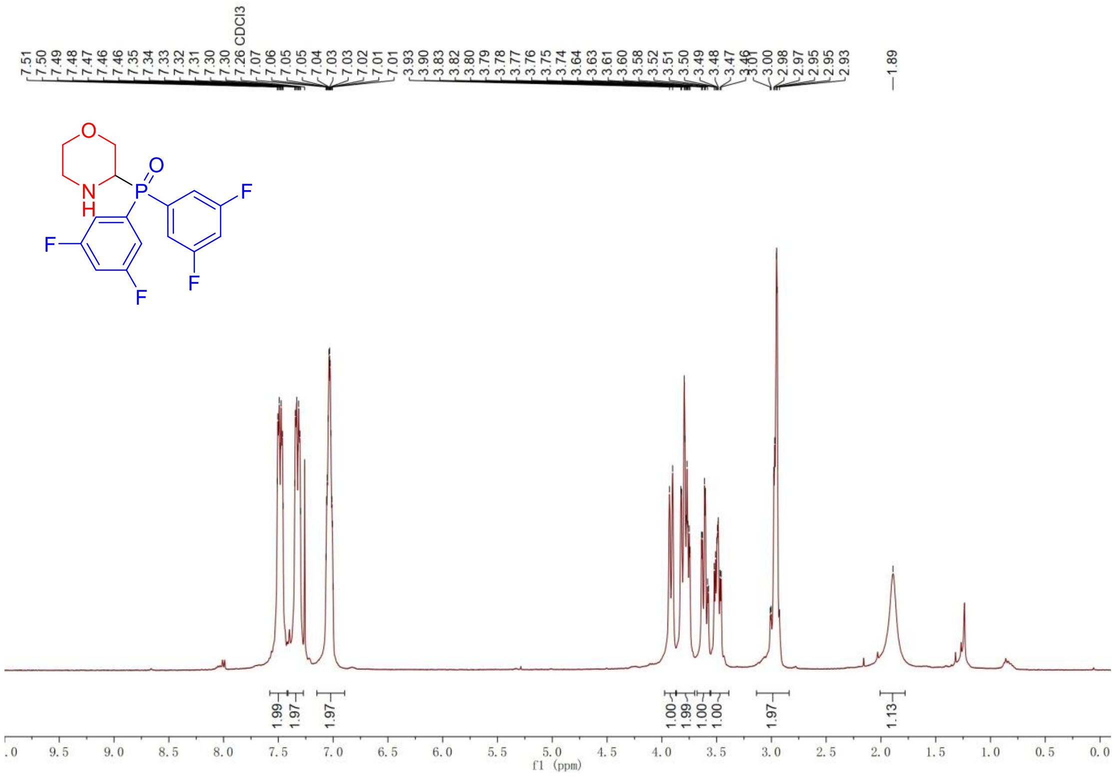

Figure S74. $^{13}C\{^{1}H\}$ **NMR spectra (100 MHz, Chloroform-*d*) of Bis(3,5-difluorophenyl)(morpholin-3-yl)phosphine oxide (3ao).**

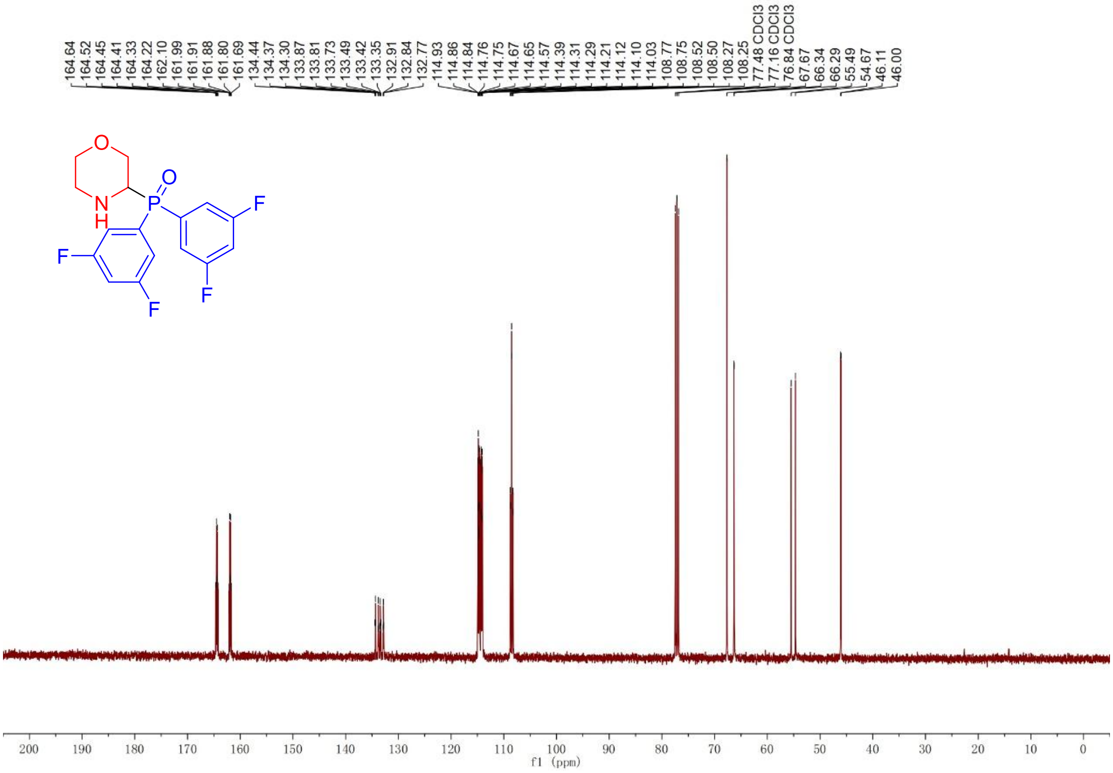

Figure S75. **^{31}P NMR spectra (162 MHz, Chloroform-*d*) of Bis(3,5-difluorophenyl)(morpholin-3-yl)phosphine oxide (3ao).**

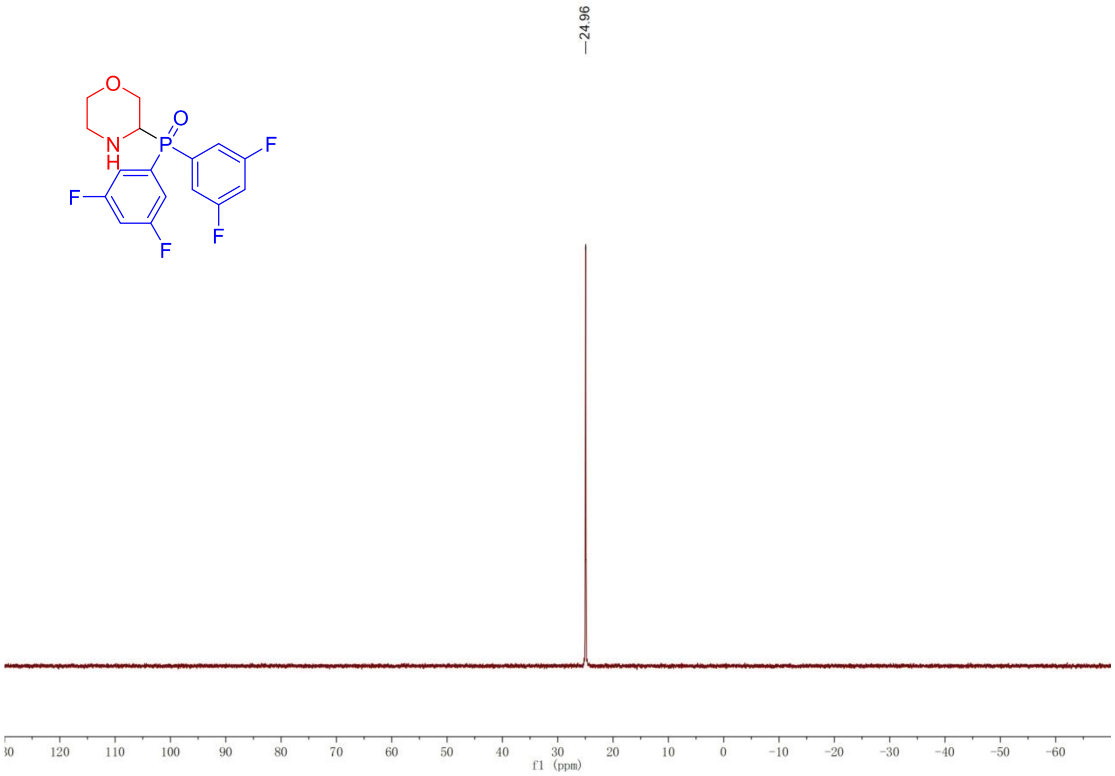

Figure S76. **^{19}F NMR spectra (376 MHz, Chloroform-*d*) of Bis(3,5-difluorophenyl)(morpholin-3-yl)phosphine oxide (3ao).**

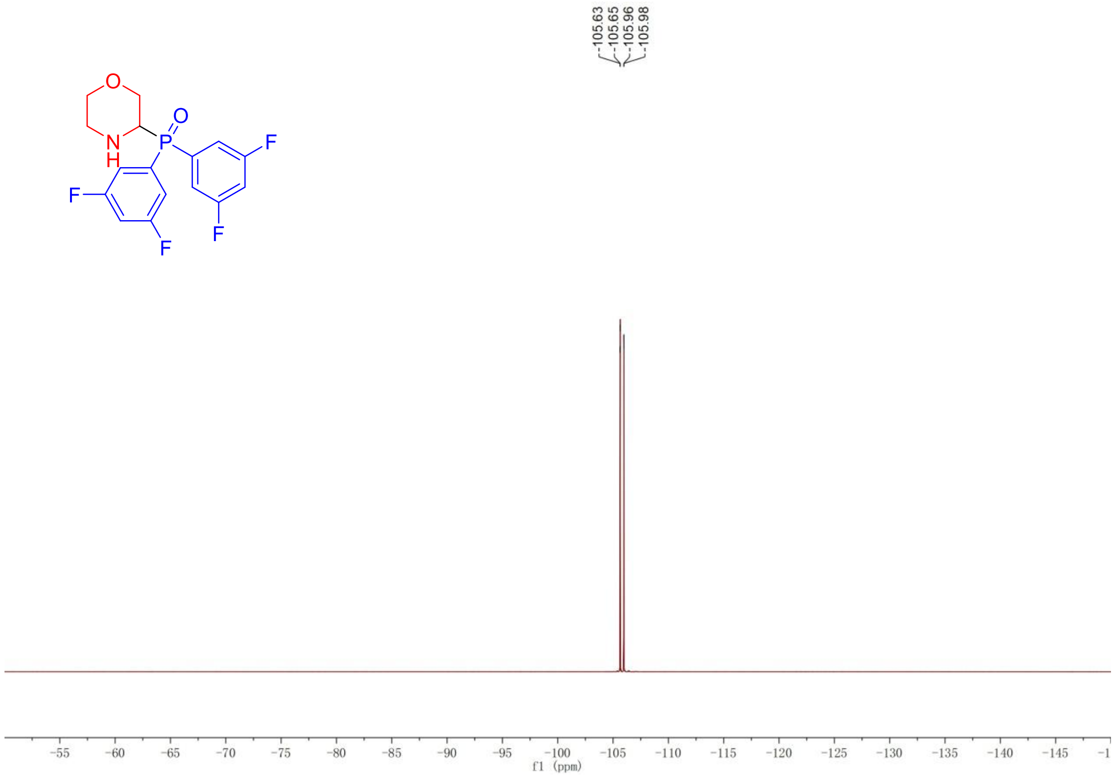

Figure S77. **^{1}H NMR spectra (400 MHz, Chloroform-*d*) of Bis(3-fluoro-4-methylphenyl)(morpholin-3-yl)phosphine oxide (3ap).**

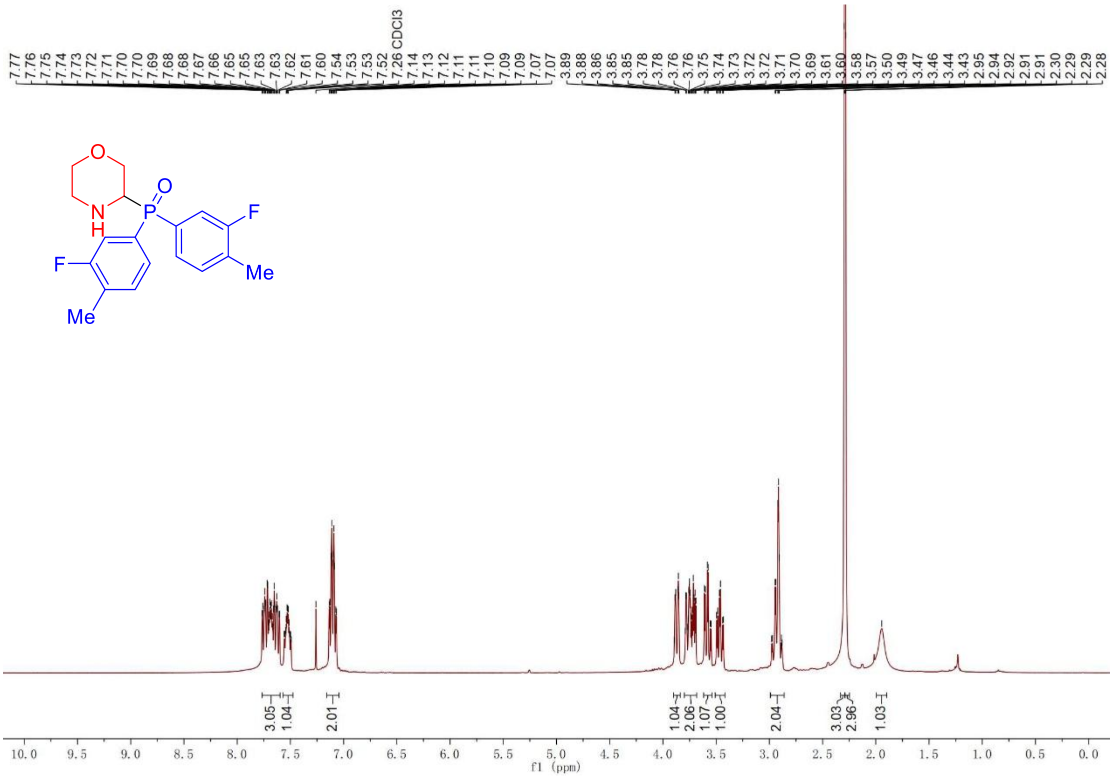

Figure S78. **^{13}C{^{1}H} NMR spectra (100 MHz, Chloroform-*d*) of Bis(3-fluoro-4-methylphenyl)(morpholin-3-yl)phosphine oxide (3ap).**

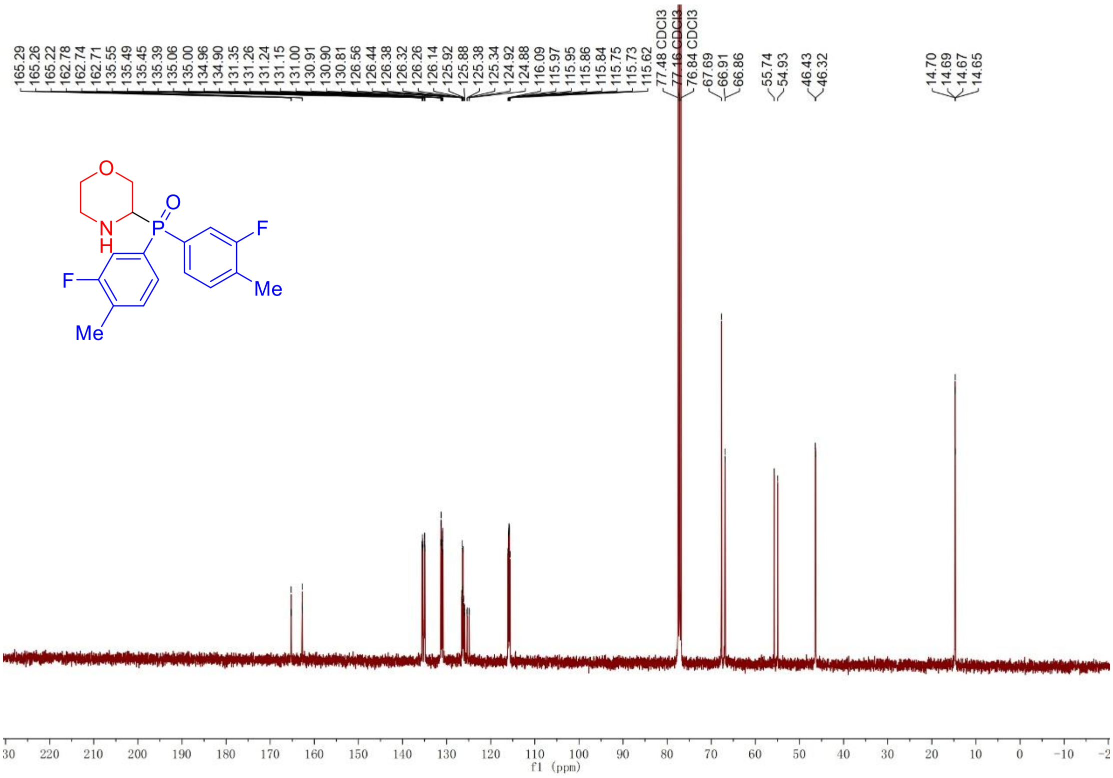

Figure S79. ^{31}P NMR spectra (162 MHz, Chloroform-*d*) of Bis(3-fluoro-4-methylphenyl)(morpholin-3-yl)phosphine oxide (3ap).

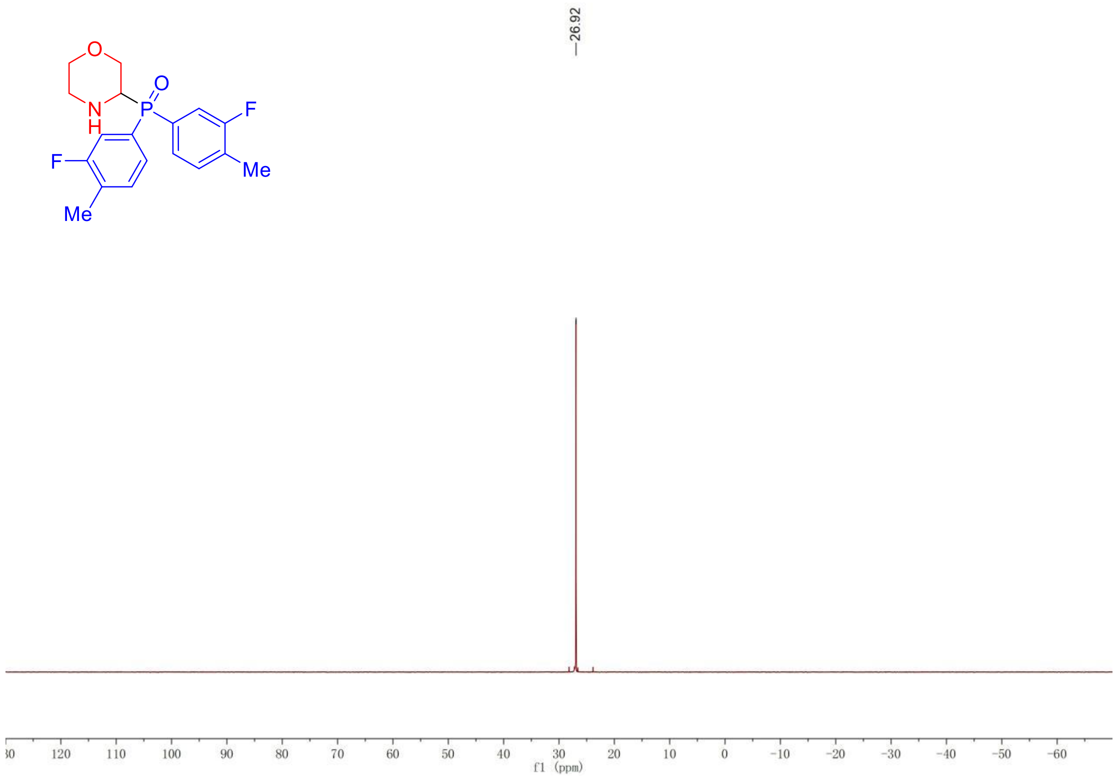

Figure S80. ^{19}F NMR spectra (376 MHz, Chloroform-*d*) of Bis(3-fluoro-4-methylphenyl)(morpholin-3-yl)phosphine oxide (3ap).

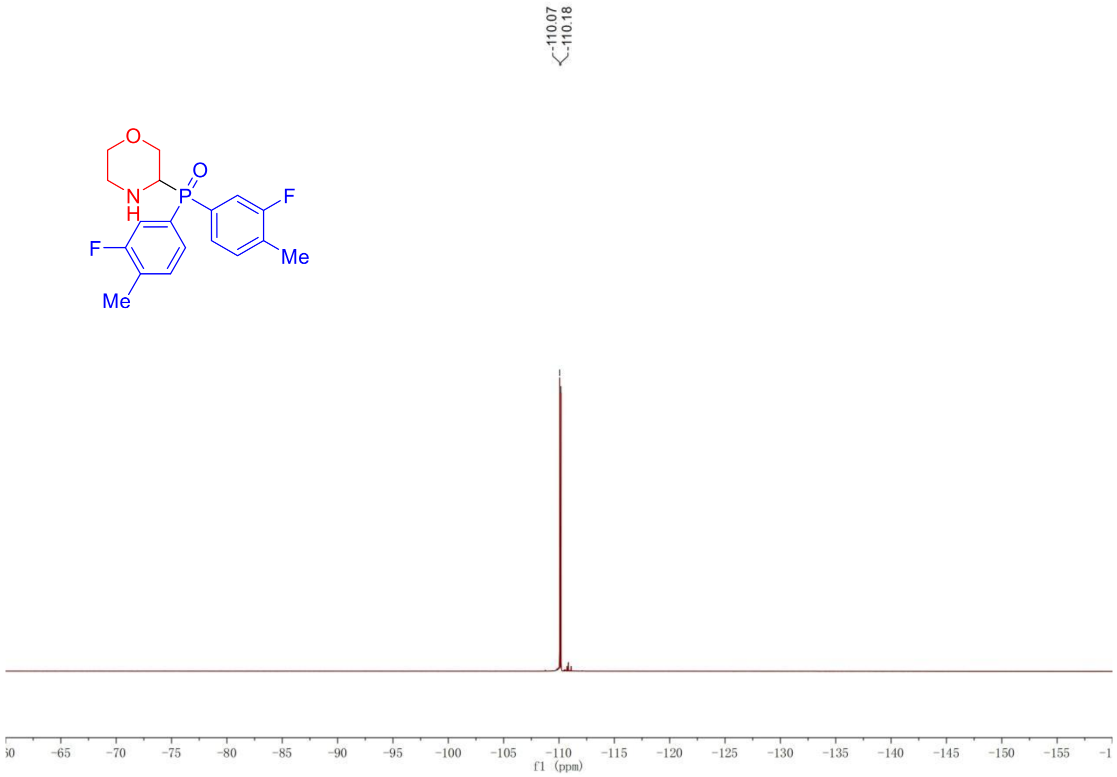

Figure S81. ^{1}H **NMR spectra (400 MHz, Chloroform-*d*) of Morpholin-3-yldi(naphthalen-2-yl)phosphine oxide (3aq).**

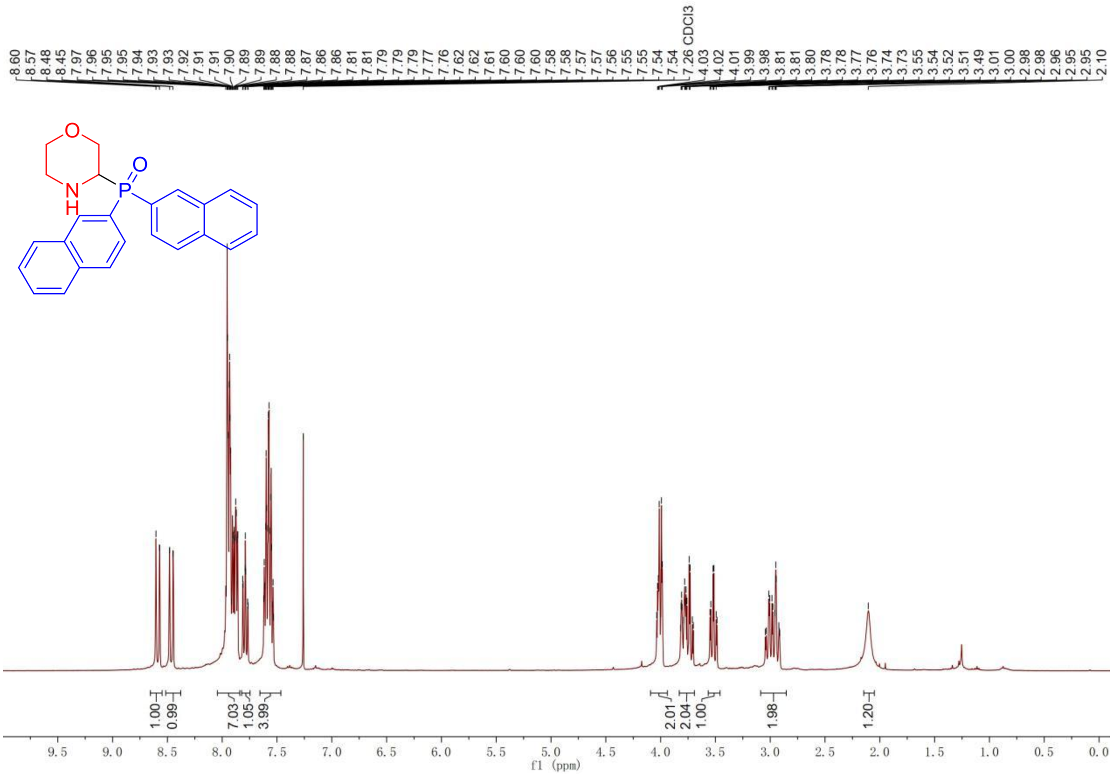

Figure S82. $^{13}C\{^{1}H\}$ **NMR spectra (100 MHz, Chloroform-*d*) of Morpholin-3-yldi(naphthalen-2-yl)phosphine oxide (3aq).**

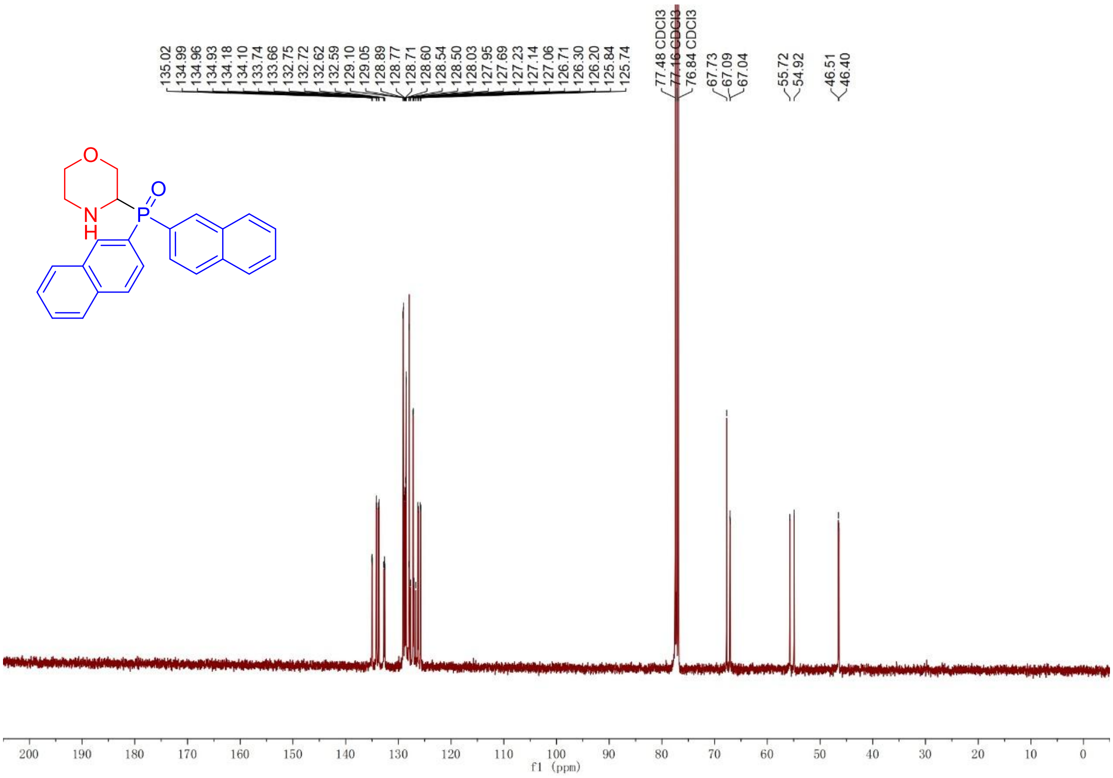

Figure S83. ^{31}**P NMR spectra (162 MHz, Chloroform-*d*) of Morpholin-3-yldi(naphthalen-2-yl)phosphine oxide (3aq).**

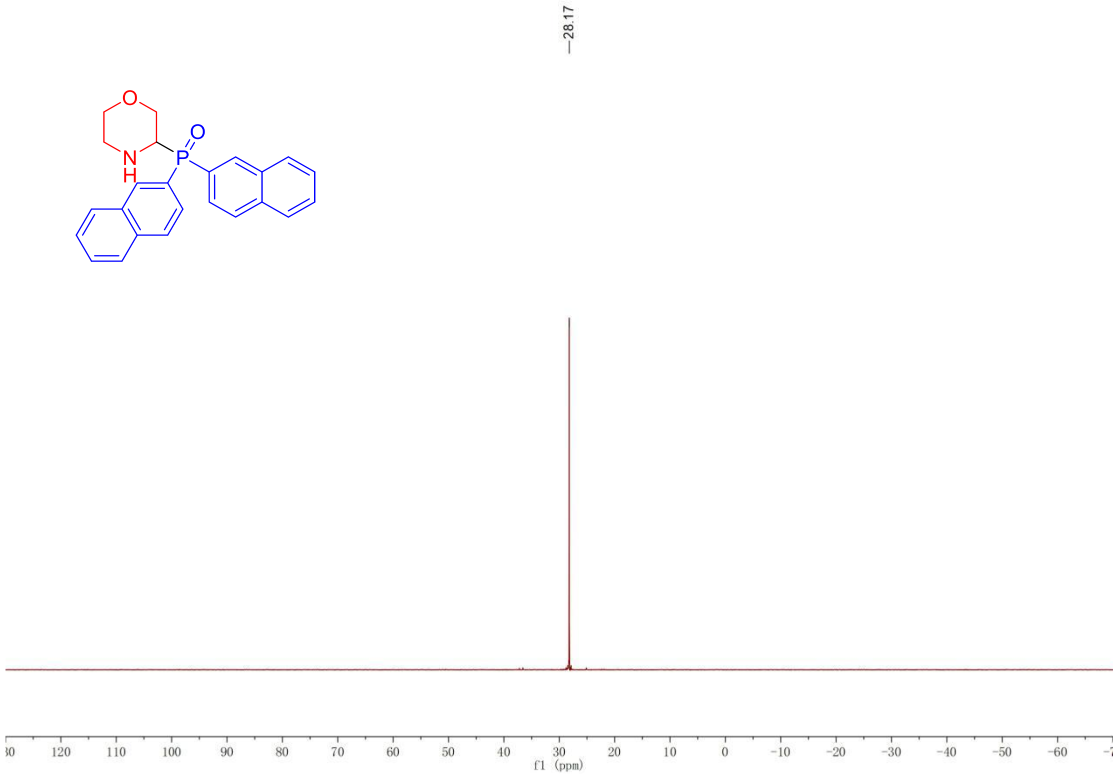

Figure S84. ^{1}**H NMR spectra (400 MHz, Chloroform-*d*) of Di(benzofuran-5-yl)(morpholin-3-yl)phosphine oxide (3ar).**

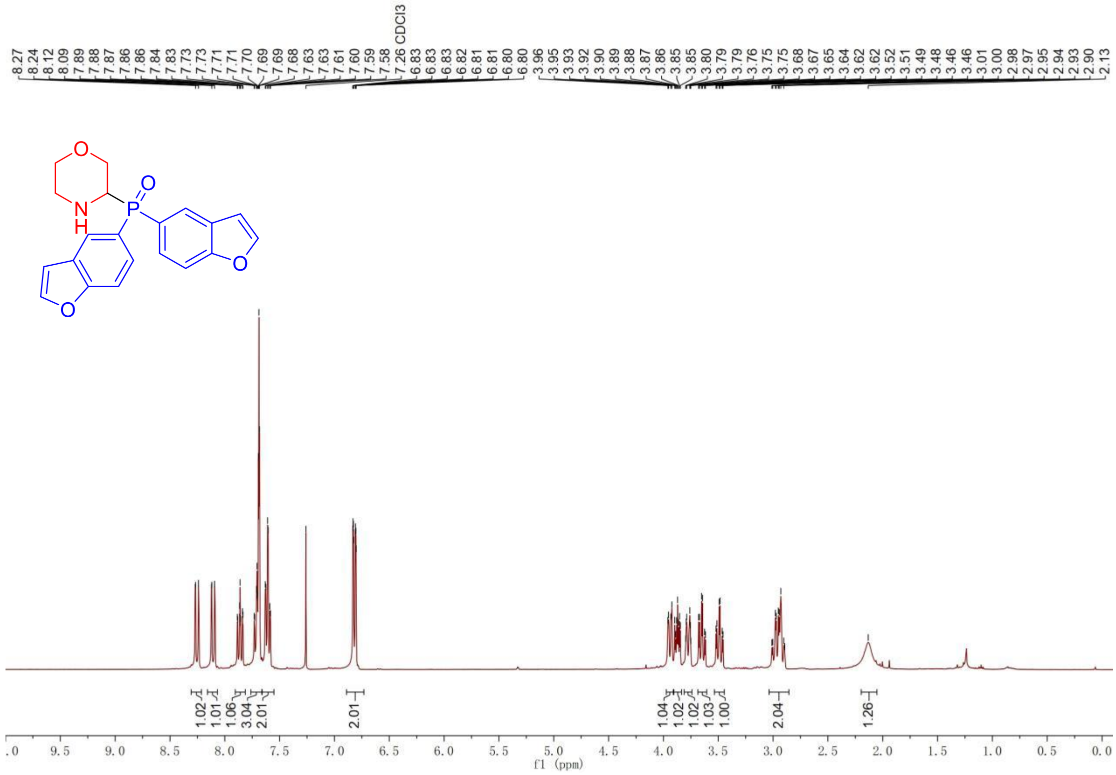

Figure S85. $^{13}C\{^{1}H\}$ **NMR spectra (100 MHz, Chloroform-*d*) of Di(benzofuran-5-yl)(morpholin-3-yl)phosphine oxide (3ar).**

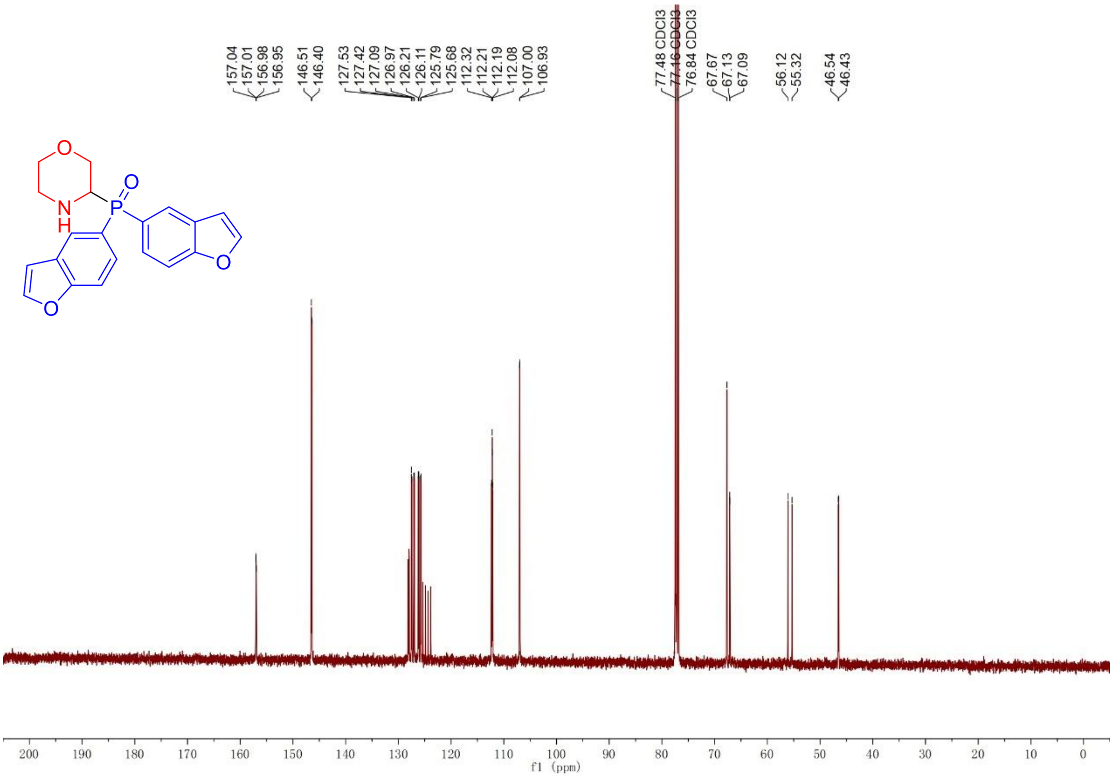

Figure S86. ^{31}P **NMR spectra (162 MHz, Chloroform-*d*) of Di(benzofuran-5-yl)(morpholin-3-yl)phosphine oxide (3ar).**

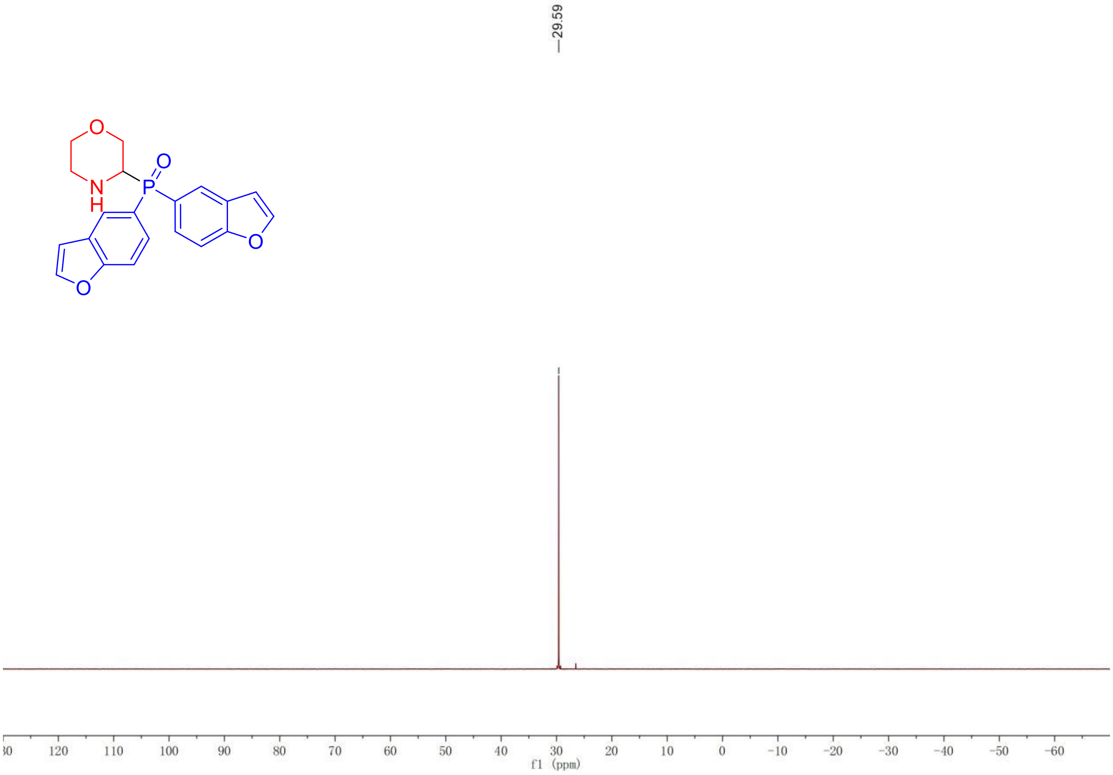

Figure S87. ^{1}H NMR spectra (400 MHz, Chloroform-*d*) of Bis(benzo[*d*][1,3]dioxol-5-yl)(morpholin-3-yl)phosphine oxide (3as).

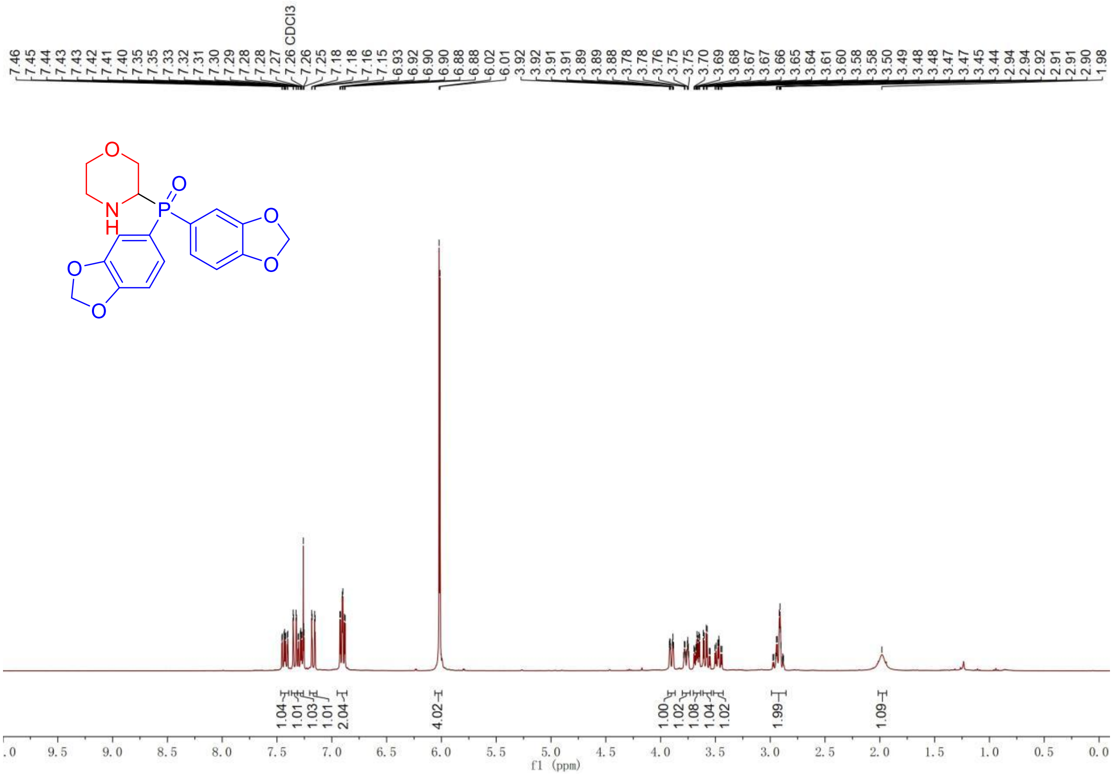

Figure S88. $^{13}C\{^{1}H\}$ NMR spectra (100 MHz, Chloroform-*d*) of Bis(benzo[*d*][1,3]dioxol-5-yl)(morpholin-3-yl)phosphine oxide (3as).

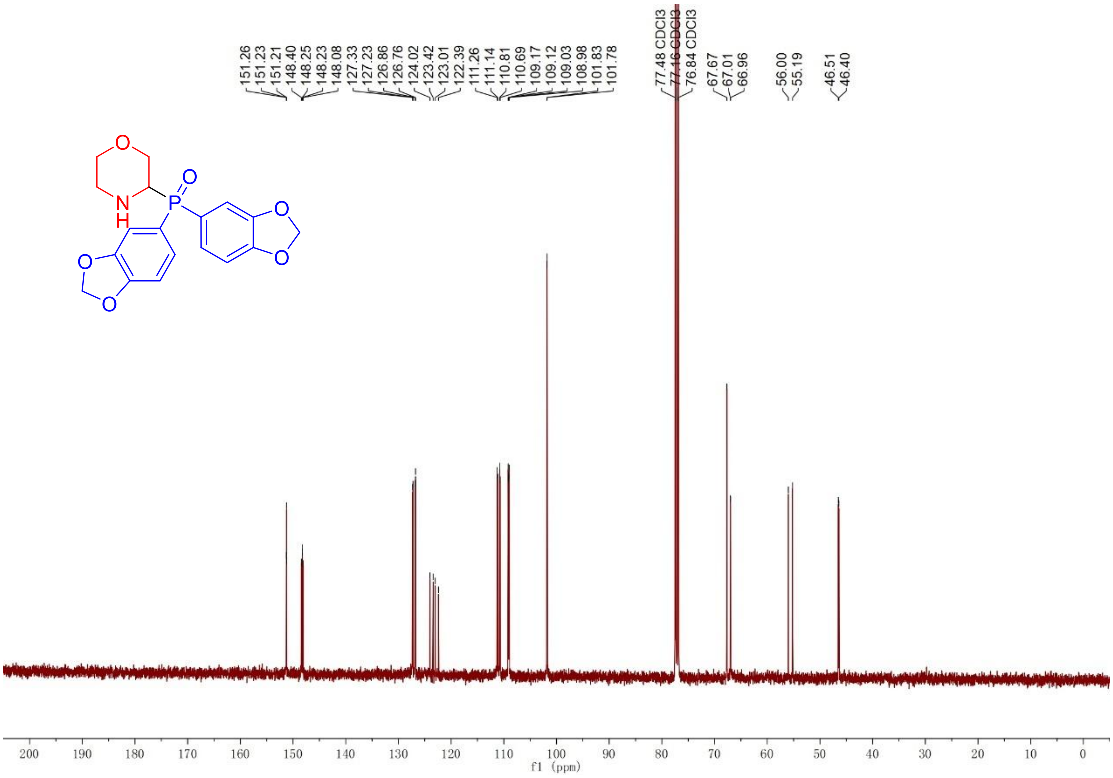

Figure S89. ^{31}**P NMR spectra (162 MHz, Chloroform-*d*) of Bis(benzo[*d*][1,3]dioxol-5-yl)(morpholin-3-yl)phosphine oxide (3as).**

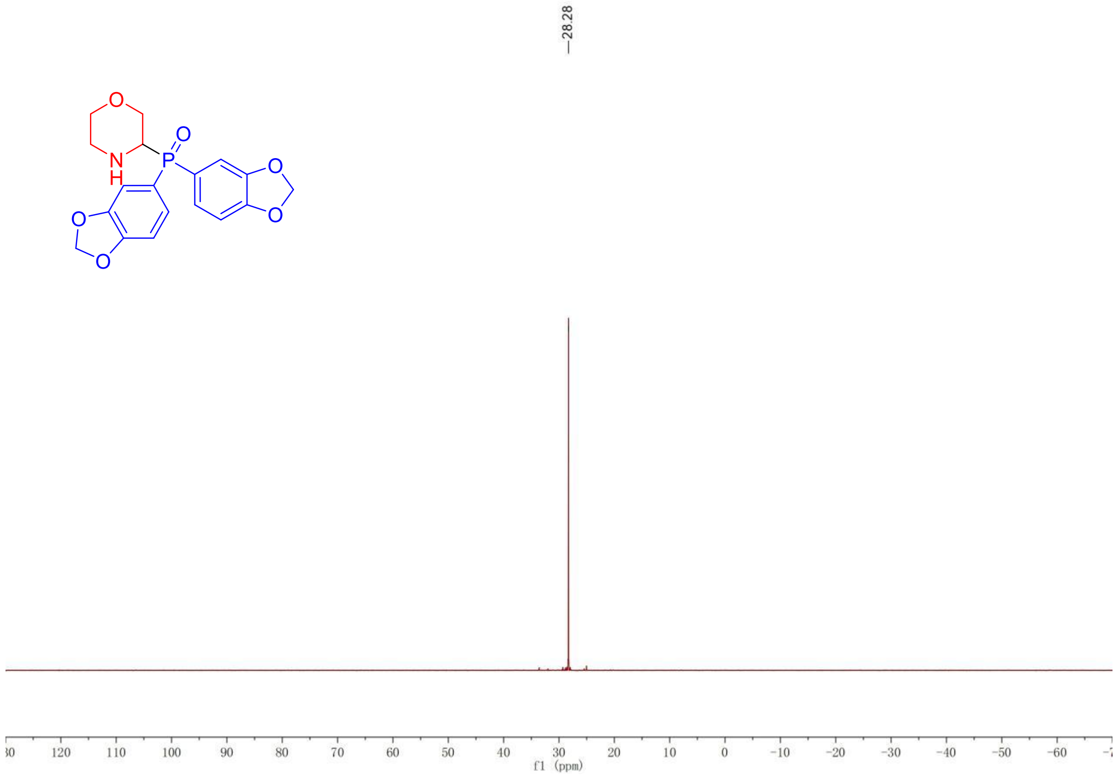

Figure S90. ^{1}**H NMR spectra (400 MHz, Chloroform-*d*) of Morpholin-3-yldi(thiophen-2-yl)phosphine oxide (3at).**

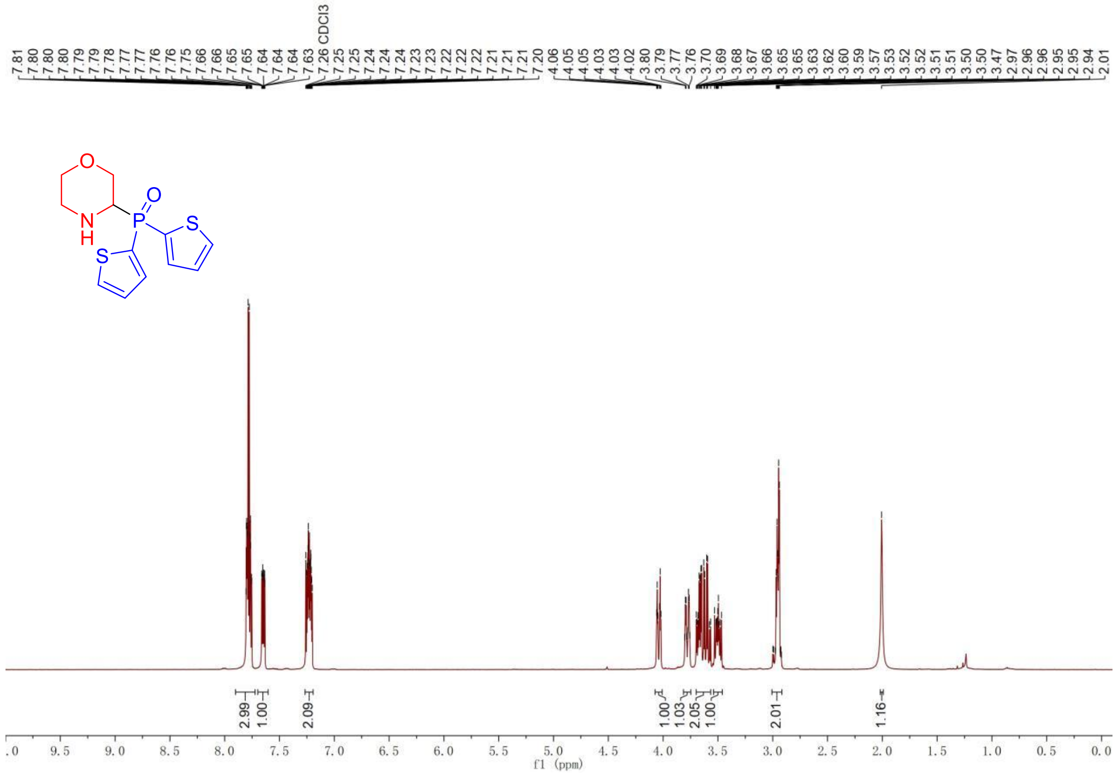

Figure S91. $^{13}C\{^{1}H\}$ **NMR spectra (100 MHz, Chloroform-*d*) of Morpholin-3-yldi(thiophen-2-yl)phosphine oxide (3at).**

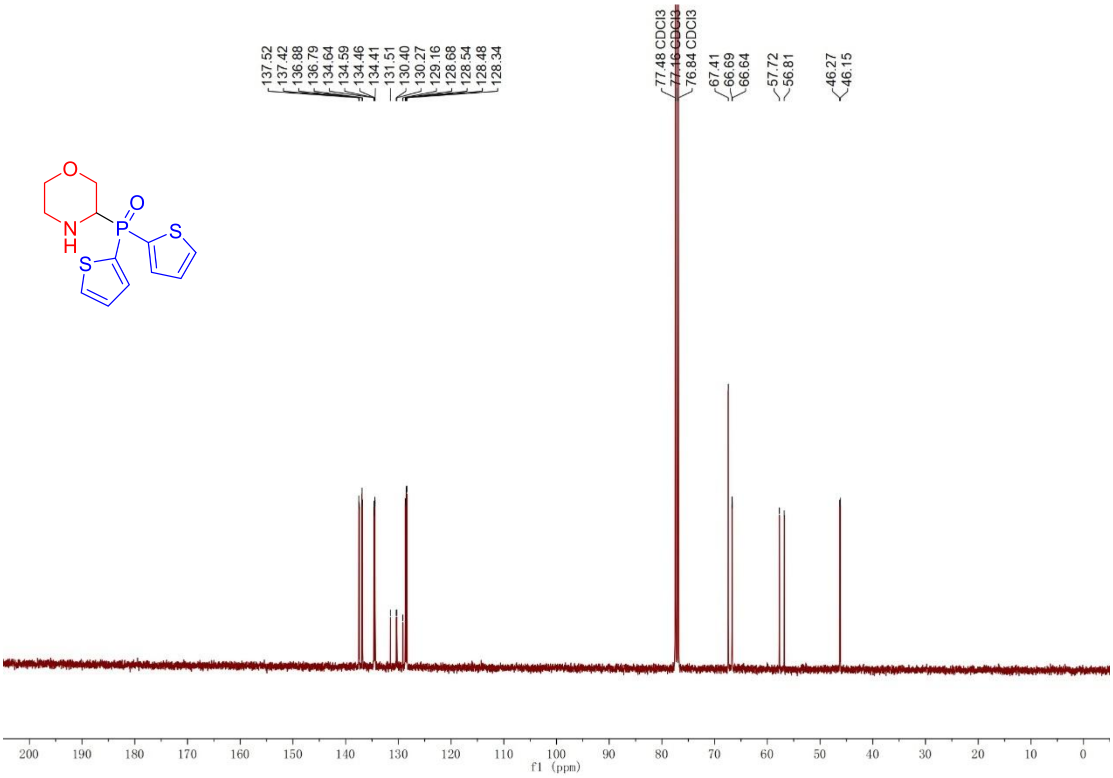

Figure S92. ^{31}P **NMR spectra (162 MHz, Chloroform-*d*) of Morpholin-3-yldi(thiophen-2-yl)phosphine oxide (3at).**

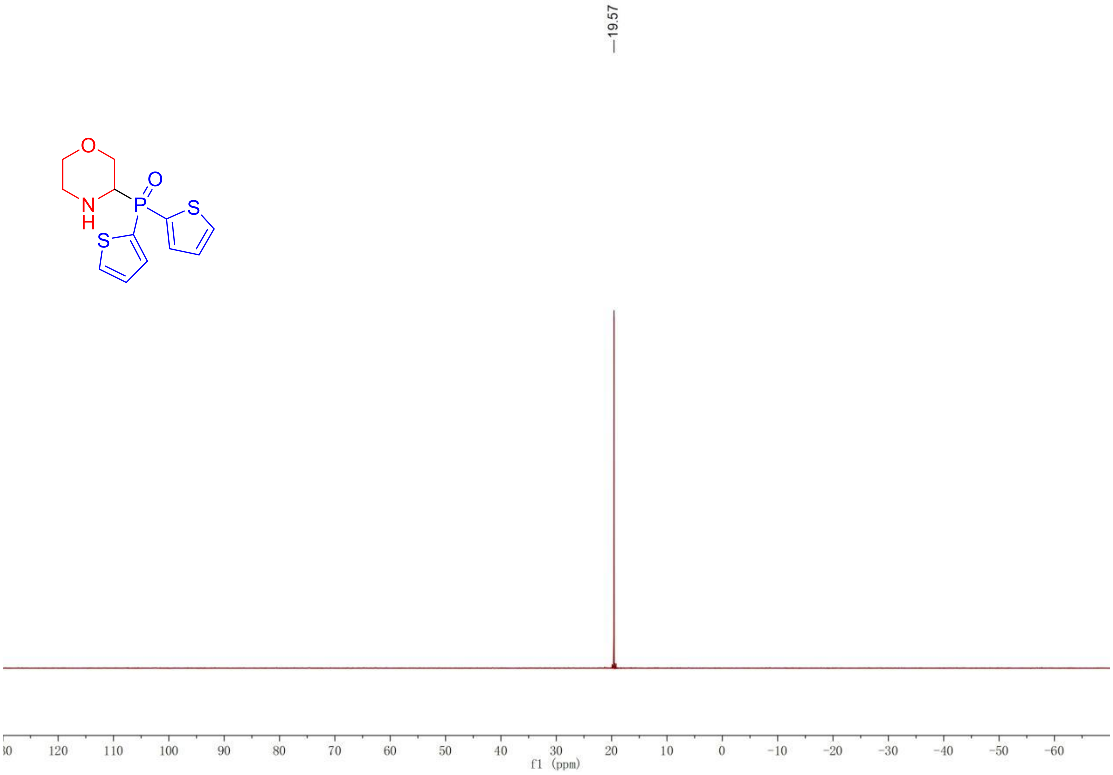

Figure S93. ^{1}**H NMR spectra (400 MHz, Chloroform-*d*) of Morpholin-3-yl(phenyl)(*p*-tolyl)phosphine oxide (3au).**

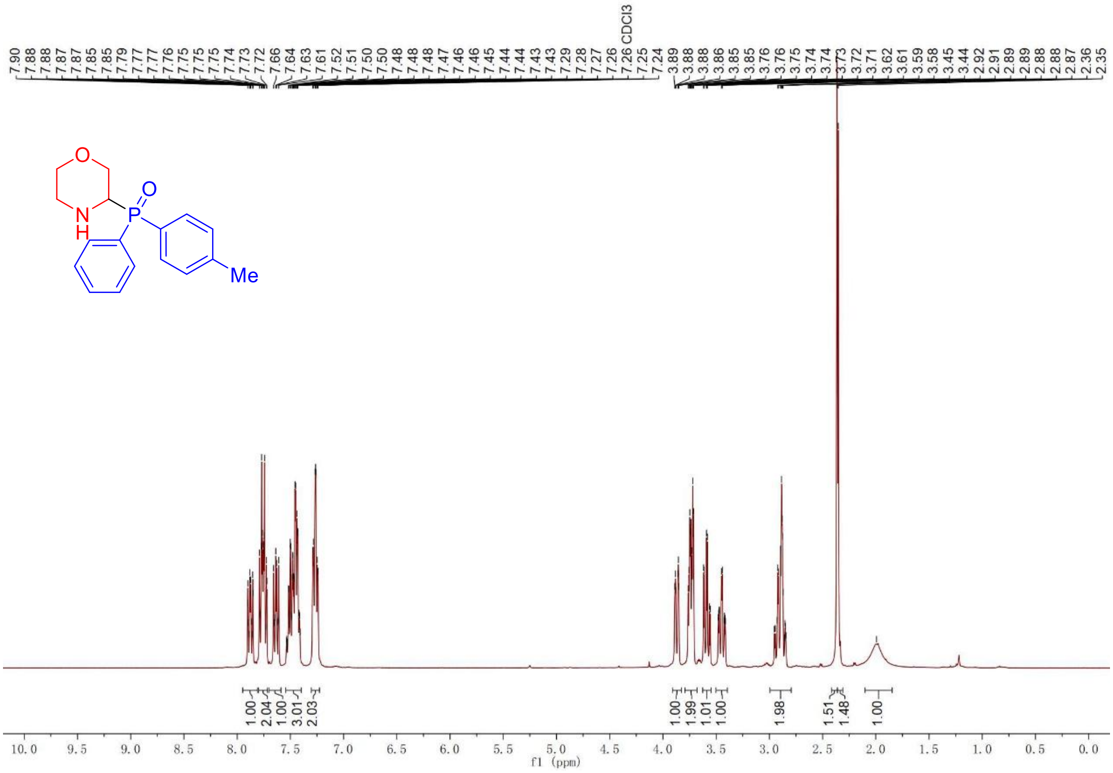

Figure S94. ^{13}**C{**^{1}**H} NMR spectra (100 MHz, Chloroform-*d*) of Morpholin-3-yl(phenyl)(*p*-tolyl)phosphine oxide (3au).**

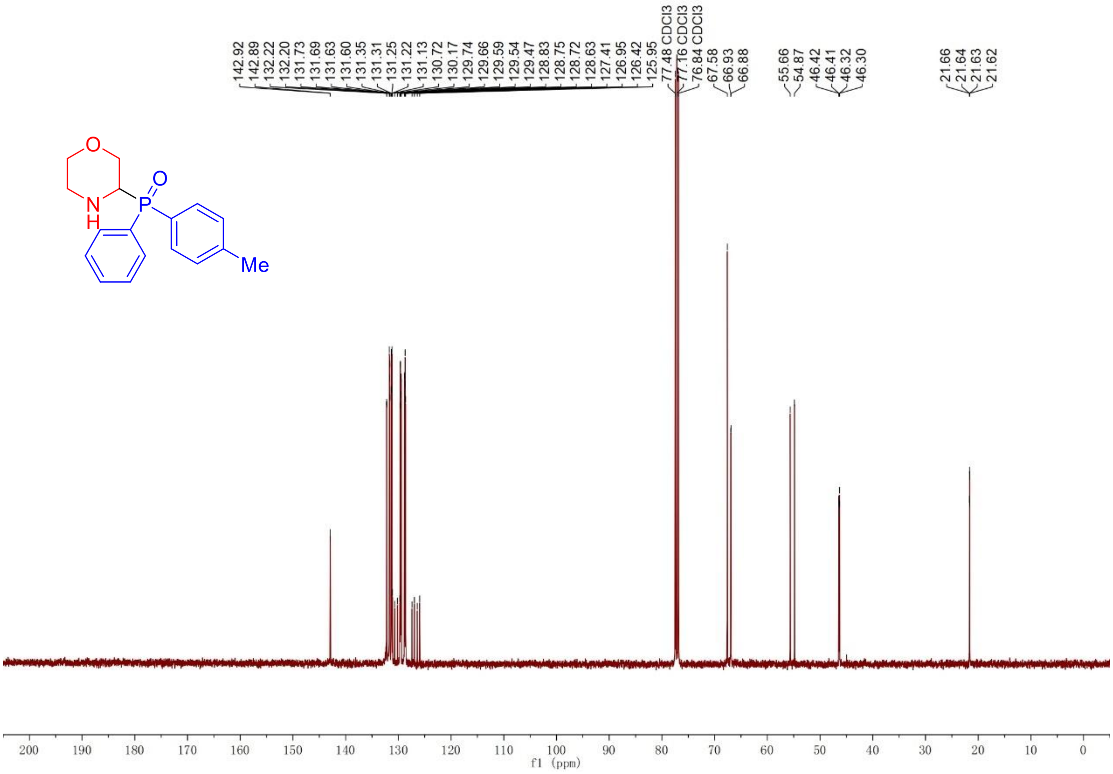

Figure S95. ^{31}**P NMR spectra (162 MHz, Chloroform-*d*) of Morpholin-3-yl(phenyl)(*p*-tolyl)phosphine oxide (3au).**

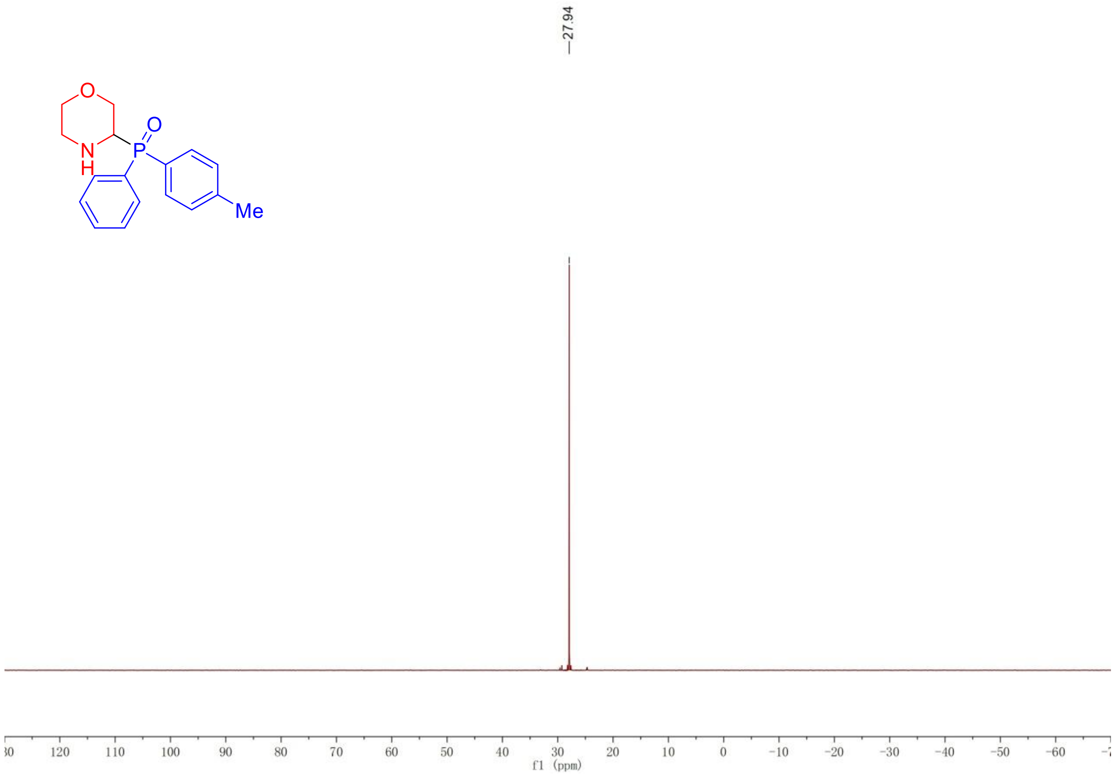

Figure S96. ^{1}**H NMR spectra (400 MHz, Chloroform-*d*) of Morpholin-3-yl(phenyl)(thiophen-2-yl)phosphine oxide (3av).**

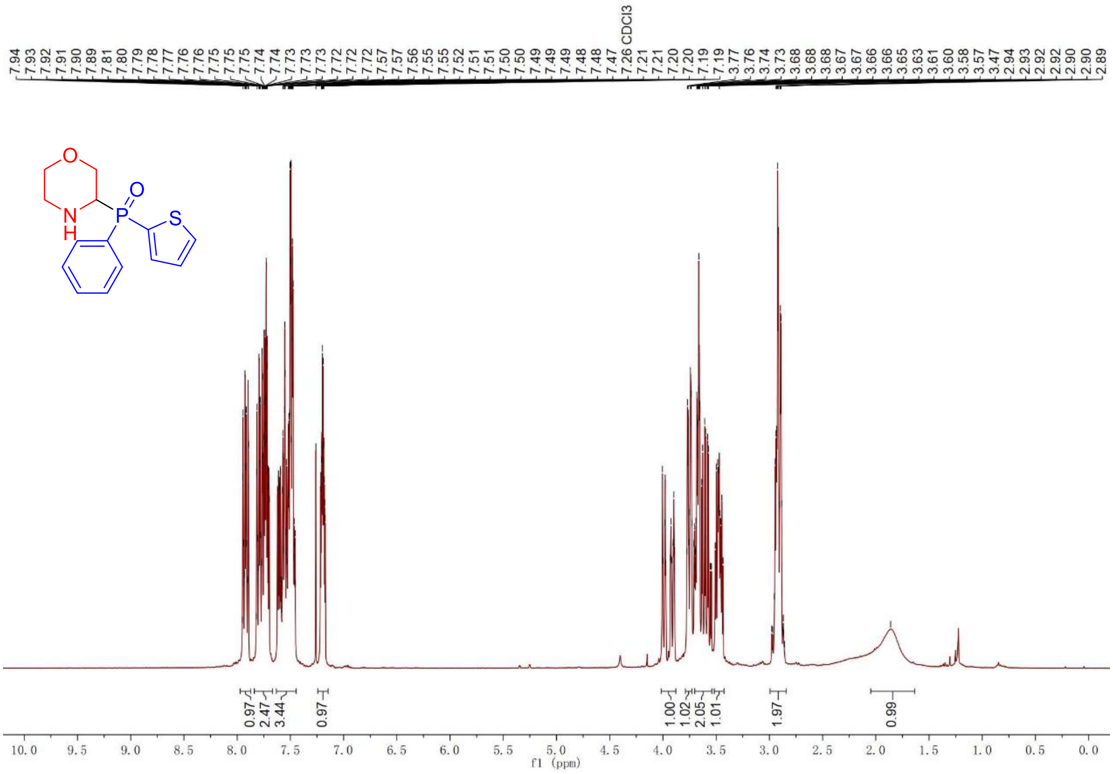

Figure S97. **$^{13}C\{^{1}H\}$ NMR spectra (100 MHz, Chloroform-*d*) of Morpholin-3-yl(phenyl)(thiophen-2-yl)phosphine oxide (3av).**

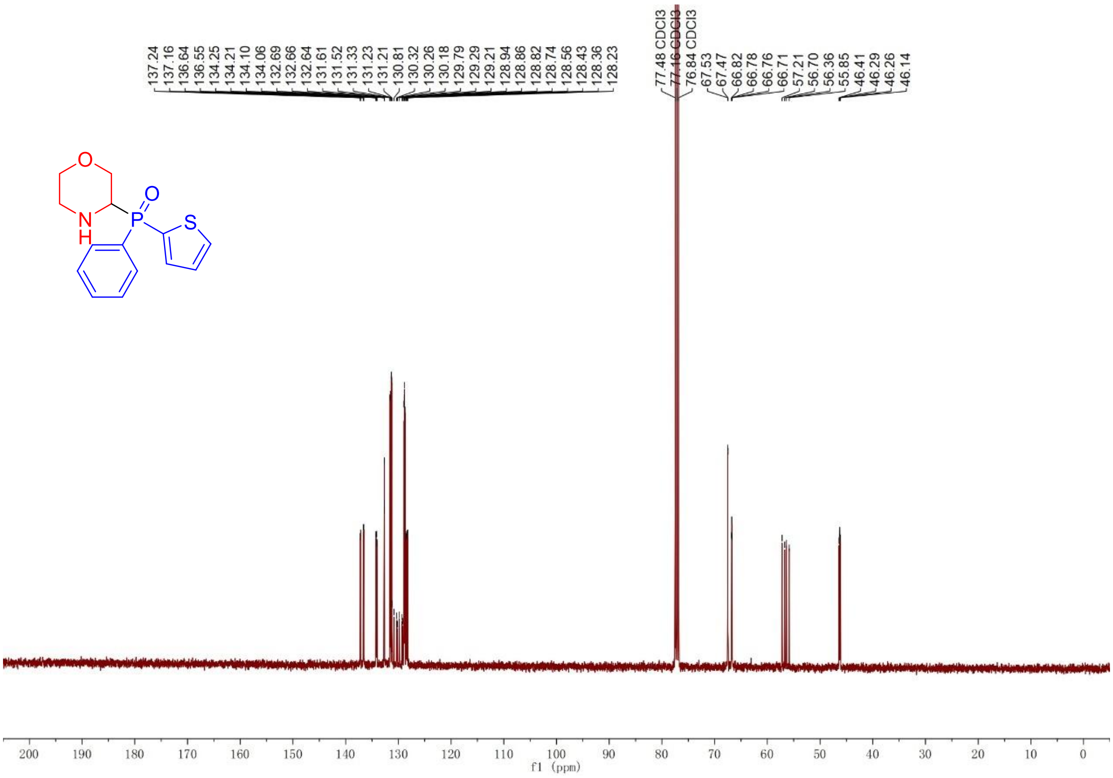

Figure S98. **^{31}P NMR spectra (162 MHz, Chloroform-*d*) of Morpholin-3-yl(phenyl)(thiophen-2-yl)phosphine oxide (3av).**

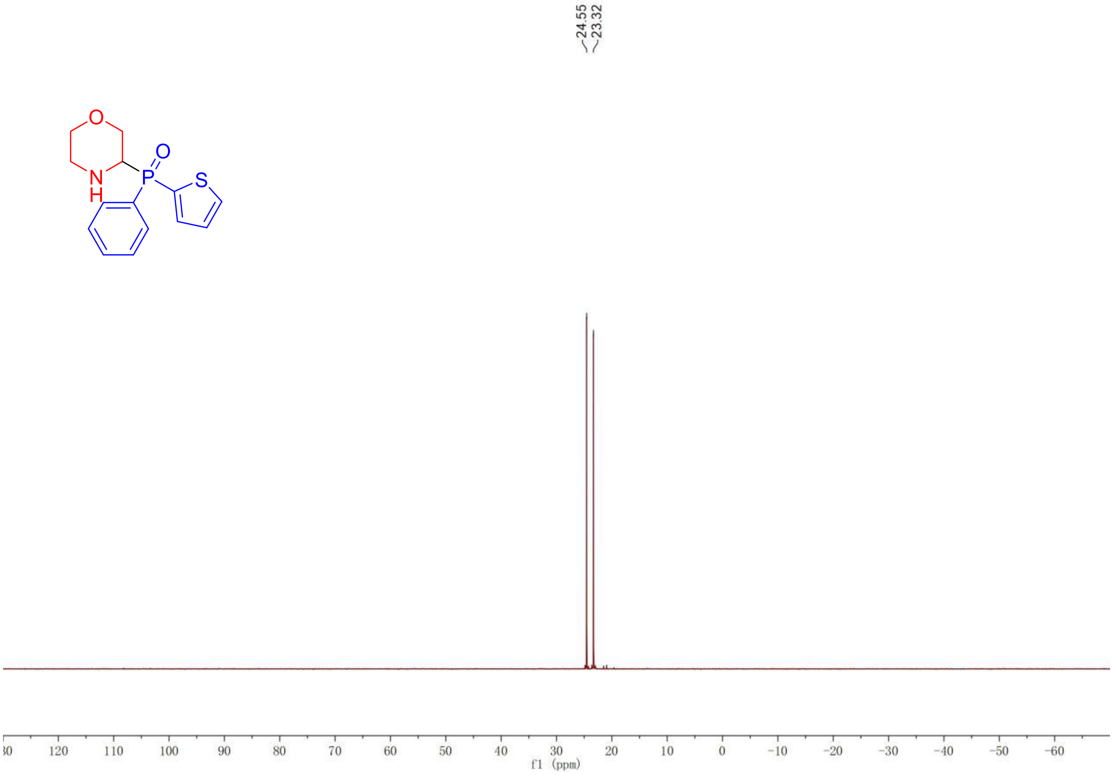

Figure S99. **^{1}H NMR spectra (400 MHz, Chloroform-*d*) of Diphenyl(pyrrolidin-2-yl)phosphine oxide (3ba).**

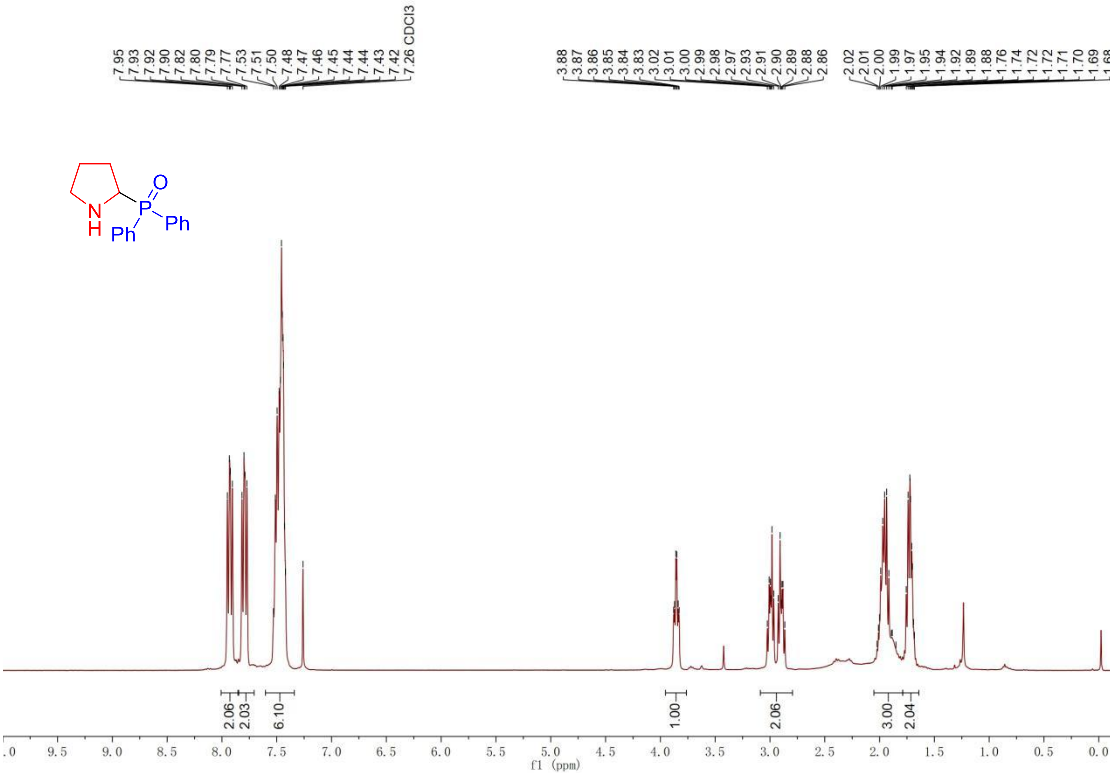

Figure S100. **^{13}C{^{1}H} NMR spectra (100 MHz, Chloroform-*d*) of Diphenyl(pyrrolidin-2-yl)phosphine oxide (3ba).**

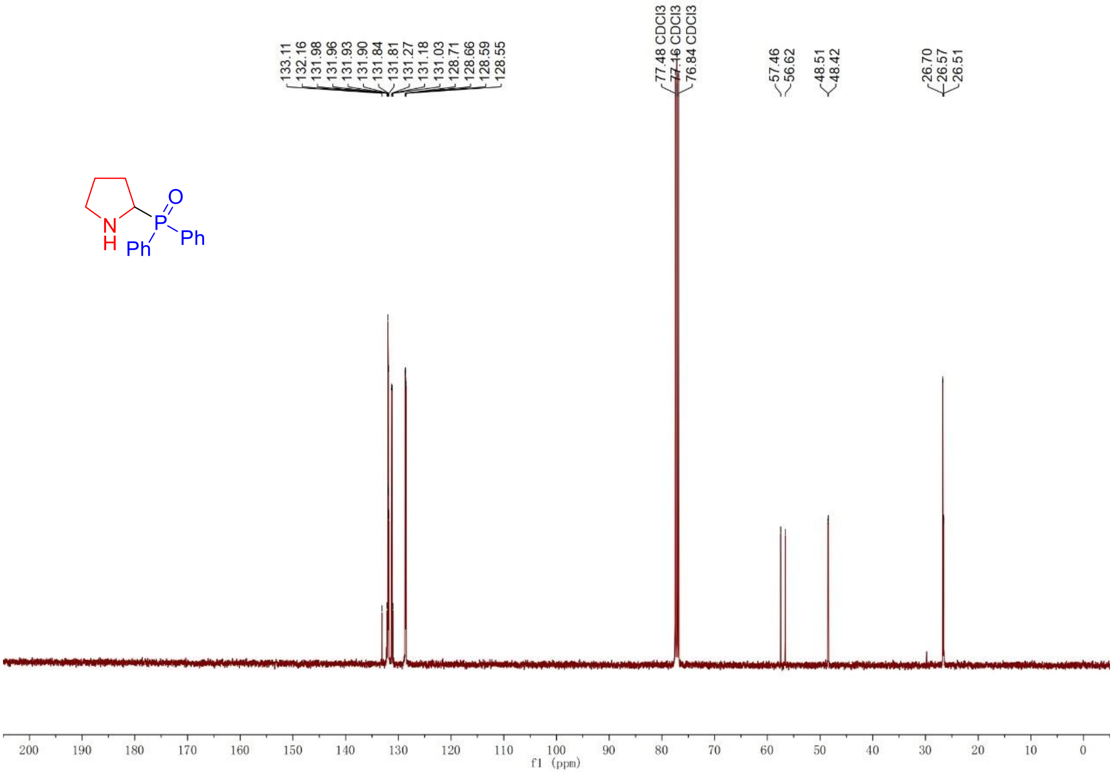

Figure S101. ^{31}**P NMR spectra (162 MHz, Chloroform-*d*) of Diphenyl(pyrrolidin-2-yl)phosphine oxide (3ba).**

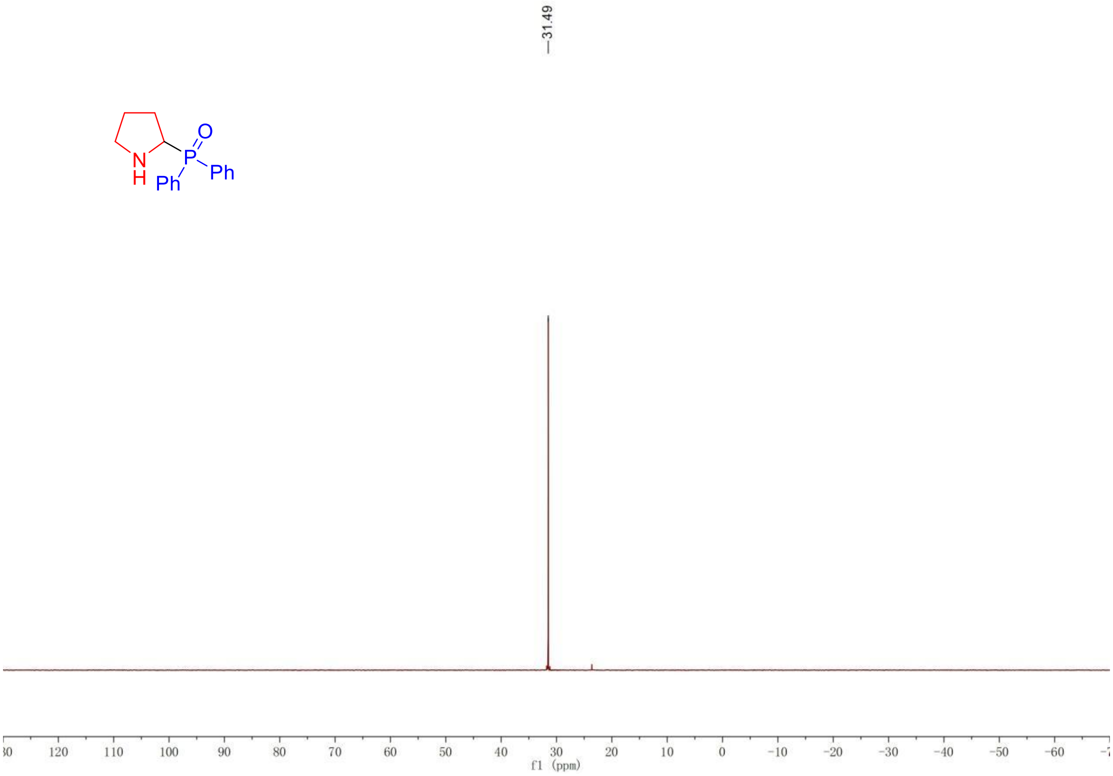

Figure S102. ^{1}**H NMR spectra (400 MHz, Chloroform-*d*) of Diphenyl(piperidin-2-yl)phosphine oxide (3ca).**

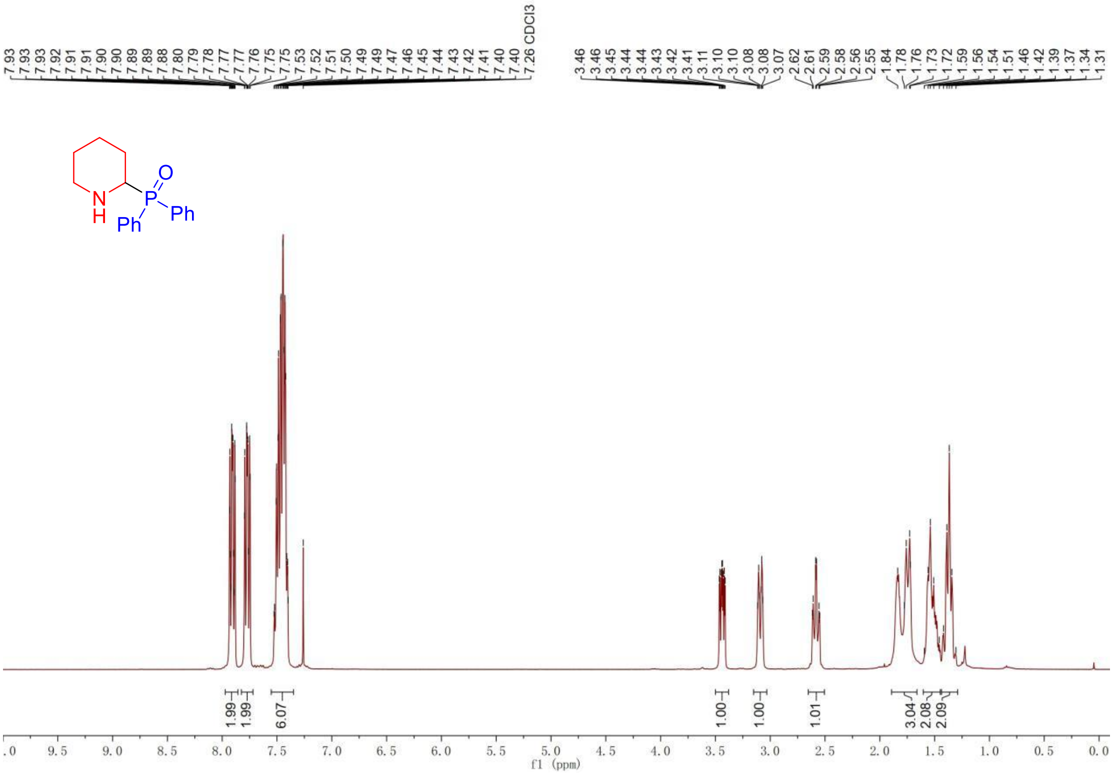

Figure S103. $^{13}C\{^{1}H\}$ **NMR spectra (100 MHz, Chloroform-*d*) of Diphenyl(piperidin-2-yl)phosphine oxide (3ca).**

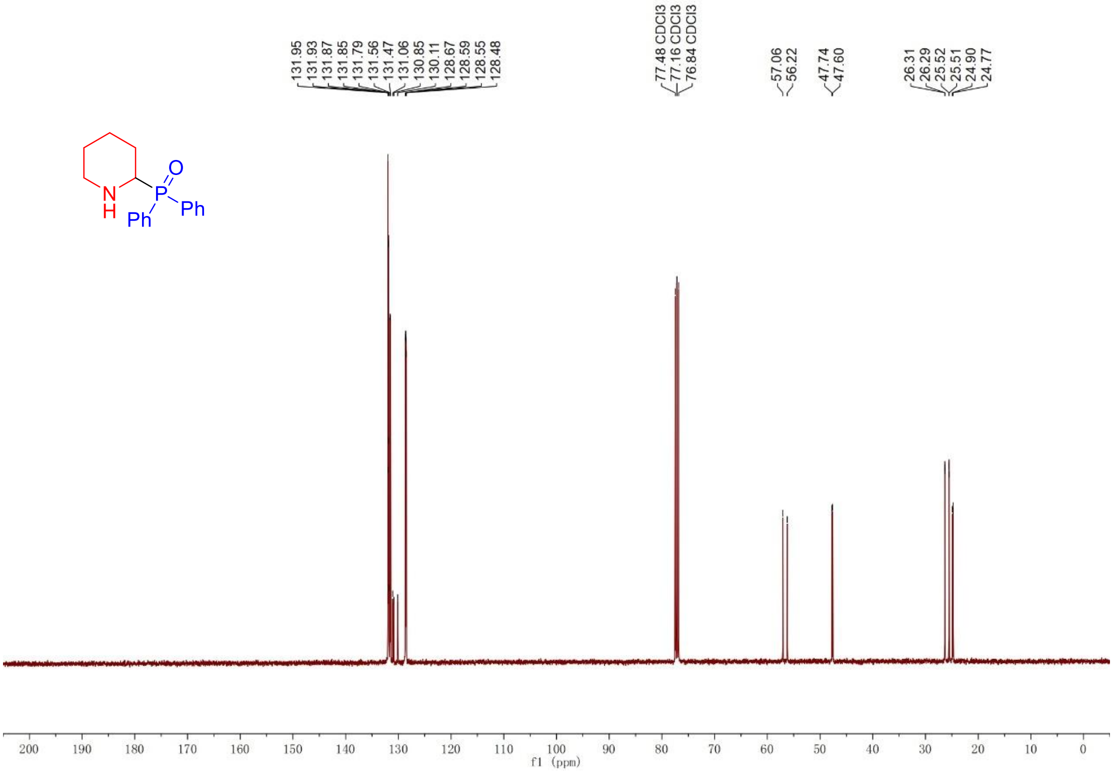

Figure S104. ^{31}P **NMR spectra (162 MHz, Chloroform-*d*) of Diphenyl(piperidin-2-yl)phosphine oxide (3ca).**

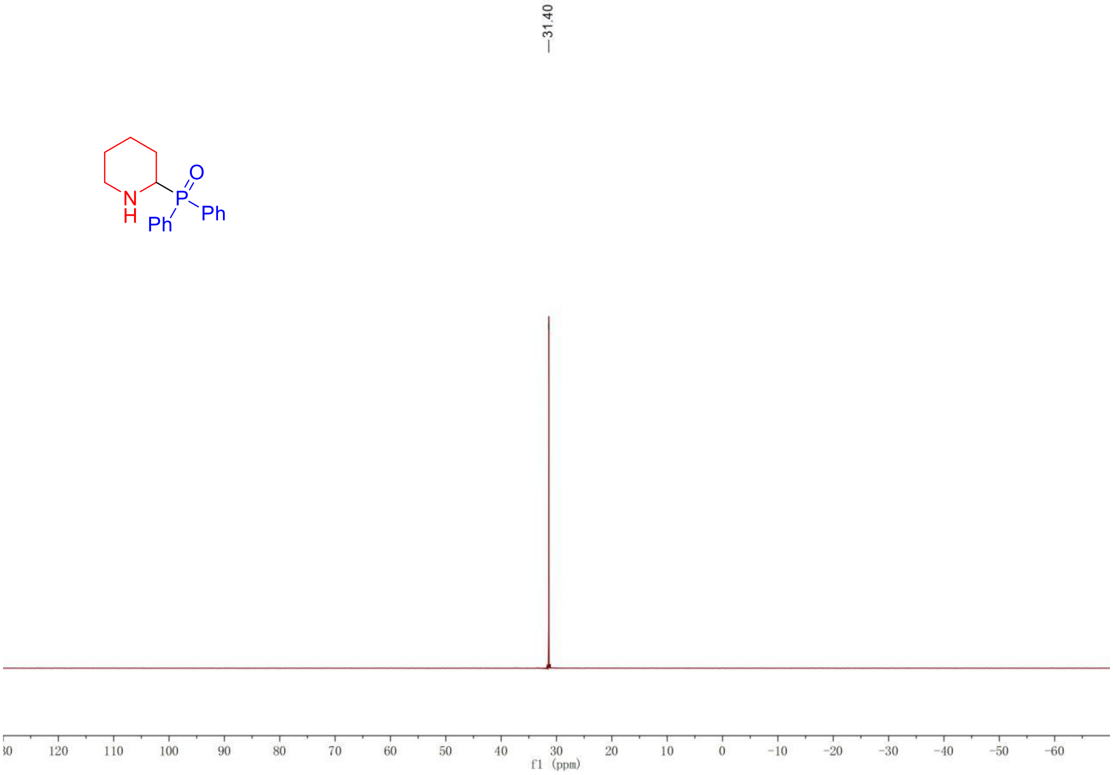

Figure S105. ^{1}H **NMR spectra (400 MHz, Chloroform-*d*) of Diphenyl(thiomorpholin-3-yl)phosphine oxide (3da).**

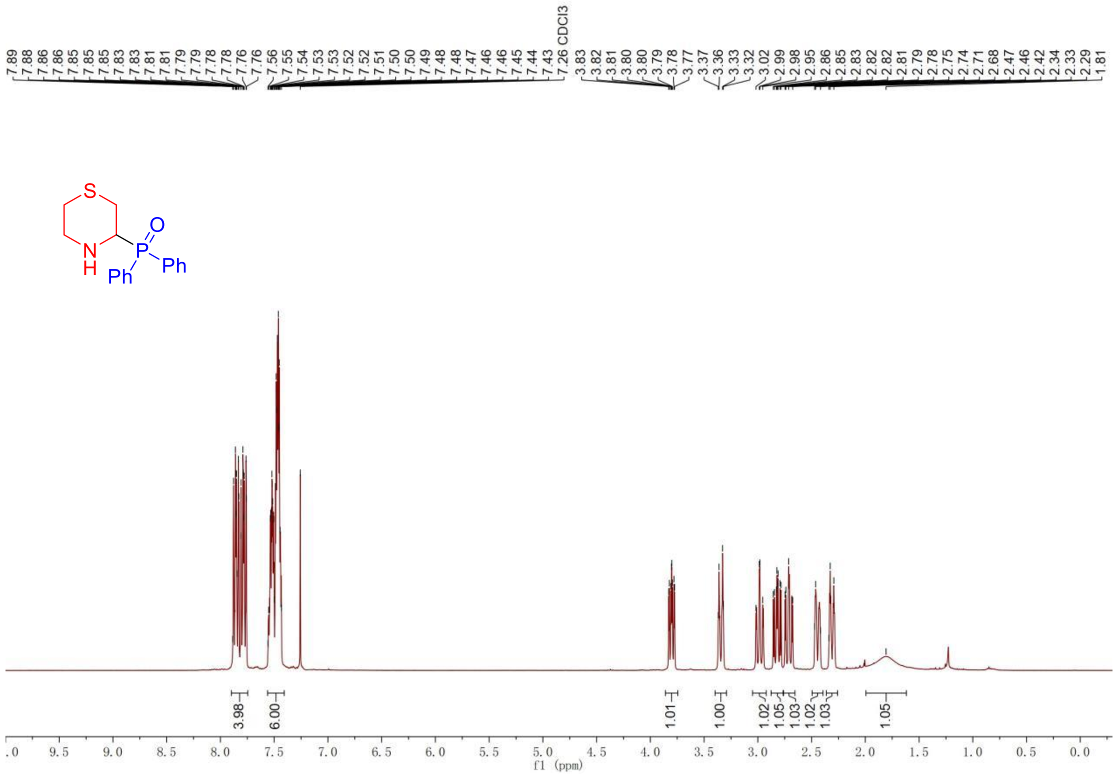

Figure S106. $^{13}C\{^{1}H\}$ **NMR spectra (100 MHz, Chloroform-*d*) of Diphenyl(thiomorpholin-3-yl)phosphine oxide (3da).**

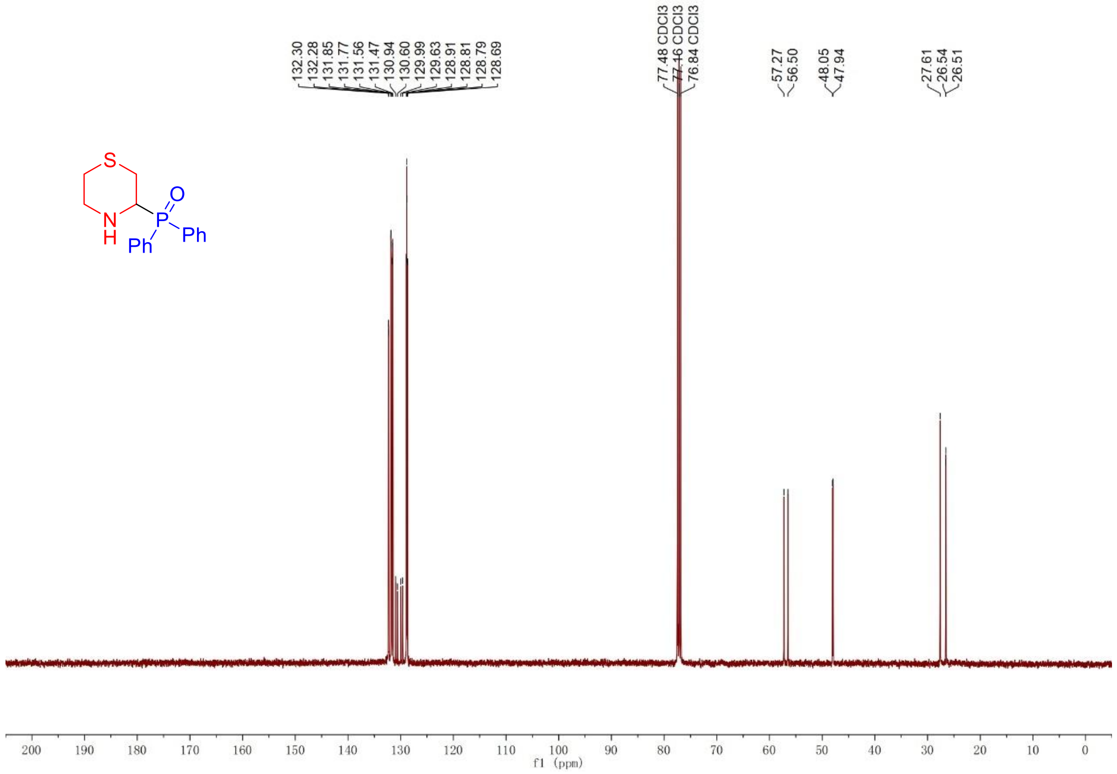

Figure S107. ^{31}P NMR spectra (162 MHz, Chloroform-*d*) of Diphenyl(thiomorpholin-3-yl)phosphine oxide (3da).

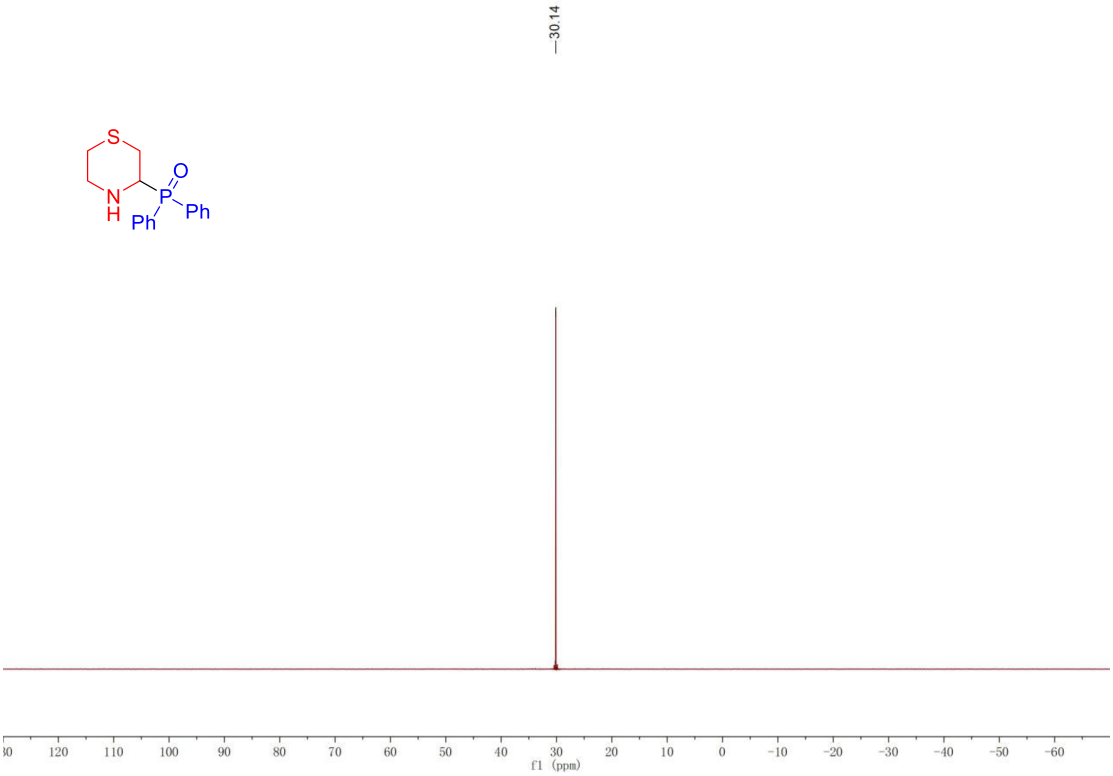

Figure S108. ^{1}H NMR spectra (400 MHz, Chloroform-*d*) of Azepan-2-yldiphenylphosphine oxide (3ea).

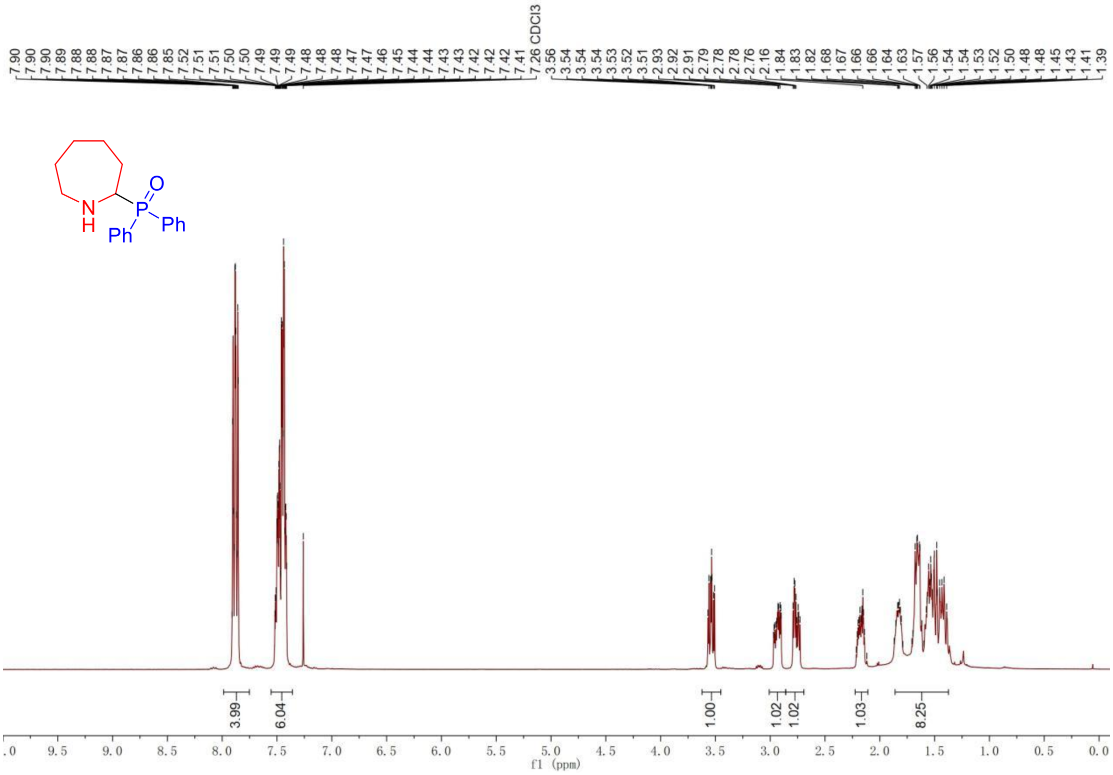

Figure S109. $^{13}C\{^{1}H\}$ **NMR spectra (100 MHz, Chloroform-*d*) of Azepan-2-yldiphenylphosphine oxide (3ea).**

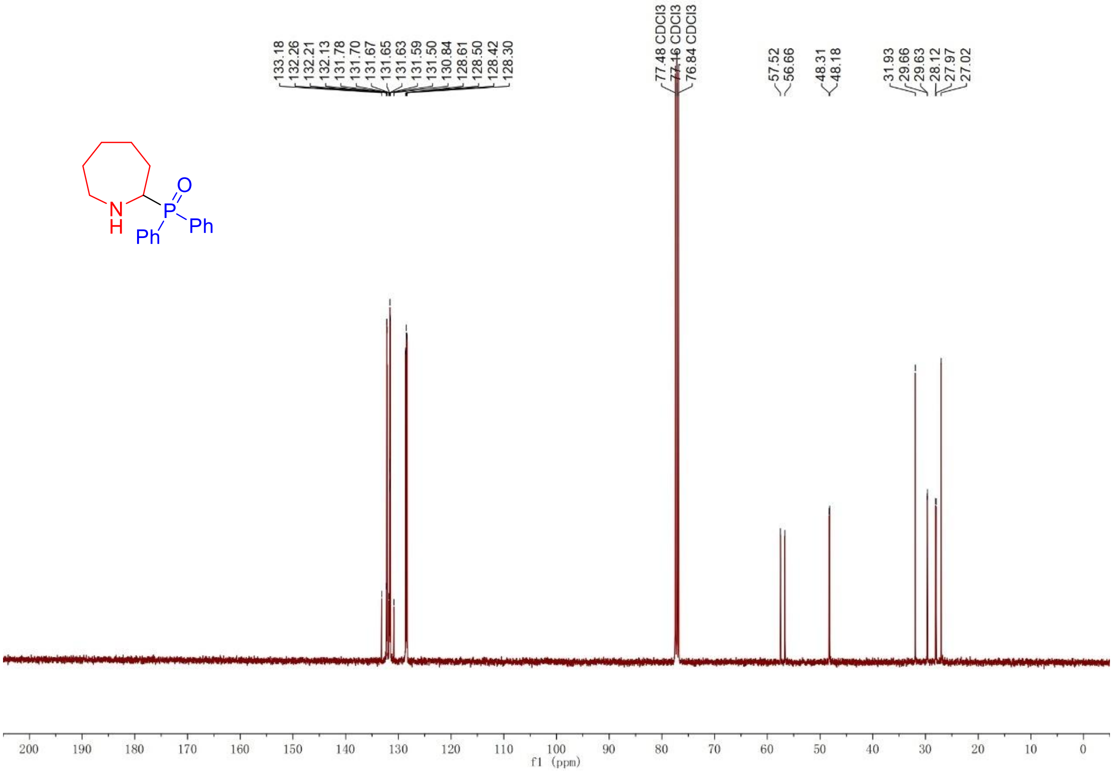

Figure S110. ^{31}P **NMR spectra (162 MHz, Chloroform-*d*) of Azepan-2-yldiphenylphosphine oxide (3ea).**

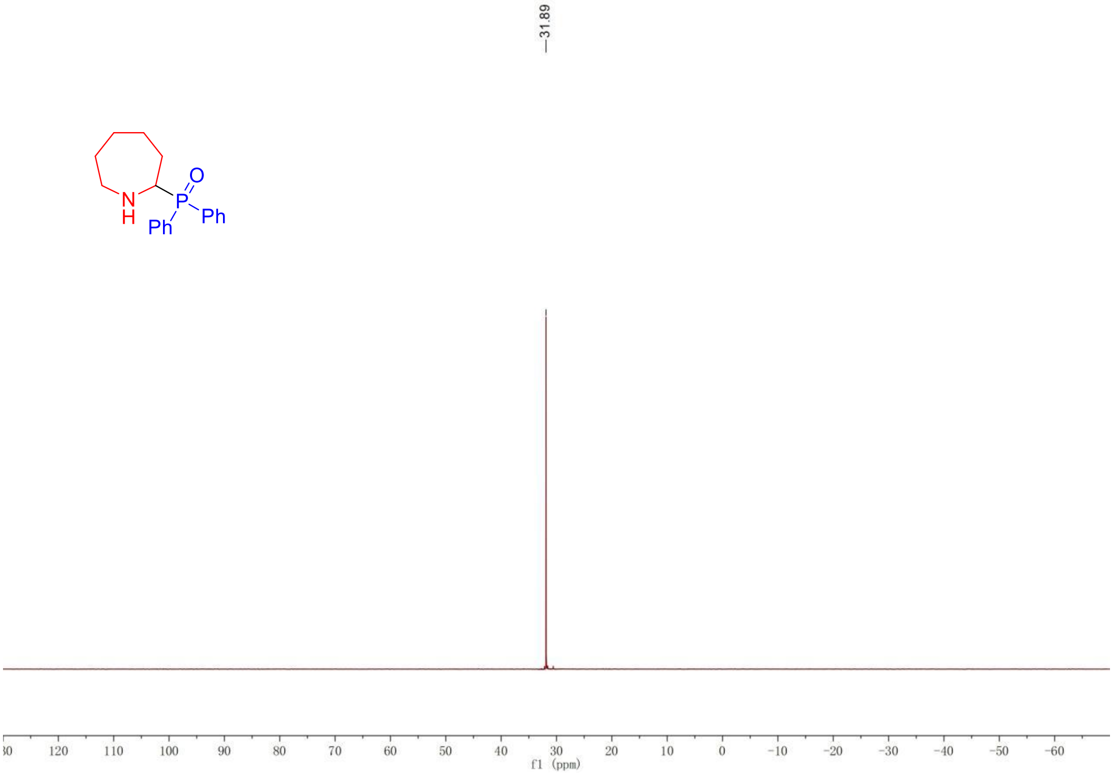

Figure S111. ^{1}**H NMR spectra (400 MHz, Chloroform-*d*) of (4,4-Dimethylpiperidin-2-yl)diphenylphosphine oxide (3fa).**

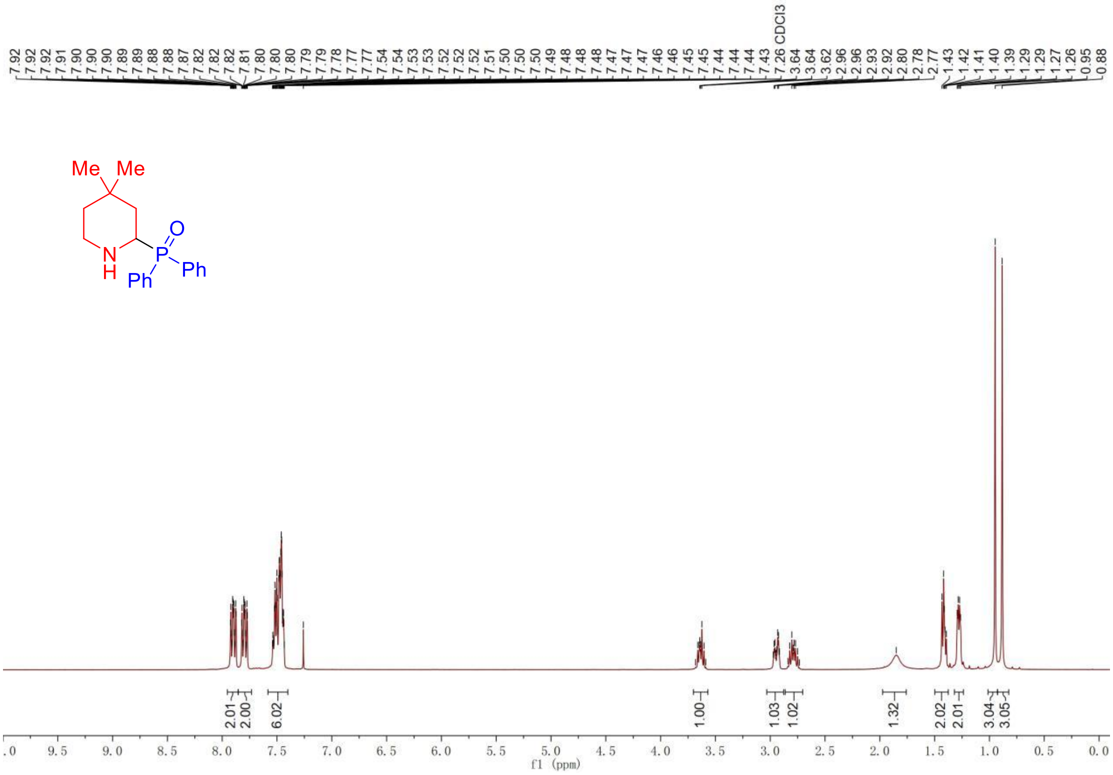

Figure S112. ^{13}**C{**^{1}**H} NMR spectra (100 MHz, Chloroform-*d*) of (4,4-Dimethylpiperidin-2-yl)diphenylphosphine oxide (3fa).**

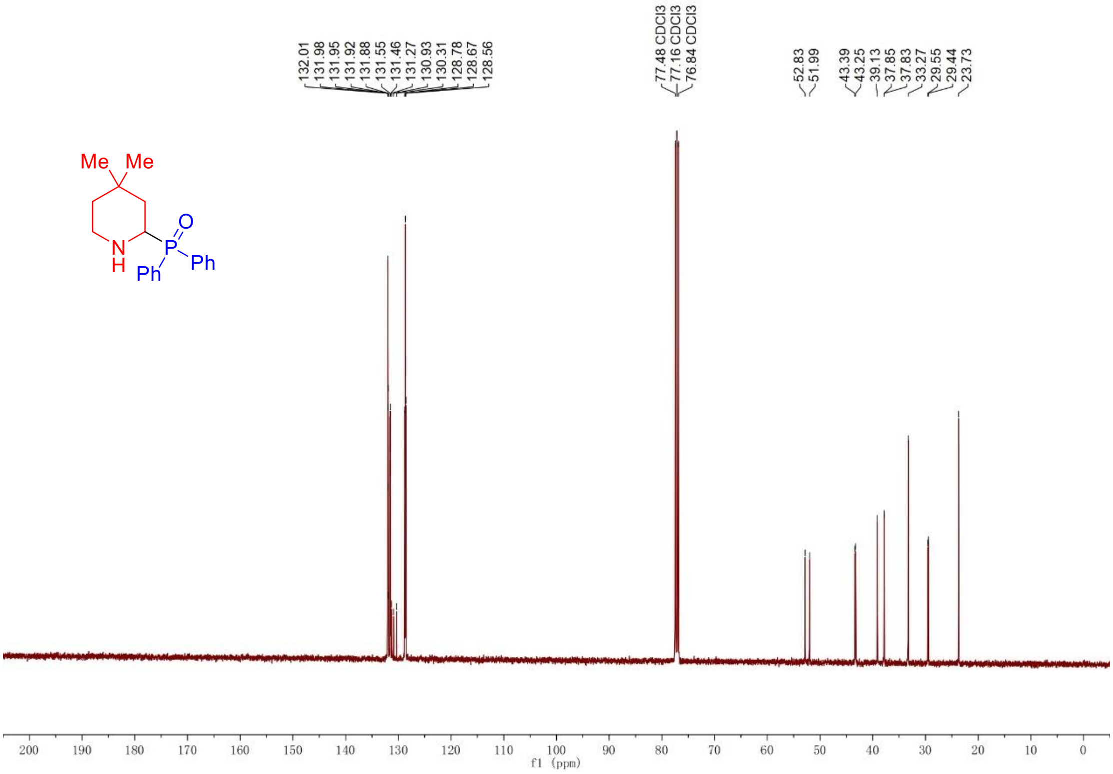

Figure S113. ^{31}P **NMR spectra (162 MHz, Chloroform-*d*) of (4,4-Dimethylpiperidin-2-yl)diphenylphosphine oxide (3fa).**

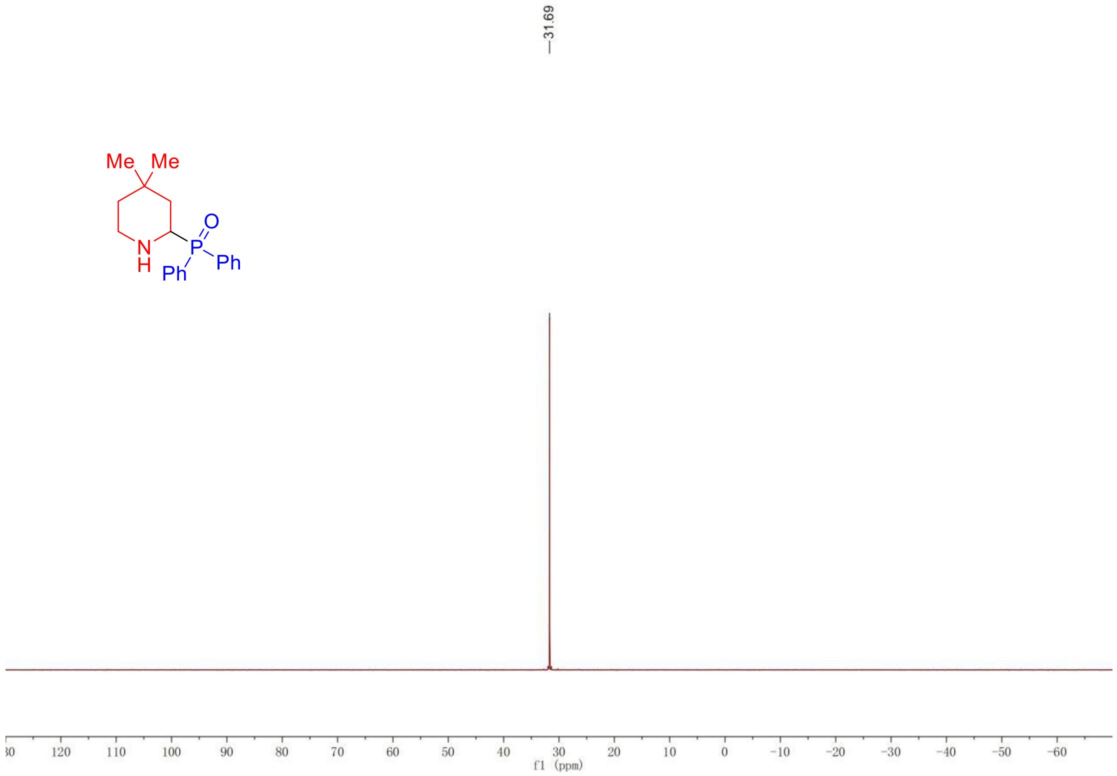

Figure S114. ^{1}H **NMR spectra (400 MHz, Chloroform-*d*) of Diphenyl(1,4-dioxa-8-azaspiro[4.5]decan-7-yl)phosphine oxide (3ga).**

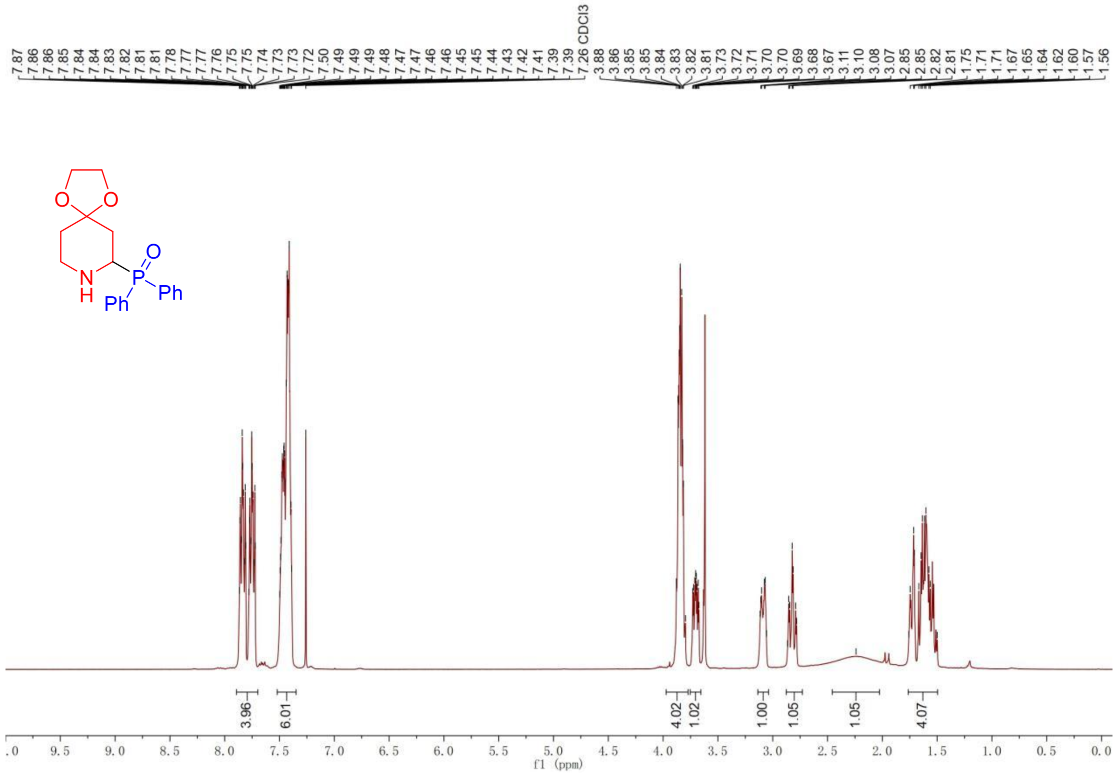

Figure S115. $^{13}C\{^{1}H\}$ **NMR spectra (100 MHz, Chloroform-*d*) of Diphenyl(1,4-dioxa-8-azaspiro[4.5]decan-7-yl)phosphine oxide (3ga).**

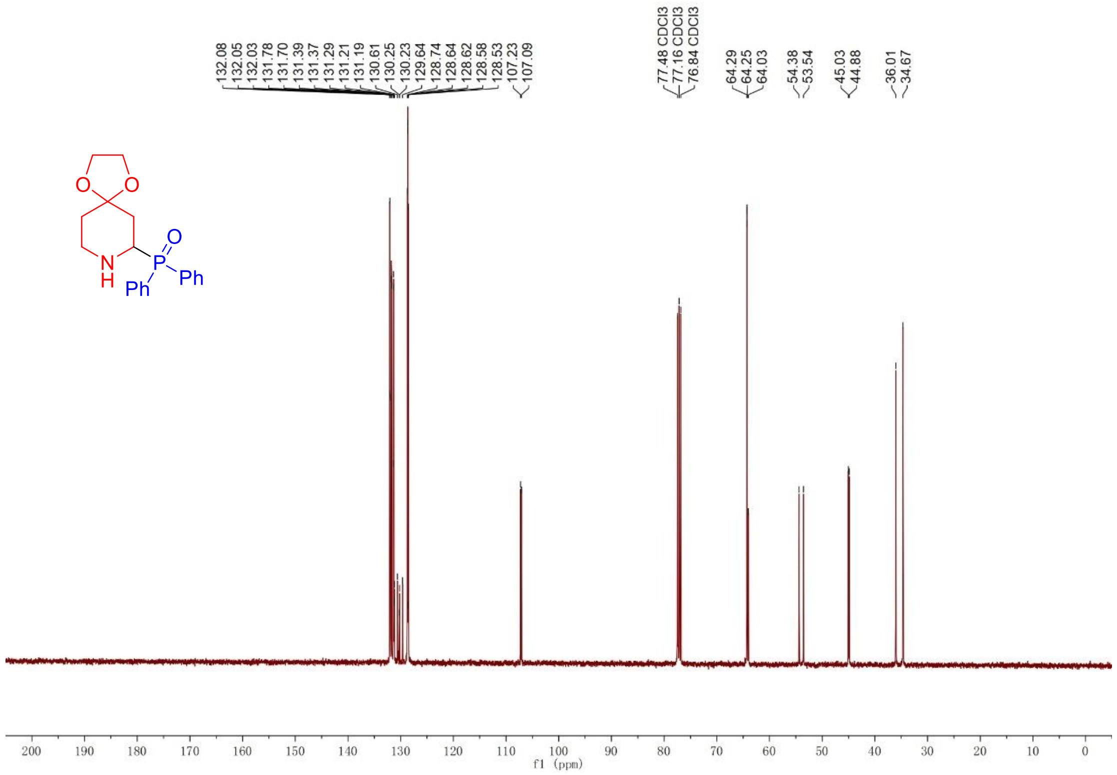

Figure S116. ^{31}P **NMR spectra (162 MHz, Chloroform-*d*) of Diphenyl(1,4-dioxa-8-azaspiro[4.5]decan-7-yl)phosphine oxide (3ga).**

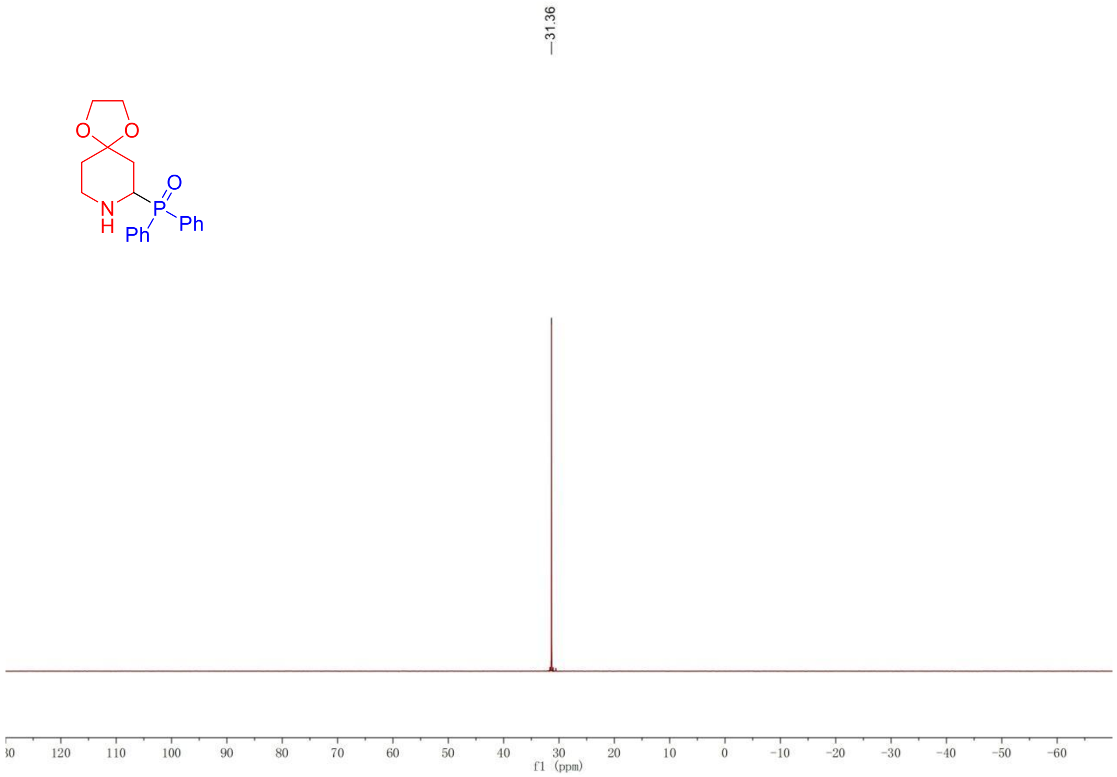

Figure S117. ^{1}**H NMR spectra (400 MHz, Chloroform-*d*) of ((3a*R*,7a*S*)-Octahydro-1*H*-isoindol-1-yl)diphenylphosphine oxide (3ha).**

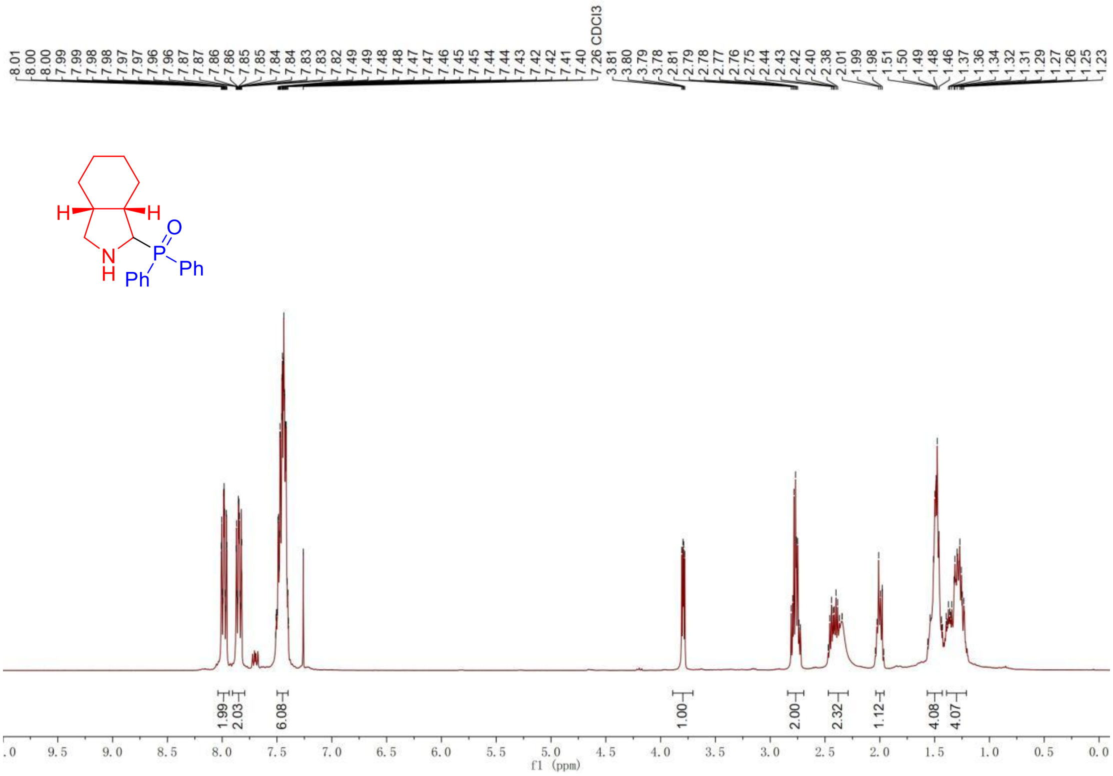

Figure S118. ^{13}**C{**^{1}**H} NMR spectra (100 MHz, Chloroform-*d*) of ((3a*R*,7a*S*)-Octahydro-1*H*-isoindol-1-yl)diphenylphosphine oxide (3ha).**

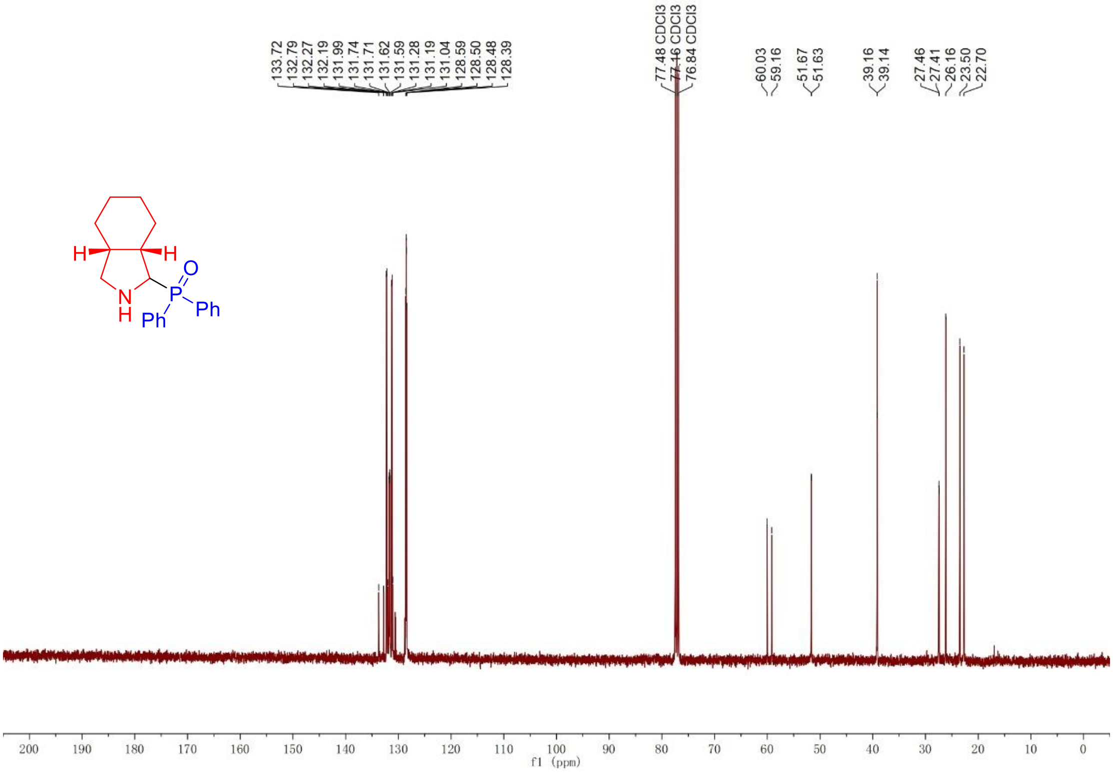

Figure S119. ^{31}P NMR spectra (162 MHz, Chloroform-*d*) of ((3a*R*,7a*S*)-Octahydro-1*H*-isoindol-1-yl)diphenylphosphine oxide (3ha).

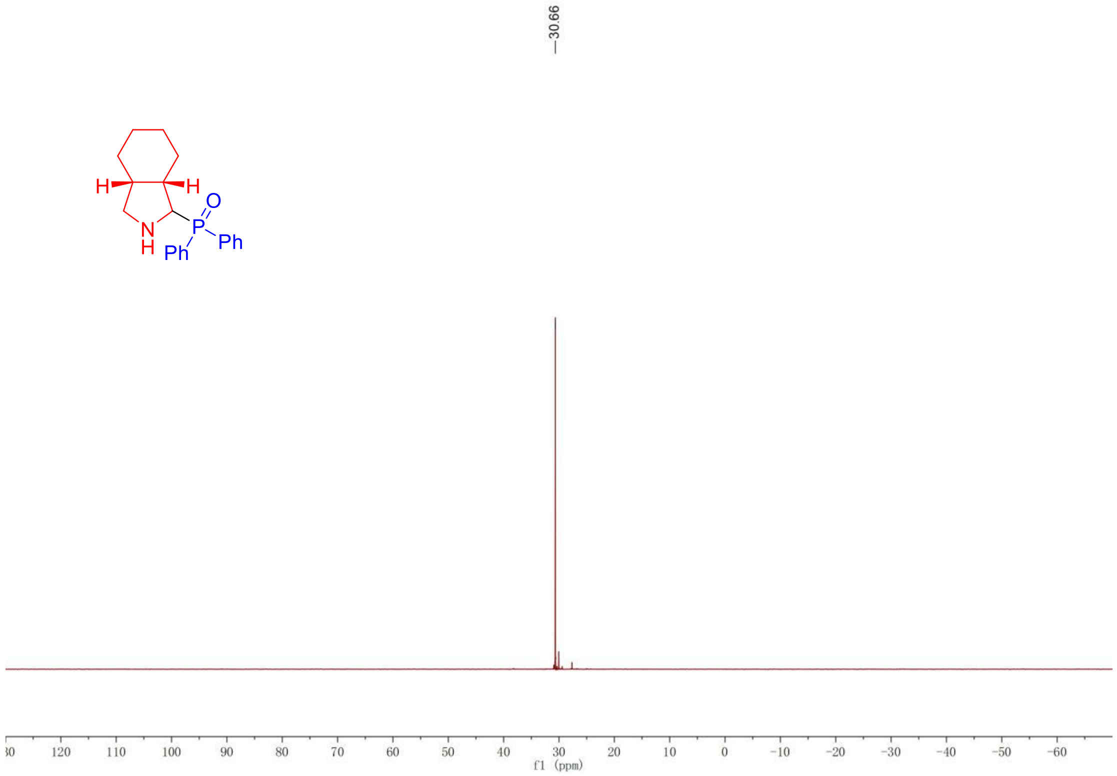

Figure S120. ^{1}H NMR spectra (400 MHz, Chloroform-*d*) of ((Methylamino)methyl)diphenylphosphine oxide (3ia).

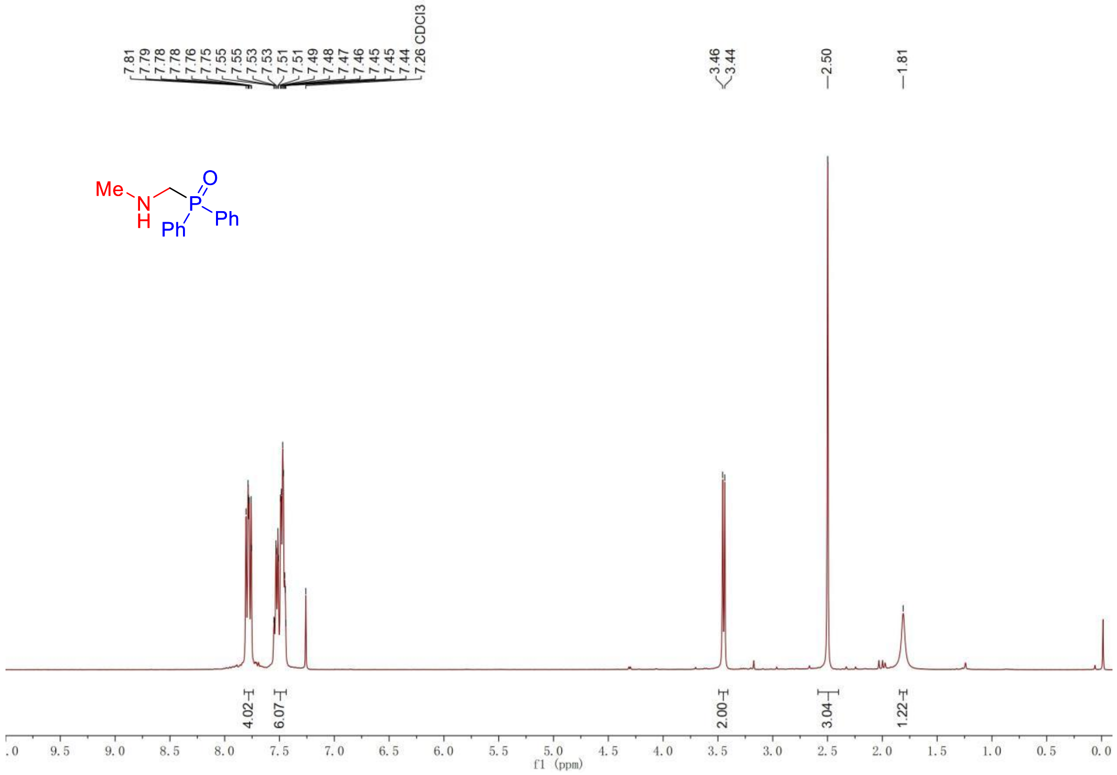

Figure S121. $^{13}C\{^1H\}$ **NMR spectra (100 MHz, Chloroform-*d*) of ((Methylamino)methyl)diphenylphosphine oxide (3ia).**

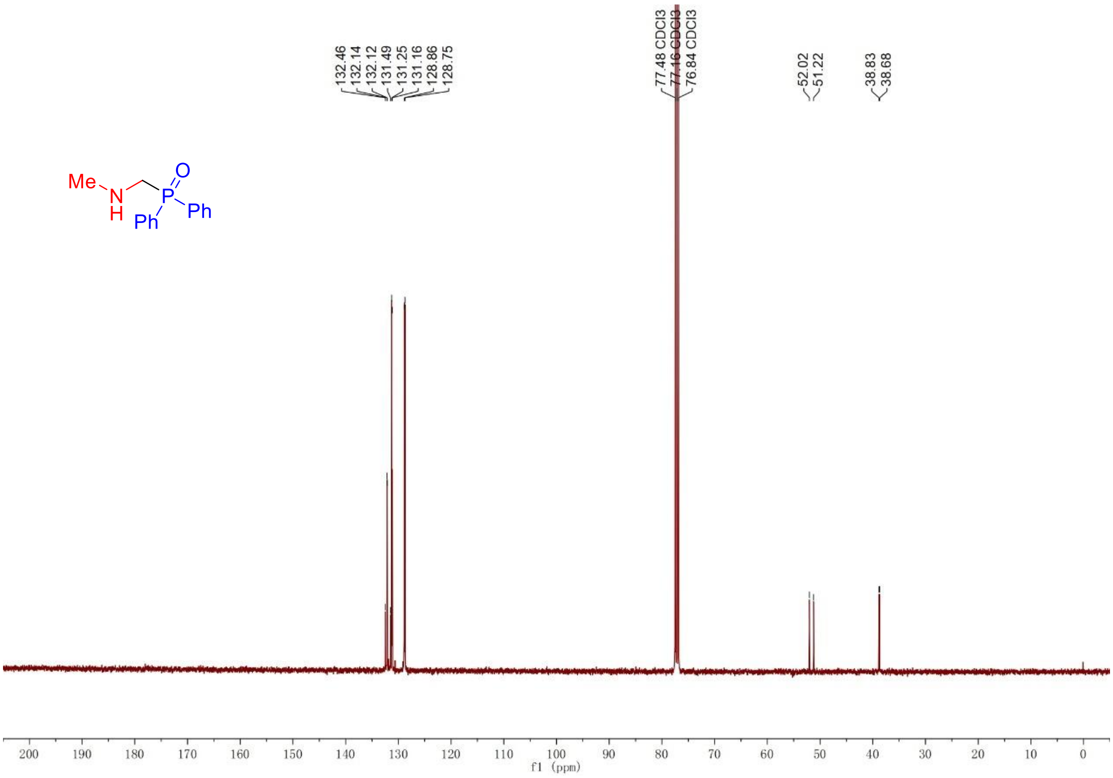

Figure S122. ^{31}P **NMR spectra (162 MHz, Chloroform-*d*) of ((Methylamino)methyl)diphenylphosphine oxide (3ia).**

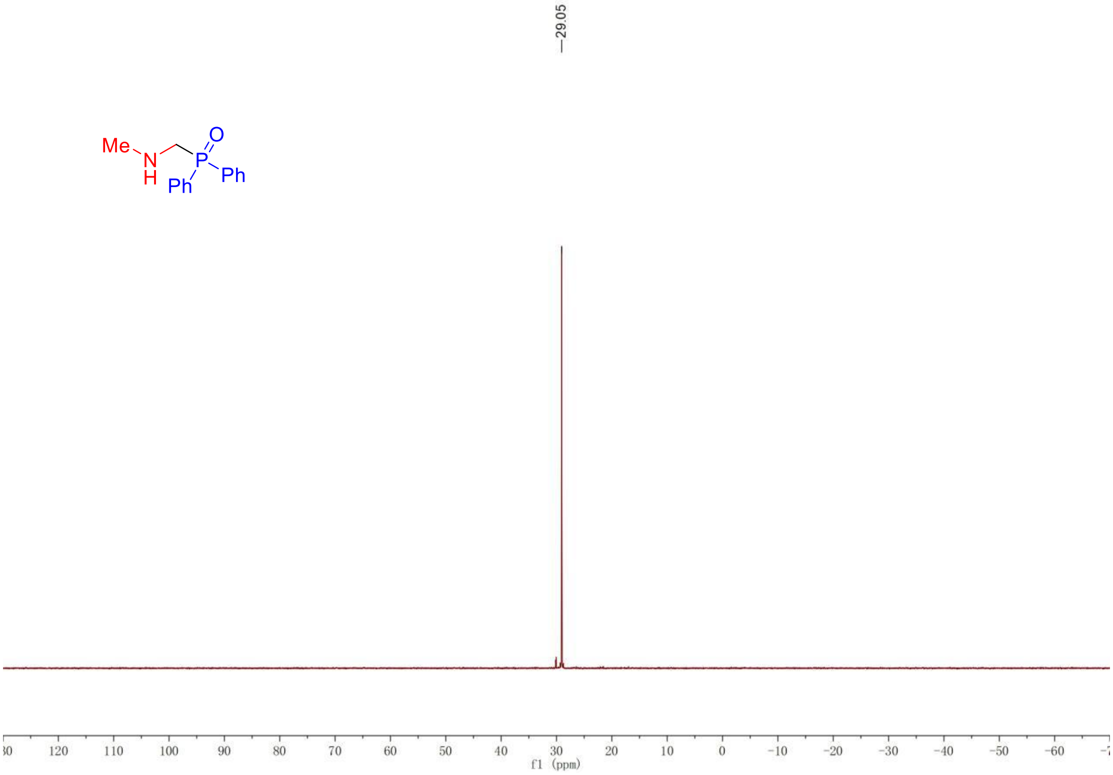

Figure S123. ^{1}**H NMR spectra (400 MHz, Chloroform-*d*) of (((methyl-*d*$_3$)amino)methyl-*d*$_2$)diphenylphosphine oxide (*d*-3ia).**

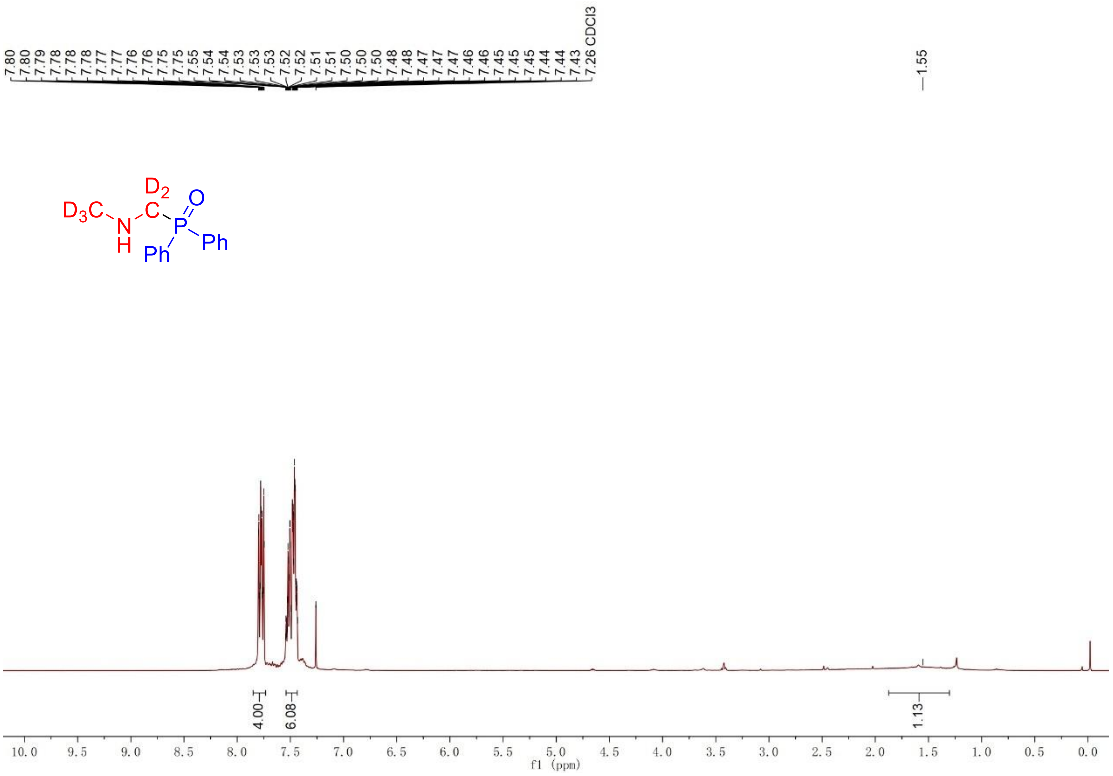

Figure S124. ^{13}**C{**^{1}**H} NMR spectra (100 MHz, Chloroform-*d*) of (((methyl-*d*$_3$)amino)methyl-*d*$_2$)diphenylphosphine oxide (*d*-3ia).**

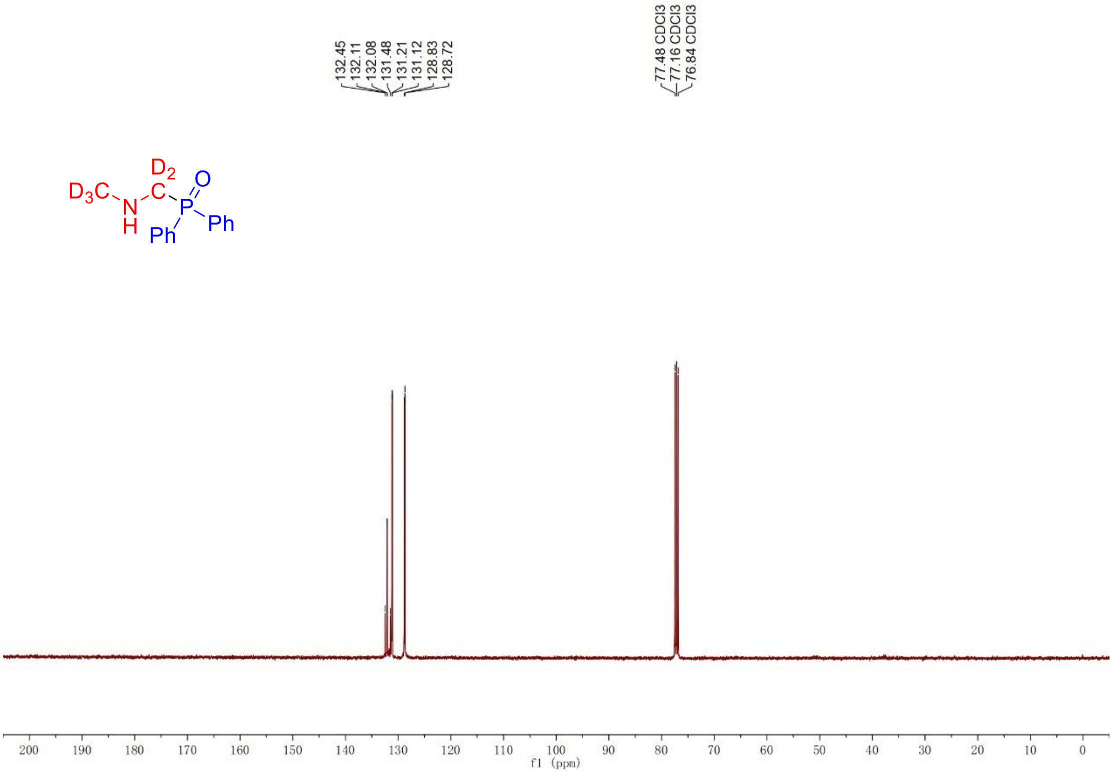

Figure S125. ^{31}**P NMR spectra (162 MHz, Chloroform-*d*) of (((methyl-*d*$_3$)amino)methyl-*d*$_2$)diphenylphosphine oxide (*d*-3ia).**

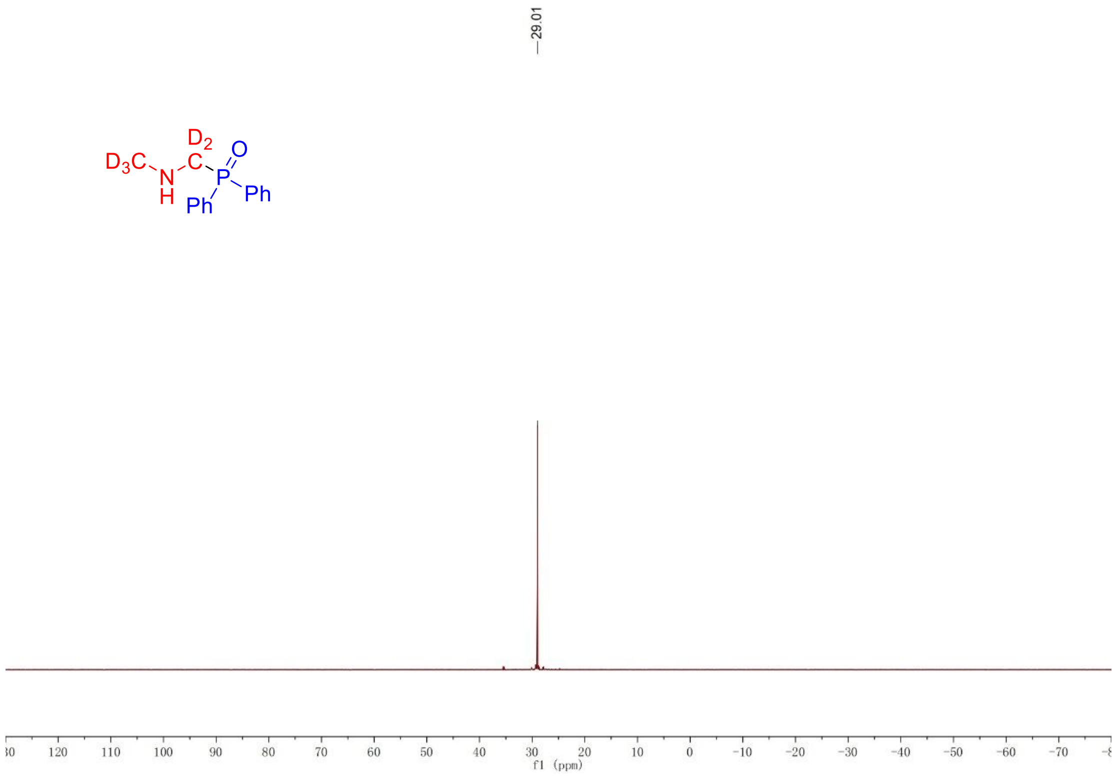

Figure S126. ^{1}**H NMR spectra (400 MHz, Chloroform-*d*) of (1-(Ethylamino)ethyl)diphenylphosphine oxide (3ja).**

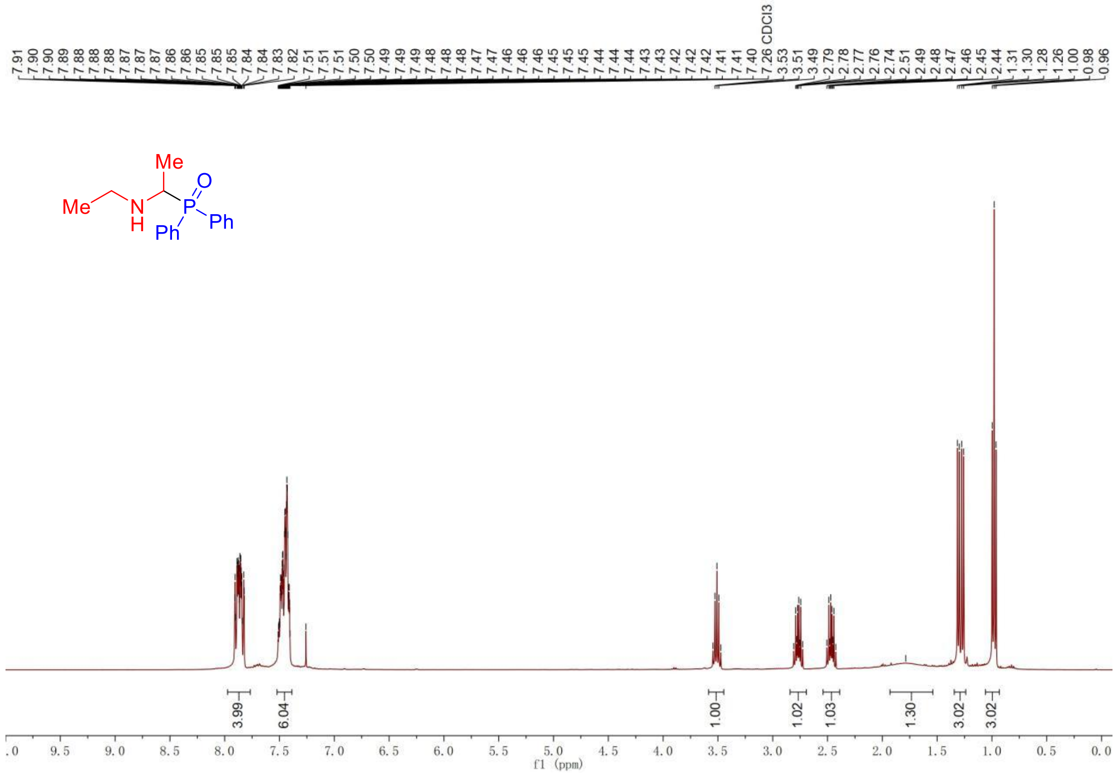

Figure S127. $^{13}C\{^1H\}$ **NMR spectra (100 MHz, Chloroform-*d*) of (1-(Ethylamino)ethyl)diphenylphosphine oxide (3ja).**

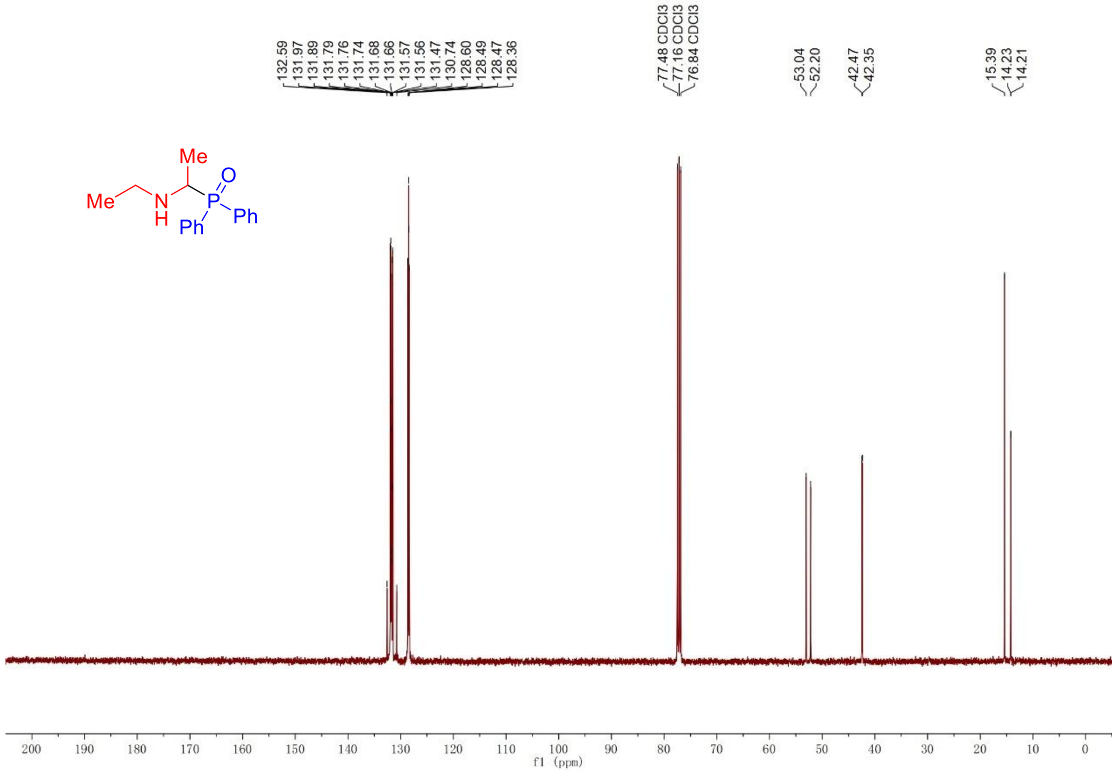

Figure S128. ^{31}P **NMR spectra (162 MHz, Chloroform-*d*) of (1-(Ethylamino)ethyl)diphenylphosphine oxide (3ja).**

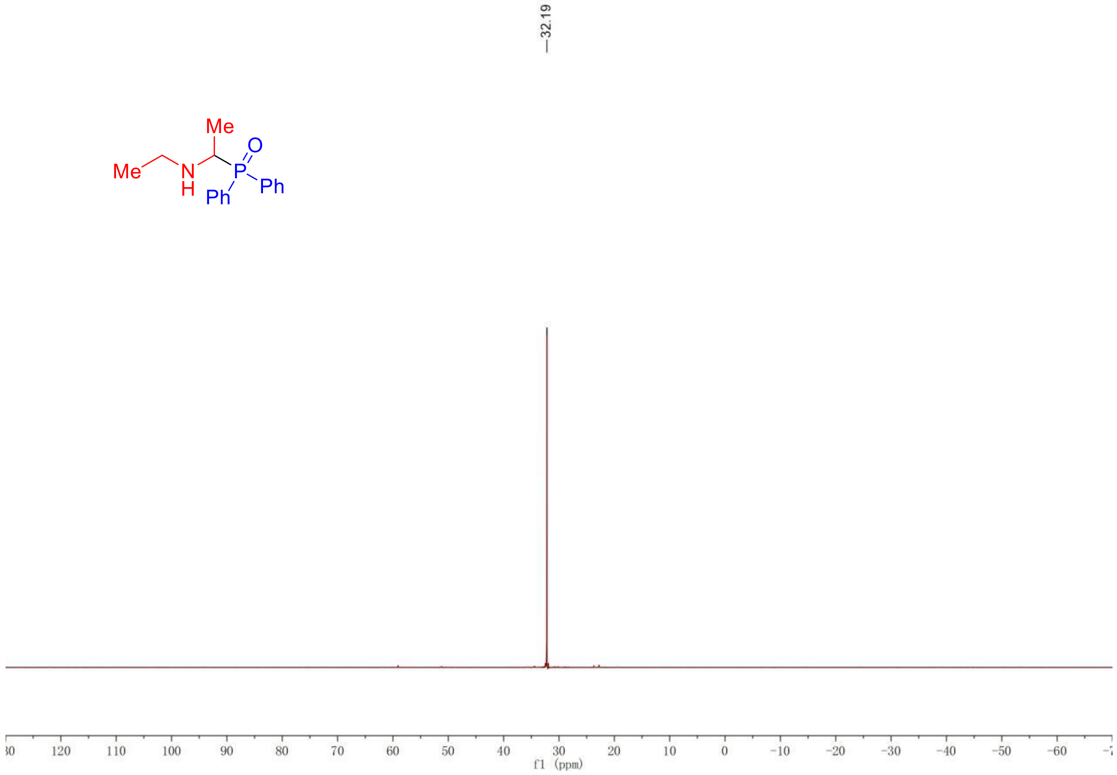

Figure S129. ^{1}H **NMR spectra (400 MHz, Chloroform-*d*) of ((Isopropylamino)methyl)diphenylphosphine oxide (3ka).**

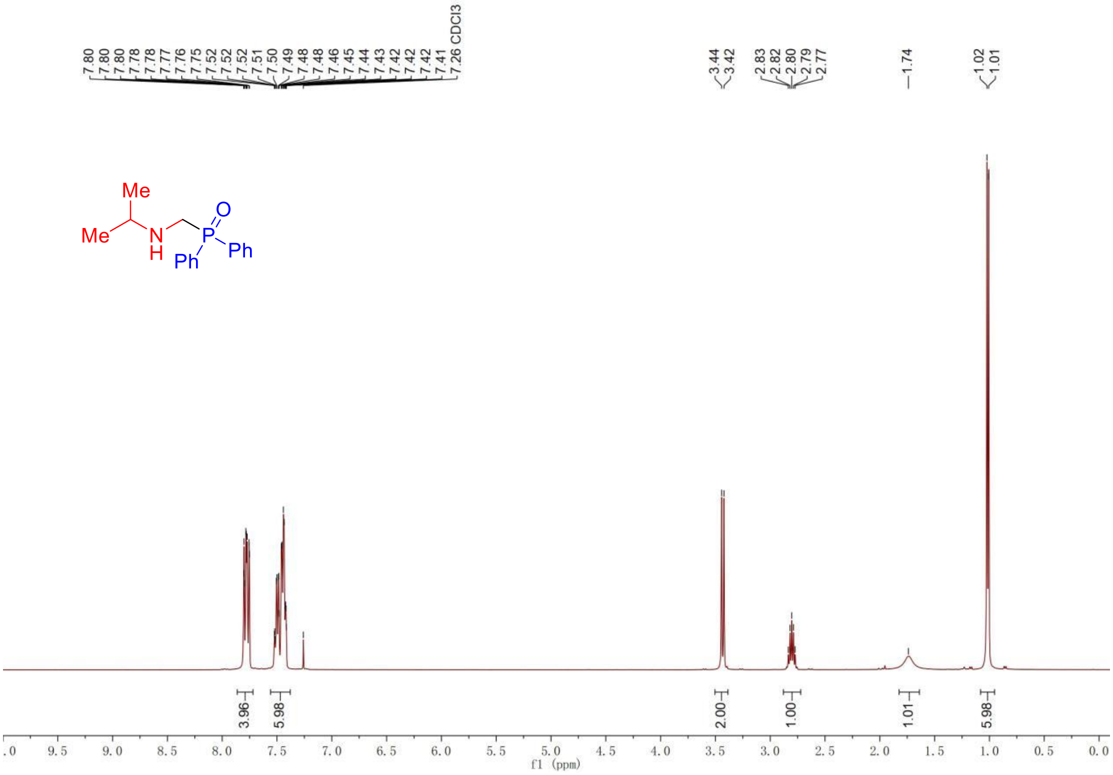

Figure S130. $^{13}C\{^{1}H\}$ **NMR spectra (100 MHz, Chloroform-*d*) of ((Isopropylamino)methyl)diphenylphosphine oxide (3ka).**

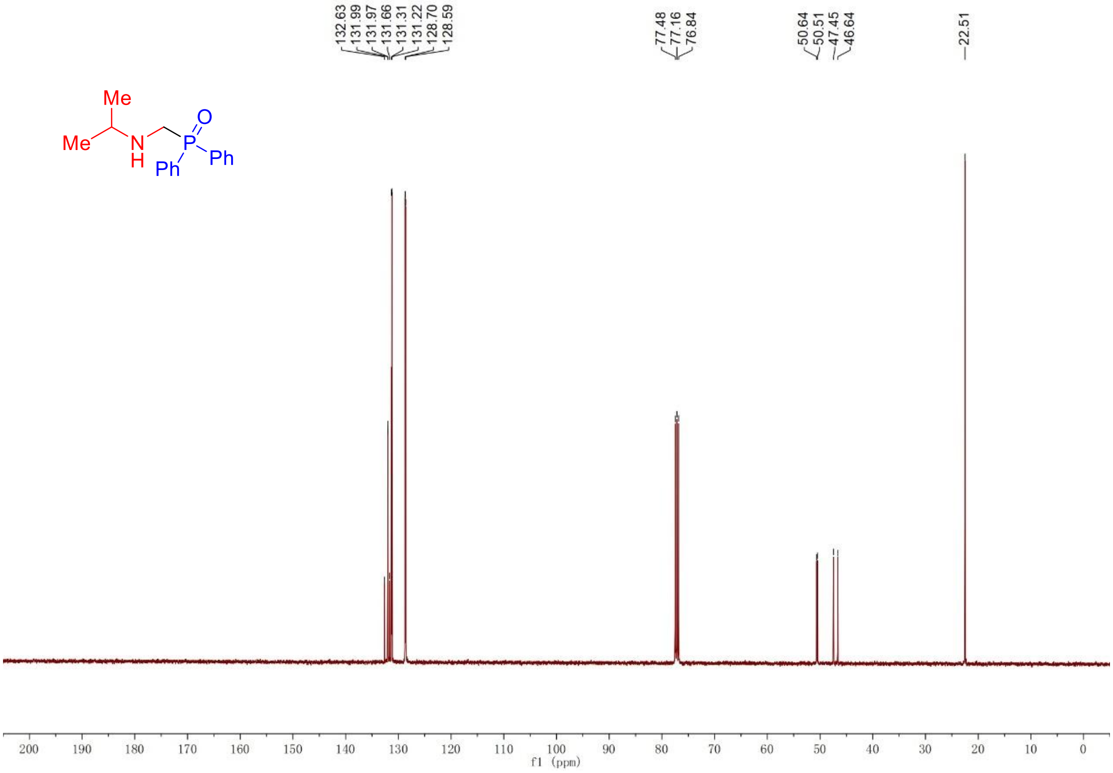

Figure S13. ^{31}P **NMR spectra (162 MHz, Chloroform-*d*) of ((Isopropylamino)methyl)diphenylphosphine oxide (3ka).**

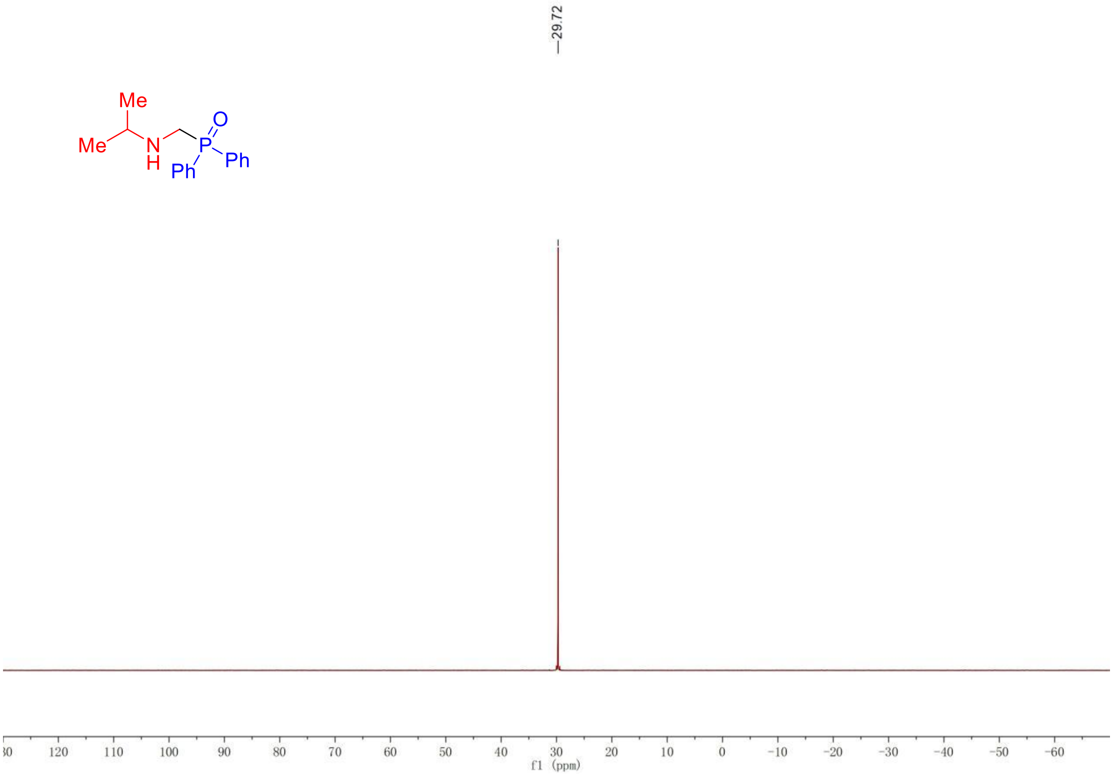

Figure S132. ^{1}H **NMR spectra (400 MHz, Chloroform-*d*) of (1-(Butylamino)butyl)diphenylphosphine oxide (3la).**

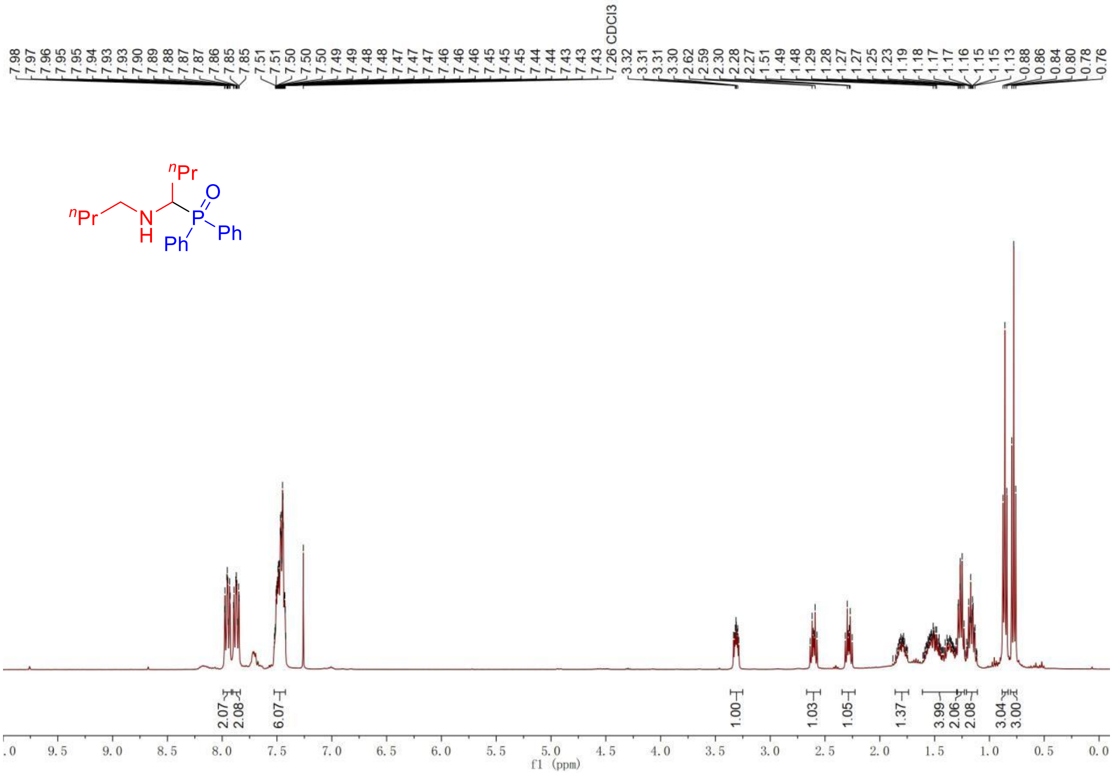

Figure S133. $^{13}C\{^{1}H\}$ **NMR spectra (100 MHz, Chloroform-*d*) of (1-(Butylamino)butyl)diphenylphosphine oxide (3la).**

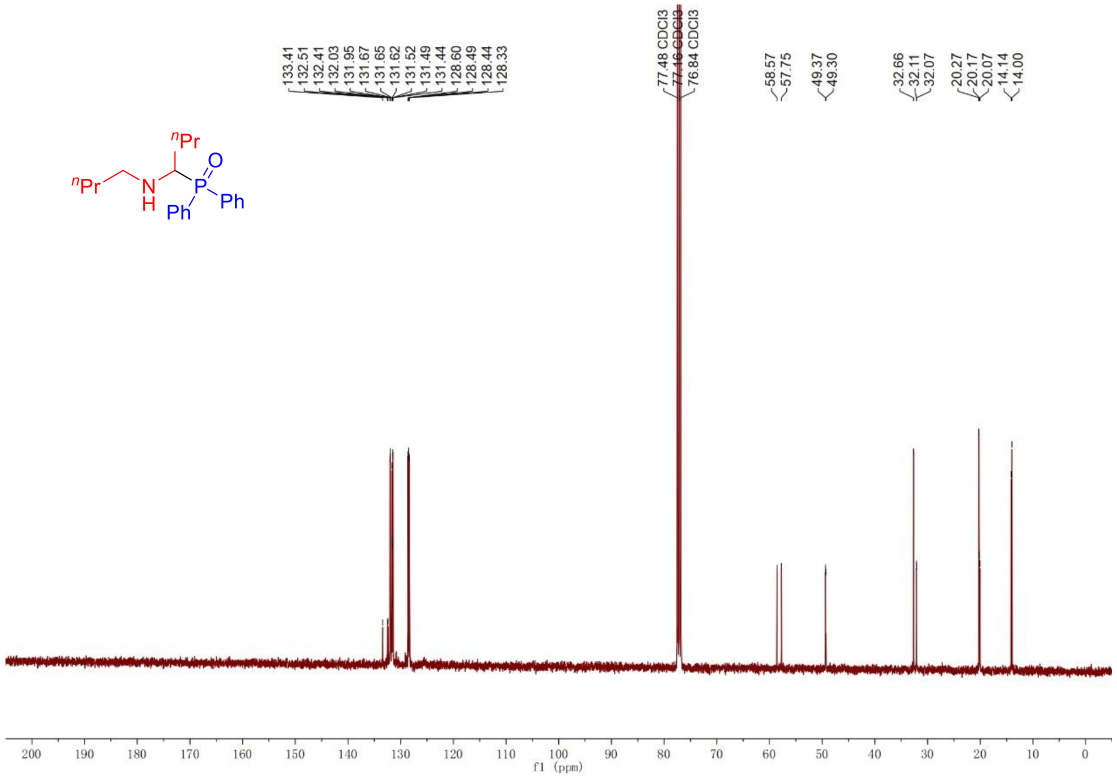

Figure S134. ^{31}P **NMR spectra (162 MHz, Chloroform-*d*) of (1-(Butylamino)butyl)diphenylphosphine oxide (3la).**

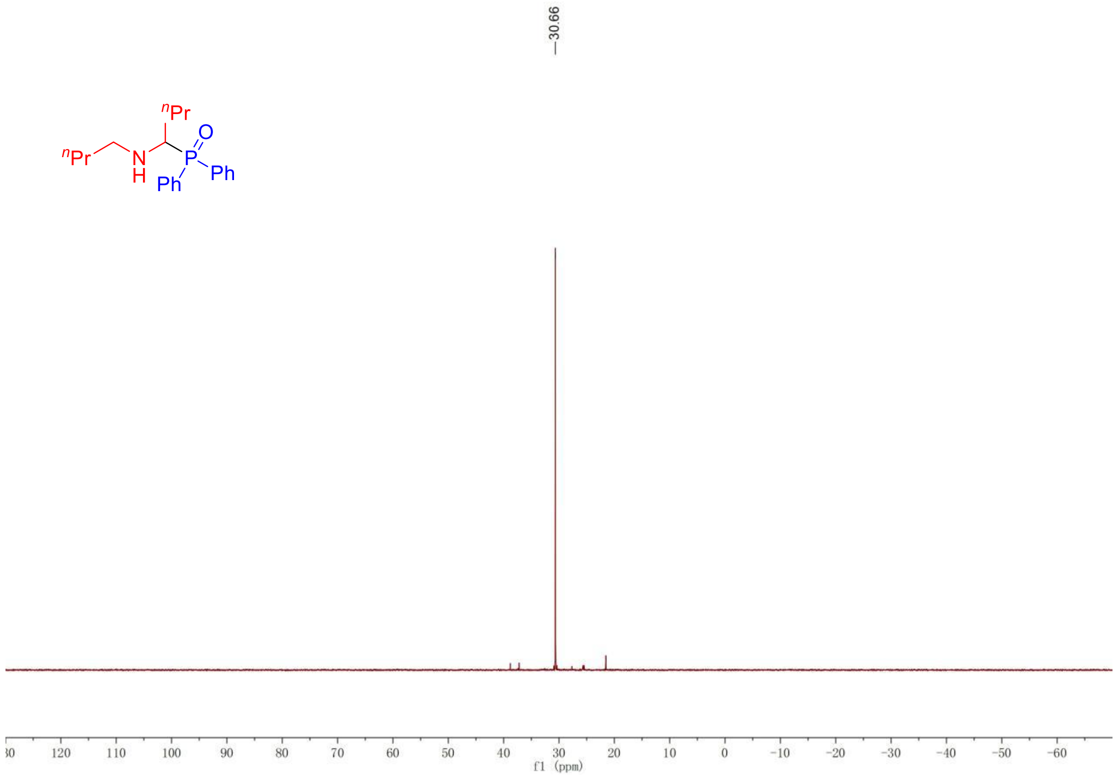

Figure S135. ^{1}H NMR spectra (400 MHz, Chloroform-*d*) of ((Cyclohexylamino)methyl)diphenylphosphine oxide (3ma).

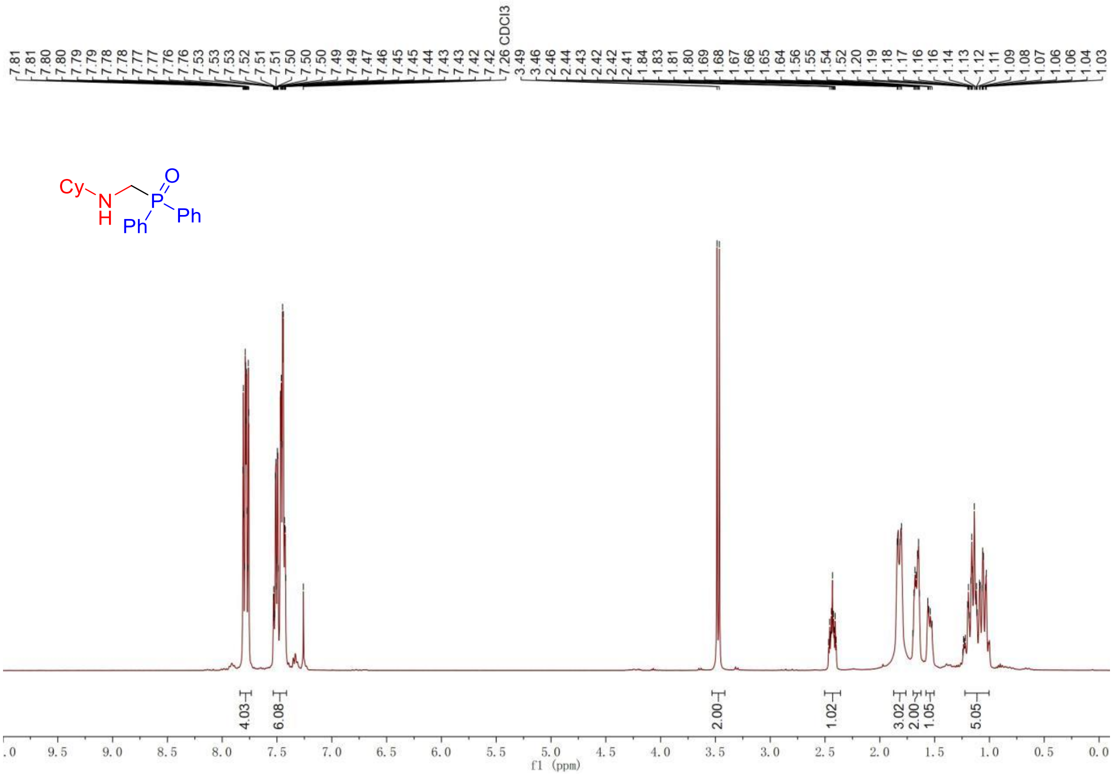

Figure S136. $^{13}C\{^{1}H\}$ NMR spectra (100 MHz, Chloroform-*d*) of ((Cyclohexylamino)methyl)diphenylphosphine oxide (3ma).

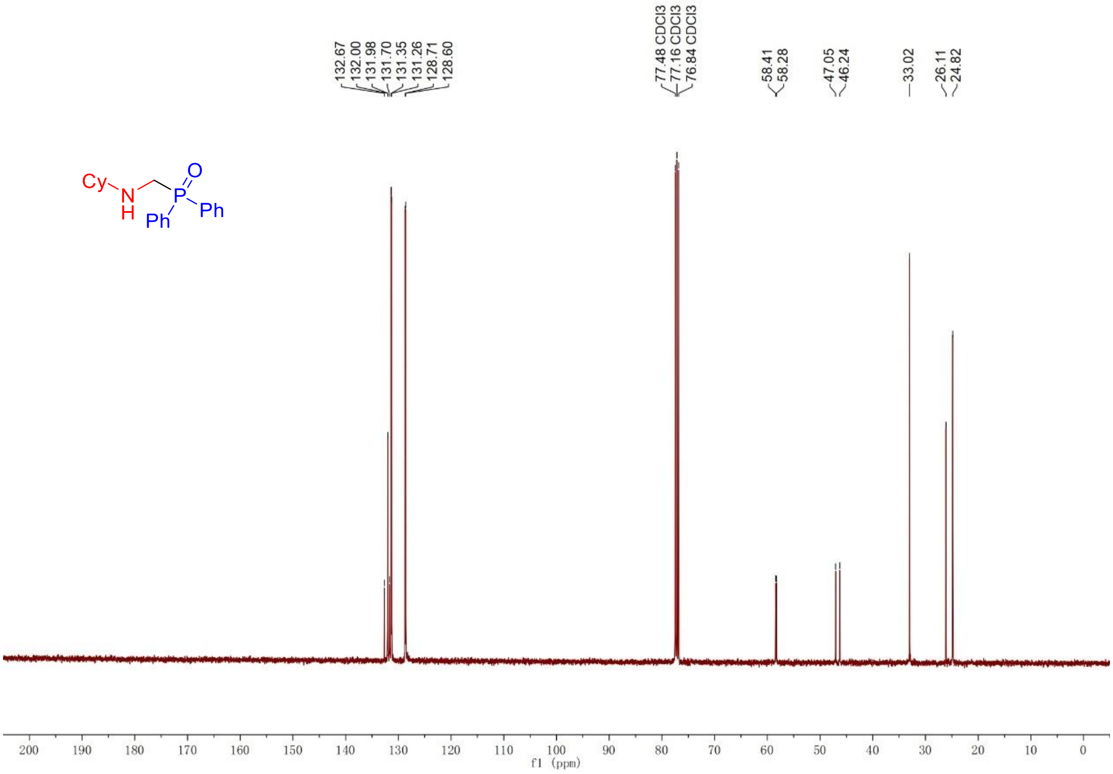

Figure S137. ^{31}P NMR spectra (162 MHz, Chloroform-*d*) of ((Cyclohexylamino)methyl)diphenylphosphine oxide (3ma).

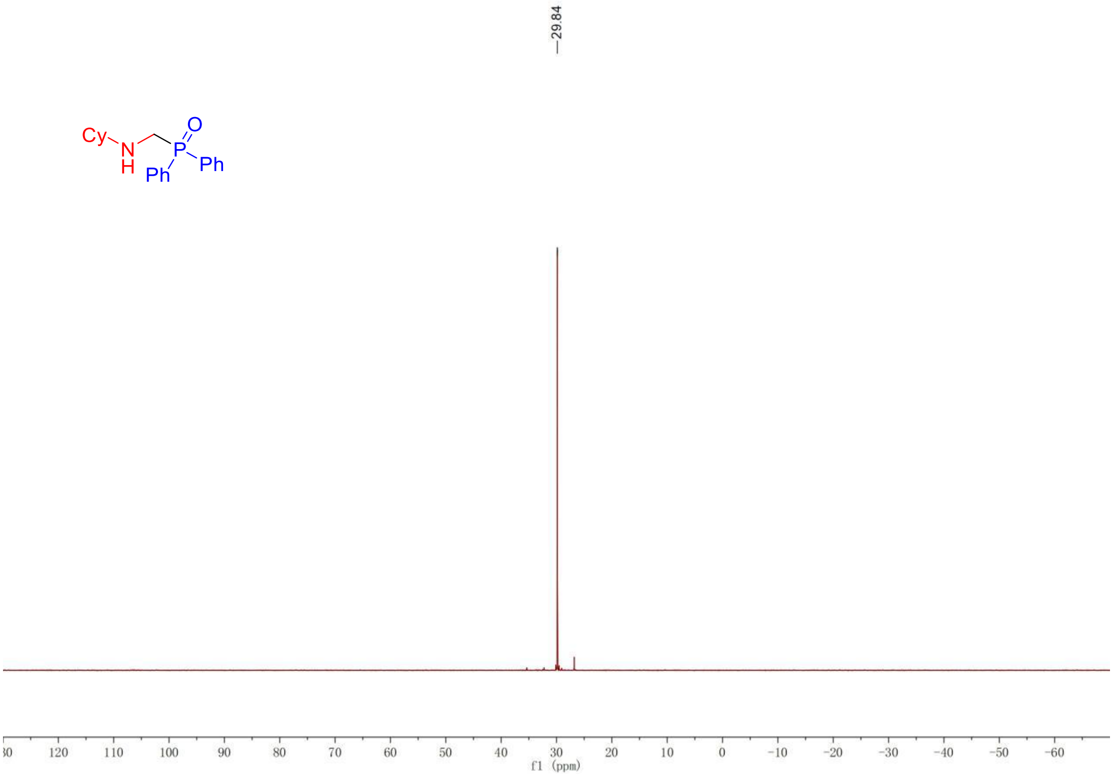

Figure S138. ^{1}H NMR spectra (400 MHz, Chloroform-*d*) of Diphenyl(((tetrahydro-2*H*-pyran-4-yl)amino)methyl)phosphine oxide (3na).

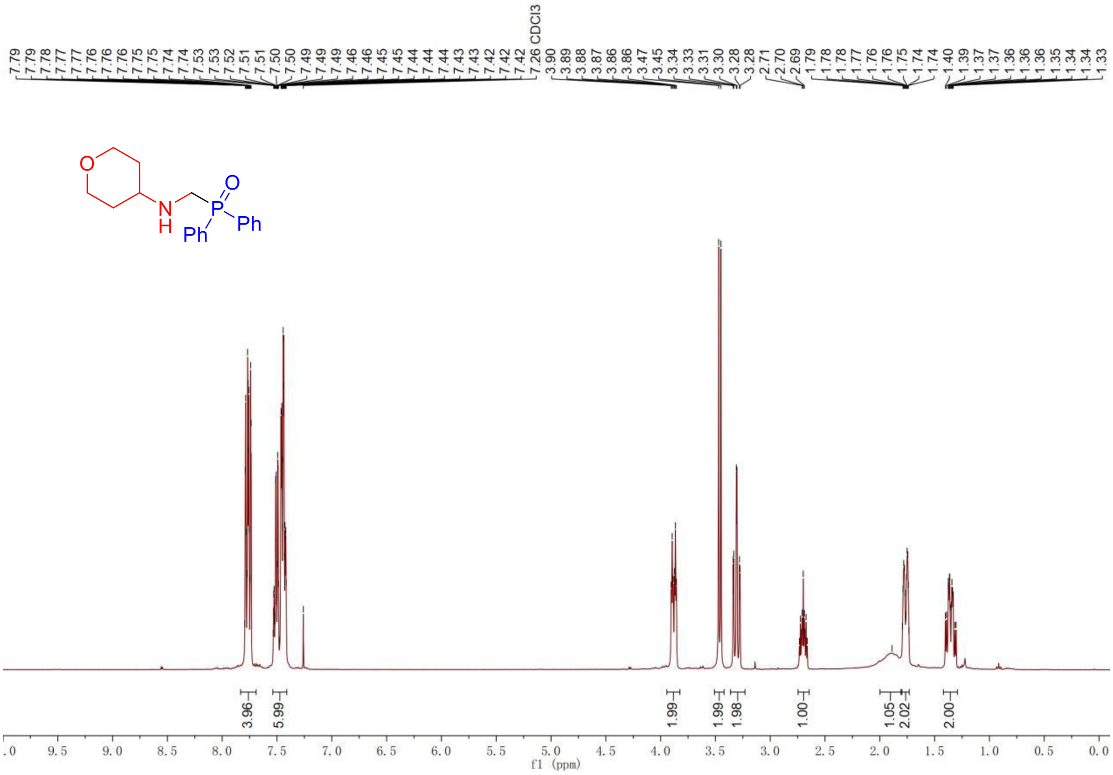

Figure S139. $^{13}C\{^{1}H\}$ NMR spectra (100 MHz, Chloroform-*d*) of Diphenyl(((tetrahydro-2*H*-pyran-4-yl)amino)methyl)phosphine oxide (3na).

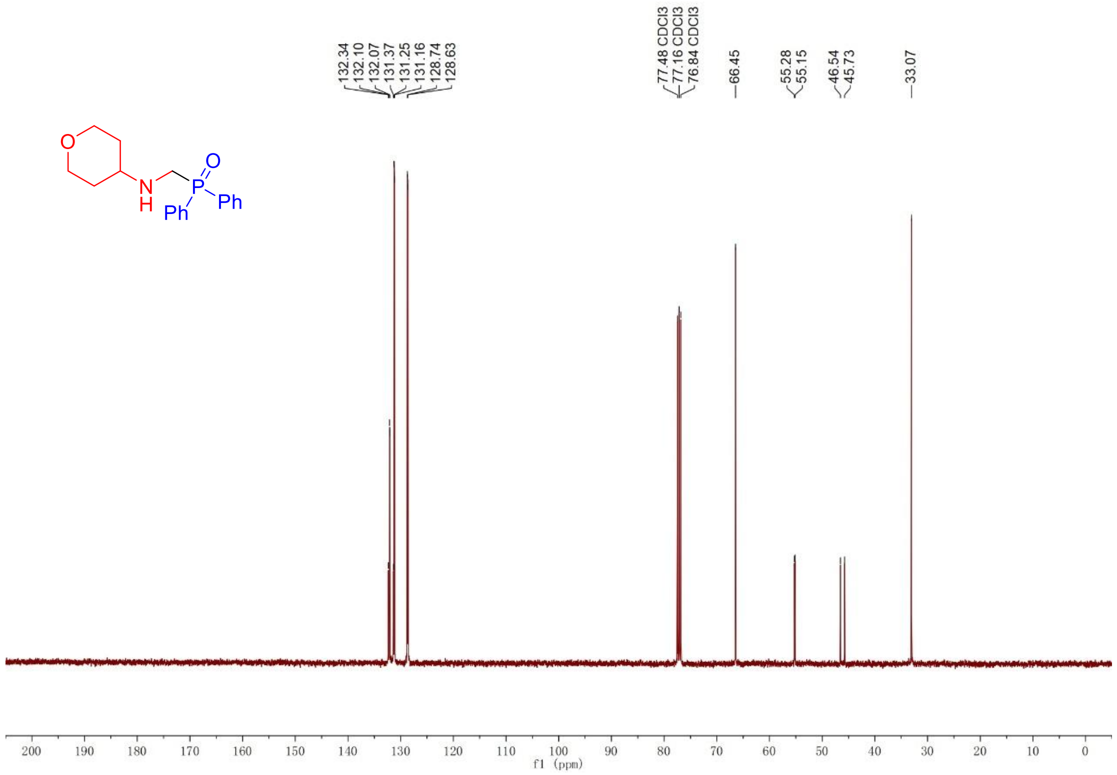

Figure S140. ^{31}P NMR spectra (162 MHz, Chloroform-*d*) of Diphenyl(((tetrahydro-2*H*-pyran-4-yl)amino)methyl)phosphine oxide (3na).

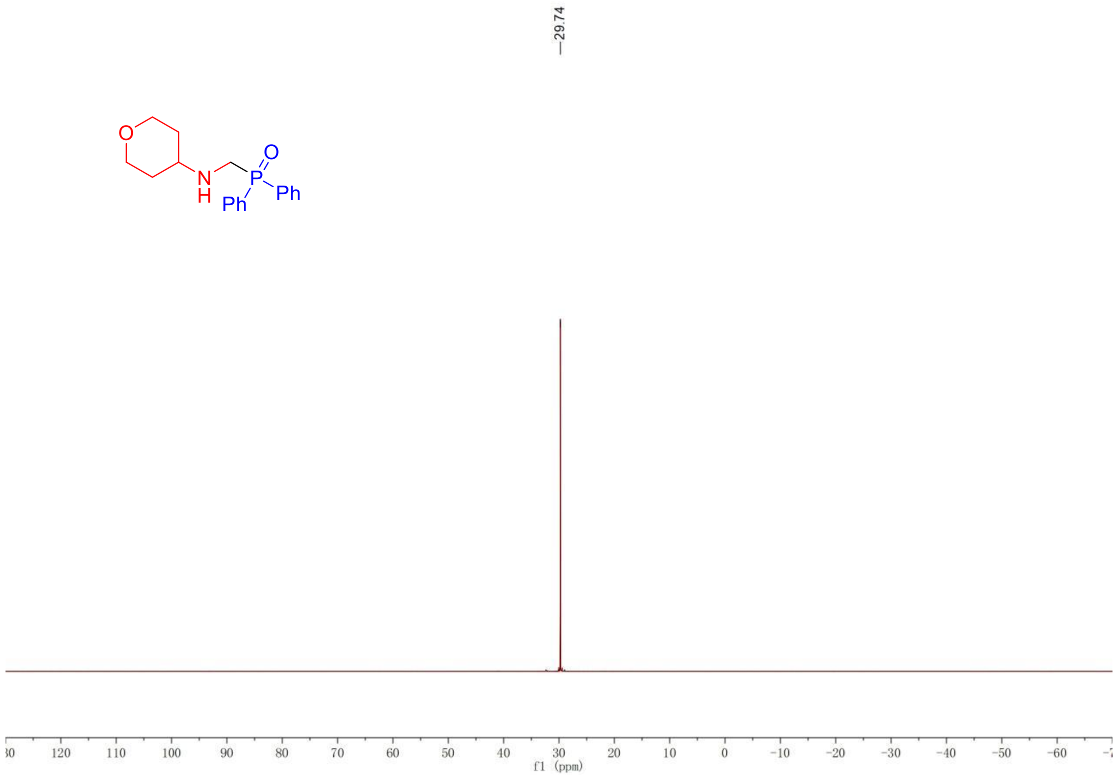

Figure S141. ^{1}**H NMR spectra (400 MHz, Chloroform-*d*) of (((3-(10,11-Dihydro-5*H*-dibenzo[*a*,*d*][7]annulen-5-ylidene)propyl)amino)methyl)diphenylphosphine oxide (3oa).**

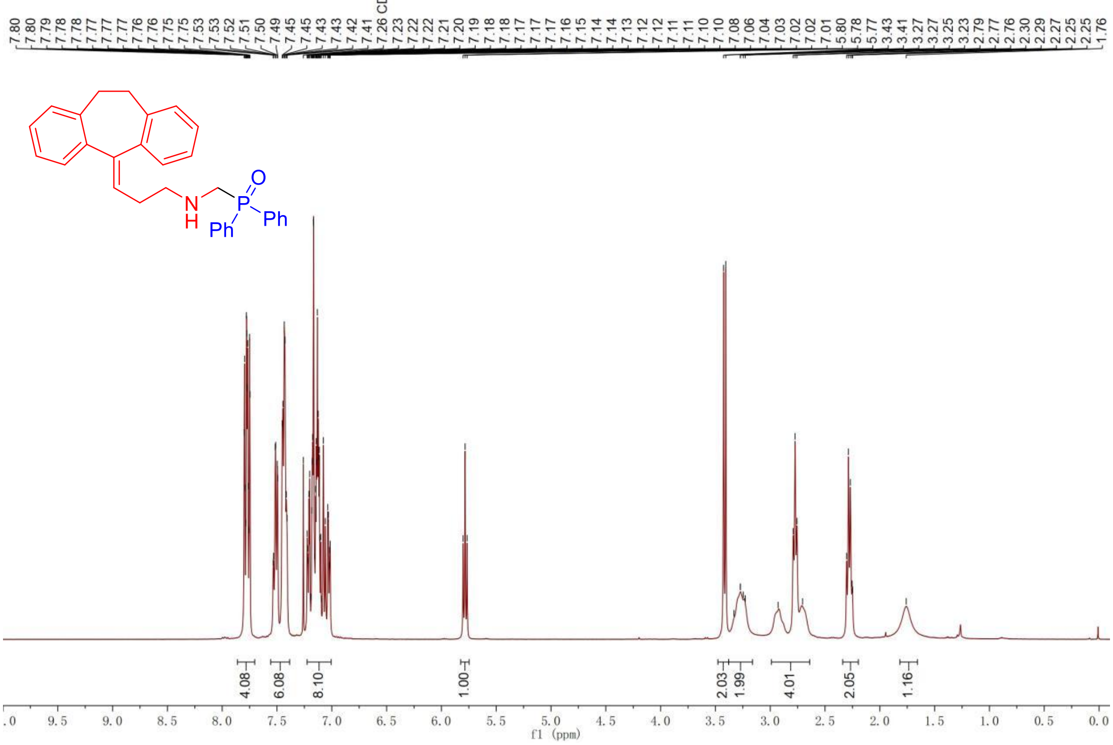

Figure S142. ^{13}**C{**^{1}**H} NMR spectra (100 MHz, Chloroform-*d*) of (((3-(10,11-Dihydro-5*H*-dibenzo[*a*,*d*][7]annulen-5-ylidene)propyl)amino)methyl)diphenylphosphine oxide (3oa).**

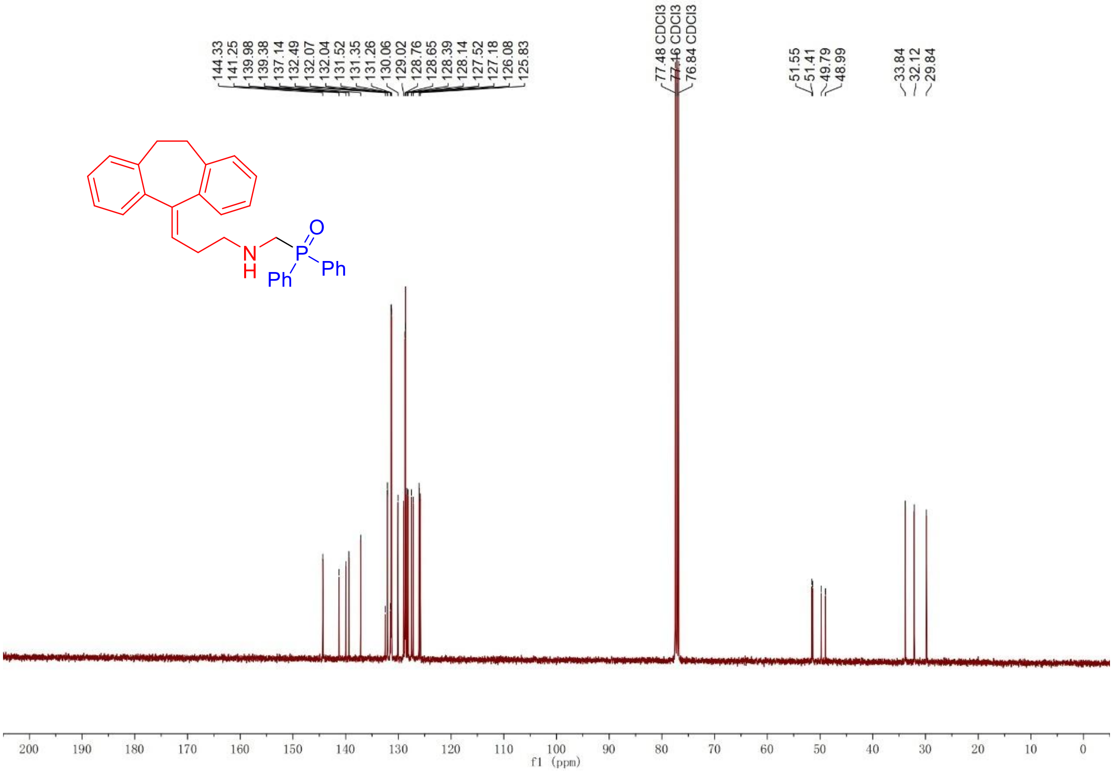

Figure S143. ^{31}**P NMR spectra (162 MHz, Chloroform-*d*) of (((3-(10,11-Dihydro-5*H*-dibenzo[*a*,*d*][7]annulen-5-ylidene)propyl)amino)methyl)diphenylphosphine oxide (3oa).**

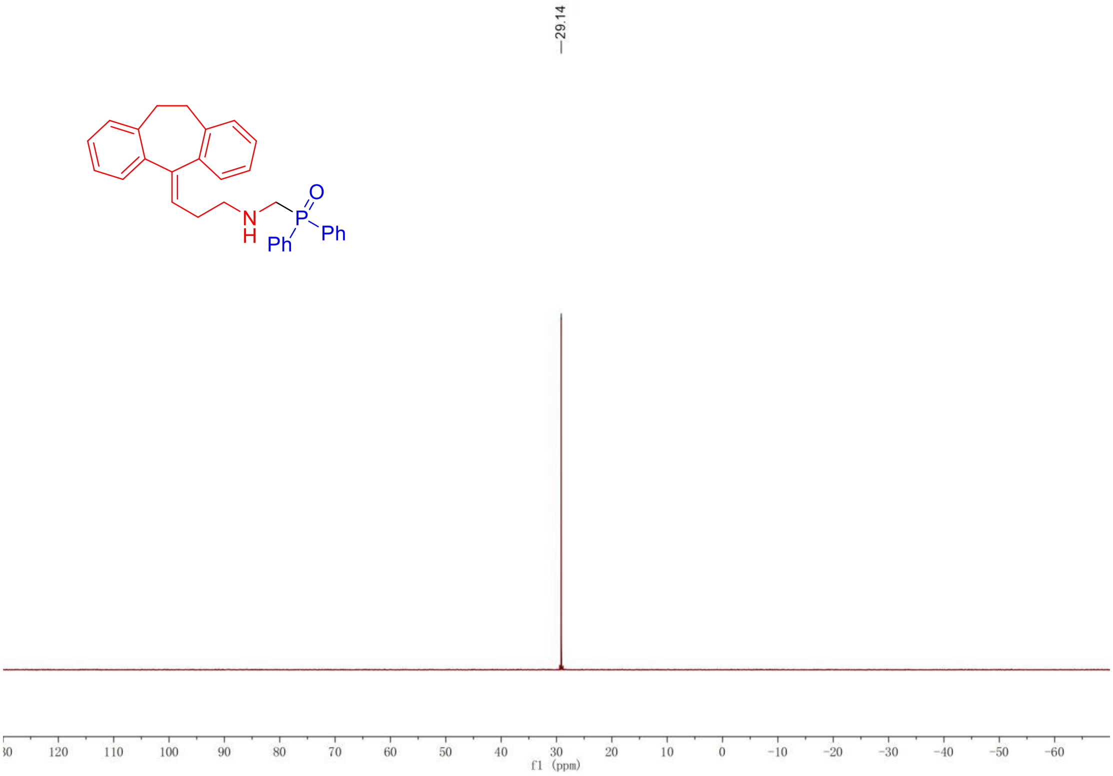

Figure S144. ^{1}**H NMR spectra (400 MHz, Chloroform-*d*) of Diphenyl(((3-phenyl-3-(4-(trifluoromethyl)phenoxy)propyl)amino)methyl)phosphine oxide (3pa).**

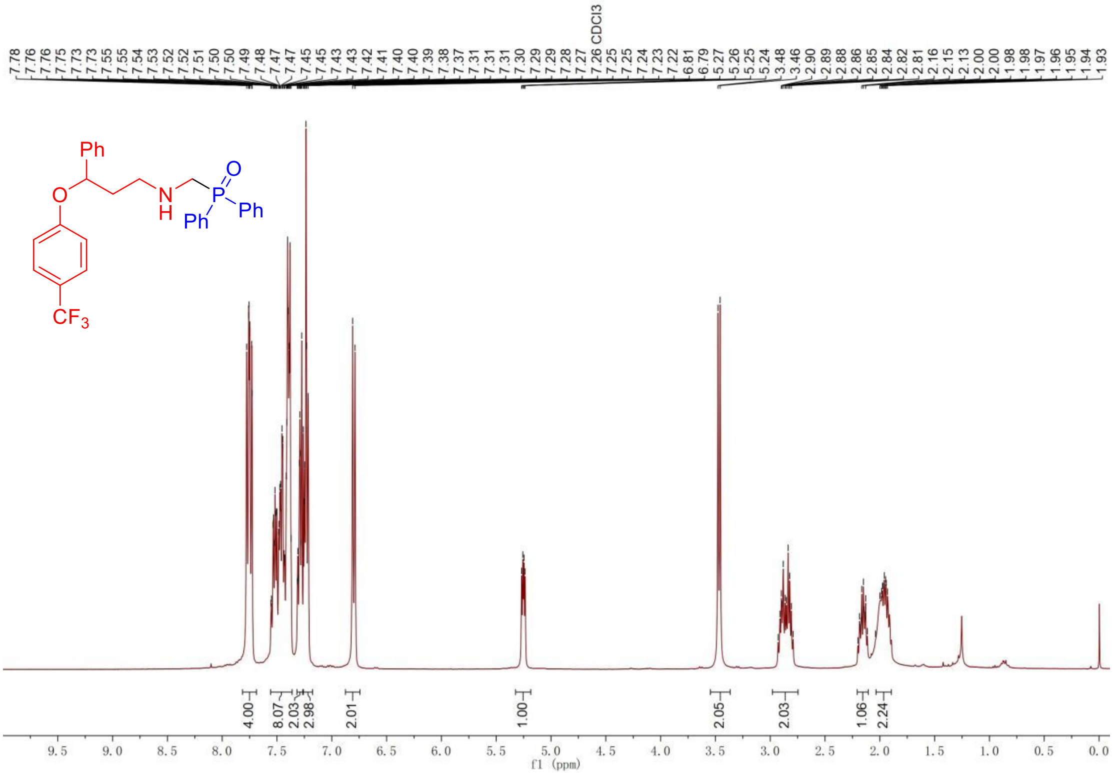

Figure S145. $^{13}C\{^{1}H\}$ **NMR spectra (100 MHz, Chloroform-*d*) of Diphenyl(((3-phenyl-3-(4-(trifluoromethyl)phenoxy)propyl)amino)methyl)phosphine oxide (3pa).**

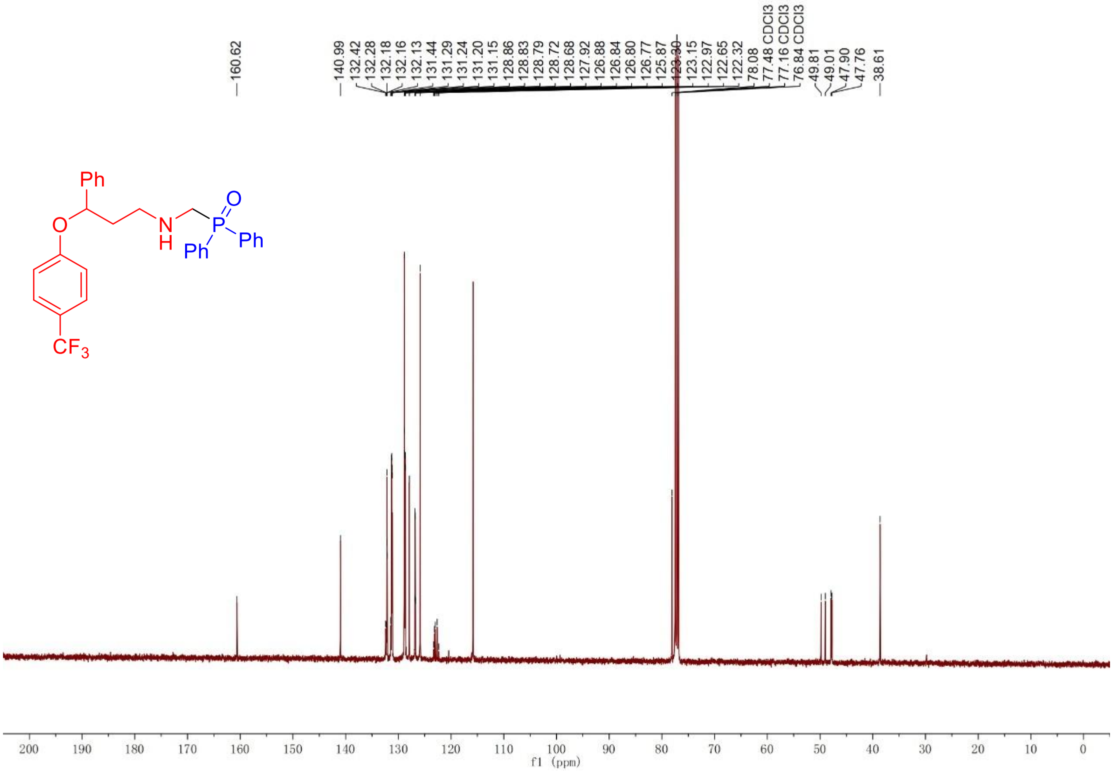

Figure S146. ^{31}P **NMR spectra (162 MHz, Chloroform-*d*) of Diphenyl(((3-phenyl-3-(4-(trifluoromethyl)phenoxy)propyl)amino)methyl)phosphine oxide (3pa).**

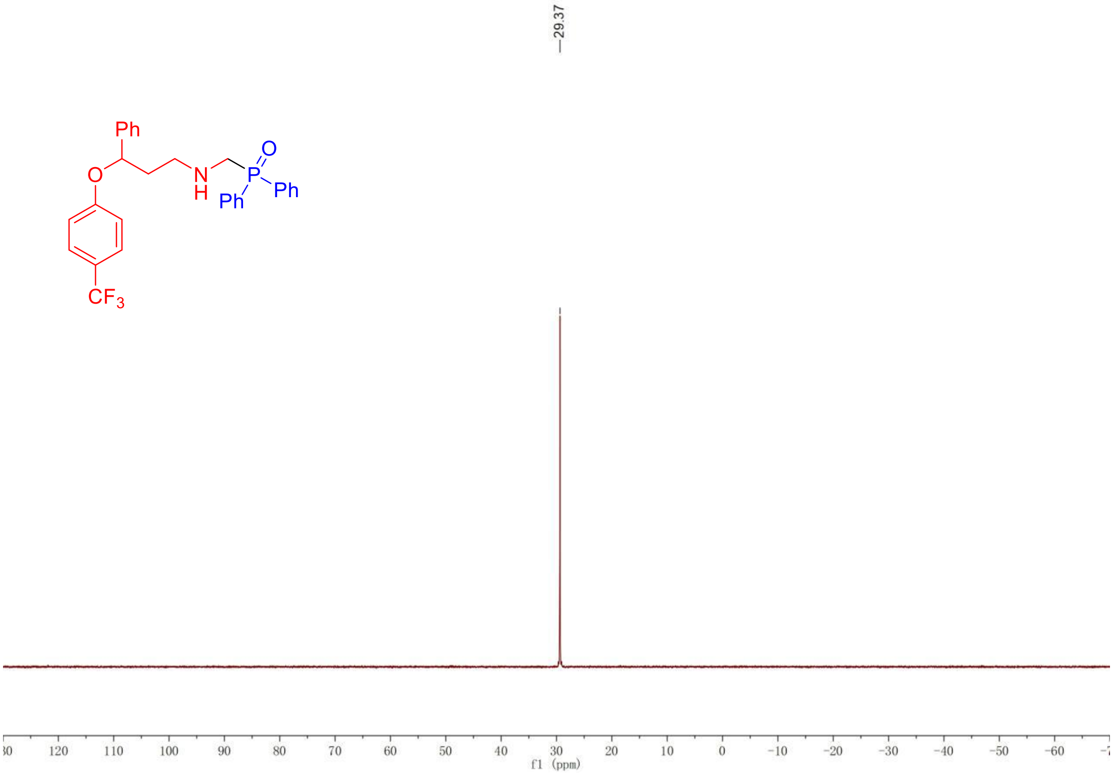

Figure S147. ^{19}**F NMR spectra (376 MHz, Chloroform-*d*) of Diphenyl(((3-phenyl-3-(4-(trifluoromethyl)phenoxy)propyl)amino)methyl)phosphine oxide (3pa).**

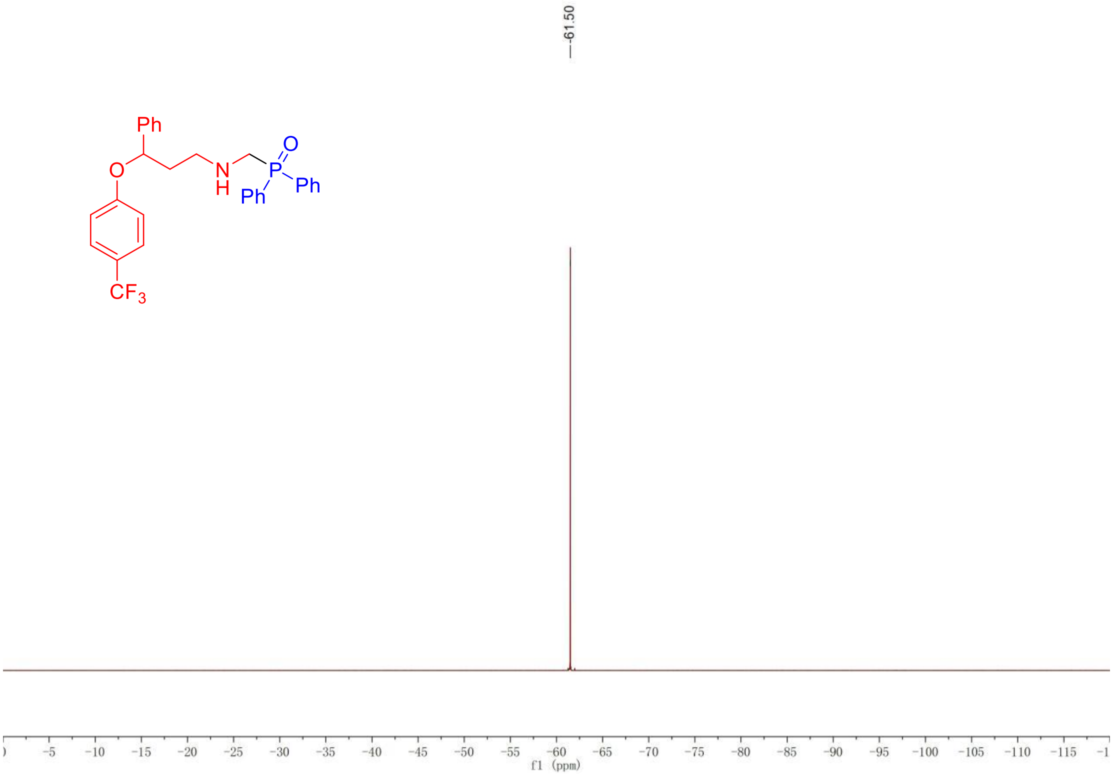

Figure S148. ^{1}**H NMR spectra (400 MHz, Chloroform-*d*) of Diphenyl(1-(phenylsulfonyl)pyrrolidin-2-yl)phosphine oxide (4ba).**

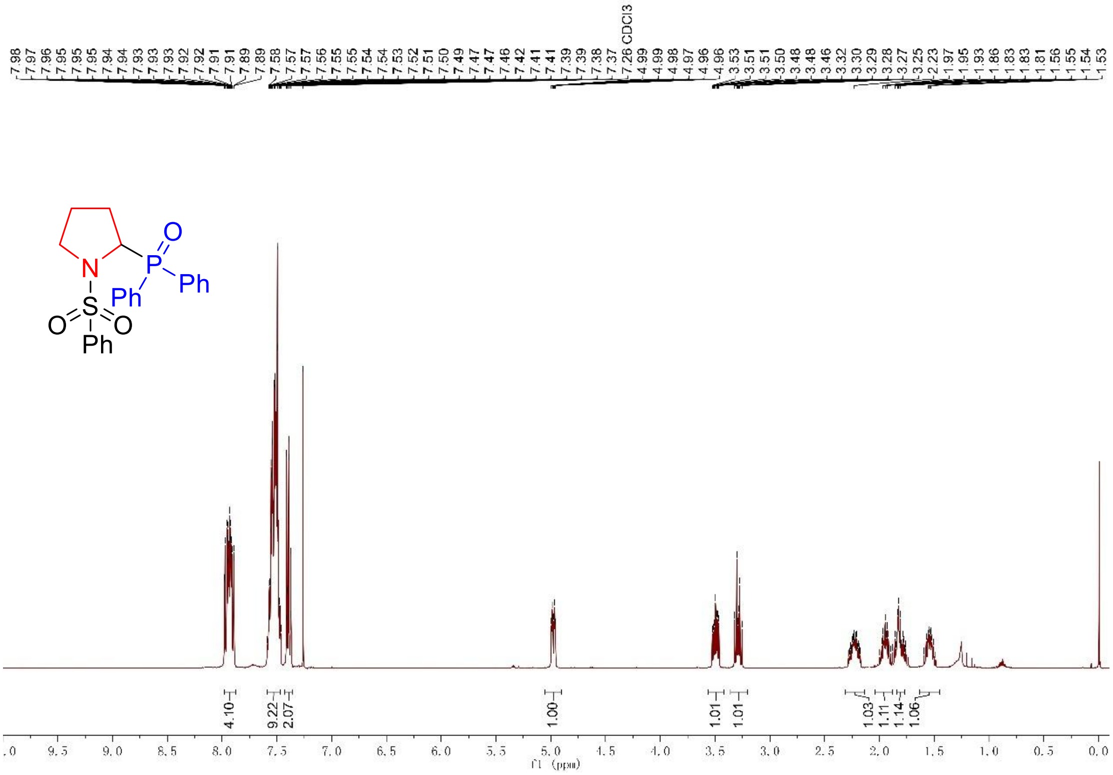

Figure S149. $^{13}C\{^{1}H\}$ NMR spectra (100 MHz, Chloroform-*d*) of Diphenyl(1-(phenylsulfonyl)pyrrolidin-2-yl)phosphine oxide (4ba).

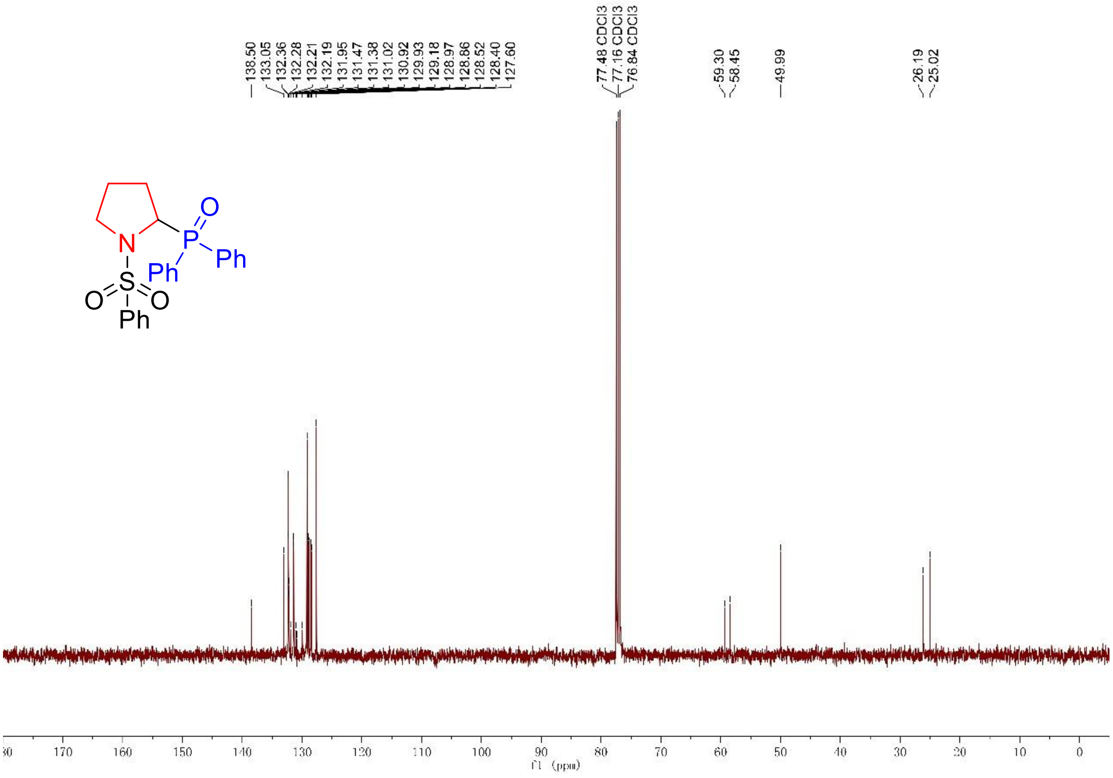

Figure S150. ^{31}P NMR spectra (162 MHz, Chloroform-*d*) of Diphenyl(1-(phenylsulfonyl)pyrrolidin-2-yl)phosphine oxide (4ba).

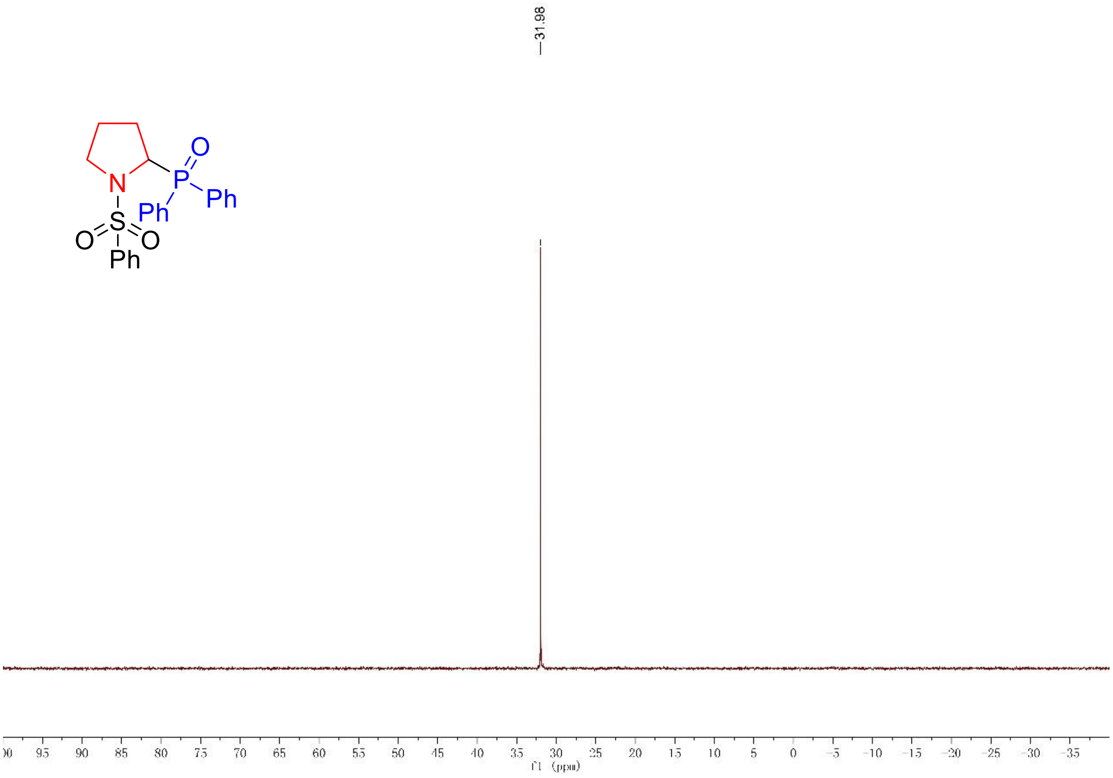

Figure S151. **^{1}H NMR spectra (400 MHz, Chloroform-*d*) of *N*-Methyl-3,3-diphenylpropan-1-amine (4ia).**

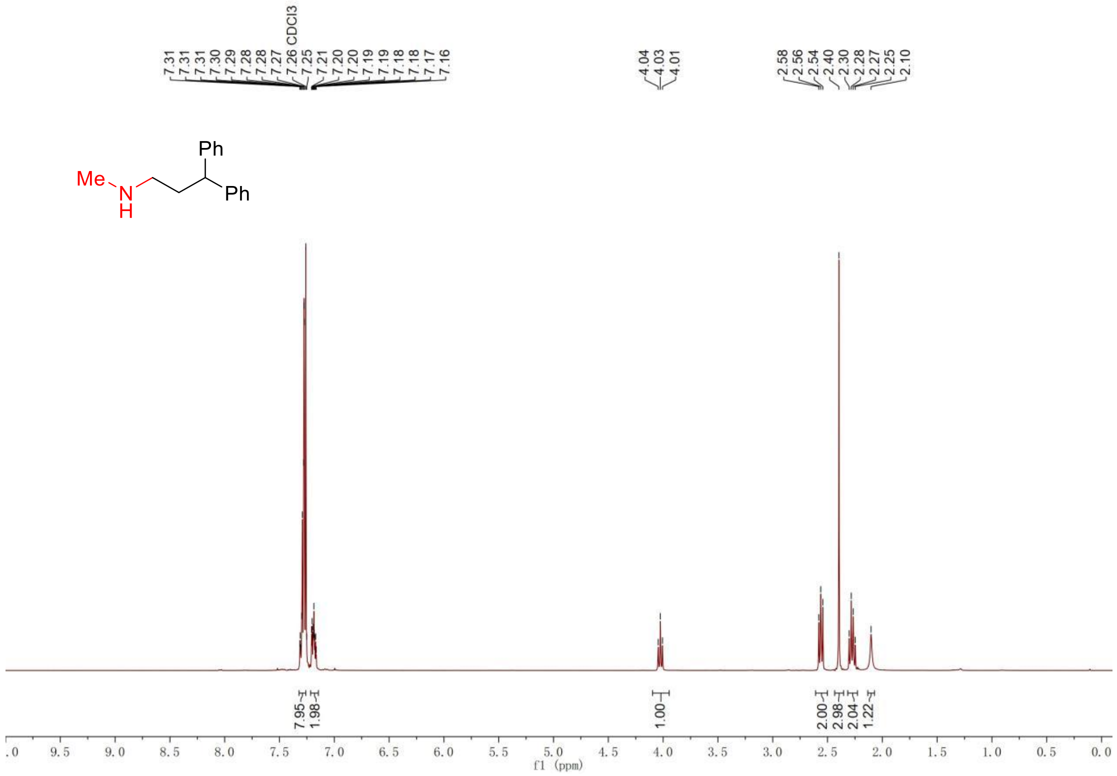

Figure S152. **^{13}C{^{1}H} NMR spectra (100 MHz, Chloroform-*d*) of *N*-Methyl-3,3-diphenylpropan-1-amine (4ia).**

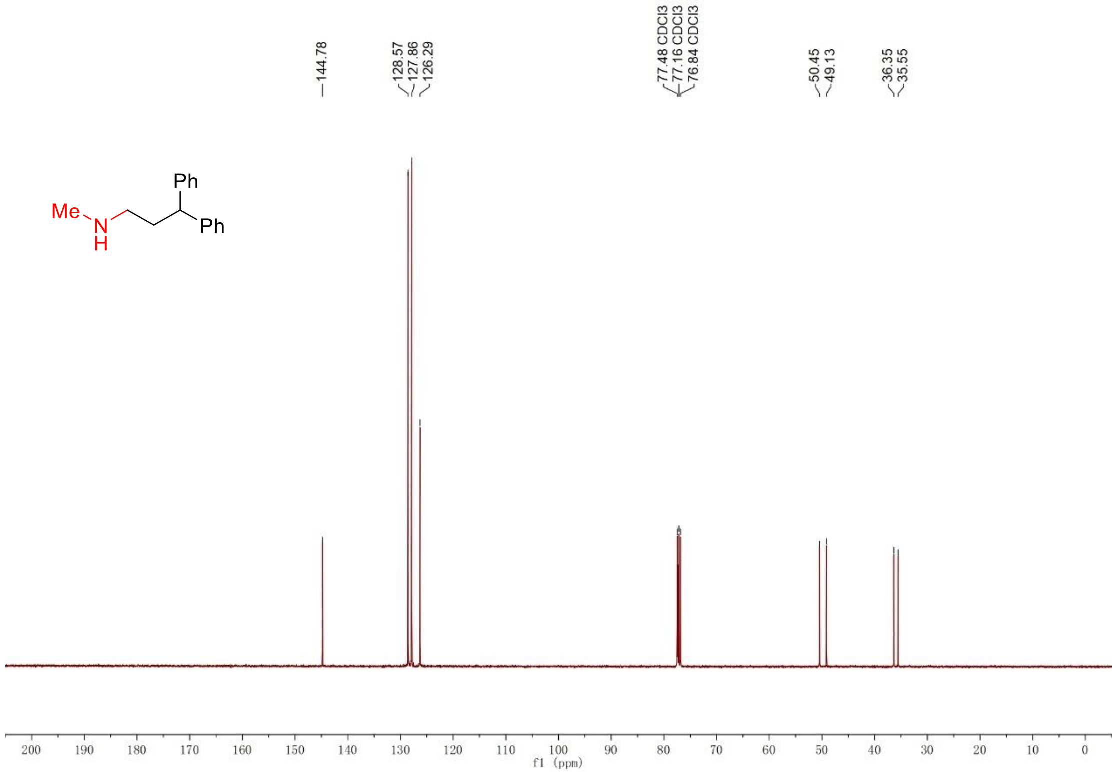

Supplement: SC-017-D5SC00268K-s002 [file SC-017-D5SC00268K-s002.pdf]
